# Perceptions of cervical cancer and motivation for screening among women in Rural Lilongwe, Malawi: A qualitative study

**DOI:** 10.1371/journal.pone.0262590

**Published:** 2022-02-07

**Authors:** Agatha K. Bula, Fan Lee, John Chapola, Clement Mapanje, Mercy Tsidya, Annie Thom, Jennifer H. Tang, Lameck Chinula

**Affiliations:** 1 UNC Project Malawi, Lilongwe, Malawi; 2 Department of Obstetrics and Gynecology, University of North Carolina, Chapel Hill, Chapel Hill, North Carolina, United States of America; 3 Department of Obstetrics and Gynecology, College of Medicine, University of Malawi, Zomba, Malawi; Aga Khan University, PAKISTAN

## Abstract

**Introduction:**

Cervical cancer is the leading cause of cancer death among women in Malawi. Low awareness of cervical cancer and negative perceptions of screening can prevent women from participating in preventative strategies. We sought to explore perceptions and motivations for screening among women who participated in a cervical cancer screen-and-treat pilot study in rural Malawi.

**Materials and methods:**

We conducted a qualitative sub-study of a community-based cervical cancer screen-and-treat pilot study in rural Lilongwe between July-August 2017. From October 2017-February 2018, 17 women who underwent screening using visual inspection with acetic acid (VIA) and same-day thermal ablation treatment were recruited at their 12-week follow-up visit post treatment to participate in this qualitative sub-study. Semi-structured interview guides that explored baseline knowledge of cervical cancer, perceptions, and motivation for screening were used for in-depth interviews (IDIs). IDIs were conducted in the local language, Chichewa, translated and transcribed to English. Data was analyzed using NVivo^®^ V12.0.

**Results:**

Findings included fatalistic views on cancer, but limited knowledge specific to cervical cancer. Misconceptions of cervical cancer screening were common; however, there was a unique understanding of screening as prevention (i.e., finding and treating early disease to prevent progression to worsening disease). This understanding appeared to stem from HIV prevention concepts known to the community. Motivations for screening included desire to know one’s health status, convenience of community-based screening, and peer encouragement.

**Conclusion:**

Despite limited knowledge of cervical cancer and misconceptions of screening, the concept of screening for prevention, desire to know one’s health status, convenient access, and peers’ influence were motivators for participation in screening. Cervical cancer screen-and-treat programs in high HIV prevalence areas should consider utilizing language that parallels HIV prevention language to communicate the need for cervical cancer screening and treatment and utilize prevention concepts that may already be familiar to women living there.

## Introduction

Cervical cancer is the leading cause of cancer-related deaths among women in Sub-Saharan Africa (SSA) [1–3]. It disproportionately affects low and middle-income countries (LMIC) where HIV burden is high and cervical cancer screening coverage is low [4, 5]. Malawi has the highest age-standardized mortality rate from cervical cancer in the world, estimated at 54.5 per 100,000 persons in 2018 [6]. The HIV prevalence rate in Malawi is also among the highest at 10.8% in women aged 15–49 [7]. Furthermore, the national cervical cancer screening program is challenged by limited infrastructure and poor access to screening and treatment [3, 8, 9] particularly in rural communities, where 84% of Malawi’s population live [10, 11].

Knowledge of the disease and understanding of preventative screening are also central to uptake of screening services, which remains limited in communities at greatest risk of disease. Several studies conducted in SSA have demonstrated low knowledge of cervical cancer screening among women as the main barrier to uptake of cervical cancer screening [12–15]. Similarly in Malawi, studies have shown that barriers to screening among women include low knowledge and low perceived susceptibility of cervical cancer, coupled with stigma and misconceptions about screening [16, 17]. In addition to increasing screening access and sustainable treatment options, it is also essential to understand women’s baseline knowledge, perceptions, and motivation for screening, to facilitate community interventions that can successfully reduce morbidity and mortality of cervical cancer.

Malawi currently utilizes the WHO-endorsed screen-and-treat strategy with visual inspection with acetic acid (VIA) and same-day ablative treatment of eligible VIA-positive lesions [18]. However, between 2011–2015, the national cervical cancer screening program had low uptake with high loss-to-follow-up after positive screens: only 27% of eligible women were screened, and over half of those requiring ablative treatment did not receive it [8]. Challenges identified included inability to sustain functional cryotherapy equipment for treatment and difficulty for women in predominantly rural communities to access the facilities where screening was offered [8]. To address these challenges, in 2017 we implemented a community-based screening pilot study utilizing thermal ablation (over cryotherapy) for same-day treatment of VIA-positive lesions in rural Lilongwe District, Malawi [19].

To gain in-depth understanding of Malawian women’s baseline knowledge of cervical cancer, perceptions, and motivations for screening, we conducted a qualitative sub-study to evaluate the experiences of women who underwent VIA and same-day thermal ablation treatment through this community-based study.

## Methods

### Study design and setting

This qualitative sub-study included women who underwent same-day VIA and thermal ablation in the community-based cervical cancer screen-and-treat pilot study, which was conducted between July and August 2017 in 4 villages (Kunthulu, Chala, Chisembwere and Mpingu) in rural Lilongwe District [19]. The four villages were greater than 20 km away from the closest hospitals that offered free cervical cancer screening. We aimed to conduct in-depth interviews with participants who completed both VIA and thermal ablation treatment at their post-procedure follow-up appointments. We aimed to interview both participants who presented for their scheduled visits and those who did not present and required tracing to reschedule.

Prior to initiating cervical cancer screening and data collection for the pilot program, education outreach was conducted about cervical cancer screening and provision of same day thermal ablation to those with VIA positive lesions through community meetings. Women who presented at the screening site were assessed if they meet the inclusion criteria for the pilot study. The pilot study population included non-pregnant women aged 25–49 years, with no history of hysterectomy or lower genital dysplasia or cancer, and who had not undergone cervical cancer screening in the past year. Visual Inspection using Cyclic Acid (VIA) was performed to those women who provided informed consent. Participants who underwent thermal ablation after a positive VIA screen were asked to come for follow-up visits at 6 and 12 weeks post-treatment at UNC Project-Malawi Tidziwe Center, which is located at Kamuzu Central Hospital (KCH), a public teaching hospital in Lilongwe.

### Study participants for the qualitative sub-study

Women for this qualitative sub-study were recruited from the 28 women who underwent thermal ablation and therefore were scheduled to attend a 12-week follow-up visit. We planned to interview up to 10 participants who attended their 12-week follow-up visit and up to 10 participants who missed their 12-week follow-up visit to capture differences in experiences and barriers to care. Those who did not attend the 12-week visit were traced by study staff and their visits rescheduled. Participants received transport reimbursement both for the follow-up visits and participation in this sub-study. We ultimately interviewed a total of 17 participants: 10 of the 18 who attended the initially scheduled 12-week visit, and 7 of the 10 who missed their initially scheduled 12-week appointments but were successfully traced and agreed to participate in the sub-study.

### Data collection

One-on-one in-depth interviews (IDIs) were conducted between October 2017 to February 2018. The rationale for using IDIs was to better understand, and deeply explore women’s knowledge, attitudes, regarding cancers in general, cervical cancer and cervical cancer screening as well as barriers to come for follow up visits. Two separate but similar semi-structured in-depth interviews guides were used, one for participants who attended their initially scheduled follow-up visits (S1 File) and the other for participants who missed these visits (S2 File). The interview guides contained 7 domains: 1) baseline knowledge of cervical cancer; 2) perceptions of cervical cancer screening; 3) screen-and-treat experience; 4) acceptability of pilot screen-and-treat program; 5) follow-up challenges; 6) community and partner support; and 7) attitudes towards self-collection vaginal swabs for HPV testing as alternative screening method. The IDIs were conducted in Chichewa, the local language by two project research offers (MT and AT), who are fluent in both English and Chichewa and experienced in qualitative research methods. They were trained about the study protocol and standard operating procedures (SOPs) related to the conduct of the study. Each IDI lasted an average of 60 minutes. The IDIs were audiotaped, translated and transcribed into English for analysis (S3 File). IDIs were conducted in a private place within Tidziwe Center, Kamuzu Central Hospital. To ensure the accuracy of the data collected, the researchers conducted checks of the data collected, and feedback was given to the interviewers on topics that needed further probing and areas that needed improvement.

### Data analysis

We began analysis by reading and re-reading the transcripts concurrently with data collection to familiarize ourselves with the content and also identify themes that needed follow up. Using an inductive approach, the team members developed the initial codebook using pre-defined themes, then continued to add themes from the data during the coding process. Each interview was then double coded by three coders (FL, AB, JC) using NVivo® version 12.0. The three coders had a weekly meeting to review and reconcile all areas of discrepancy until complete agreement of the coded text was reached. Content analysis was performed using NVivo® version 12.0 and in-code memos between transcriptions were used to assure reliability of coding and validity of findings. Coders used Microsoft Excel and constructed matrices to summarize themes and collate them by participant characteristics. All themes were well saturated after 17 IDIs. In this paper, we present findings from analyses of domains 1 and 2.

### Ethical considerations

This study was reviewed and approved by the National Health Sciences Research Committee of Malawi (NHSRC) and the University of North Carolina (UNC) at Chapel Hill Institutional Review Board. Further approvals were also obtained from the village local authorities and the Lilongwe District Health Office. All women provided a written informed consent before participation in the IDIs.

## Results

### Background characteristics of participants

Most of the 17 women interviewed were between 30–40 years of age, and the majority had attended only some primary school (Table 1). Almost all women were married, but 4 (24%) reported being married in a polygamous relationship. Furthermore, almost all participants were not employed and had no access to tap water and electricity. All participants interviewed were HIV negative, and the majority reported that their partners had additional sexual partners (Table 1).

**Table 1:** Demographic characteristics of participants (N = 17).

Age (years)	n (%)
20–29	2 (12)
30–39	8 (47)
40–49	6 (35)
50–59	1 (6)
**Level of education**	
No formal	4 (24)
Primary school	12 (70)
Secondary school	1 (6)
**Marital status**	
Married monogamous	11 (65)
Married polygamous	4 (24)
Other	2 (12)
**Total lifetime partners**	
1 partner	6 (35)
2–3 partners	10 (59)
≥4 partners	1 (6)
**Employment**	
House wife	10 (59)
Business	1 (6)
Farming	4 (24)
Unskilled labor	1 (6)
Teacher	1 (6)
**Running water in home**	
No	17 (100)
**Electricity in home**	
No	16 (94)
Yes	1 (6)

### Baseline knowledge and fatalistic perception about cervical cancer

The majority of participants reported knowing very little about cervical cancer disease. The most common sources of information about cervical cancer were through the radio, from friends who were screened or through family planning clinics. While most women reported not knowing specifics about cervical cancer, there was a shared fatalistic view towards cancer in general.

*“I heard nothing [about cervical cancer] before health workers came to our village*. *I was anxious and afraid since I knew that cancer is a deadly disease and has no cure.” (Participant #269)*

Some derived these fatalistic views from experiences with others in their communities who were diagnosed with cancer:

*“I have seen an old woman die of cancer…she started complaining that her leg was hurting… .by they time she went to the hospital*, *the leg was already rotten, and the doctors diagnosed it as cancer…so she died.” (Participant #385)*

Others described even more personal experiences with loved ones:

*“My mother suffered from cancer*. *She was discharging watery fluids, and they told us that we delayed seeking health care. I was with her [at [the hospital] until she died. My last born also died of cancer when he was eight years. The doctors reached the extent of telling me that ‘there is nothing we can do,’ I went home on Wednesday, and the next Wednesday the boy was no more” (Participant #351).*

Both these participants described what appeared to be late presentation of cancer when treatment options were limited or futile. Therefore, it is not surprising that fear of cancer diagnosis was reported among many participants. In addition to decreased survival, many participants also described worry about prolonged suffering due to cancer and how the debilitating state of the disease can affect their families. For example, this participant expressed concern that her children may not receive care and financial support if she was diagnosed with cancer:

*“I am worried that if I am found with the disease (cancer) I shall be in trouble*. *Even my children as well, for it takes some time while you are still struggling with the disease before you die… sure that was my fear.” (Participant #293)*

Some shared personal stories of cancer and loss. This participant described losing both her mother and son to cancer, also touching on the concept of futility in seeking care too late:

*“My mother suffered from cancer*. *She was discharging watery fluids, and they told us that we had delayed in seeking health care. I was with her at Ethel [Ethel Mutharika Maternity Wing] until when she died. My last born also died of cancer when he was eight years…The doctors reached the extent of telling me that ‘there is nothing we can do,’ I went home on Wednesday and the next Wednesday the boy was no more” (Participant #351).*

Participants also expressed a concern that due to lack of knowledge about cervical cancer, concerning signs and symptoms can be missed. This suggests that increased education can perhaps lead to earlier diagnosis:

*“I have learned that cervical cancer makes others discharge water and blood from the vagina*. *Initially I would attribute such problems to other diseases without knowing what really it was.” (Participant #245)*

### Perceptions of cervical cancer screening

Most participants reported that prior to undergoing cervical cancer screening, they had limited understanding of the screening process. Myths and misconceptions of the screening process were common, including painful exams, fear of receiving a positive screening result and stigma surrounding intention of specimen collection by healthcare workers. The perception that cervical cancer screening is painful or uncomfortable appeared to stem from community experiences with family planning procedures or other gynecological exams:

*“People were scaring [my sister] that the metal [inserted for screening] is painful*.*” (Participant #370)*

Another participant reported that she had heard that the screening procedures was using *“spanners” and “pulling the cervix as is the case when doing sterilization.” (Participant #207)*.

Fear of a positive screening diagnosis stemmed from three main concerns: 1) a positive screen meant a diagnosis of cancer, and cancer was perceived as fatal (*“They think if you’re found VIA positive that means it’s a death sentence*.*” Participant #240);* 2) treatment involved removing the uterus (“*They would take out the uterus to treat it*.*” Participant #116)*; 3) surgical treatment is risky (*“My other worry was that after they have removed the cervix*, *will I still be alive*? *Won’t I end up leaving my children hopeless*?*” Participant #207)*. Less commonly mentioned, the stigma of a positive screen was also reported and compared to that of HIV:

*“It is the same as HIV*, *when someone has been found with it, instead of encouraging them [to get treatment], you find that others even ridicule them.” (Participant #395)*

Lastly, distrust of healthcare workers was a common theme reported by participants in their communities. Several participants referred to the ‘blood sucker’ myths that healthcare workers are vampires who suck the blood from participants for satanic purposes as a barrier to utilizing healthcare facilities for screening. One participant reported:

*“They were saying if I have cancer signs*, *they will deliberately make us go to the hospital so that they can be sucking my blood and eventually I die…they say it’s satanic.” (Participant #269)*

Another participant reported that healthcare workers sold the vaginal discharge they collected:

*“They say…those people who came were collecting vaginal discharge*, *and they were collecting from those who had sex…[then] they say they sell it at [the hospital].” (Participant #171)*

This statement reflects the myths in the community that healthcare workers collect blood, or in this case, vaginal discharge, to sell to foreigners for satanic rituals.

### Understanding prevention through HIV

Despite limited understanding of the cervical cancer and common misconceptions of cervical cancer screening in the community, participants demonstrated that they understood the need for screening–that diseases detected early can be treated to prevent fatality.

*“I have seen my people dying of cancer*, *and I thought the earlier the better, I should be screened and see how I can be assisted rather than being screened when there can be no solution to the problem.” (Participant #351)*

Many participants used the concept of HIV prevention to describe this concept of disease prevention and the importance of early screening:

*“I think such clinics [for cervical cancer screening] are important because cancer is just like HIV*, *you need to get tested for you to know your status. It is always difficult when you get tested at a late stage, but when you get tested in the early stages, it is better and you even receive counsel.” (Participant #385)*

Language from HIV/AIDS campaigns were also used directly. Many referred to wanting to “know one’s status” through cervical cancer screening, as HIV screening provides HIV status. However, in this case, “to know one’s status” is used to describe one’s health condition in general:

*“[I went for screening] so that I can know my status*. *I wanted to know how my body was.” (Participant #253)*

Participants also made direct comparisons of cancer to HIV disease, using AIDS as a gauge for the seriousness of the disease:

*“Cancer is deadly as compared to AIDS*.*” (Participant #269)*

*“Cervical cancer is a very deadly disease that everyone is afraid of it more than AIDS*.*” (Participant #233)*

## Motivation for screening

When participants were asked why they decided to undergo cervical cancer screening, three main motivation factors emerged: 1) desire to know their health status, 2) availability of screening services directly in their community, and 3) motivation from peers.

### a. The desire to know their cervical cancer “status”

The most common reason for participating in cervical cancer screening, expressed by over half of the participants, was wanting to know one’s health status:

*“Better to get screened so that you should know your status….I might assume that I am fine*, *and yet I have cancer spreading inside of me.” (Participant #207)*

*“I did that [screening] because I wanted to know the condition of my body, you can just be staying and never be certain you are okay or not. So*, *this time I thought it wise to go get screened.” (Participant #293)*

Most participants further shared a sentiment that early diagnosis and treatment was important to prevent further progression of a disease, and reported this as a motivating factor for participating in cervical cancer screening:

*“I wanted to get early treatment, because we were told that if detected early, you can get treated on the spot. So that encouraged me to do screening so that I get treated early.” (Participant #239)*.

### b. Availability of services within the community

Participants also reported that bringing screening directly to their village facilitated their ability to access services. For example, this participant was motivated to attend screening despite hearing about fears surrounding a speculum exam:

*“For me*, *I felt that it was a very precious thing being visited by doctors in our village and for me to know my condition despite the talks by other people who were saying: ‘why should I have metals inserted into my genital’.” (Participant #269)*

Some participants reported to have experienced concerning symptoms such as vaginal bleeding, backache and abnormal vaginal discharge. They further explained that the nearest health facilities were located a considerable distance from their villages, and most do not offer screening services. This participant experienced abnormal vaginal discharge and was concerned this may be a sign of cervical cancer as she had heard about it on the radio; however, she was unable to find a facility for screening:

*“Our hospital is just so small*, *and they don’t have the equipment to perform such tests. So, we kept searching for a place where we could get screened…When we heard that the service has come close to us, we just praised God because he had made a way for people to know their status.” (Participant #385)*

### c. Motivation from peers

Lastly, many participants described encouragement from peers who had undergone screening. They recounted how their peers provided valuable information about cervical cancer, shared their own experiences with the screening procedures, and the advantages of being screened for cervical cancer. This was evident through the increased number of women that presented for screening during the last few days of the campaign as they were waiting to get firsthand information and experience from other women in their village. One participant explained:

*“We were wondering "what are we going to face inside [the screening room]… later one colleague comes out and she explains to us everything*. *Thereafter we all gathered courage and determined to go in, since we want a healthy life.” (Participant #207)*

## Discussion

This paper explored baseline knowledge of cervical cancer, perceptions, and motivation for screening among women in rural communities in Malawi. Overall, our findings demonstrate lack of baseline understanding of cancer in general and cervical cancer screening among our participants. The lack of baseline knowledge is consistent with findings from a study conducted in Kenya which showed that almost all participants (91%) had heard of cancer, but only 29% had heard of cancer specific to the cervix [20]. Similar findings were also reported in a study in Southern Ghana which showed that 68.4% of the sampled population had never heard about cervical cancer [13].

Our results further show that many participants shared personal anecdotes of caring for, or hearing about, family and/or friends who suffered and ultimately died from cancer. This familiarity with and fatalistic view of cancer reflects the high burden and low survival rate of those diagnosed with cancers in Malawi, as diagnosis is often made at advanced stages. A retrospective study conducted at NdiMoyo Palliative Care Center in central Malawi revealed that the median survival time from diagnosis for the 5 top cancers among adults (cervix, Kaposi’s sarcoma, esophageal, breasts and liver) was 9 months, and the survival rate was especially low for cervical cancer at 2.9% at 4 years or more [21]. The fatalistic view of prolonged suffering and inevitable death after cancer diagnosis no doubt cause cancer-related fear that prevent uptake of cervical cancer screening. This was evidenced in our results where most participants interviewed reported to have never been screened for cervical cancer before the community-based screening campaign. This is also supported by similar findings from several studies conducted earlier in Malawi and other countries in SSA [13, 22–25] that demonstrated that knowledge of cervical cancer was associated with utilization of cervical cancer screening, which is critical in cervical cancer prevention. There is need for community sensitizations programs to increase awareness about cervical cancer and importance of screening to facilitate uptake of cervical cancer screening leading to early detection of precancerous cells. This would likely motivate women to undergo screening and lead to earlier detection of cervical dysplasia and cancer.

Our participants reported many myths and misconceptions of cervical cancer screening, including fear regarding removal of uterus and instruments used to conduct pelvic exams, as well as distrust in healthcare workers and suspicion of specimen collection. These barriers demonstrate inadequate understanding of the screening procedures that providers need to be aware of and which need to be addressed when giving health education messages. These findings are consistent with findings from other studies conducted in Uganda, which also found myths and misconceptions related to cervical cancer screening, such as removal of their ovaries and/or uterus and cutting off of some flesh [26]. The ‘blood sucker’ myth that several participants referred to has been propagated in Malawi for generations and has led to fatal incidences of hysteria and mob violence in recent years [27]. Collection of vaginal discharge for satanic purposes has been incorporated into the myth. It is therefore important that such myths and misconception are taken into consideration when designing and implementing cervical cancer screening services within similar settings in the country.

Despite limited knowledge of cervical cancer, fatalistic views towards it, and misconceptions of screening, the concept of screening for prevention, desire to know one’s health status, convenient access, and peers’ influence were motivators for participation in screening. Most of our participants demonstrated a keen awareness of the concept of preventative care, that screening detects early, often asymptomatic disease that can then be treated to prevent serious health consequences. This understanding was echoed in almost all participants, consistent with another study conducted in the country [14]. Language and the concept of screening to “know one’s status” appeared to be borrowed from HIV campaigns [28] to describe the importance and acceptance of screening as routine health surveillance. While HIV/AIDS was used directly as a gauge for deadly disease in describing the fatalistic views of cancer, it was also used to draw parallels for the need for routine screening, prevention, and early treatment, even among our participants who were all HIV negative. Another study in Malawi found that HIV-infected women reported increased self-perceived risk for cervical cancer, which acted as a cue to action for screening [14]. But in this study, HIV educational activities appeared to have also taught HIV-uninfected women about the importance of health screening of all types. Further work may seek to better understanding this unique and successful health messaging in settings where HIV prevalence is high and HIV education is widespread.

The study also revealed that some women participated in our screening program because they felt at risk for cervical cancer or experienced concerning symptoms, such as abnormal vaginal bleeding and discharge, having the screening done within their communities. This finding about perceived susceptibility to cervical cancer is similar to findings from a study conducted in southwest Ethiopia and studies conducted in Kenya, which found perceived susceptibility for cervical cancer as the main factor that Increased cervical cancer screening utilization [26, 29–31]. Other participants on the other hand, expressed gratitude that the campaign *“brought the services close to their homes” (Participant #385*, *age 39)* and created an opportunity for women to overcome some of the challenges they face, including transport cost and waiting time at health facilities. Long travel distance to health facilities is a known barrier to screening in SSA [32, 33]. Lastly, many participants also reported being encouraged after hearing about screening from peers and community members who underwent screening. Participants themselves reported eagerness to take their screen-and-treat experiences back to the community and share them with others to counter the stigma and misconceptions of screening. Furthermore, peer support and the importance of interpersonal relationships in promoting screening and prevention is well documented in the literature [14, 15, 34].

Our study was limited by the fact that it only included the views of women who participated in the community-based cervical cancer screening, and we did not explore perceptions of women who did not undergo screening and thermal ablation. Nevertheless, our participants reflect similar demographic characteristics as women living in rural Lilongwe where uptake of cervical cancer screening is low due to similar challenges.

## Conclusion

Our study demonstrated that women who underwent the pilot community-based cervical cancer screening program in rural Lilongwe, Malawi had limited knowledge about cervical cancer screening. However, despite rumors and misconceptions, women were motivated to undergo screening because they wanted to know their health status. Cervical cancer screening and treatment within the community was also seen to help reduce fear, dispel misconceptions within the community, and provide convenient access for screening, which motivated participants to go for screening and educate others in the community about its importance. The positive views of community-based cervical cancer screen-and-treat by the study participants creates an opportunity for the Malawi Ministry of Health to scale it up, although it will require a collaborative effort with funders and implementing partners to reach these rural communities. Finally, cervical cancer screen-and-treat programs in high HIV prevalence areas should consider utilizing language that parallels HIV prevention language to communicate the need for cervical cancer screening and treatment and utilize prevention concepts that may already be familiar the community.

## Supporting information

S1 File 12 week follow-up qualitative interview guide18 Sep 201. (DOC)

S2 File Missed 12 week qualitative interview guide 11 Sep 2017. (DOC)

S3 File Via transcript 2017. (ZIP)

## References

[1] Ferlay J, Soerjomataram I, Dikshit R, Eser S, Mathers C, Rebelo M, et al. Cancer incidence and mortality worldwide: sources, methods and major patterns in GLOBOCAN 2012. International journal of cancer. 2015 Mar 1;136(5):E359–86. doi: 10.1002/ijc.29210 25220842

[2] GLOBOCAN. Estimated Cancer Incidence, Mortality and Prevalence Worldwide in 2018 2019 [cited 2020 10 June]. Available from: https://gco.iarc.fr/today/data/factsheets/populations/900-world-fact-sheets.pdf.

[3] Chidyaonga-Maseko F, Chirwa ML, Muula AS. Underutilization of cervical cancer prevention services in low and middle income countries: a review of contributing factors. Pan Afr Med J. 2015;21:231. doi: 10.11604/pamj.2015.21.231.6350 26523173 PMC4607967

[4] Clifford GM, Franceschi S, Keiser O, Schöni-Affolter F, Lise M, Dehler S, et al. Immunodeficiency and the risk of cervical intraepithelial neoplasia 2/3 and cervical cancer: A nested case-control study in the Swiss HIV cohort study. International journal of cancer. 2016 Apr 1;138(7):1732–40. doi: 10.1002/ijc.29913 26537763

[5] Massad LS, Xie X, Burk R, Keller MJ, Minkoff H, DʼSouza G, et al. Long-term cumulative detection of human papillomavirus among HIV seropositive women. Aids. 2014 Nov 13;28(17):2601–8. doi: 10.1097/QAD.0000000000000455 25188771 PMC4289460

[6] Arbyn M, Weiderpass E, Bruni L, de Sanjosé S, Saraiya M, Ferlay J, et al. Estimates of incidence and mortality of cervical cancer in 2018: a worldwide analysis. The Lancet Global health. 2020 Feb;8(2):e191–e203. doi: 10.1016/S2214-109X(19)30482-6 31812369 PMC7025157

[7] UNAIDS. UNAIDS Country factsheets Malawi 2019 HIV and AIDS Estimates 2019 [cited 2020 02 September]. Available from: https://aidsinfo.unaids.org/.

[8] Msyamboza KP, Phiri T, Sichali W, Kwenda W, Kachale F. Cervical cancer screening uptake and challenges in Malawi from 2011 to 2015: retrospective cohort study. BMC Public Health. 2016 Aug 17;16(1):806. doi: 10.1186/s12889-016-3530-y 27535359 PMC4989288

[9] Maseko FC, Chirwa ML, Muula AS. Health systems challenges in cervical cancer prevention program in Malawi. Global health action. 2015;8:26282. doi: 10.3402/gha.v8.26282 25623612 PMC4306748

[10] National Statistical Office (NSO) [Malawi]. 2018 Malawi Population and Housing Census Report-2018. 2019.

[11] Bank W. https://data.worldbank.org/indicator/SP.POP.2529.FE.5Y?locations=MW 2019 [cited 2020 30 Dec]. Available from: https://data.worldbank.org/indicator/SP.RUR.TOTL.ZS?locations=MW.

[12] Lipworth WL, Davey HM, Carter SM, Hooker C, Hu W. Beliefs and beyond: what can we learn from qualitative studies of lay people’s understandings of cancer risk? Health expectations: an international journal of public participation in health care and health policy. 2010 Jun;13(2):113–24. doi: 10.1111/j.1369-7625.2010.00601.x 20536535 PMC5060527

[13] Ebu NI, Mupepi SC, Siakwa MP, Sampselle CM. Knowledge, practice, and barriers toward cervical cancer screening in Elmina, Southern Ghana. International journal of women’s health. 2015;7:31–9. doi: 10.2147/IJWH.S71797 25565902 PMC4284003

[14] Moucheraud C, Kawale P, Kafwafwa S, Bastani R, Hoffman RM. "It is big because it’s ruining the lives of many people in Malawi": Women’s attitudes and beliefs about cervical cancer. Preventive medicine reports. 2020 Jun;18:101093. doi: 10.1016/j.pmedr.2020.101093 32322461 PMC7168763

[15] Nyambe A, Kampen JK, Baboo SK, Van Hal G. Knowledge, attitudes and practices of cervical cancer prevention among Zambian women and men. BMC Public Health. 2019 May 4;19(1):508. doi: 10.1186/s12889-019-6874-2 31054569 PMC6500583

[16] Fort VK, Makin MS, Siegler AJ, Ault K, Rochat R. Barriers to cervical cancer screening in Mulanje, Malawi: a qualitative study. Patient preference and adherence. 2011 Mar 14;5:125–31. doi: 10.2147/PPA.S17317 21448296 PMC3063659

[17] Ports KA, Reddy DM, Rameshbabu A. Cervical cancer prevention in Malawi: a qualitative study of women’s perspectives. Journal of health communication. 2015;20(1):97–104. doi: 10.1080/10810730.2014.908986 25116413

[18] The Government of Malawi MoH. National Cervical Cancer Control Strategy. United Nations Population funds, 2017.

[19] Chinula L, Topazian HM, Mapanje C, Varela A, Chapola J, Limarzi L, et al. Uptake and safety of community-based "screen-and-treat" with thermal ablation preventive therapy for cervical cancer prevention in rural Lilongwe, Malawi. International journal of cancer. 2021 Mar 9. doi: 10.1002/ijc.33549 33687746 PMC8141028

[20] Sudenga SL, Rositch AF, Otieno WA, Smith JS. Knowledge, attitudes, practices, and perceived risk of cervical cancer among Kenyan women: brief report. International journal of gynecological cancer: official journal of the International Gynecological Cancer Society. 2013 Jun;23(5):895–9. 23694983 10.1097/IGC.0b013e31828e425c PMC3662490

[21] Msyamboza KP, Manda G, Tembo B, Thambo C, Chitete L, Mindiera C, et al. Cancer survival in Malawi: a retrospective cohort study. Pan Afr Med J. 2014;19:234. doi: 10.11604/pamj.2014.19.234.4675 25838862 PMC4377240

[22] Touch S, Oh JK. Knowledge, attitudes, and practices toward cervical cancer prevention among women in Kampong Speu Province, Cambodia. BMC cancer. 2018 Mar 15;18(1):294. doi: 10.1186/s12885-018-4198-8 29544466 PMC5856224

[23] Getachew S, Getachew E, Gizaw M, Ayele W, Addissie A, Kantelhardt EJ. Cervical cancer screening knowledge and barriers among women in Addis Ababa, Ethiopia. PloS one. 2019;14(5):e0216522. doi: 10.1371/journal.pone.0216522 31075122 PMC6510425

[24] Getahun F, Mazengia F, Abuhay M, Birhanu Z. Comprehensive knowledge about cervical cancer is low among women in Northwest Ethiopia. BMC cancer. 2013 Jan 2;13:2. doi: 10.1186/1471-2407-13-2 23282173 PMC3559275

[25] Mitiku I, Tefera F. Knowledge about Cervical Cancer and Associated Factors among 15–49 Year Old Women in Dessie Town, Northeast Ethiopia. PloS one. 2016;11(9):e0163136. doi: 10.1371/journal.pone.0163136 27690311 PMC5045174

[26] Bukirwa A, Mutyoba JN, Mukasa BN, Karamagi Y, Odiit M, Kawuma E, et al. Motivations and barriers to cervical cancer screening among HIV infected women in HIV care: a qualitative study. BMC women’s health. 2015 Oct 12;15:82. doi: 10.1186/s12905-015-0243-9 26458898 PMC4603977

[27] Africa BN. Malawi cracks down on ‘vampire’ lynch mobs. BBC News Africa. 20 October 2017 [10 December 2020]. Available from: https://www.bbc.com/news/world-africa-41692944.

[28] Nyabadza F, Mukandavire Z. Modelling HIV/AIDS in the presence of an HIV testing and screening campaign. Journal of theoretical biology. 2011 Jul 7;280(1):167–79. doi: 10.1016/j.jtbi.2011.04.021 21536051

[29] Nigussie T, Admassu B, Nigussie A. Cervical cancer screening service utilization and associated factors among age-eligible women in Jimma town using health belief model, South West Ethiopia. BMC women’s health. 2019 Oct 28;19(1):127. doi: 10.1186/s12905-019-0826-y 31660938 PMC6819648

[30] Oketch SY, Kwena Z, Choi Y, Adewumi K, Moghadassi M, Bukusi EA, et al. Perspectives of women participating in a cervical cancer screening campaign with community-based HPV self-sampling in rural western Kenya: a qualitative study. BMC women’s health. 2019 Jun 13;19(1):75. doi: 10.1186/s12905-019-0778-2 31196175 PMC6567898

[31] Morema EN, Atieli HE, Onyango RO, Omondi JH, Ouma C. Determinants of cervical screening services uptake among 18–49 year old women seeking services at the Jaramogi Oginga Odinga Teaching and Referral Hospital, Kisumu, Kenya. BMC Health Serv Res. 2014 Aug 6;14:335. doi: 10.1186/1472-6963-14-335 25100298 PMC4271847

[32] Coleman JS, Cespedes MS, Cu-Uvin S, Kosgei RJ, Maloba M, Anderson J, et al. An Insight Into Cervical Cancer Screening and Treatment Capacity in Sub Saharan Africa. Journal of lower genital tract disease. 2016 Jan;20(1):31–7. doi: 10.1097/LGT.0000000000000165 26579842 PMC4691409

[33] Moon TD, Silva-Matos C, Cordoso A, Baptista AJ, Sidat M, Vermund SH. Implementation of cervical cancer screening using visual inspection with acetic acid in rural Mozambique: successes and challenges using HIV care and treatment programme investments in Zambézia Province. Journal of the International AIDS Society. 2012 Jun 18;15(2):17406. doi: 10.7448/IAS.15.2.17406 22713260 PMC3499800

[34] Gatumo M, Gacheri S, Sayed AR, Scheibe A. Women’s knowledge and attitudes related to cervical cancer and cervical cancer screening in Isiolo and Tharaka Nithi counties, Kenya: a cross-sectional study. BMC cancer. 2018 Jul 18;18(1):745. doi: 10.1186/s12885-018-4642-9 30021564 PMC6052645

