# Supplementary material for: Perceptions of cervical cancer and motivation for screening among women in Rural Lilongwe, Malawi: A qualitative study
Source: PLoS One. 2022 Feb 7;17(2):e0262590. doi: 10.1371/journal.pone.0262590 (PMC8820632; doi:10.1371/journal.pone.0262590)
Supplement: S1 File — (DOC) [file pone.0262590.s001.doc]

**12 week Follow-up Qualitative Interview Guide**

PID ____ ____ ____ Visit Date ____ ____/____ ____ /____ ____(dd/mm/yyyy)

Interviewer ID: ____ ____ ____ Start time: ___ ___: ____ ____AM/PM

________________________________________________________________________________________

***Interviewer Script:*** *Thank you for meeting with me today! I really appreciate your time and your input will be very helpful. I am working with a team of researchers from the University of North Carolina Project in Malawi, Your input is also important to help us understand how best we can conduct cervical cancer screening campaigns in Malawi.*

*There is no right or wrong answer. Everything you say will be confidential and only used to make this health program and health questionnaire better. I will audio record this interview to help me remember what was said, but your name or any identifiable information will not be connected to anything you say.*

________________________________________________________________________________________

*The VIA screening and Thermo-coagulation treatment experience:*

1. **Can you tell me your understanding of the cervical cancer screening and treatment you received ~12 weeks ago?** *(PROBES: What screening did you undergo? What was the purpose of the screening? What was your result? What other procedures did you undergo?)*
2. We would like to hear your thoughts about cervical cancer screening campaigns like this one where you got screened, **why did you choose to get screened or participate in this study?**
   1. **Was there anything you were worried about before the screening?** *(PROBES: what had you heard about the screening? what were your concerns? Any misconceptions about the screening? )*
3. **When you heard that the results of your VIA screening was abnormal, how did it make you feel?** *(PROBES: were you scared? Did you understand what it meant?)*
4. **What do you think was done well? What could have been done better?** *(PROBES: location, time, efficiency, comfort, explanation, education, partner involvement?*
   1. **What was the easiest part? What was the hardest part?** *(PROBES: Was there anything unexpected?)*

*Follow-up challenges:*

1. **It can be hard for people to come for follow-up, did you have any challenges coming for this follow-up visit? If so, what challenges?** *(PROBES: money, transportation, unable to get away from duties, did not feel it was necessary, partner/relatives did not want her to come)*
2. **What challenges do you think other women have to come for follow-up?**
3. **How best can we help women overcome those challenges?** *(PROBES: any suggestions on how we can make it easier for women to followip)*

*Partner and community support:*

1. **Did you discuss this screening with anyone else***? (Probe: like your partner, family or friends?*

**A. What did they say?** *(PROBES: were they encouraging? What questions did they have?)*

1. **Does your partner know that you had cervical cancer screening? If so, what does he think? *(****PROBES: did you need his approval? What support did you need from him? what does he think about cervical cancer screening? Was he interested in learning more?)*
   1. **If not, why not?**
   2. **Did you discuss the results with him?** **Why or why not?** *(PROBES: what did he think? Was he supportive?)*
2. **After thermo-coagulation treatment we advise against not having sex for 1 month to allow for healing, was this a challenge for you? Was your partner supportive of that?** *(PROBES: did he allow you to stay for 1 month without having sex?)*
3. **Do you think male partners should be more involved with cervical cancer screening for women? If so, how so?** *(PROBE: Should they come to screening with their wives?* **If not, why not?** *How can we include men more?)*
   1. *How best can we educate men about cervical cancer?*

*Knowledge and personal risk assessment*

1. **Is there anything new you have learned about cervical cancer or cervical cancer screening that you did not know before the study? *(****PROBES: Cause of cervical cancer? Prevention of cervical cancer? Screening options? Treatment options?)*
2. **Who do you think should be screened for cervical cancer?** *(PROBES: age, sexually active, pregnant, HIV-infected)*
   1. **How often should women be screened?** (***Probes:*** *once in lifetime, yearly, only with symptoms etc.)*

*Recommendations for future screening campgains*

1. **What do women in your community think about cervical cancer screening?** *(PROBES: limited knowledge, stigma, do not think they are at risk)*
   1. **Do you think that women in your comunity understand the importance of cervical cancer screening? Why or why not?**
2. **In your opinion, do you think that women are interested in receiving this screening and treatment service? What makes you think that way?**
   1. **Why do you think would someone not want to be screened?** *(PROBES: are they afraid to find out the result? Is there stigma against positive screen? Are they afraid of the treatment? Are they afraid to be diagnosed with cancer?)*
3. **What are some of the barriers that women might face in receiving this service? :** *(PROBE:* *about partner, friends, significant others)*
4. **In your opinion, how should cervical cancer screening be provided to ensure that more women can get screened?**
   1. **How can you encourage them for screening?**

*Self-collecting vaginal swabs*

***Interviewer Script:*** *Let’s now discuss about self-collected vaginal swab for cervical cancer screening. A new method has been developed for cervical cancer screening. It involves having a woman collect a swab from her vagina and submitting it at her convenience to a health facility for testing. However, unlike VIA, the woman would not get her result immediately and would have to return to health facility to get her result a few hours later or the next day.*

1. **What do you think about this idea? Would you be interested in undergoing screening this way?** *(PROBES: what are good things about it? What are bad things about it?)*
2. **How does the idea of self-collection compare to the VIA screening you went through?** *(PROBES: Which method do you prefer? Why or why not?)*
3. **What do you think other women in your community would think about the self-collected vaginal swab technique for screening?**
4. **Do you think more women would undergo screening with this methods? Why or why not?**
5. **What difficulties would women face in self-collection technique?** *(PROBES: what are your concerns about it? What would hinder collection?)*
   1. **What are some reasons, if any, why you think women would not want to self-collect? Why do you think women would prefer to go to a hospital for screening with medical providers?**

*Now let’s talk about your recommendations for the future of the National cervical cancer screening in Malawi:*

1. **In your opinion, should MOH consider including self-collected vaginal swab for cervical cancer testing to the cervical cancer screening programme?** Why or why not?
   1. **Would this make it easier for women to undergo screening? Why do you feel that way?**
2. **What groups of women could be most suitable for self-collected vaginal swab for cervical cancer testing?** What makes you think that way?

a. What groups of women would not be suitable? What makes you think that way?

Do you have any questions or comments?

Thank you for your participation in this discussion today. This information will help us to improve on the cancer screening campaigns in Malawi.

End time___ ___: ____ ____AM/PM
