# Supplementary material for: Perceptions of cervical cancer and motivation for screening among women in Rural Lilongwe, Malawi: A qualitative study
Source: PLoS One. 2022 Feb 7;17(2):e0262590. doi: 10.1371/journal.pone.0262590 (PMC8820632; doi:10.1371/journal.pone.0262590)
Supplement: S3 File — (ZIP) [file pone.0262590.s003.zip › VIA 240.docx]

**PARTICIPANT ID: VIA240**

**DATE OF INTERVIEW: 19 October 2017**

**INTERVIEWER ID: 466**

**TYPE OF INTERVIEW: 12 weeks follow up**

**KEY: I= Interviewer, R= Respondent**

**Interview summary:**

According to this woman, she was afraid at first to go for screening because of what was being said by the community, but she and her friends decided to go because she thought the services was very helpful. During the screening process, she learnt that she was found VIA positive, she was surprised because she was not expecting it.

Regarding the follow up challenges, she thinks distance and transport money is the main challenge, she suggested that the clinic staff should be provide a vehicle to pick women from various pickup point and increase stipend because it’s not enough. The participant recommended that male involvement is important, because knowing what is going on in their partners’ bodies, this can help then live happily.

On self-collection of vaginal swabs, she said a simple way of doing screening, and many women can benefit from it, and she is recommending that the government should adopt a self-sampling vaginal swab for HPV testing, but after being trained by physicians how best it can be done.

**Interview text:**

1. I: Thank you for meeting with me today! I really appreciate, your time and your input will be very helpful in this study. I am working with a team of researchers from the University of North Carolina Project here in Malawi, here where we are. They call it UNC. Your input is important to us to help us understand how best we can conduct cervical cancer screening campaigns here in Malawi. There is no right or wrong answer this is not an examination that they are going to mark, right?
2. *R: True.*
3. I: Yes, whatever you are going to say is going to be confidential that is why here we are the two of us, and we are not going to use especially what you are going to say is going to be used only to make this health program and health questionnaire better. I am going to record the interview in this thing so that it should help me with what was. said as I cannot remember whatever you have said even if am writing I cannot manage.
4. *R: You cannot manage indeed.*
5. I: Yes, but also your name or anything that you are going to say that can identify you it is not going to be connected with anything what you are going to say in this recording. So the first thing I want to find out about your cervical cancer screening, can you tell me your understanding on the cervical cancer screening and the treatment which you got?
6. *R: Yes the goodness what i saw…..?*
7. I: Be a bit loud so that it should be audible here.
8. *R: The goodness which I saw in helping cervical cancer screening! The way I saw it,* *the day you have gone for screening I was afraid, I thought it is going to be a bad thing with what the community says. So after seeing that we have been screened with the instructions which they gave us to use when telling us the date like when to report to the here and seeing that am seeing improvement! With what they told me, when screening me! Like the way my body is. Because the time they were screening me I was having other signs, since that time up to now I haven’t seen any bad on cervical cancer screening. Am feeling much better.*
9. I: Alright, you have explained that they were bad talk from the communities, can you tell me what was it like
10. *R*: *They talk of the community.* *They were saying things like “the world is dangerous these days,*
11. I: What were they saying that what is difficult with the world?
12. *R:* *The world is difficult like showing that they will be collect other things here so that we should be found with diseases*, *give us sickness* *at the village*
13. I: What were they saying that they were collecting from you?
14. *R: Blood so that we should be like we are diagnosed with diseases in our bodies but we just accepted saying that no, everybody takes care of her own life. So we saw that these things are helpful, these things have never happened, am seeing change may be if we are going to be found with diseases you will see it from us, yes*
15. I: Alright what procedures did they use in screening for cervical cancer?
16. *R:* *During screening they have their equipment we should say the owners, so they a metal one but we don’t see it down but we just feel that they are examining us, we know pretty well that here they the thing has gone inside it has touched the cervix*. *They said if you are found with abnormal VIA, so may do thermo-coagulation.*
17. I: Hm.
18. *R:* *We could see that others were being screened and going back not telling us anything to say that they have this, but we could just see how they were conducting themselves and saying that we should do thermos, that’s when you knew that its VIA positive.* *The way they know themselves*.
19. I: Can you explain well to me what happened when they were screening you, for you to know that you are having thermo-coagulation?
20. *R:* *I felt a little bit of pain, that’s when I knew that* *they are doing thermo-coagulation. I felt a little pain during screening.*
21. I: What did they say at the time they were screening you?
22. *R:* *They said that, woman we have found the abnormal VIA screening result on the cervix and we are going to do thermo-coagulation.*
23. I: Hm! alright thank you so much, do you know why they were screening?
24. *R:* *They wanted to know if I may have abnormal VIA or not.*
25. I: Right so you have said that they told you that you have been found with abnormal VIA screening result, are there procedures which were taking place that you can tell me?
26. *R:* *Concerning the screening?*
27. I: Yes!
28. *R:* *What I was feeling in my body*?
29. I: Especially during the time they were screening you, what was happening.
30. *R:* *I should not lie, I cannot manage to explain because I just saw that the doctors were touching this and that, so for me all I wanted was just to be treated. I just felt pain and then they told me that we have done the thermo-coagulation the cells have been* *thermo-coagulated.*
31. I: Hm alright, so I want to hear you views concerning the campaigns of cervical cancer screening like the one you have been screened with, why did you choose to be screened or may be taking part in the study?
32. *R:* *Because* t*he things came in a haste, right* a*nd also that health personnel are coming we didn’t know, we were at the church and we just saw the village headman saying can 2 women stand up and should go mop, because the vehicle has arrived. So that is when I said people have come? A lot of people come here, they said health personnel have come they are looking for you, that is when we had a conscious. Oh cancer is very dangerous so we should not be shy when cancer we know we have it, it is not curable. “What should we do women”? we were discussing saying lets go others refused saying cancer is dangerous, I cannot make the mistake of screening because cancer is very dangerous knowing how they are because we will be fear will find us there but we said we should be screened they should tell us whether we have it or not and we should get treatment that’s* *it.*
33. I: Hm alright did you have any worries before screening took place?
34. *R:* *I should not lie they are* *no worries I should say there* *was none*
35. I: What did you hear about screening before people came to conduct the screening?
36. *R:* *Before people came*!
37. I: Hm!
38. *R:* *Hearing what others are talking* *about*?
39. I: like may be people are coming for screening services? May be there is screening?
40. *R:* *No there is nothing that I heard we heard everything the same day.*
41. *I:* What about that people are screened in health care facilities, what did you hear?
42. *R:* *I heard it on the radio.*
43. I: What did you hear from the radio?
44. *R: I heard that the issue of cervical cancer screening is very important, that women should go to* *the health care facilities to be screened, we thought it is not important to do cervical cancer screening, we thought it is better to do VCT, and yet cancer is a very dangerous, So when I heard that health personnel have come, I decided to go for it*.
45. I: Did you have any worries after you heard that cervical cancer screening people are coming!
46. *R:* *I was* *afraid that if I get screened found VIA, that means my friends will be talking about me saying I have cancer. And that I will be discriminated against.*
47. I: Hm alright, so you explained, what people were saying, that you will be drawn blood, is there misconceptions about cervical cancer screening.
48. *R:* *No*
49. I: There is none?
50. *R:* *Yes!*
51. I: when you heard the results of VIA screening, that they were abnormal, how did you feel?
52. *R:* *After being screening?*
53. I: Yes after being told that you have abnormal VIA after screening, how did you feel in your heart?
54. *R:* *I felt fear that, I have been diagnosed, they say cancer is difficult, I am going to die. That was my worry*.
55. I: Okay!
56. *R:* *That was my worry which was there, I said maybe God has a purpose that was my worry but I just accepted that, what has happened has happened! but still more I should thank the health personnel for the treatment they gave us. I did not know that I could can be found positive after screening.*
57. *I:* Did you understand what this meant?
58. *R*: *Yes I understood!*
59. I: What did you understand?
60. *R:* *The instructions which they gave us after screening. They told us that we should not have sex for six weeks to allow proper healing. Then after six weeks should come for review.*
61. I: Ok, so when you were found that you have abnormal VIA screening result of the cervix, did you understand what this meant?
62. *R*: *So, my understanding was that the abnormal VIA screening result on the cervix is the cancer itself, it is the beginning of cancer.* *So in the community I was not hiding, I was able to share the results with other women, and encouraging them to do the same, because if it’s been detected early, you will be able to get treatment.*
63. I: Alright!
64. *R: We are the ones who had thermo-coagulation, have been thermo*-*coagulated*
65. I: The time when screening was been done, what was done better to you?
66. *R*: *On my body?*
67. I: Especially the during screening, and all the event taking place, you should tell me what you think went well?
68. *R*: *where I live?*
69. I: At the screening site, the time you were being screened, what *was* done well?
70. *R:* *screening to me, was done well. I did not see anything wrong!*
71. I: Okay, so according to you, cervical cancer screening went on well?
72. *R*: *Yes*
73. I: What about, what was not done well?
74. *R*: *nothing*
75. I: The place where screening was done, how was it like?
76. *R*: *The place was an early childhood development* (EDC) *school.* *They came with equipment used when doing the screening.*
77. I: Do you feel your privacy was maintained?
78. *R*: *Our privacy was maintained because they covered the place, and no one could peep* *through nor hear anything from outside.*
79. I*:* Okay, alright what about the time, when you were being screened?
80. *R: The time was in the morning around 8am.*
81. I: 8:00, how long did it take to be done with you?
82. *R: We were taking little bit of time for us to come out because it depended on how the person is.*
83. I: So how did this affect you?
84. *R:* *This affected me because they saw that I had an abnormal VIA and that is why I took time for thermo-coagulating.*
85. I: How did you feel seeing that you were taking a long time?
86. *R:* *There was nothing that I felt!*
87. I: Mostly in your thoughts, since you took a long time, how did you feel?
88. *R:* *No there was no worry, because I saw* *others before me, also taking a long time and I knew I would not come out early.*
89. I: How was the doctor’s conduct?
90. *R: The way I saw it, it was easy to understand. And they conduct was good, they encouraged us not to be shy.*
91. I: Hm, alright is there anything else they were saying?
92. *R*: *They said that “this is not our last visit coming here we will still be coming”, we provided locator information, they were documentation, “We want to be following you in your homes and see if there is improvement” that’s what they were saying.*
93. I: What was the easiest part when they were screening you?
94. R: *The part which was easy, was during a session with people who were found without problems, they were easy to handle because they were* *coming out fast.*
95. I: What was the hardest part?
96. *R*: *The hard part was a session with VIA positive women because they were taking long time to get out.*
97. I: Was there anything that you did not expect which happened at that time?
98. *R*: *We had no answers because we were taken by surprise, what we were not expecting to take place is* *what happened!*
99. I: Like what?
100. *R*: *Like, being found VIA positive.*
101. I: thank you so much, so I want to ask you concerning challenges about for follow up visit now, it may be difficult for people to come again to the health care facility or may be at mobile screening site did you have problems coming to the health facility for this follow up visit?
102. R: *Yes there were problems!*
103. I: What were the problems?
104. *R*: *Because the problem was this which* *we were found with.*
105. I: Especially problems because you have a problem you need to come to health care facility.
106. *R*: *Yes.*
107. I: But may be having a problem of you leaving home coming to the health facility,
108. *R: Problems are there for us to leave from home to get here, we need transport money.*
109. I: okay.
110. *R*: *So we may take transport from other people like borrowing so that we should come here, but the worry is that getting from here the money is not enough.*
111. I: How much money do they give you?
112. R: *K3, 500, so for us to leave our home coming here it is not enough, because transport is expensive now.* *Sometimes a person needs to buy something to eat, like a banana and you will see that this is not helpful, they should increase the stipend.*
113. I: Alright what else can make you fail to come here to health care facility for a follow up visit?
114. *R*: T*he issue of transport like I have said, or may be something has happened in the village.*
115. I: Somethings like what?
116. *R*: *like funeral.* *Or maybe, feeling sick that I cannot manage to walk, this can also make me to miss a follow-up visit.*
117. I: Is there anything else?
118. *R*: *No*,
119. I: Let us talk about other women, which are the challenges other women may have which may make them fail to come for the follow up visit.
120. *R*: *Fear.*
121. I: Fearing for what especially?
122. *R*: *Fear they say they cannot go for cancer screening as I already said that in the communities there is a lot of misconceptions, they think if you found VIA positive that means it’s a death sentence.* *This is why other women are failing to come for cancer screening but when we go home and explain our experiences, they have start admiring saying may be had it been that I went for screening I would have been in this group.*
123. I: Okay, what challenges which may make other women not come even if they did the screening well, the doctor told them to come to the health care facility after six weeks, what can make them fail to come.
124. *R*: *Women from the community* *those who did screening and given a date like* *the* *way we come here*?
125. I: Yes!
126. *R*: *This may be due to worry or lack of means* *of transport* *and misunderstanding*.
127. I: Misunderstanding about what?
128. *R*: *Not understanding what of the doctors said or not taking it seriously.*
129. I: Is there another problem?
130. *R*: *No.*
131. I: How best can we help women to overcome those challenges?
132. *R*: *There is need for us who come here to talk to them and encourage them.*
133. I: What else can happen?
134. R: *On the same issue?*
135. I: May be on the issue of the study or what can happen that these people can be treated so that their challenges could be addressed.
136. *R: The campaign should not stop. So that those who do not take is seriously should understand.*
137. I: Alright is there anything else that we can do which can help that women should be coming or may be dealing with the challenges women encounter.
138. *R: Telling these women our experiences and our stories can encourage them to go for screening.*
139. I: Alright then I want to know about support of your partner and also from the community where you come from, did you discuss about this screening with anybody else?
140. R: *Like in my house or in my community* *in the village?*
141. I: In way you can tell me.
142. *R*: *I encourage others a lot or I discuss a lot with my friends even in my house with my husband and I also shared my results, and that if I didn’t go screening I would have not lived for long, sometimes you may be sick without feeling any pain. Like me I was not sure if I was sick because I was just staying not feeling anything in the body. So I plead with other women to try* *screening.*
143. I*:* So here it shows that you told your partner you told other women may be we should start with your husband when you explained to him what did he say*?*
144. *R*: *When I explained to my husband he said that you have done a wise thing because we would have been just staying here or may be when you started falling sick we would just be saying its malaria*, *not knowing your body is damaged but we should pray that the health personnel should treat you.*
145. I: Did he had any questions?
146. *R*: *He did not have, the way I was explaining saying you should understand me better, I have been found with an abnormal VIA screening result and they did thermo-coagulation on me, he said have they thermo-coagulated you? Are you going to be saved? He had fear. I told him how I was feeling in my body because I was not feeling anything. He asked again that they thermo- coagulated you inside and I said yes they need to do that inside, we should abstain for six weeks, he agreed that it is true we should abstain.*
147. I: So when you told your friends what did they say?
148. *R*: *Friends did not show interest at first, then I said stay but what you should know is that I did that, they said go and I told them I will go again on the sixth and the health personnel will be visiting us in our homes meaning they don’t have bad intentions. I have done all I could to encourage them.*
149. I: Alright, you said that you explained to husband that you had an abnormal VIA screening result and you ha a discussion but what does he think about your cervical cancer screening?
150. *R*: *My husband had fear that am I not going to live long with him because cancer is very difficult, from there he asks me whether they have diagnosed me again or if the cancer is still there, he asks if the cancer is still there and I tell him am healed because last time I was told that there are no abnormal cells but may be you can have another screening test after 3 years.*
151. I: If you want to do anything or coming here do you seek the permission of your husband*?*
152. *R*: *That I should come* *here?*
153. I: So that he should give you permission for you to come here?
154. *R: No, I just tell him when am coming back here. He even drops me off to the bus depot He tells me that these are helpful things whatever they tell you there, you should be telling me here.*
155. I: Apart from dropping you off the depot, is there any other support you require from him?
156. *R: Money, if he has some he gives me, if not, he borrows and give me.*
157. I: on top of thermo-coagulation of the abnormal cells we advise you to abstain for six weeks to allow healing process to take place as you have explained, was this a challenge to you?
158. *R: No it was not.*
159. I: *Why*?
160. *R: Because of the instructions we were given, we following those instructions, may be if we missed the instruction we would have been in a bigger problem than the current one.*
161. I: Does your husband support or agree with this?
162. *R: He supports.*
163. I: How?
164. *R: he tells me to continue coming, like I have explained, he says “unless the health personnel tells you that you are completely healed”*
165. I: What about abstaining for six weeks?
166. *R: A man is a problem because my husband has another wife so I tell him to go to the other wife, it is better for him to go to another wife because that will help me heal.*
167. I: Alright meaning that he didn’t allow you to go a month without sexual intercourse?
168. *R: Yes because I told him that am sick and he understood that it really is difficult.*
169. I: Do you think that men should be involved in cervical cancer screening for women, should they be involved?
170. *R: They have to be involved because if they do are not involved then these programs will not be successful.*
171. I: How can they be involved?
172. *R: when we tell them stuff that we have been told at the hospital they should be able to understand what this person is saying is the truth because if we miss the instructions then everything will be messed up.*
173. I: How do you see it, if they can be coming along when their wives are coming for cervical cancer screening?
174. *R: My husband wishes to be coming with me saying so he know the place we go to. So that he should hear instructions himself? That’s why my husband really wants to see this place.*
175. I: What can be your advice concerning male involvement in cervical cancer screening?
176. *R: I also have hope that one day it will be possible that they will come with their wives and see for themselves.*
177. I: What about to other men what can you say?
178. *R: I do not know about other men,*
179. I: Is it important, because we want this to help cervical cancer screening services to go on well, so we want you to tell us your thoughts are, should men be coming with their wives when coming for cervical cancer screening specially in the context cancer?
180. *R: Yes I want the men to be involved.*
181. I: Why do you think they should be coming?
182. *R: So that they participate in the healing process of their wives.*
183. I: How do you think we can encourage the men to be involved?
184. *R: We will try to encourage them.*
185. I: Especially us from the hospital how can we encourage them to be involved? You can also take part in encouraging them but then we are talking about men in general not just your husband these men how can we encourage them to come here to the health care facility? Or that they should be involved?
186. *R: What is needed from you is that when you doing follow ups, you should also be explaining to our husbands about our results, this could also be encouraging to the men.*
187. I: So I was asking how we can help men especially how we can encourage them concerning cervical cancer?
188. *R: That is what I was saying there is need for you hospital people to get the men involved when you are discussing results.*
189. I: I also wanted to know if there is something new you have learnt about cervical cancer or cervical cancer screening that you did not know before the study started?
190. *R: Yes, since the campaign is new to us, and we have been educated about cervical cancer,*
191. I: The time you were being examined what did they say causes cervical cancer?
192. *R: They said cancer is brought by males that is what we were taught. cancer is brought by males because men go around and they are not easily satisfied that’s where they take that disease and pass it on to the women that is how cancer is spread.*
193. I: Okay how can cancer be prevented, what were you told?
194. *R: I was told that cancer can be prevented with these cervical cancer screening campaigns that were established in the communities*
195. I: What about the choices of getting medication, to choose the type of medication?
196. *R: So that we should be protected? Choice of medication, you doctors are the ones who need to tell us because you are the ones who know what medication corresponds with that disease.*
197. I: Who do you think is supposed to go for cervical cancer screening?
198. *R: Like at my place.*
199. I: Like everyone in general, who do you think is supposed to go for screening?
200. *R: A Woman.*
201. I: What kind of women?
202. *R: Women, because I was advised that from the age of 25 needs to go through cervical cancer screening, every woman.*
203. I: Why from the age of 25?
204. *R: They said that is the time they started sleeping with men, some at the age of twenty something means they already know a man it takes time to sleep with a man.*
205. I: Are there other people that can go through cervical cancer screening?
206. *R: Like at my place, yes a lot.*
207. I: Like pregnant women, are they supposed to go through cervical cancer screening?
208. *R: No, pregnant women said no they are not supposed to do cervical cancer screening.*
209. I: Why?
210. *R: Because they are expecting, they say the screening might affect the baby and they will say you doctors are bad people.*
211. I: What about those with HIV?
212. *R: If it is about cervical cancer screening it is possible because AIDS is something else and cancer is another disease, so maybe there is enough protection the other side and the cancer part they can also be given protection or treatment.*
213. I: So how do you think women should frequently do cervical cancer screening, in your opinion?
214. *R: Women need to go through cervical cancer screening according to how the doctors see it.*
215. I: I want to know your opinion not what the doctors are doing, but if you think how the women should be screened so that the work goes on well.
216. *R: If the women can see that they have been cervical cancer screened like we did within a short period of time they should go to the hospital again.*
217. I: The short period should be for how long?
218. *R: Maybe if it can be six months or five, so the person herself should feel it that it has been long since she last got cervical cancer screened and get screened again.*
219. I: Why do you think that a woman should go again after six months?
220. *R: Because women as I hear she has too many rooms to get infections so maybe if they can be reluctant to wait for few more years the cancer cells will multiply so when it gets to three years the disease would have settled in the body so it is needed for a person to frequently get cervical cancer screening to see how this is going to help her.*
221. I: Women from your community now I want to know your advice in view of future cervical cancer screening …. How do you think women from your community think about cervical cancer screening?
222. *R: Women from my community at first they did not think about it but now that we have done the cervical cancer screening they are thinking about it that they are delaying it is better they go do cervical cancer screening that they started asking us that they want to be coming along to do the cervical cancer screening so that they should get their bodies checked out, so I see these things are helpful because the women as and they say maybe they might be allowed when doctor went the day before yesterday and told us to be going in pairs because there is so much work so the women continued asking because the day you came you gave us form so can we not take the women along but it can be possible if you can find your own transport to and from because some of the women have already gone so I see as if they are hating you because of what they are doing that side because they already gave in their names and the women they really want to go and get a cervical cancer screening*
223. *.*I: Do you think these women are understanding the advantages of cervical cancer screening?
224. *R: Yes they understand very well and how they see their bodies now.*
225. I: How do they see it?
226. *R: That in their bodies they drink water now and they have loin pains again and looking at this so that’s when they ask that is it not possible for them to go the main hospital and get cervical cancer screen , maybe if the health care facility can verify the pelvis has disease.*
227. I: Thank you very much, what are your thoughts, do you think women are interested to do cervical cancer screening and get medication treatment?
228. *R: Yes they are interested.*
229. I: What is making you think like that?
230. *R: We want to be free cancer, and get treated.*
231. I: What do think is the cause of people not wanting to do cervical cancer screening?
232. *R: Reluctance by women in the community that is why can it not be possible for a reluctant woman to do cervical cancer screening.*
233. I: Because they are reluctant?
234. *R: Yes because they are reluctant.*
235. I: Other reasons?
236. *R: Other reasons might be because of misconception in the community.*
237. I: Is there anything else.
238. *R: Nothing else.*
239. I: We were talking about barriers that women can meet when getting treatment what is it that can be a barrier? , so that they do cervical cancer screening.
240. *R: Sometimes some males can discourage their women to go for screening because of jealousy, because there is undressing involved.*
241. I: How can a community be a barrier to cervical cancer screening?
242. *R: Misconception in the community can be a barrier, because they say, you are undressed by the hospital people, and women tend to be shy.*
243. I: Is there anything else that can be a barrier to women when they want to get treatment for cervical cancer screening and medication for cervical cancer?
244. *R: The other barriers can be they are sick but the heart wants but the disease will not let them go for cervical cancer screening.*
245. I: Is there anything else?
246. *R: I think nothing.*
247. I: In your opinion how cervical cancer screening services should be provided to ensure that a lot of women get screened?
248. *R: they campaign should be intensified and reach more women all over, so that more women can benefit from the service.*
249. I: So how the service should be provided? How exactly should they provide it?
250. *R: The program should continue, they should continue going to the communities to do screening and treatment.*
251. I: How can you encourage the women so that they go for cervical cancer screening?
252. *R: I would use my story and experience as a tool to encourage my fellow women to go for cervical cancer screening, because I am a living example.*
253. I: So now I want to ask you about self-sampling vaginal swab for HPV testing is a new way of cervical cancer screening it involves women taking vaginal fluids and cotton from a vagina and handing it to the clinicians or hospitals, this method the results are not instant, it will be delaying for some hours, or the following day so what do you think about these things?
254. *R: I didn’t know about other testing methods.*
255. I: About this testing using cotton what do you think about it?
256. *R: This method can work with those with difficult husbands, so maybe many women can follow this method, especially those who are shy also.*
257. I: Can you be interested being screened in this way?
258. *R: Yes,*
259. I: This way is still being studied, it is not taking place yet but we want your views to be used by researchers study when they get multiple views then they will see if this method is going to work, it is a method that was already implemented but they want to see if women have accepted it, so you have already mentioned those with difficult husbands what are some of the things that can be advantages of this method?
260. *R: Concerning this cotton method the person is still supposed to go the hospital right?*
261. I: She will be taking her own vaginal fluids, we will ask each other as we go on where they will be taking these vaginal fluids but they will be taking them alone and hand them over at the doctors going to the hospital when they are free.
262. *R: So this is a very helpful method.*
263. I: How?
264. *R: It is helping because if you are doubting yourself, u can just comfortably collect the sample yourself.*
265. I: What are the disadvantages of this method?
266. *R: I think there is no disadvantage on the cotton method, it is quite simple.*
267. I: Do you think it is supposed to be taken from home? The taking of the vaginal fluids should it be from home?
268. *R: Even right there at the hospital, it can be possible.*
269. I: Do you see it as an efficient?
270. *R: It is efficient, and using cotton will not be painful, rather than being screened by a physician.*
271. I: This self- sampling of vaginal fluids, so do you think it is efficient?
272. *R: Yes,*
273. I: Okay, so how can you compare being self-sampling and cervical cancer screening as you did, how can you compare the two?
274. *R: These two methods are very hard to compare because taking the sample alone is not like the way it is done by physicians at the hospital, we are not professions, compared to how the professionals can do it.*
275. I: Which method are you happy with?
276. *R: The method I am happy with is the same one I did, because results were instant and same as treatment.*
277. I: Okay, so how do you think some women from your community can think of self- sampling of vaginal fluids?
278. *R: They might like this method and many will be screened.*
279. I: Why?
280. *R: Like I said that many women are reluctant because they don’t want to undress, with this method a lot of people will be willing.*
281. I: What are the problems the women can face when taking their own vaginal fluids?
282. *R: When it comes to taking their own vaginal fluids maybe they can meet problems, maybe the sample will inadequate for testing.*
283. I: Why will they not find them?
284. *R: The sample can be different if taken by nonprofessional, and by the time it gets to the lab it may maybe dry or too soft, maybe it won’t be as the lab require.*
285. I: What are your worries on taking your own vaginal fluids?
286. *R: My worry is I cannot manage maybe because you doctors will train us on how best we do self-sampling then it will be possible*
287. I: What are the reasons, if there are any, that women would not want to take their own vaginal fluids.
288. *R: The reasons, are what we are saying, that you doctors are professional unless we are trained, maybe that can work.*
289. I: Okay, so now let us talk about your views of the future of cervical cancer screening in our country, in your opinion the ministry of health of Malawi should adopt this new method?
290. *R: I will agree that they should include that method so that many women should benefit.*
291. I: Which remaining people?
292. *R: women that haven’t gone for screening yet.*
293. I: Why will they be saying it is good?
294. *R: Because they will be taking their own vaginal fluids.*
295. I: Okay, do you see this making cervical cancer screening simple for women?
296. *R: Yes, it is not something hard it is actually a good thing.*
297. I: Why are you thinking like that?
298. *R: Because I can now differentiate now, I feel much better.*
299. I: So which groups of women are supposed to take their own vaginal fluids with cotton to the hospital for cervical cancer screening?
300. *R: I did not understand.*
301. I: Which groups of women are supposed to? Because at first place you said some women hate undressing so I want you to tell me another groups of people except for those who do not want to undress that might need to take their own vaginal fluids with cotton for self-testing?
302. *R: The group.*
303. I: What kind of women?
304. *R: They can be middle aged women or older.*
305. I: What groups of women that are not supposed to?
306. *R: For cervical cancer screening?*
307. I: Yes, that they should not self-test?
308. *R: as I said there are many women who are reluctant to go through cervical cancer testing with the method I went through, they would rather adopt a self-sampling method.*
309. I: Do you have anything else to add now that we have reached end of our discussion maybe you have questions.
310. *R: No, I think…*
311. I: Or something you can add?
312. *R: There is nothing really.*
313. I: Thank you so much for your time, we have held you for long but you have given us good facts which will improve our work, I am very grateful, this is end of our discussion.
314. *R: I am also grateful, thanks.*

**End of interview**
