# Supplementary material for: Perceptions of cervical cancer and motivation for screening among women in Rural Lilongwe, Malawi: A qualitative study
Source: PLoS One. 2022 Feb 7;17(2):e0262590. doi: 10.1371/journal.pone.0262590 (PMC8820632; doi:10.1371/journal.pone.0262590)
Supplement: S3 File — (ZIP) [file pone.0262590.s003.zip › VIA 253 Missed.docx]

**PARTICIPANT ID: VIA253**

**DATE OF INTERVIEW: 2 Nov 2017**

**INTERVIEWER ID: 466**

**TYPE OF INTERVIEW: missed 6 and 12 follow up visits**

**TRANSCRIBER ID: 901**

**KEY: I= Interviewer, R= Respondent**

Interview text:

1. I: Thank you very much for meeting me today. I really appreciate for your time, your responses will be very helpful and also very beneficial. I am working with researchers from University of North Carolina here in Malawi, yes. So sorry that you failed to come the time you were told to come here. So we want to hear from you. So we want to hear your experiences, relating to ahh screening of cancer of the cervix and also the assistance you have received. But we also want you hear the challenges that you had before the screening, right? Yeah. And especially the challenges you faced so that you fail to come to the follow up visit. Everything you say is very important to help us understand

[ door opening]

1. I: Yes, it’s very important for us to understand how best we can conduct cervical cancer screening campaigns in Malawi. So there are no right or wrong answers. Everything that you speak is very important to this research. Everything that you speak will be confidential and will only be used in improving health care procedures and also the questionnaire. Yes, and I will also record all the research, particularly these questions to help me to write everything you have said in the way you have responded. Yes, because you will be speaking, you will speak many things so I cannot manage to write everything, right? But your name and any other identifiers will not be linked to the recording done here. What we will record in here is your voice only.
2. *R: mmmh*
3. I: Yes, so my first question is can you tell me your understanding of screening of cancer of the cervix and the treatment you received.
4. *R: My understanding is that when they told me that I have cancer cells that cause cancer of the cervix. They said that they have a way of applying some medication that burn the cells. So I made sure to access that treatment*
5. I: mmmh
6. *R: Yes, so after I received that treatment I came here and they told me they would help me another time*
7. I: You came…?
8. *R: I came ahh what month was that? In (Month), oh maybe in (Month)? I came, on 7th of (month). I came so they told me to come back on the 19th of (Month). So on the 19th of (Month), a car would come to go there, they said we should come in pairs if two*
9. *I: mmmh*
10. *R: So with the communication for me to come on 9th, but because of transportation,*
11. I: On 9th?
12. *R: 9th (month)*
13. I: ok
14. *R: Because of transportation, I failed to come, I failed to come here. Then I received this receipt saying that on the 23rd I should be found here. I did my best to find transportation so that I could be here. Sure*
15. I: Ok, can you explain clearly on the transportation challenge that you faced, can you explain so that we clearly understand what happened so that you failed to come?
16. *R: Money was a challenge to buy fuel to get to (name of location), because where we stay is far to reach (Name of location), yes, that’s why we failed to come here. Even the first time that we came here, when we were returning we walked a long distance on foot. So I felt very weak in my body. Sure, sure*
17. I: Ok, part from transportation challenges, what *else was there that posed a challenge for you to come here?*
18. *R: No, there was nothing else, except transportation*
19. I: Ok
20. *R: Sure*
21. I: What do you think are the challenges that other women can cause other women not to come for their follow up visits, apart from the challenge that you faced? What other challenges can be there for women?
22. *R: Others challenges, others can just relax and just be lazy to come here. Others can be sick*
23. I: Mmmh, what else can be the cause?
24. *R: [ laughing shyly]*
25. I: Challenges are many
26. *R: Yes, there are many challenges*
27. I: What else can it be?
28. *R: I said maybe there can be a funeral, maybe sickness or funeral*
29. I: Yes, you didn’t mention the funeral,
30. *R: ohhh*
31. I: So, a funeral,
32. *R: A funeral*
33. I: mmmh, what else?
34. *R: It could be that they received the message late.* *So if they got it late they cannot make it here. Sure*
35. I: Mmmh so what do you think can be done to end these challenges?
36. *R: What can be done to end these challenges. For a person who has been found with cervical cancer?*
37. I: Yes, these challenges of women failing to come here at the clinic
38. *R: ohh. What you can do is maybe if you send transport from here to go and pick the women Yes*
39. I: Mmmh another way?
40. *R: Another way, you should telephone*
41. I: Telephone?
42. *R: To say so and so, you should be found here*
43. I: Mmmh
44. *R: Yes*
45. I: Another way?
46. *R: [ laughing shyly]*
47. I: What else can be done to help women not to miss so that they should not have challenges when coming here at the clinic?
48. *R: Another way, ahh this, I noted that the transport is insufficient, it needs to be increased, our homes are far. Yes, because it’s*
49. I: If it was increased by how much?
50. R*: Even if you increased by a thousand, like I said that the first trip, I felt it, I would have said let’s go together. I walked a long distance on foot. They kept saying let’s go, its near. We were just walking based on what we thoughts, each time I asked if we had arrived, they kept saying ‘no, let’s keep going,’ ‘no let’s keep going’. I suffered. Indeed, when I did that I said mmmh ‘Mom, I have suffered, ‘if it was that they had picked us and dropped us back, maybe we would have travelled well.*
51. I: Mmmh, ok, thank you. Since I had already started the question I thought that I should also ask in advance about the other people on the challenges other women face. So you have explained that you failed to come for the other visit because of transport challenges.

What other challenges did you encounter at the time you were screening for cervical cancer? You explained that they found that you have

1. *R: Yes, cancer cells*
2. I: And you saw that its good to be screened?
3. *R: It’s good that when women here that people screening for cervical cancer have come, they should go, because their lives will go on. Yes, I said that. Sure*
4. I: How was this testing done?
5. *R: This testing? We would lie down, face up. Then they would put metal thing. Yes, so when they put those metal things and saw the cervix. They said they would be putting medication. So that they could see the cells that cause the cervical cancer. Yes, then they would cut a sample of the body tissue from the cervix. Sure*
6. : I: Ok. So how were your results?
7. *R: They said no I am fine, where they sprayed the medication, the cells were burnt. So today they have taken other body tissues from all the four parts. So that they screen again*
8. I: What do you mean by all 4 sides?
9. *R: This side of the cervix, this side, this side and this side [???]*
10. I: Ok Thank you. Maybe to just explain, when you arrived what happened? The things that happened when you came?
11. R*: Here?*
12. I: Where you went for the screening. What was the process like up to the stage when they collected the body tissue?
13. *R: What happened was that, since they said “we found you with cancer of the cervix”, “So we need to get some body tissue to take for testing. If the cancer has spread far or not”. They did that. That’s what happened that time*
14. I: Mmmh, so they just started testing you? Or there were other things?
15. *R: They began with counseling. Yes, they started with counseling, telling us that cancer of the cervix comes because of the men. If they ‘meet’ with the woman who has the cancer cells, then they ‘meet’ (meaning having sex) you, that way they infect you. Yes, sure*
16. I: Thank you, is there anything else that happened?
17. *R: No there wasn’t*
18. I: Ok. So I want to know your thoughts of the awareness on the screening of cancer of the cervix, like it was done for you. The way you were screened. Why did you consent to be screened?
19. *R: So that I know my status. My heart told me to go and find out right there. Sure*
20. I: Why did you want to be screened?
21. *R: I wanted to know how my body was.*
22. I: Maybe there was something that was making you anxious, before the screening was done?
23. *R: Before the screening was done, I was anxious because my back hurts a lot, So I wanted to know what was causing this, maybe it was some sickness, I wanted to know at the health outreach, the clinic had come close so I needed to go for screening so I could know what my body was like. That’s why I left home and went there. Sure*
24. I: Ok. What did you hear about screening?
25. *R: [sighs]*
26. I: Because before something happens, there are things that you hear, right? There is something happening, this and that is happening etc
27. *R: Oh yes*
28. *:* I want to hear things like that. What did you hear that relates to screening?
29. *: R What I heard relating to screening, people were saying there are people who do screening coming. Who do cervical screening. Others, when they find that cancer has spread in the cervix they are able to remove the uterus. Yes, if it is just inside and has not spread elsewhere they are able to remove the uterus, but if it the cancer has spread to other parts, they just say go and wait for your time.*
30. I: mmmh
31. *R: Sure so I said let me go and hear how my body is. Sure*
32. I: Was there anything else they said apart from that?
33. *R: No, nothing*
34. I: So what were your concerns when you heard that?
35. *R: I was not anxious, I just wanted to make sure that I go and meet the doctors and know how my body is*
36. I: What myths/ misconceptions did you hear regarding screening?
37. *R: Nothing*
38. I: Nothing? When I say myths or misconceptions do you understand what I mean?
39. *R: no, [inaudible]*
40. I: Myths, things that people just say
41. *R: What people were saying, yes*
42. I: That may not be true
43. *R: No there wasn’t*
44. I: There wasn’t?
45. *R: No there wasn’t*
46. I: Ok, so when you heard that the results of your screening were not good, how did you feel? How did you feel when you heard that you have cancer cells that can cause cancer of the cervix?
47. *R: I felt, I realized that everything has its time, so it was time that I should encounter that, sure.*
48. I: In your heart how did you feel when you were told about your results?
49. *R: There was nothing that happened, I just said it was time*
50. I: Mmmh ok. Were you not afraid?
51. *R: No, no way, I wasn’t afraid*
52. I: So from your understanding what did you think it meant when they said you were found with cancer cells in your cervix?
53. *R: What I thought was if the cancer has started only in the cervix then they would remove the uterus but if it had spread to other parts of the body, that would be it, it means it is my time*
54. I: Mmmh, so thinking of those things how did you feel? Just like any person?
55. *R: [All laughing] Every person fears, yes, a person has fear. Sure*
56. I: So you felt fearful?
57. *R: Yes, I was afraid. Yes, sure*
58. I: How long did you remain fearful?
59. *R: That fear lasted just for a day, the next day I was ok*
60. I: How could another person tell that you were fearful? What was happening to you?
61. *R: I was having a headache. I had a horrible headache. Yes, up to the extent that I went to the Doctor for checkup, he checked me, I told him that I have a horrible headache. He just said ahh, “its fear” [laughing] “Its fear of what I told you yesterday”. So I just said ok. Then I noted the following day the headache stopped. I became ok. Sure*
62. I: So for it to stop what happened*?*
63. *R: Nothing. I just noted that it had stopped on its own. Sure*
64. I: When the doctor told you that its fear that was causing that, what did you do?
65. *R: I said give me Panadol to take. And at that time there were no drugs. Sure, they just said we will give you another day, sure*
66. I: Mmmh. Ok, what do you think went well at the time you were being screening for cervical cancer*?*
67. *R: [ inaudible]*
68. I: [Laughter]. What went well?
69. *R: What went well was that they used the metal equipment and tested the cervix, and saw the cells. So it went well because I never felt any pain. Sure*
70. I: mmmh. Ok, what do think could have gone well/better? When you were being screened? Something that you felt would have been better if it had been done a certain way.
71. *R: mmmh I have failed to answer*
72. I: You have failed to answer?
73. *R: mmmh*
74. I: Ok. What was the easiest thing on the things that were done?
75. *R: Nothing*
76. I: Or the easiest
77. *R: no, there was nothing*
78. I: Can you explain?
79. *R: There was nothing, everything was difficult*
80. I: Ahh like what was difficult?
81. *R: For them to open the cervix. To do the work they were doing. To do the checkup. That I felt was difficult*
82. I: What did you see to be difficult, what makes you say that it was difficult?
83. *R: I could feel/hear what was being done, yes*
84. I: What was happening?
85. *R: The equipment for testing, it was making clanging noises, they would try this then that or another, I saw that it was difficult*
86. I: It was difficult. Ok. Was there something that happened which you did not expect at the time?
87. *R: Yes, there was*
88. I: Like what?
89. *R: When I was leaving home I didn’t expect that they would find me with it. Cancer, ahh the cells, that they wouldn’t find me with the cells on the cervix. Sure and I just realized they had told me that they had found me with the cancer cells*
90. I: Ok. Thank you. So did you speak to anyone about the screening?
91. *R: No. I didn’t speak to anyone. Sure, because the said that it was confidential. So as Doctors told me to, I just kept quiet*
92. I: Mmmh, so you didn’t tell anyone?
93. *R: No, no one*
94. I: What about your husband?
95. *R: Only to him I disclosed, but to others I only said they had found me with a cyst in the uterus. So I need to be going to meet the Doctor. Now even if they ask me, I respond the same way. Because they told me that this is confidential. Sure*
96. I: So what did your husband say when you told him?
97. *R: When I told my husband he just said, “oh ok you need to be getting treatment at the hospital. Sure*
98. I: mmmh, were there any questions that your husband was asking?
99. *R: No, none*
100. I: Mmmh. Ok, so what does your husband think about cancer of the cervix?
101. *R: My husband thinks cancer of the cervix comes because us women we at times take traditional medicines and out into the cervix open, because I explained to him that this sometimes comes when you had a sexual encounter with someone who had the cancer cells (HPV) who then slept with someone without cancer therefore transmitting it. So he said, that is a lie, but the medicine that you women use to put in the cervix, when you insert that medicine (traditional), those medicines are the ones that cause cancer. So I just said that is not true*
102. I: mmmh on the medicine, what did the health care workers tell you about the issue of inserting medicines (into the cervix)?
103. *R: They didn’t say anything*
104. I: Ok. Thank you. So when you want to access medical services, what assistance do you require from them?
105. *R: From my husband?*
106. I: Yes, your husband?
107. *R: The assistance I need is just transportation to get here to the clinic.*
108. I: Mmmh, when you want to make a decision to go to the clinic do you need to seek his permission to go or not?
109. *R: No, he does not refuse, I shouldn’t lie. To say that I shouldn’t go? The challenge is often the money to buy fuel to put in the motor bike to travel to the clinic. As for him he does not refuse, he agrees for me to go. Sure*
110. I: Does he indicate that he has interest to learn more about cancer of the cervix?
111. *R: Yes*
112. I: Mmmh, why do you say that?
113. *R: Because when it’s time to, he encourages me to go to the clinic. Sure*
114. I: Mmmh, so after you told him about your screening, after telling him about your results, just to add on, what was your discussion like?
115. *R: There was nothing that we discussed, he just said it’s a lie that it’s the men who carry the cancer virus and infect the women. He just disputed that*
116. I: mmmh, ok, so after burning the cancer cells, we tell you not to have sexual relations with your husband for up to?
117. *R: For 6 weeks*
118. I: Yes, close to a month, right?
119. *R: Yes*
120. I: So that it heals?
121. *R: Mmmh*
122. I: How difficult was this for you?
123. *R: It wasn’t difficult. Because we are older. We really understood what the doctors said, sure*
124. I: Mmmh, what role did your husband take after you explained to him that you had been told not to have sexual relations for up to a month
125. *R: He just agreed. That we should do just as the doctor had said. Sure*
126. I: Mmmh ok, he was being difficult?
127. *R: No he wasn’t being difficult. Sure*
128. I: Ok. So do you think that the men should be allowed to be there when the women are being screened for cancer of the cervix?
129. *R: No, it just needs women*
130. I: Can you explain?
131. *R: It needs women because the men, since the women are shy with the men, men are men*
132. I: Let’s say for example, you, you go in with your husband, then another woman with her husband, so that they are involved when their wives are being screened for cervical cancer?
133. *R: oooh*
134. I: How do you think they should be more involved*?*
135. *R: The men can play a role, am saying they can take a role because, the woman should know how she is in her body. That’s how a man can play a role. They should agree when health personnel come to the community, to allow their women to go. Sure*
136. I: Mmmh, ok. Do you think they should be coming to the clinic with their husbands?
137. *R: Yes, they…*
138. I: When their wives are coming to be screened, should the husbands come along?
139. *R: Yes, so that they wait for you and you should encourage each other on the way going back. Yes, like I have said on my part that the distance is long, that I have to travel without a car, I cannot walk alone, no, I need to walk with him. That’s the reason. If I had a slim body, I would have said ahh I can walk alone to get there, but I can’t. It’s a long distance, for sure*
140. I: Mmmh ok. So what can we do to encourage the men to get involved? How can we help them to play a role?
141. *R: I have failed to answer*
142. I: Mmmh how can we teach the men about cervical cancer?
143. *R: We can tell the men that they should allow their wives to go to the clinic to do cervical cancer screening. The should not stop/discourage them. Sure*
144. I: Mmmh, how else can we teach them?
145. *R: I have failed to answer*
146. I: Mmmh ok, thank you. So now I want to know a lot about what you already know, right? Is there anything new that you have learned related to cervical cancer or to do with cervical cancer screening, which you didn’t know before? Before this research started?
147. *R: Yes, there are. Because I didn’t know that when screening they insert metal equipment, and pull the cervix, the put some medication and checking to see what the cells look like, sure*
148. I: Mmmh ok, is there anything else, that you didn’t know?
149. *R: No there isn’t. [ inaudible] there isn’t*
150. [ *all laughing]*
151. I: What? What are you thinking? [ laughing]
152. *R: [ laughing]*
153. I: [ laughing] Huh? Mom?
154. *R: Nothing!*
155. I: What are you thinking?
156. *All: [* laughing]
157. *R: looking at the time, that its late*
158. I: The time? It’s true, it is late. ok, we will try to hurry up
159. *R: ok*
160. I: Ok. So who do think should be screened for cervical cancer?
161. *R: A woman*
162. I: which women?
163. *R: women, from what age?? But up to 50.*
164. I: up to 50 years of age?
165. *R: I should say up to 49 years of age. From 50 years upwards they said no*
166. I: Why did they say no to those from 50 upwards?
167. *R: That person is grown*
168. I: Ok. How frequently should these women be screened?
169. *R: These women should be screened after every 3 months [ inaudible], after three months, they should wait two months, the third one they should be screened. Sure*
170. I: Mmmh. Ok. Why do you think they should be screened every three months?
171. *R: So that a person should know her body is. Sure*
172. I: Mmmh. Ok. So what do the women in your community think about cervical cancer screening?
173. *R: The women in our community*
174. I: mmmh
175. *R: Think that, others rush to get screened while others refuse*
176. I: Mmmh
177. *R: They say that that’s how God created them, so it should be like that, while others rush to get screened so they find out how things are in their bodies*
178. I: mmmh
179. *R: So we got screened and we told them no, the goodness is when you get screened you get to know how your body is, yes*
180. I: Mmmh ok. Do you think the women in your community understand the importance of cervical cancer screening?
181. *R: Yes, some understand while others do not understand. Those who don’t understand are the ones who refuse to go for screening. Yes*
182. I: Ok. Why do you think that they understand*?*
183. *R: I answered you that it because they go, to the screening place, [ inaudible]*
184. I: [ laughing] Thank you. In your thinking, do you think that the women in your community are happy to get screened and to receive treatment?
185. *R: Yes*
186. I: Why do you think that?
187. *R: Because they go and get screened. Sure*
188. I: Mmmh ok, what do you think would stop a woman from wanting to get screened?
189. *R: Fear*
190. I: Fear. What else?
191. *R: The other thing is some women are just laidback think that it is not important, that even if they get tested it is not important.*
192. I: mmmh like they don’t take it serious?
193. *R: mmmh*
194. I: What else?
195. *R: There is nothing else*
196. I: On the fear, what do they really fear?
197. *R: They fear that they may be found with it. That they will remove the uterus. That’s what the women fear, sure*
198. I: Why would they fear to have their uterus removed?
199. *R: I don’t know the reason why*
200. I: Mmmh. Ok. What other challenges would women face when they want to get screened or when they want to access the service for cervical cancer screening? What challenges can they face?
201. *R: It can be that they are menstruating. Yes, because if you are menstruating you cannot get screened. They cannot screen that person, they say, sure*
202. I: What else, apart from the menstruation?
203. *R: Mmmh, I have failed*
204. I: That relates to the husband maybe?
205. *R: Maybe the men can refuse them maybe not go to for screening*
206. I: Do you think the men can do that?
207. *R: yes, there are some men who can do that. They say “don’t go for screening”*
208. I: Why do they refuse?
209. *R: Because of fear, they think that if the wives go for screening and they are found to have cancer of the cervix they will remove it. Yes*
210. I: Mmmh, ok, how can the woman’s friends or relatives also prevent the women from going for screening?
211. *R: They can help by encouraging the man to let the wife go to the clinic for screening*
212. I: mmmh, especially the friends or relatives of the woman preventing her from getting screened*?*
213. *R: For her not to be screened?*
214. I: mmmh. How can they do that?
215. *R: oh*
216. I: A person failing to get screened because of
217. *R: Her friends have said no,*
218. I: Her friends
219. *R: oh! [laughing] Her friends have made her afraid, that they put metal equipment there? [laughing]*
220. I: Yes [ laughing]
221. *R: “Don’t get tested” [ laughing] Others say “a child comes out of there [ laughing] oh, go and you will see for yourself” [ laughing] but you! “It feels like a huge baby will come out from there”*
222. I: They say those things?
223. *R: yes, women speak like that*
224. I: So in your thinking, how do you think the cervical cancer screening should be done to ensure that many women get screened or checked?
225. *R: It’s up to you doctor to try and go into the communities, to tell people that screening is good because you get to know how things are in your body. Because if you don’t get screened you cannot know how things are in your body. Yes, so it’s good for one to get screened, so they know how they are. Sure*
226. I: So how can these women be encouraged to go for screening?
227. *R: They should be encouraged that screening for cervical cancer, because, I will keep repeating myself, when one gets screened you know how your body is, while if you don’t get screened it means you have killed yourself. Sure*
228. I: Mmmh ok. So now I want to talk us to talk about self-screening. Collecting vaginal fluid and screening yourself. Let’s now talk about self-screening using a cotton swab to collect vaginal fluid on your own. To screen cervical cancer. A new method has been put in place to screen cervical cancer. It requires a woman self -collecting vaginal discharge using a cotton swab and taking it to a health Centre at a time when the women is free. Right? But still it is different from the screening that you underwent. But still if a woman self-collects the vaginal discharge using a cotton swab, she does not get her results at the same time. Yes, so she is supposed to come another time to collect her results or to come another day to collect the test results. What do you think about this idea?
229. *R: This idea is also good. Yes, it is easier to do that*
230. I: Can you explain how you think it is easier?
231. *R: It is easier because if a health center is near you can take the vaginal discharge and take it there to give the doctor. Then the doctor will screen it. Or is it the mobile Doctors who will be doing this maybe?*
232. *I*: Whether you to the Hospital or at (name of location) there is a clinic, you can take them there. Yes, or if there is another place where they provide health services. You can take them there
233. *R: Oh ok. So they send them here?*
234. I: These are just suggestions that…
235. *R: oh ok*
236. I: They would want to be screening that way, that a woman should do self-collection
237. *R: oh ok*
238. I: Then she should take the things to the hospital/clinic. How the clinics will be testing, there I can’t know. Right? Yes. So you have said that it is an easier way, so I wanted to understand how think it is easy?
239. *R: It’s because you will just take the collected things and give the doctor, and they will give you a specific time to come and collect your results. It means they will go and hear their results. Whether you have the cancer cells or not. If you have them, you will be told what time to meet with the doctor they will go to the hospital. Sure*
240. I: Mmmh. What other advantages are there in using this method which you see?
241. *R: The advantage is what I have just told you, that it is brief. Yes*
242. I: Mmmh. What are the disadvantages that you see?
243. *R: The disadvantages that I see, maybe the doctor who receives those things, some can get lost/he can lose some. That is also a problem, because then you cannot not know how your body is. While with this instant screening you get to know everything right there. Sure*
244. I: Ok. Another disadvantage*?*
245. *R: No, I will not answer*
246. I: Ok. So for this, where do think is the best place to collect? At home or where?
247. *R: At the clinic*
248. I: At the clinic?
249. *R: Yes*
250. I: Why do you think they should be collected at the clinic?
251. *R: At the clinic is where there is a good storage place, yes*
252. I: Ok,
253. *R: Sure*
254. I: Mmmh, so do you think this method is dependable, how dependable is it?
255. *R: The dependable way is that of meeting the Doctors. This other method is not very dependable like I already said that maybe it can get lost on the way. Sure*
256. I: Ok, thank you very much. How can you differentiate self-screening or self-collection of vaginal discharge and the other method though which you were screened?
257. *[silence]*
258. I: How can you differentiate the two methods?
259. *R: Differentiating between self-collection of vaginal discharge and this method?*
260. I: mmmh
261. *R: The difference is that this one when they screen you and find you with the cancer cells, they spray the medication right away, while the one you do yourself with the cotton swab. You cannot be assisted quickly, that’s the difference. You can receive assistance after a long time*
262. I: mmmh. Ok. How do you think other women in your area will think about this self collection of the vaginal fluid? To be screened for cervical cancer?
263. *R: only if they can go to the clinic to meet the doctors, yes, or else if they meet us and we tell them that there is another way of collecting vaginal discharge using a cotton swab and getting it tested at the clinic. That way they can find a change to go and get screened if they have cancer. Sure.*
264. I: Ok, so what would the women think? About this method of this self- collection screening? What would they think?
265. *R: The women would think that this is a good method. But the would worry that it would take long to get the results. That’s the difference*
266. I: Mmmh ok. Why would they think that it is a good method?
267. *R: They would be meeting the doctors. Yes, because people say there are metal clamps They do it like they are pulling the head of a child [????] So people fear that. Sure*
268. I: Mmmh ok, thank you. Do you think that more women would want to be screened through this vaginal discharge self-collection?
269. *R: Yes, they would get screened. Sure. Mmmh, that’s what people say, they are afraid of the metal equipment*
270. I: yes
271. *R: There are metal things there. And others turn back on the way, saying there are metal things, but this other method they would say that its easier [ inaudible]. Sure*
272. : Ok. Thank you. What challenges do you think the women would have regarding the self-collection? What challenges would they have?
273. *R: The challenges are those I said, here the Doctors could take time, and the cancer could have spread through the uterus and maybe even to other parts yes, the results have not yet been received. So that is a big challenge as opposed to going to the clinic and getting screened by yourself right there to know your results quickly*
274. I: Mmmh how about challenges related or to do with the actual discharge collection process, for them to collect their own vaginal discharge? What challenges would the women face?
275. R: Nothing
276. I: Nothing? Ok, thank you very much. What do you other think are other challenges that could be there, if any, that you think women would want to do self-collection apart form the fear of the metal equipment? What others things can cause fear for them to do self -collection?
277. *R: Some women just fear going to the clinic. They really refuse to go to the clinic to meet Doctors. This could be a reason why they cannot do the self-collection of the vaginal discharge to go to the clinic to get screened*
278. I: Why do they refuse?
279. *R: Fear. Some were just born with fear; they say “We can’t there” Go we will meet when it’s time for death, there is no now who will wake up at [inaudible]*
280. I: But what do they fear?
281. *R: They fear the Doctor*
282. I: [laughing] That he will do what?
283. *R: I don’t know*
284. I: Or maybe they feel shy?
285. *R: Yes, some are shy, but a mother, how do you give birth at the clinic and feel shy to get screened?*
286. I: Mmmh ok, thank you. So now we want to talk about your comments for the future, right?
287. *R: Mmmh*
288. I: On how the process of screening should be done. In your thinking should the Ministry of Health consider this method of self -collection of the vaginal discharge to add on to the original method which you underwent in its procedures of cervical cancer screening?
289. [ silence]
290. I: From your view point. Should this method be added in the methods of cervical cancer screening? That a person should choose the method they want, whether it’s the one that uses the metals or the self-collection of the vaginal discharge?
291. *R: This self-collection method is good, but not so good as I told you earlier, that for you to know how you are it will take time, or will the results come the same day? Will they come out the same day?*
292. I: There we don’t know because it was not explained on this questionnaire, right?
293. R: oh oh,
294. I: But it is a method that the health care system wants to use to help women, ok?
295. *R: Because if it was giving the results right there it would have been good, but if it’s that you will have to go and come back another time to be given your results, its bad. The disadvantage is like I have told you already that by the time they discover or get the results, it could be that something has already gone wrong in the body, yes*
296. I: But they have explained that once you collect the vaginal discharge, you will be bringing them to a clinic, right?
297. *R: Yes*
298. I: “I have brought my sample here” my vaginal discharge collection here
299. *R: Yes*
300. I: Screen for me, so the medical personnel will take them for screening, after screening they get the result. So maybe they will tell you that, maybe you will bring the sample at 9 in the morning and maybe they will tell you to come maybe around 11 or maybe to come around 1 to get your results. So maybe after you collect your results is when the Doctor will determine when to give medical assistance or medication or how, right? But because they didn’t indicate on this paper, we also don’t know. So in your response you have said that if the assistance is give quickly it is ok. Fine. So do you think this will make it easy for women to go for cervical cancer screening?
301. R: Yes, it would be easy because you can just get the vaginal discharge and go to get it tested it will be easy sure
302. I: Ok, thank you. Which groups of women do you think would be most be crucial to do the self-collection of the vaginal discharge for screening?
303. *R: The young girls*
304. I: The girl groups?
305. *R: yes, and the slightly older ones. Yes*
306. I: Why do you think that?
307. *R: I am thinking that way so that they know how their bodies are,* yes
308. I: Ok, and which groups should not be doing the self-collection of the vaginal discharge to do the cervical cancer screening?
309. *R: mmmh, I have failed to answer*
310. I: mmmh ok. Do you have any questions or comments you would like to add?
311. *R: No there* isn’t
312. I: Ok. Let me thank you very much for taking part in this discussion, I am aware that there is no time, but your time is important but also all the information you have given us will help a lot in advancing the cervical cancer screening work in Malawi, especially when they are doing sensitization, on what kind of messages will be given, thank you so much
313. *R: Thank you*

End time: 51: 56
