# Supplementary material for: Perceptions of cervical cancer and motivation for screening among women in Rural Lilongwe, Malawi: A qualitative study
Source: PLoS One. 2022 Feb 7;17(2):e0262590. doi: 10.1371/journal.pone.0262590 (PMC8820632; doi:10.1371/journal.pone.0262590)
Supplement: S3 File — (ZIP) [file pone.0262590.s003.zip › VIA 278.docx]

**PID: VIA 278**

**DATE OF INTERVIEW: 9 Nov 2017**

**INTERVIEWER: 466**

**TRANSCRIBER: 969**

**TYPE OF INTERVIEW: 12 weeks follow up**

**KEY: I= Interviewer, R= Respondent**

**Interview Summary**

Client reported for her 12 week visit and she IDI was conducted after the visit. She reported to have decided to be screened because she wanted to know how she is in her body. She explained that at first she was afraid to have the screening test because of the misconceptions going around in her village that the team is going to burn the cervix so that women should no longer have children. However, she made her mind and had the test. She has now seen that many women are willing to have the test in her village. She feels men should be encouraged to escort their wives to the hospital so that they should know the type of treatment they receive as some do not believe that their wives really came to the clinic. On sensitization, she feels the hospital people should continue going to the communities and spend more time as women take time to make a decision. On self-collection of swabs she feels being screening by the doctor is better because they test them so many things including pregnancy tests.

1. I: thank you for coming. I appreciate for your coming and everything that you are going to say. I am part of a team of data collectors from UNC Malawi. Where we are now is called UNC Project. yeah, your ideas are very important to us they will help us understand the importance of cervical cancer screening campaigns. As you answer the questions... there is no right or wrong answer. Everything which you are going to say is very important. We want to learn a lot from you. Whatever you are going to tell me is private and confidential it shall be used only for the purposes of this study and help improve health services. I will record the discussion so that later I can be able to write down everything which you are going to say since I cannot be able to remember everything you can say. Please note that whatever you are going to say shall never be linked to your name or any kind of your personal information that identifies you. First of all I would like to know about cervical cancer screening and the treatment which you received. Can you tell me your understanding of the cervical cancer screening and the treatment which you received?
2. *R: The cancer treatment which I received, they made me lie on the bed and started to screen me. Thereafter they told me that I have cancer and later they said we will burn it... then they said we are done you may rise...eeeh*
3. I: So what was your understanding according to what happened?
4. *R: They said with what we have done on you, you should spend six weeks without having sex. I understood and followed the instruction-I spent six weeks without sex*
5. I: So what method did they use when they screened you?
6. "..." Or what instrument did they use to screen you?
7. *R: They had a glass, and they used certain metals which I didn’t see.*
8. I: So why did they screen you?
9. *R: They wanted us to have healthy life*
10. I: What really did they want to see as they screened you?
11. *R: They wanted to see cancer cells that are in our body.*
12. I: After they explained to you that we have find you with cancer cells, what other things happened?
13. *R: I was passing water?*
14. I: I am referring to this day when they tested you... what else happened apart from ... entering into the room to screen you? or what really happened upon your arrival?
15. *R: After we arrived, we just saw that a big glass was twinkling and not that I could see the whole of it... just saw the glittering light*
16. I: From which side was it twinkling?
17. *R: from the lower part*
18. I: Oh... that was when you got inside the room where the screening was being done?
19. *R: yeah*
20. I: but what happened before you got inside?
21. *R: before we got inside I was so afraid and frightened*
22. I: so you could just arrive and straight get into the room?
23. *R: no, I first joined groups of my friends*
24. I: so what were you doing in those groups?
25. *R: we were discussing that: "what are we going to face inside; of what manner is cervical cancer". Then we could just say leave it like that we are going to see... later, one colleague gets in, then comes out. She explains to us everything... Thereafter we all gather courage and determined to go in since we want life.*
26. I: I want to know your thoughts about cervical cancer campaigns. Why did you choose to get screened or take part in that study?
27. *R: I wanted to know my status. And after I came from there I went to my friends and explained it to them. Some were saying that, "others are dying in the cancer treatment process... they use spanners and such and such..." As such some were being discouraged and never took part. As we taking now some are admiring us... and one of has come with us.*
28. I: ooh, you have come with someone, dis she attend the first session?
29. *R: No she didn’t... she has decided to come so that she can also know her status. so she has been sent to the other side*
30. I: mmm... Alright thank you. was there anything that you were anxious about before the screening happened? yes you explained that you were afraid before the screening, but now before you knew about the screening what made you anxious?
31. *R: Nothing made anxious. was just saying better I go and get screened so that whatever is problem in my body should come out. after I got screening I feel much better than before*
32. I: Before you got screened what were you feeling?
33. *R: I was feeling itching and my back ached so much... now all that stopped I am very quite ok*
34. I: Was there any other fear you had before the screening?
35. *R: there wasn’t*
36. I: so after you got the results of your screening and being told that you have been found with cancers cells, how did you feel?
37. *R: I was very happy because I knew that doctors will help me*
38. I: Mmh, after they told you that madam we have find you with cancer cells, I want you to tell me how you felt in your heart as a human, how did you feel?
39. *R: I didn’t feel hurt, I was happy that they find me with it. It made a difference comparing to the times we spent with women at home... so with this I knew that whatever was inside me is now solved*
40. I: Alright. What do you think happened well at the time they were screening you?
41. *R: I have forgotten some of these*
42. I: you forgot? Can you please try to remember what you went through? "..."
43. *R: I have forgotten*
44. I: you forgot?
45. *R: yes, some of them I have forgotten*
46. I: In the beginning you explained to me that they beamed you with a big bulb, and inserted you metals etc... So I want things like that. According to you, what do you think in such happenings went on well?
47. *R: "..." I have forgotten*
48. I: What do you think could have happened well?
49. *R: You mean when they screened me?*
50. I: Whether it was during screening or counselling, what do you think didn’t happen well? Or what should have happened for you be satisfied?
51. *R: "..." [very long pause]*
52. I: Everything was ok?
53. *R: Yes everything went on well*
54. I: Which part did you find easy?
55. *R: The screening part*
56. I: How easy was it?
57. *R: Because it was like after you get into the room they made you lie on the bed and if you are sick they could call another doctor. Then they could tell us that, "we found you with the disease and we have burned the cancer cells here is the tool we used"...[I: mmm]*
58. I: Which part did you find to be difficult?
59. *R: Difficult part...*
60. I: feel free we want to hear a lot from you. So don’t be afraid what you are going to tell us will help improve future cervical cancer screening services. we are together not so? don’t be afraid tell us everything... this is your hospital... not so... [laughs: hahaha...]... we are here because of you, feel free. Which part was difficult to you that time?
61. *R: I didn’t find anything difficult, I felt everything was just ok and to me everything was good.*
62. I: Alright thank you very much. After they screened you, you were required to come for a follow up visit. It can be difficult to some people or even you to come back for the follow up visit. Did you have any challenge when coming back for the follow up visit?
63. *R: I didn’t have... they just said that we had to come back to the hospital*
64. I: Io, I mean problems making you to fail to come here even though you desired greatly to come?
65. *R: No there wasn’t anything like that*
66. I: What other problems can women from your area have that make them fail to come? for there are some women who are not coming, what could be their problem?
67. *R: Some say, "why are they requiring us to go in groups of two, that might be satanic, it is not a hospital". like myself I was assigned to come with a colleague, and they told us to come last month... and then... they came home but they didn’t find me so they left "a thing" (money for transport) so I went to collect it from (Name of place). So he said some are afraid they saying that they are surprised to be told that they should be going in groups of two so it must be satanic. So I said no, I won’t buy those idol speculations... that is a hospital, from where did you hear... I have a problem I will go if you don’t come with me... so different people encouraged me.*
68. I: So it was your friend saying all this?
69. *R: Yes, people from home told me to come, they said that they don’t kill people at the hospital. Here I am today and I have come alone*
70. I: So, that friend of yours also got screened during your time?
71. *R: Yes*
72. I: What really does she think happens here to call it satanic?
73. *R: She thinks that when we come here we are being miss treated like sucked blood so that after we get home, we die; and yet they don’t know really what is happening here. Whilst some from our home side want to come.*
74. I: Apart from thinking that what happens here is satanic, what other problems are making women fail to come here? maybe they can’t think that it is satanic, but have a different problem making them unable to come here?
75. *R: May be money*
76. I: Mmm mmh
77. *R: Sickness, money problems*
78. I: Anything else besides money and sickness: what else?
79. *R: Marital problems*
80. I: Mmm mmh, what exactly about these marital problems?
81. *R: "..."*
82. I: What can happen in marriage that can make a person to fail to come?
83. *R: It is possible that when your day to come here is closer you may quarrel with your husband and even fight each other as well. so you say with what has happened if I go there something bad might happen, its better I don’t go.*
84. I: So what exactly can be the cause of this fights?
85. *R: May be to do with the cancer...*
86. I: What about it?
87. *R: ... The Man may say that, "you have been going there since, what exactly do you go to do, may be you go seeing boyfriend"*
88. I: So its like forbidding you?
89. *R: yes.*
90. I: Mmh mm. alright other problems? ... you have talked about a number of problems like money etc... so how can we help these women in order to overcome such problems?
91. *R: May be visiting them in their homes*
92. I: what else can we do?
93. *R: "..." That’s all*
94. I: What should we be doing after we visit them in their homes?
95. *R: You should tell them that you are screening women for cancer, some argue about it*
96. I: So its like trying to educate them?
97. *R: Yes, teaching them*
98. I: How helpful do you think this is?
99. *R: "..." It can be helpful to someone who doesn’t understand and always have bad feelings towards the screening.*
100. I: Now I would like to know about the support you get from your husband or community. did you discuss with anyone about getting cervical cancer screening?
101. *R: Yes*
102. I: Who did you talk to?
103. *R: My father, my relatives that’s all... also the chief*
104. I: Alright. So after you told your father what did he say?
105. *R: He said that I made a very good choice... by then I was bidding farewell that I am going to the hospital. And so he said ok, go well.*
106. I: What about your friends, what did they say after you told them?
107. *R: They said we are admiring... it’s just that we are on our monthly period and we can’t go with you.*
108. I: mmh mh alright, what about the chief?
109. *R: He says, "You did well so that people can emulate from you"... that was to those who say "these are useless I better go and do other thing"*
110. I: Were there questions which they were asking?
111. *R: Yes they did. They asked that when we come for follow up visit do they screen us again. so I answer them that no they don’t they just ask us questions*
112. I: So does your husband know that you had cervical cancer screening?
113. *R: No he doesn’t because he is far.*
114. I: where is he?
115. *R: he is in (Name of district)*
116. I: Doing what?
117. *R: Its his home.*
118. I: So you moved from (Name of district)?
119. *R: Yes*
120. I: Why?
121. *R: There was problem that happened there... I went there in (Month) with my daughter and she died after some time upon our arrival. He is the one who sent me money to go there. so I stayed for a month and the second one it is when my daughter died. so after seeing what was happening there I decided to come back to my home village.*
122. I: By then you had not screened for cervical cancer?
123. *R: So I just come back here and since then he has never come to see me. He just make calls.*
124. I: I am sorry it is a painful thing
125. *R: It happens*
126. I: Its a hard thing but still we just had to ask.
127. *R: Only those who ask know the road to where they want to go. (meaning you can only know about the problems when you ask)*
128. I: After testing you, you were told not to have sex for a month. Was this a hard thing for you?
129. *R: No, there wasn’t any problem*
130. I: mmm mmh...mmh so how was it that you were able to take the whole month without sex?
131. *R: Because I don’t have a man*
132. I: So do you think that men should get involved in women's cervical cancer screening, here i am taking about any other woman?
133. *R: Yes they should get involved*
134. I: Why are you thinking that way?
135. *R: Because a lot women here are dying because of cancer... so it’s good that they should so that they know what they should do in order to stay longer.*
136. I: So how do you think they should get involved?
137. *R: learning from their friends and even you doctors.*
138. I How can they learn from friends
139. *R: It’s just how I have come here when I go back home I teach him that this is how it goes... this and that... it’s not a problem and so on. so it means that some will be bold enough that when they come again (doctors) will they will go for screening*
140. I; So how can they learn from us?
141. *R: After when you come to the village... or some volunteers themselves to come like we have done*
142. I: Alright... what about the issue that they escort their spouses when coming to the hospital, what are your thoughts?
143. *R: It’s for him to see the type of treatment his wife gets. They should see it together... maybe when a wife comes here she lies or when she comes back and explains to him everything that happened a lot of men disagrees. So it’s good to come to the clinic together.*
144. I: So in your opinion, how do you think should we go about encouraging men to take part in these screening campaigns?
145. *R: Visiting them in communities like you did at (name of place). Even in schools you can be coming and teach them... inviting both men and women*
146. I: So we should only be educating people in schools and hospital?
147. *R: Even here*
148. I: What else?
149. *R: Even here and at antenatal clinic*
150. I: Now I want us to look at what people know and the fear they have of getting cervical cancer. Is there anything new you have learnt about cervical cancer or cervical cancer screening which you didn’t know before the study?
151. *R: …Which I didn’t know?*
152. I: Yes which you didn’t know... is there anything strange about cervical cancer which you didn’t know?
153. *R: Yes i didn’t know*
154. I: what didn’t you know?
155. *R: I didn’t know how they screen cancer, how they test it and again how they treat it by burning cancer cells.*
156. I; Is there anything else?
157. R: No
158. I: Who do you think should get screened for cancer?
159. *R: Women*
160. I: What type of women? women are of different types. which ages or years?
161. *R: 25 years, 30, 40, even those that are not pregnant and should not be in menstrual period*
162. I: Mmh mm... if you say should not be in Menstrual period what do you mean?
163. *R: Should not be in menstrual period*
164. I: Apart for that who else?
165. *R: Normal woman*
166. I: what do you mean by that? Be open... if you say normal, what do you mean? when you say a woman is normal, what makes her exactly to be normal?
167. *R: Can you ask again.*
168. I: We were talking about who can get screened and you listed a number of them... and you said that another type is that the woman should be normal. So I would like to understand what that mean?
169. *R: When we are going to screen for cancer you have to take a bath and be smart. for some just go as long as they get the treatment*
170. I: Alright. How often should women be screened for cervical cancer?
171. *R: Should do that after years*
172. I: How many years?
173. *R: Three or four*
174. I: What makes you think that way?
175. *R: "..." So that they see how you are in your body*
176. I: Is there any other reason why you have chosen that 3 or 4 years... "..."... nothing?
177. *R: Yes there isn’t any other reason*
178. I: Now I would like to know your ideas on future cervical cancer screening campaigns. what do women from your community think about cervical cancer screening? "..." women from your area what do they think about cervical cancer screening?
179. *R: "..." I have forgotten*
180. I: What do they say about cervical cancer screening?
181. *R: they ask, "Where do they screen for cancer"? so i always answer them that they screen it from the Vagina and that when you go for screening you should not be pregnant, should not be menstrual period and they say... ooh ok.*
182. I: So what do they think after you tell them this?
183. *R: So they say when they are screening do they screen using a glass or what exactly do they do? So i say they test with spanners to see the growth of the cancer cells and as well applying some medicine and the burning...*
184. I: Oh ok... what do you think they know about cervical cancer? do they know much or little or what exactly do they know? What is their level of knowledge?
185. *R: I have forgotten*
186. I: what about issues of discrimination, how is it in your area?
187. *R: discrimination? (she didn’t understand the local term)*
188. I: when I say discrimination do you know what it means? Being discriminated against... because you have a certain problem "..."
189. *R: You cant know that*
190. I: How is it in your area?
191. *R: [coughs]*
192. I: Are those people who were found with cancer or cancer cells get discriminated against?
193. *R: Yes*
194. I: What do they do to them for you know that they are being discriminated? How are you living with people in your area especially those who know that you were found with cervical cancer?
195. *R: I am living with them without any problem*
196. I: So how come you are saying that there is discrimination? What about other women who come here who were found with cervical cancer, how are they coping with other women?
197. *R: No problem*
198. I: what do they think about facing a risk of having cervical cancer?
199. *R: They think that perhaps they have cancer*
200. I: What makes you think that?
201. *R: It because of how they feel in their body*
202. I: Alright do you really think that these women understand about cervical cancer screening?
203. *R; Yes they understand*
204. I: What makes you say they understand?
205. *R: They participated in the screening exercise...*
206. I; what did you just say?
207. *R: They got screened and now they realized that for one to screen for cancer needs such and such*
208. I: Did they get screened?
209. *R: No they didn’t... but they want*
210. I: So you think that they understand the importance?
211. *R: Yes*
212. I: Mmh mm... ah so, how do they understand about the importance of cervical cancer screening?
213. *R: They understand it because you save your life*
214. I: Do you think that women from your community are interested in getting screened for cancer and its treatment?
215. *R: They are not interested*
216. I: What makes you think that?
217. *R: When the doctors come and announce that there is a gathering tomorrow, they don’t come. they wait till they see their friends have gone... and later start regretting that if they had participated*
218. I: Alright... in your thoughts, why don’t they want to go?
219. *R: They say it is idols*
220. I: they say it is idols... So when they say that what do they really mean?
221. *R: Idols?... "..." [I: mmm]... they tell me that they just use us... and want to make fool of people that there is such and such and your problems in the body will end. and thus they say it is useless better I do something else*
222. I: What do you think can make a person not to want to get screened? Apart from that it is idols, mere trick... what can make people not to want to get screened?
223. *R: They don’t want to know about their health status*
224. I: What else?
225. *R: Husband forbidding them from having the screening test*
226. I: Mmm mmh, another thing?
227. *R: They think it is sterilization type of family planning*
228. I: What did you say?
229. *R: Whey they screen the cervix for cancer they think they are also closing the womb that they should never have children again.*
230. I: They think in that way?
231. *R: Yes*
232. I: Oh ok... another thing?
233. *R: There isn’t anything more*
234. I: Oh ok... [laugh] We want to hear a lot from you... this is just an interaction. What problems can women face as they receive cervical cancer services?
235. *R: There are so many problems. When you are going for testing people ask you that you have made up your mind to go there and have the screening?... what will they do… they will burn and close your womb. Even myself during the first time I wanted to return back home… they asked me, “Where are you going”? I said: "to (Name of place)", to do what: test for cancer, they are also sterilizing women that they should never have children. "So if you go, you won’t be desired anymore by men because they will burn your womb". And I replied that I will see what will happen right there provided I get helped*
236. I: What are other problems apart from what you have said?
237. *R: Getting sick, pregnancy "..."*
238. I: Ok. In your opinion how should cervical cancer screening services be offered to ensure that a lot of women volunteers to test?
239. *R: We should be encouraging our friends to do the same tomorrow*
240. I: So you should be telling your friends? who should be doing that?
241. *R: Us*
242. I: By "Us" you mean... "..." why are you supposed to tell your friends?
243. *R: Because we saw wonderful things. the way we are today we differently as compared to how we were*
244. I: so You mean you who got screened should tell your friends?
245. *R: Yes, so that they can also get courage... even doctors can also be explaining to them*
246. I: Alright, we can surely tell them, but how can we encourage them that they reach the extent of getting screened?
247. *R: Come near, as you came to (Name of place) spent some weeks and come again you shall see how they can respond.*
248. I: Ok. now i want us to talk about self-testing. Now lets us talk about a person taking her own cervical cancer testing samples. a new method of cervical cancer screening has been discovered. This method involves a woman taking samples of vaginal fluid using cotton and deliver it to health centre or hospital any time when the woman is free. However unlike the method you used, the woman is not going to the results the same time. she has to wait for some hours or come after a day. What are your thoughts about this?
249. *R: "..." [Inaudible segment]*
250. I: Have you understood the question?
251. *R: Yes*
252. I: What have I said?
253. *R: You said that: "woman taking samples of vaginal fluid using cotton and deliver it to health centre or hospital...*
254. I: ... Take cotton and insert it in her vagina... what are your thoughts about it?
255. R: It can’t help
256. I: Why are you saying that?
257. *R: Because they can’t reach the cervix*
258. I: Why do you think so?
259. *R: There is a difference between a doctor testing you and doing it by yourself*
260. I: So you are saying that is not helpful with the way i heard you
261. *R: Yes*
262. I: So... anything how bad it be, still has got some good part of it. What advantage do you see in this method of self-testing? What goodness can be there?
263. *R: There is no problem in self-testing, but some are afraid to test themselves so they go to the hospital*
264. I: This self-testing I am talking about do you know it? It’s like taking cotton and insert in the vagina to extract vaginal fluid and take to the doctor for him to test... this is a newly discovered method. So we want to hear your thoughts about this method
265. *R: It is a good method*
266. I: How good is it?
267. *R: You can do it at your own time depending on how you feel in your body... so you decide to take cotton and do whatever with it and take it to the doctor*
268. I: Could you be interested to get screened using this method?
269. *R: Yes I can do*
270. I: What are the advantages of this method? so that when it gets approved they should know about its advantages
271. *R: Its advantages?*
272. I: Mmh
273. *R: "..."*
274. I: Alright what is the disadvantage of this method? don’t be afraid, don’t answer to impress me but answer only these things you think are required to happen... you won’t tell. Alright so do you think people should be taking samples of vaginal fluid from home?
275. *R: No that is not possible*
276. I: Why do you think so?
277. *R: They should do that at the hospital so that they can clearly see what is in their body at the same time*
278. I; Alright, do you think that this method is reliable?
279. *R: It is not reliable*
280. I: Why do you think so
281. R: Because you can’t know a lot of things
282. I: What can make you not to know a lot?
283. *R: You can take it from home, and as you walk you can find that the cotton is not there so as you get to the hospital... [I busted out into laughing: hahahah...]*
284. I: Where will the fluid go?
285. *R: Its been sucked by the bag*
286. I: Alright. What else make you think it is not reliable? ... "..." alright, how do you compare and contrast this method and the one which was used on you?
287. *R: The one that was used on me is better*
288. I: Why do you think so?
289. *R: If I had taken the fluid from and brought it here it would not have been possible to find me with cancer. Because I might bring the fluid when I am pregnant. But that time they were testing everything to see how you are in your body*.
290. I; Which method would you prefer?
291. *R: the screening done by the doctors*
292. I: What do you think can women from your area think about taking their own vaginal fluid using cotton and take it to the doctor for screening? ... Have understand the question? After you tell them that there is method of cervical cancer testing that involves.... what can they think?
293. *R: They can do it by themselves so that they get help*
294. I: What can make them do that?
295. *R: They want to see what is in their body*
296. I: Why would they opt for this method?
297. *R: Because they will have full control in using the method no one will see their nakedness*
298. I: They don’t want someone to see them naked
299. *R: Yes*
300. I: Do you think that a lot of women can choose to get screened using this method?
301. *R: No they can’t choose this method. Like I said, you can do it home so considering the distances we have to travel like from my home (Name of place) to (Name of place) is very far so you can find that the fluid is not there if you did not do it correctly.*
302. I: Let’s assume that the screening is being done at the hospital, can they choose to collect the swabs themselves for testing?
303. *R: No that is impossible, the doctors should do it.*
304. I: I mean taking vaginal fluid by themselves
305. *R: They can take*
306. I: So you think a lot of women can choose to take the fluid by themselves?
307. *R: No.*
308. I: Have you answered me yes or no?
309. *R: They cannot be taking it by themselves*
310. I: They cannot take it by themselves why?
311. *R: Doctors should do it.*
312. I: What problems can women face in the conduct of taking vaginal fluid by themselves for cervical cancer screening? What problems can they face?
313. *R: I have failed*
314. I: Alright, thank you very much. What are your fears about people doing self-collection of swabs for testing?
315. *R: Fear of getting screened*
316. I: So your fear is about people getting screened?
317. *R: Mmm*
318. I: Any other fears? Alright which reasons do you think can make women not to be interested to take vaginal fluid samples for testing?
319. R: *(Not clear)*
320. I: What make you think they would prefer the sample to be taken by a doctor?
321. *R; It is the doctors that know everything about the person*
322. I: Alright now lets see your comments on the future of cervical cancer screening campaigns. in your opinion, should the MoH recommend the method of taking vaginal fluid samples using cotton as another method for cervical cancer screening here in Malawi? apart for the method you used, should they add the one where people take samples by themselves?
323. *R: Yes they can take*
324. I: Why are you thinking that way? Why should there be this method of self-testing?
325. *R: Should we say a person can’t take the samples herself? Should test herself?*
326. I: No, just taking the fluid sample by herself and the testing will be done by the doctor... so you said that this method should be recommended so, I am asking why should it be added?
327. *R: It’s an easy way of testing cancer... they have seen that the way of taking your own fluid sample is easy and give it to the doctors*
328. I: Is there any other reason apart from that it is easy?
329. *R: "..."*
330. I: Alright, do you think that this can make it easy for women to get cervical cancer screening?
331. *R: It is difficult*
332. I: How difficult is it?
333. *R: You cannot take the samples by yourself at home*
334. I: We are not talking about where one can take the samples, it is about "taking fluid samples" by yourself and deliver it to the doctor... so I wanted to know if it is difficult... especially can it make cervical cancer screening easy to women?
335. *R: Yes it can*
336. I: Why?
337. *R: Because they will do it by themselves and get the vaginal fluid to the doctor and wait for results*
338. I: Alright, what groups of women would be suitable for using this method that involves the taking of samples of vaginal fluid?
339. *R: I have failed*
340. I: Alright, which type women wouldn’t be suitable for using this method that involves the taking of samples of vaginal fluid and deliver it to the doctor?
341. *R; Pregnant women*
342. I: Other women?
343. *R: … Who are on menstrual period*
344. I: Other... why are you saying that a pregnant woman should not use this method?
345. *R: They don’t test for cancer on pregnant women*
346. I: This testing will only involve taking vaginal fluid and nothing else will be inserted and the doctor will only test the fluid
347. *R: That is difficult to me*
348. I: Alright that marks the end of my questions unless you have comments or questions... or would like to share your experiences which you didn’t as we interacted...
349. *R: You are saying that person taking vaginal fluid and give it to the doctor, if they test can they find cancer in it?*
350. I: It is a method which doctors are still researching about... so after they test it is going to indicate that this person has cancer cells.
351. *R: So how will they burn the cancer cells?*
352. I: That shall be communicated by the researchers and doctors. It is another method which researchers are trying to find out if it is going to be proper for people to test themselves... and again if you want to know more about this I can refer you to a doctor who knows much about cancer, and can tell you more
353. *R: Why do you say that pregnant women should not screen for cancer?*
354. I: What did they tell you?
355. *R: They said because of the pregnancy*
356. I: Because of pregnancy... what about the pregnancy?
357. *R: They said that pregnant woman doesn’t screen for cancer*
358. I: So you didn’t ask why?
359. R: I didn’t
360. I: So its good to ask the doctor who knows about cancer and ask why should a pregnant woman...
361. *R: There is one at our home tall and black... she comes with her husband; she tested and was found with pregnancy... do you know her?*
362. I: I don’t know her
363. *R: She comes here and she came the other week*
364. I: Is there any other question?
365. *R: No*
366. I: Thank you very much that the end of our discussion....

**END OF TRANSCRPT**
