# Supplementary material for: Perceptions of cervical cancer and motivation for screening among women in Rural Lilongwe, Malawi: A qualitative study
Source: PLoS One. 2022 Feb 7;17(2):e0262590. doi: 10.1371/journal.pone.0262590 (PMC8820632; doi:10.1371/journal.pone.0262590)
Supplement: S3 File — (ZIP) [file pone.0262590.s003.zip › VIA 370.docx]

**PID: 370**

**DATE OF INTERVIEW: 20 Nov 2017**

**INTERVIEWER: 466**

**TYPE OF INTERVIEW: 12 WEEKS FOLLOW UP**

**TIME: 52 MINUTES 34 SECONDS**

**TRANSCRIBER ID: 466**

**KEY: I= INTERVIEWER, R= RESPONDENT**

**INTERVIEW TEXT**

I: Thank you for meeting with me today. I really appreciate for your time and you should know that your input would be very helpful. We would like to hear your views right. I: Am working with a team of researcher from the UNC project in Malawi. So your input is very important to us because we would like to understand how best we could conduct cervical cancer screening I Malawi, so there is no right or wrong answer we will receive whatever you are going to tell us right. You should be free and tell us how you think right? Therefore, what you are going to tell us will be confidential and it will only be used to make this health program and questionnaire better. We would like to just help, we would like to know how best we can conduct cervical cancer screening so you should not be worried that my voice or what right? You should just tell us what you think we can help with right. Therefore, I will audio record our interview to help us with what was said but your name or any identifiable information will not be connected to anything you say. As I have said do not mention your name right, have you understood me?

*R: Yes I have understood*

I: All right, so can you tell me your understanding on cervical cancer screening and treatment that you received in the past 12 weeks? Tell me your understanding on the cervical cancer screening?

*R: It is just that my understanding I because I did not know anything. Since we just went when they called us when we went there it’s when they told us to also come here, so it just helped me, maybe because I didn’t know that I have a certain problem so it did me good because I discovered that I have a certain problem that they can help me with.*

I: Mmhmm, how were you screened?

*R: Aaah screening meaning what?*

I: Like they screened you…

*R: They screened those inserted metal instruments.*

I: Ok so when you went there to be screened right, the aim what did they say it was. The aim of being screened or why they were doing that?

*R: They wanted to see people who have cancer of the cervix.*

I: And your results how were they?

*R: Aaah they just told me to come again here they said the body tissue that the took its was found with infection so they just told me to acme here.*

I: Mmhmm

*R: So when I came here they gave an injection and certain medication they told to come again on the 7th, and on the 7^th^ of (Month) its when they told me that they are supposed to screen again but there are no instruments, its when they called me to come again today. So they have not told me properly that…*

I: Ok they did not tell you they just told you to come at such date.

*R: Yes*

I: Like that?

*R: Yes*

I: Ok so you have said that they inserted metal instruments and took body tissue right?

*R: Mmhmm*

I: What else happened? That happened that day.

*R: They said they are burning.*

I: They said why they are burning?

*R: They said they found an infection there right. So they were burning so I do not know if it was the beginning of cancer or what it was but they told me, they did at first then a certain man came and said we would like to burn where the infection has been found he is the one who did.*

I: Where you are saying they did the first time, what did they do?

*R: A certain woman is the one who did it the same day, she said she could not see properly it is when she called that man and came he is the one who burned, that operate.*

I: Mmhmm, so I would lie to hear your views on cervical cancer screening, like that you were screened right?

R: Mmhmm

I: Why did you choose to be screened? Alternatively, why did you choose to take part?

*R: It did me good because I wanted to know how my cervix is like.*

I: Did you have doubts? What is that made want to know?

*R: I did not doubt anything right. But I was hearing that women are supposed to go for cervical cancer screening so it made me lucky that if people have come in our community that means I have to go and be screened.*

I: Is there anything that you were worried of before the screening was done?

*R: No*

I: I Just thoughts that aah people have come here I should go and get screened isn’t there anything that was going through your mind?

*R: Aaah [laughs]*

I: Any worry?

*R: There was nothing that was going through my mind since I did not know if I can be found with the disease or not.*

I: So knowing about it, what good has it done too you?

*R: It helped me a lot because I did not know that maybe my cervix have got a certain problem right. Yes, so when I went and they told me it has helped me a lot because it like I have been relieved in my life.*

I: That is an advantage right?

*R: Mmhmm*

I: You have known, now the disadvantage that you have seen after being screened what it is.

*R: Aaah, like the problems that I faced?*

I: Mmh

*R: It is as if when they just did it I was discharging smelly vaginal fluid right.*

I: When they just did it?

*R: When they just did it after how many days I have forgotten.*

I: After they burnt.

*R: Yes, it is when I started discharging that vaginal fluid and my stomach and back were hurting. But later on they stopped*

I: Maybe for how long?

*R: The pain?*

I: Yes like the vaginal fluid?

*R: The vaginal fluid maybe 2 to 3 weeks but the others they did not take even a week for the pain no.*

I: Ok, so you have said you were hearing that women should go for cervical cancer screening. So when you saw that they have come you went right.

*R: Mmhmm I went*.

I: What is it that you heard about the screening? Alternatively, what you heard that women should go for cervical cancer screening what is it? What was the message?

*R: Because a certain friend pf mine cane here, she was here at Ethel Mutharika.*

I: Mmhmm

*R: Yes, she is the one I heard from at first when I came to start here then it is when she was telling me that they have told us that every woman is supposed to go for cervical cancer screening. Because maybe we just walk around without knowing that our cervix have got certain problems, so they took us, so it gave me interest that when the people came it gave me interest that I should also and get screened I should see how my cervix is.*

I: And myths about the screening? Alternatively, what people talk about the screening what is it? Other people?

*R: Since we people are different, some the feel like its good right, to go for screening while other people say eeh that means you are doubting yourself why are you going for screening? Some are afraid they said that it hurts. They talk about many things so for those of us who went like other people I was telling them that there no any other problem and it is not painful but also good for a person to know how you are.*

I: So as if some were saying its good some were saying it is bad right.

*R: Yes*

I: Those who were saying its good they were saying its good because of what?

*R: Like what am saying that it is good because they also knew how they are in their cervix*

I: Mmhmm, so you have said they did not tell you that your result are not good, that how are your results?

*R: On that, they just told me that I have been found with cancer. They did not tell me maybe because I did not ask but they just told me that your results the body tissue that we took we have found infection. They told me that, so when I came here they gave an injection and gave me medication so it is when they gave an appointment date that we should come again on the seventh. When we came, it is when they told me that am supposed to be seen again so the instrument are the ones they said are not available.*

I: Ok

*R: Yes they called me on the phone that I should come today*

I: Ok, so they have taken the body tissue and tell you that it has an infection right, how did it made you feel?

*R: It gave me fear.*

I: Why? Why the fear?

*R: My thought were like is my cervix destroyed? How is my stomach? So when I saw that I do not feel any pain I was as if iih let me still go and hear the results how they are now.*

Mmhmm, but when they took that and told you that it has an infection, that body tissue did you understood what it means.

*R: My thoughts now?*

I: Yes

*R: I thought that maybe and have found that it’s the cancer disease on the cervix, and on the other hand I was like maybe its another disease, but I did not know really what kind of disease but in my thoughts I was like oh maybe they have found cancer on the cervix.*

I: So why did you ask? That you should know here you were just thinking right, that maybe its cancer maybe it is another disease. You did not ask

*R: On that I was just confused [laughed]*

I: Ok so that day when you were being screened right, everything that happened you got here whether they welcomed you, they told you maybe sit here and screened you right? What is it that you felt went well what do you think is it?

*R: Went well when they were screening me?*

I: Yes, what you feel aah this thing went very well, and did well to me?

*R: Being screened it’s I feel it was good since it is what am saying that I knew how I am.*

I: And what you feel they would have done better what is it? What you feel this did not went well right, that maybe they would have done as this, or they would have done a certain way what is it?

*R: On that eeh, it will be difficult*.

I: Everything was fine? Getting here being welcomed form the beginning until the end?

*R: Yes aah they welcomed us very well, since they were explaining to us at first, they explained to us how the disease start what we can do everything they explained to us very well. Then we went for screened there was no problem.*

I: Mmhmm, ok so to you they did not tell you what they found, they just tell that the body tissue has been found with a disease and they just told you to come again at a certain date. So When you got here, they gave you an injection they did not tell you anything you say. They just told you to come again at a certain date right. Then you came again all that to you how do you see it?

*R: I feel like maybe am the big problem right, because I did not ask, maybe they explained and I did not get them maybe I forgot but when I came here its when they told me that the body tissue that they took has been found with an infection so in my thought I was just thinking that oh, it’s the cancer the disease that they are talking about?*

I: Mmhmm, they did not say what kind of disease they just said we have found with a disease?

*R: Yes*

I: Ok and the easiest part what was it, here we have talked about what did not went well and the part that was easy on the whole process that you went through to you the easiest part what was it?

*R: From where they screened us or*

I: Mmhmm from when you reached here they have screened you and you done all and you have left the part that you felt this one only was easy, which part was it? From when you arrived from being screened?

*R: Like maybe when they finished screening me right since it was the first time I have never done that they insert metal instruments or they do what so when we were done when they were giving me a form to come with it here, there its when I understood very well, since they explained again very well, but there at first I was afraid when they were inserting the metals and the like, that aren’t they going to hurt me?*

I: Mmhmm, so the fear was there that isn’t that going to hurt me but still you accepted them to insert it?

*R: Yes*

I: Why, what encouraged you?

*R: I wanted to know [laughed]*

I: Mmhmm, and was there anything that you did not expect that happened? The UN expectable?

*R: That I could be found with the disease it is what I was not expecting, since I was expecting that I could be feeling something in my stomach right.*

*R: Yes but I was not feeling any problem, yes but when I went there it’s when they found that I have been found with that problem.*

I: Mmhmm. Ok so it can be hard for other people to also come right, like the way that they told you that now you should come at a certain date right, to other people it can be difficult right?

*R: Other people fear, since other people are afraid*.

I: Mmhmm like the one you what challenges did you have? To come on the follow up visit?

*R: So when they screened me when I started discharging the smell vaginal discharge, it is what scared me, at first I felt like maybe it will happen for a few days but it was still going on. Yes so they were like hot, so that scared me that iih am supposed to go again and see what is it?*

I: You felt it could be what when the discharge was coming out what was going through your mind?

*R: I was like is it an infection? How I felt that is it an infection or something is destroyed in my stomach when they were doing with that thing, it is what I was thinking?*

I: Did you explain to anyone, that since I got screened this is happening

*R: No*

I: Why?

*R: Since people were refusing right like my neighbors, they were refusing.*

I: To be screened?

*R: Yes, so I just felt that there is no reason to tell them it means they would have been telling everyone now, everything so I just left it.*

I: ok but when they screened you, they have told you, and they have told you to come here right? On a certain date, what challenges did you have in preparing for that visit, were there any challenges? They have screened you and you are done you have gone home they have told you come at such date right, so preparing for that visit?

*R: Transport was making me not to have peace of mind, transport. However, the others are what am saying that I felt pain that time right, later then it stopped, so even when the discharge was coming out I saw that after 2weeks they stopped.*

I: The discharge now?

*R: Yes and I started menstruation normally*

I: Mmhmm, and to other women who went to be screened that day right, and were told to come at a certain day, what do you think can be barriers for them to come again?

*R: Some fear, some not accepting, some maybe just being laid back, some transport*.

I: Mmhmm those with fear lets start with those with fear; what can they be afraid of?

*R: I don’t know their problems. There was this other friend of mine she is the one who knows very well because for me to know that we should come here, my friend was the one who came to tell me, I was coming with her here she is the one who told me that they called they say we should go to the clinic but still other people hid themselves, they were missing they were not know who they are.*

I: The other people who were screened that day?

R: *Yes*

I: Ok, hiding themselves as all of you were screened and the phone call came from one person.

*R: They came those people in our area and they were looking for people so I think they did not find the other people, because they was something happening I think there was a funeral the day they came, so then her its when she recognized me and a certain lady its when they came to tell us then its when we came here, so other people maybe they do not accept quickly*.

I: What can make them not to accept quickly?

*R: Childishness I feel so*.

I: Apart from childishness?

*R: Aaah what I was saying that maybe transport.*

I: Those who are laid back? You think why they are laid back.

*R: I feel like maybe they have not accepted the results that were found with them.*

I: Ok how can we help women to able to come back how can we help on these challenges? So that maybe these challenges should be reduced with the aim that these women should be able to come back here, what can we do?

*R: I feel like if they have the names.*

I: People from here?

*R: Yes people from here if they have names for those who did not come, they can just go to the village headman it means they can know them quickly the who did not come.*

I: If they do not have names?

*R: Aaah I doubt since they were writing names.*

I: Ok they were writing names.

*R: Yes they were writing names*

I: Maybe some gave a fake name.

*R: That is difficult then*

I: Ok all right, so whom did you talk to about your screening? You have said yes that your neighbors were refusing right but to say you have been screened whom did you talk to?

*R: That friend of mine that I came here with.*

I: The other one?

*R: That is all*

I: Like maybe your partner or anyone from your family, or your husband?

*R: I do not have a husband but my sister I was just explaining to them right, the advantage of going for cervical cancer screening, I was just explaining to people like her.*

I: When you explained to them, what did they say?

*R: As my sister was saying, she is scared because people were scaring her that the metal is painful.*

I: And your friend?

*R: The other ones were saying the same thing that the metal is painful and the like, and I was telling them that the metal is not painful it is just ok, there is no any problem.*

I: Or maybe discourage you that why were you going there and the like?

*R: Yes*

I: What were they saying?

*R: They were like why were you going were you doubting yourself? They were saying things like those.*

I: So you have gone and explained to them that I went for screening and you were discharging smelly vagina fluid that time and when they were asking you why you were going there how did you feel? You have already gone there and you are explaining to them, and they are asking you why were you going? How did you feel? Looking at, that time you were discharging smelly vaginal discharge.

*R: What scared me is what was coming out, I was scared but what people were saying I did not mind because I knew that everyone has her own choice right, so those ones it was their choice not to go but still I was afraid because of the discharge that why but somehow I was strong because they told us that you will be producing smelly vaginal discharge, they told us so.*

I: When you were telling like your sister was it seeking permission or just explaining to them or it was like just a chat? What happened for you to explain to her?

*R: I wanted to tell them right, that if they also want they should go for screening.*

I: Ok. So when they told you the results did you tell them again? That the body tissue that they took they say it has an infection did you tell them?

*R: I did not tell them.*

I: Why?

*R: Aaah she is too talkative I knew that it means she will be telling everyone.*

I: Ok, so you did not want her to tell everyone?

*R: Aaah no*

I: Ok, so apart from burning service we are advising you not to have sex for a month to give room for healing was this difficult for you?

*R: No There wasn’t any other problem*

I: There wasn’t a problem?

*R: The goodness to me right I do not have a husband so I did not see it as something to worry about, it is different with having a husband in the house, for you to tell him that other men do not understand but tom there was not any problem.*

I: Do you think men can take a big role in a woman`s cervical cancer screening? Can men take part?

*R: Yes*

I: In what way?

*R: Mostly like marriage right since they have to understand what caused for someone to be found that she has cancer right, so they are supposed to give permission to the lady to go like that friend of mine that I come here with she is married she has a husband, we were leaving together her husband was the one who was reminding us sometimes start off to the clinic, meaning that he took part it concerned him that his wife has been found like that.*

I: So you think like for that one right they were reminding you right, what is the other advantage that a man has taken part in the cervical cancer screening?

*R: We can take care of our life*

I: Ok can you explain, the husband has taken part what is the advantage, can you explain?

*R: Because like when they were explaining to us they said the other problem that causes it is like when the husband has several sexual partners, it is what makes that he brings an infection in the house so if he is to take part it can help that the infection should be reduced now.*

I: Ok all right. So for us to encourage them to be coming right, what can we do?

*R: Maybe visiting the people in the village,*

I: when we have visited them?

*R: Help them enlighten them the goodness of cervical cancer screening.*

I: So to women you are going to explain to her that cervical cancer is like this and that, to men how can we explain to them about cervical cancer how can we sensitize them?

*R: I feel like there is no problem explaining to him the way you can explain to a woman.*

I: Why isn’t there any problem, because they are two different people right, one is a woman one is a man why don’t you see any problem?

*R: But for people like marriage it is easy because he is supposed to understand what the cancer is caused of, how does it come to infect a person? Therefore, it is easy to explain like the way you can explain to a woman.*

I: Is there anything new that you have learned concerning cervical cancer or cervical cancer screening that you did not know before the study was conducted? Anything new that you have discovered because of the study, about cervical cancer?

*R: Because I did not know that when conducting cervical cancer screening what method do they use, so I knew how they do it, but also I knew the methods that they use to deal with the disease to end or prevented.*

I: What else? What new thing that you learned? Or else now you know right that this is how they do the cancer screening, that it does not have to spread they do this right, how do you think it is going to help you? Now that you know right, how is it going to help you? Alternatively, the benefit to you now that you know what you think it is going to be.

*R: The benefit to me is that since I did not know and now I know and I have been found with it that means I know that my life now is like this, because if I have come here at the clinic, it where they know and it is where they are able to end that problem or seeing that we will not be able to deal with this problem, right? So the advantage is I have known and I know which way to follow.*

I: Mmhmm, so you think who is supposed to undergo cervical cancer screening? Which women that are supposed to under go, is it childbearing women, married women, HIV positive women, who? Which group of women is supposed to be screened for cervical cancer?

*R: I feel like whether child bearing women or women who are infected or women who are married, those ones, I don’t know on the part of women that if they are able to do the screening to women who are not married or they do not have babies yet, but women like us who have given birth, who were once married but also women who are infected those are the women are supposed to be screened.*

I: But these girls do you think they are also supposed to be screened or thought you do not know if they can have the cancer or not, just thinking do you think they are supposed to be screened or not?

*R: But I feel like it is good if they are to be screened as well.*

I: Why?

*R: Since nowadays, better in the past but now most women they are sleeping with men so they say sometimes women get infected from men, so they are supposed to be screened they should know how they are their cervix.*

I: Ok all right, so how often is screening supposed to be done? Like maybe after how long, who is supposed to be screened and how frequent, maybe like you when were you screened?

*R: It was in august*

I: August, so lets say it was in august right, you think the screening should be done maybe after how long? Like from august how long should it take?

*R: I feel like maybe every year right.*

I: Every year should they screen each woman or only those who were found with the problem at first or the ones who were not screened the first time who should be screened every year?

*R: The first time since like this problem is found right, she will come at the clinic and you will see them yourself right that this problem has ended or she is supposed to continue, that means you must start other people who have never screened.*

I: You think what can be the advantage.

*R: That means the infection can be reduced.*

I: For it to be reduced is it because the screening is being conducted now and then or what do you think can be the reason?

*R: Since when you have screened them right, you give advice so the for someone who listens to that advice, it means it can make the disease to be reduced.*

I: Ok, so women in your area what do them think about cervical cancer screening? The neighbors that mentioned what are their thoughts on cervical cancer screening.

*R: Some think that when they say they have cancer it means they have an infection like AIDS their thoughts right some they think of many things.*

I: But do you think they really know what cervical cancer is? Alternatively, what is the cause, that they have that knowledge? Like that kind of knowledge, do they have it? On the other hand, not, on how you see it?

*R: They don’t have*

I: That they completely do not have.

*R: Aah, I do not understand what the problem is.*

I: Do you think women in your area understand the goodness of cervical cancer screening?

*R: To those who accepted it they understand the goodness.*

I: For you to understand was it because you have gone for screening or?

*R: I heard about it at first right, what I have already said that a certain friend of mine came at the clinic, so I heard about it even on the radio they were announcing that women should be going for cervical cancer screening.*

I: So we can assume that other women also heard about it right.

*R: A lot yes*

I: Maybe the goodness of being screened do you think they know it or not, the goodness of being screened for cervical cancer.

*R: People know the goodness but it is just that they do not want.*

I: Ok, so in your thoughts do you think women have the interest to be screened for cervical cancer and get treatment?

*R: Yes*

I: What has made you to say yes?

*R: Because like other people accept to be coming here right and get treatment, which means that, other have the interest.*

I: We can assume that how many women who have interest?

*R: Mmhmm, because I don’t know how people come here right*

I: Just assuming

*R: But still it is many people who say that right that we went for cervical cancer screening like how they were there, there were many people who went for cancer screening*

I: Comparing to those who did not go? Who were a lot?

*R: The ones who went were a lot*

I: Ok, what do you think can be the reason why a person cannot want to be screened? You have talked about fear you have talked about childishness, but what else can be the reason why people can refuse to be screened? Maybe like being afraid of what other people will say what you think can make them.

*R: Mmhmm, that is difficult*

I: It is difficult.

*R: Yes thats what exactly makes them to refuse?*

I: But just assuming right for a woman who is married and another one who is not married, who do you think can make a decision easily to be screened?

*R: Those who are married*

I: Why is she different for the one who is not married what`s the difference?

*R: Because those who are married right, anytime they can make a decision to have a baby right, so they are supposed to know that is their cervix ok?*

I: Mmhmm

*R: Yes*

I: What barriers can women face when receiving this treatment? Barriers and challenges?

*R: From the clinic?*

I: Whether from the clinic or right there at home

*R: Maybe some their husbands right they maybe stop them, some men do not accept right yes so maybe that cab stop them.*

I: Stop them to come at the clinic or to be screened?

*R: Yes not to be screened*

I: Mmhmm, others?

*R: It is what I said that maybe just being laid back.*

I: So in your thoughts the support of cervical cancer screening how is it supposed to be initiated so that many women should be able to be screened. In your thought?

*R: It is what I have said that, going out in the villages*

I: Like how they did with you?

*R: Yes because maybe just telling people that you should be going to the clinic or what maybe it’s a few who can come here maybe going out in the villages*

I: What can make them fail to come here? Like maybe the others?

*R: Like maybe others transport, maybe some the distance right,*

I: But still when they go to the villages right, some are still refusing like the ones you were giving examples right, so like that group what do you think we can do? As the neighbors that were running away, what do you think, we can do for them to still come.

*R: Maybe what I have said that maybe going like in the villages and go like at the village headman maybe like that*

I: The village headman like to.

*R: Sensitizing them, since you will go with them right so maybe there are other people whom they are refusing but you know their names, maybe if they go through the village headman and tell him that we are looking for these people to come to the clinic*

I: Those that were screened?

*R: Yes*

I: So for those who completely refused what can we do so that they are able to come? Those who completely refused, those ones it is better they went they just refused to come to the clinic right.

*R: Firstly maybe you should be coming in an area right and conduct a sensitization meeting, just calling people and explain to all of them there and one for oneself and her decision, that means since you will be* *giving out appointment dates that we will come at such date to do, that means there because of people understanding, there then its when they will be able to go rather than, just being told by their friends, it is difficult.*

I: Ok the other challenge does not understand.

*R: Yes not understanding*

I: But like you how can you encourage them to go and be screened? That means we will hold the meeting right but like you how can you encourage them.

*R: I can be telling them the advantage of being screened for cervical cancer.*

I: The advantage you can say is. Let`s say you are explaining to someone what advantage are you going to tell that person? You have talked about the metal instruments you have talked that you had a backache, what advantage can you tell a person?

*R: Its good to tell her that truth she should know right, people know the truth that to get screened they are supposed to insert metal instruments to see how that person is right so explaining to them it is good they should know that, how their cervix is rather than just staying, and maybe most women complain about pain in the stomach and the like not knowing what is causing that, so explaining to them things like that, some people will understand and go.*

I: All right, so now we are going to talk about collecting the vaginal swab with cotton wool by yourself for cervical cancer screening right. You know the method that you went through of inserting metal instruments down there right. So the new method to conduct cervical cancer, it is about the woman self-collecting vaginal swab and submit at the clinic so that they screen at their convenience right, have you understood?

*R: Yes*

I: That you will collect vaginal swab and submit it at the clinic or any other health facility so that they screen for cervical cancer in another method right? So the difference with this self collecting of specimen is that the woman will not be getting results at the same time and she is supposed to come again at the clinic to get her result after a few hours or the next day right, so that means now she will be told to wait for a few hours or they will tell her to come the next day right, so what are you thinking on this method? That the woman should be self-collecting the vaginal swab and submit to the clinic for cervical cancer screening? What do you think?

*R: I feel like it is very different with how they did that right, because this she will be collecting herself and bring it here like today, and tomorrow she is supposed to come again so the transportation there will be very difficult, since and with time also right since lets just say this time in the village it’s the farming season so for come to come today and tomorrow she is supposed to come again, that means maybe when you have given her the results you will give her an appointment date shat should come again, that I feel like that will be a challenge to people comparing to the other method.*

I: Ok all right, apart from that what else like your other thoughts?

*R: I feel like some will not even do it they will just leave it and not come back.*

I: Just leaving it like when they have collected the vaginal swab or before they have collected the vaginal swab?

*R: Before they collect the vaginal swab just throwing away the cotton wool and not coming back.*

I: Why do you think can be the reason?

*R: I feel like some will not take as useful, some just as I said that just being laid back right, rather than just screening and see the results right there*

I: Can you be interested in this method? The method of self-collection of vaginal swab? Can you be interested?

*R: But with time, it can be difficult.*

I: Mmhmm so to you which one can you like?

*R: The one that they did last time.*

I: Ok you have chosen that method but the advantage of this other one what can it be? The advantage of the method that I have told you?

*R: This one is good because since they do not insert metal instruments right, that like how people were afraid that they feel pain and the like it is good that you can do it yourself and submit, I feel like it is easy.*

I: Another advantage? Or else what is the disadvantage of this method? You have said other people will just be throwing away the cotton wool the other disadvantage of this method. Alternatively, do you think it is reliable?

*R: Aah not that much,*

I: Why?

*R: It is good but not reliable because of what I have said that you will give a person to do it at home then she should bring back it is different with giving her here and she has to do it here or people have come in the village you will do it then you will take it, then you will call her to come that is better, not that one should come here and do it or she should bring tomorrow then she should come again, on that I feel its difficult.*

I: So you feel like its better she comes today and give her the cotton wool whilst she is here she should do it and leave it but you can call her to come the next day there is no problem?

*R: Yes*

I: As that rather than she should do, it at home and she should come back.

*R: Yes*

I: Ok so how can you compare the self-collecting method and the other method how can you differentiate? We are comparing this method of doing it yourself and that other one. I know you have chosen the other one but then why are not happy with this one, why?

*R: That’s what I have said that the way to do it can be difficult rather than that it should happen while you are there, but that someone should do it at home and then bring it, that is then difficult but the method itself this one is easy than the method of inserting metal instruments, it is but for someone to do it at home then bring it then she should come again, that is difficult but lets say we are doing it right here using the cotton wool right here there is no problem there?*

I: There is no problem

*R: Yes, there is no problem*

I: Ok all right, and what do you think other women in your area can think of the method of self-collection of vaginal swab for cervical cancer screening? Other women? Which method can they like the self-collection or using the metal instruments?

*R: To other people who are afraid of the metal instruments method they can choose that method.*

I: You think they can be how many who can choose this method than the other method? Who can be many?

*R: Because of fear, the other ones can be many*

I: What challenges can women face on this method of self- collection?

*R: The time they are collecting. I feel like there is not much of a challenge.*

I: And submitting the specimen for screening?

*R: The transportation now*

I: Apart from transportation? Embarrassment, fear?

*R: Embarrassment yes*

I: Why embarrassment or fear?

*R: Since when you are doing this you wait for two things that they will find me infected or not.*

I: Ok. So the fear is that maybe they will find it or not?

*R: Yes*

I: Not fear with the method?

*R: No*

I: And are other reasons that you think women cannot want to be self-collecting the specimen, why would not they want, the other reasons?

*R: Aah, other reasons? Maybe just their thoughts I do not what they would be thinking the other people, but aah, it is difficult.*

I: Ok so like you, you have said you can choose the metal one right.

*R: Mmhmm*

I: So to other women also why do you think they can choose to be screened by a health worker than to do it himself or herself? Why can they choose the other one than this, other women?

*R: Since they know that this one when its done its like everything have been done and everything has come out like results right, while this its like you do it and bring it do it then come with it here and come to get the results the other day so this other one its like they have done it and they have found the results right there.*

I: So let us say one has done it and one has followed the other methods both of them have been given the results right, which results can be reliable or trustworthy which one can it be.

*R: The metal one,*

I: The metal one?

*R: Mmhmm*

I: Why?

*R: My thinking I feel like it is as if they are seeing everything and they screen everything according to how the cervix is like right, whilst this way they will just screen the vaginal swab only maybe they cannot see anything else that is my thinking now.*

I: Ok so let us talk about your recommendations in the future of the National cervical cancer screening in Malawi. In your opinion, should the Ministry of Health consider including self- collection of vaginal swab for cervical cancer testing to the cervical cancer-screening program?

*R: Both of them?*

I: Yes now the one that is in operation is the one that you did right, but do you think this one should it considered and included in the Ministry of Health?

*R: They should just try in other people to see is it going to helpful.*

I: They should just try.

*R: When they see that, it is all well that means they can go ahead with it or both of the methods, whether they are going to choose both or one.*

I: Why are you saying they should include them right away?

*R: Since it is what I have said that when they insert the metal instruments I do not know if the use the cotton wool and collect the vaginal swab are they able to screen all the infections or just cancer from the vaginal swab? Nevertheless, if it is a method that they screen everything then there is no any other problem.*

I: Ok, and do you think it can be easy for women to be screened lets say they have tried the other method and they have established it do you think it can be easy for women to be going for cervical cancer screening?

*R: Yes*

I: Why do you think it can be easy?

*R: Pardon?*

I: Why do you think it can be easy?

*R: Because maybe to others it can be easy because other people are embarrassed it is different with the time you are delivering a baby that time you don’t think of anything you think of iih as long as am assisted but this time that you are normal maybe to be naked and the like as we were doing some people are embarrassed, maybe other people can choose that method by seeing that they will do it their selves.*

I: Ok so which groups of women could be most suitable for self-collected vaginal swab for cervical cancer testing? Which groups do you think?

*R: Including the girls?*

I: Yes maybe the girls, we also talked about women maybe who are child bearing, some married, some HIV positive which group do you think is the most suitable for self- collected vaginal swab for cervical cancer screening?

*R: Maybe the girls mostly right, the ones who are not yet married and they do not know what we do.*

I: Like doing what?

*R: Maybe being naked to be screened right, maybe they can be embarrassed most girls to be naked and be seen by someone*

I: Mmhmm, so the girls are the most suitable for this method?

*R: Yes*

I: Ok and which groups of women cannot be suitable, the ones you feel that these ones not this method, which groups?

*R: Mmhmm, on that no*

I: Ok, so this is the end of our discussion but if you have anything to add, you can tell me now.

*R: But I think the most part is what I have said that maybe the people right they should be going out in the village and sensitizing people with the aim that other people should be reached because like the time they went there right, some people were knowing about it late, that people came so for them to come here like at the clinic it is difficult but if you go and give them that message not necessarily just telling them but telling them in a way of sensitizing them that there are people who are coming from the clinic who want to talk to you explaining to them later then give them an appointment date that we will come at such date that we will come and we will do this this.*

I: So you think that more people will be screened?

*R: Yes*

I: Why?

*R: Since people will understand now right rather than just explaining to each other people do not understand they think wrongly but if you explain yourself that means they choose themselves that this is a good way.*

I: Anything to add?

*R: But I would like to ask that is it possible if they have found someone with cervical cancer and is undergoing treatment is it possible that it ends like for good?*

I: Any other question?

*R: Answer that question first*

I: No, like how I am I cannot be able to answer that question I will lie but I will send you to someone who can answer that since everyone has a role right, I will lie.

*R: Alright*

I: But there is another question if I fail that I will also refer you to other people.

*R: No not right now.*

I: Ok, so thank for your time right

*R: Thanks a lot.*

I: Thanks.
