# Supplementary material for: Perceptions of cervical cancer and motivation for screening among women in Rural Lilongwe, Malawi: A qualitative study
Source: PLoS One. 2022 Feb 7;17(2):e0262590. doi: 10.1371/journal.pone.0262590 (PMC8820632; doi:10.1371/journal.pone.0262590)
Supplement: S3 File — (ZIP) [file pone.0262590.s003.zip › VIA 385 Missed.docx]

**PARTICIPANT ID:VIA 385**

**Date of interview: 09 Dec 2017**

**Interviewer ID: 466**

**Length: 45 minutes**

**Transcriber ID: 466**

1. I: Thank you for meeting with me today, and for allowing us to have this discussion in your house.
2. *R: Thank you very much.*
3. I: I really appreciate your time and your input will be very helpful. I am working with a team of researchers from the University of North Carolina Project in Malawi.

Sorry you were not able to come for your 12-week follow-up appointment. We would still like to hear from you regarding your experience with the cervical cancer screening with VIA and thermo-coagulation treatment. We would also like to hear about any difficulties you had afterwards or any challenges you had to coming for your follow-up visit. Your input is also important to help us understand how best we can conduct cervical cancer screening campaigns in Malawi. There is no right or wrong answer. Everything you say will be confidential and only used to make this health program and health questionnaire better. As I said, I will audio record this interview to help me remember what was said, but your name or any identifiable information will not be connected to anything you say. Do you understand?

1. *R: Yes.*
2. I: Alright, thank you. first of all, can you tell me your understanding of the cervical cancer screening and treatment you received?
3. *R: What I can say is that screening for cervical cancer is good because you know if you have it or not. Just as they taught us, they said that when you have cervical cancer, you don’t experience any problems, you spend your day to day life normally and he person only realizes it after it is too late. They said that was why they were conducting the research, so that they can screen women. I think it is good because from the screening, you were able to know whether you have the cervical cancer or not, that is what I can say.*
4. I: Alright, so in your case, how were you screened?
5. *R: I was screened and in the end, they told me to come to central hospital. they also told me not to have sex with my husband for 6 weeks, up until I get my final results at central hospital. They told me to go there [at central hospital] I think on 4th (Month) and I went there with my husband. When we got there, they welcomed us very well. Because I am a prayerful person, when they told me to come to central hospital, I knew that I had been found with the cervical cancer, but what they told me was that I will get my results when I come to central hospital. So I put it in my prayers and I prayed for it. When I left for the hospital on 4th (Month), they told me that the doctor had left my results and they showed that I did not have cancer, but it was another disease. They told me that they will inject me and then give me some oral medicine and they said I should come back 2 (month). It is on the 2nd of (Month) that I did not come to my scheduled appointment and from what I saw, I said to myself that what I had asked God to do for me was fulfilled and I was fine. So I thanked Him for His faithfulness and also because I had seen His greatness. I was worried before, but they told me that what they had found was not cancer, it was another disease. Although they did not tell me what the other diseases was, they just gave me an injection and tablets and told me that I should come back so that they can see if the other disease is cured or not. That is why I took it lightly, I asked of God and He responded and I was found without cancer, so I did not see why I had to go back [chuckles].*
6. I: Alright, the day you got screened, what kind of screening did you undergo?
7. *R: when they came, they had their own equipment which they were inserting in the vagina. From there, they were taking vinegar and applying it on the thing they inserted in the vagina. I didn’t understand how they were able to tell if the person had cervical cancer or not, but they had their own methods. When I was being screened, it was a female doctor doing it, but then she later called 2 other male doctors who also wore gloves. When they had examined me, they told me that I should not have sex with my husband for 6 weeks, up until I come to central hospital and they examine me again.*
8. I: Okay, what results did they give you, they told you not to have sex until you come to the hospital, but what result did they give you?
9. *R: After the screening, they told me that “it seems we have found you with a problem”. That is when they gave me back my health passport book and I came back home. But as I have said, I am a very prayerful person and so I prayed. I told Him that there is no disease that you have failed to cure and there is nothing that has ever overcome you, so I want to see your greatness. I told him that when I go to the hospital, I want to receive favor, they should welcome me very well, and that is exactly what happened.*
10. I: Okay, you have told me of the equipment which they inserted in the vagina and the vinegar and that the first nurse called 2 other nurses; what else happened on that day?
11. *R: Umm, we also got tested for HIV, although that was not the first time I got tested, but after I showed them my previous test results, they told me that I still needed to get tested again. From there we went for cancer screening. They also asked us the importance of the screening which was taking place. They told us that a lot of women are dying of cancer and that is why the government came up with the screening from the community method so that if the person is found with cancer, thermo-coagulation can be done and the cancer cells can die.*
12. I: Okay, was thermo-coagulation done on you?
13. R: Aaa, maybe they did, maybe what they were doing was thermo-coagulation. They were inserting things inside my vagina, so maybe they did that.
14. I: Okay, but you are not sure if they did it or not?
15. *R: I don’t know. They told me that “you will start producing a lot of vaginal discharge”, and that really happened. However, all I see is the greatness of God.*
16. I: Alright, thank you. I would like to hear your thoughts on cervical cancer screening, just like the one which you took part in.
17. *R: Okay.*
18. I: Why did you choose to get screened or to take part in this study?
19. *R: Alright, all along, I wanted this cancer clinic, I really wanted it. There was a certain time when I was producing a lot of vaginal discharge and it was yellow in color. I used to think that maybe because my husband was being promiscuous at that time, maybe it was sexually transmitted diseases. I like to listen to the radio and on this day I was listening to a doctor talking about cancer and he said that sometimes the sign of cervical cancer could be abnormal menstruation, sometimes a sign could be smelly vagina discharge. I used to listen and think that maybe from the signs this doctor is explaining, maybe I have cancer. I was always looking for a place where I could get screened so that I know if it really is because of cervical cancer that I was producing that discharge. Then, I heard that somewhere (Name of area), at a hospital run by white people they test for cancer. Another friend told me that they also test for cancer at (Name of hospital) just that there is usually a lot of people. So when the people for screening came, it was actually a friend of mine who came and told me that the cancer clinic I wanted is being done at (name of area) then I went there with that friend. At this point, having received my final results that I don’t have cervical cancer, I can only praise God. I think such clinics are important because cancer is just like HIV, you need to get tested for you to know your status. It is always difficult when you get tested at a late stage, but when you get tested in the early stages, it is better and you even receive counsel.*
20. I: Alright, so you have said that you heard on the radio that one sign is yellow vaginal discharge, and you also experienced that, not so?
21. *R: Yes.*
22. I: So before the screening happened, what worries did you have?
23. R: I was worried. I used to say that ‘maybe because of what I am experiencing, it could be cancer’ and so we even asked the doctors several questions on that day [chuckles]. They told us that what they aim at is to do the screening and the results are what will show whether there is cancer or not. When they told us that we will get the final result when we go to central, I thought that maybe with the vaginal discharge I was having, it could be cancer. That is when I prayed for it before I went to central for my results, and the results I received there showed that I did not have cervical cancer. Since that time, since the injection they gave me, I don’t have any problems.
24. I: Even the vaginal discharge?
25. *R: Yes! It now comes out normally, the way every woman produces it and there is no problem [chuckles].*
26. I: Alright, thank you very much. You have said that you thought of going to a certain hospital so that you should get screened?
27. *R: Yes.*
28. I: Meaning you had heard of cervical cancer screening before?
29. *R: Yes, I heard it on the radio. The messages were there that ‘go and get screened for cervical cancer, know your status’. The messages were there, but because our hospital is just so small, they don’t have the equipment to perform such tests. So we kept searching for a place where we could get screened and that is when some people were saying that (Name of hospital) offers the services and the other clinic as well. When we heard that the service has come close to us, we* just praised God because he had made a way for people to know their status.
30. I: Alright, you saw it as a good thing but there could be others who were saying different things about the ring as well; what misconceptions were there about the screening?
31. *R: Aaa, I should not lie, I did not hear any misconceptions concerning the screening, that is from the group we went to have the screening with. We were all just grateful that the service we needed has been provided to us and we will know our status. The friend I went with was told that she is fine, I was the last one and they told me that I should go to central to get the results. After I went to central they told me that I don’t have cancer, instead it is another disease, I just praised God for that.*
32. I: Alright, how did you feel when they told you that it seems you have cancer, but you should come to central hospital on 4th (Month) to get your final result?
33. *R: Because I had put it in the hands of God, I just accepted it. I knew that there is no one who can deal with that problem except God. I know that God created educated people like you to do this work so that we can assist each other while here on earth, but the all-knowing one still remains God. So I accepted it. I was worried yes, but after I prayed about it, the worries were gone and I accepted it. Just like with HIV testing, after the get the blood sample, they ask us that “are you ready to hear your results”? and we respond to say yes, we are ready. At that point we have already received all the counsel and we are ready to hear and accept whatever we are told, whether we are found with the virus or not, so I just accepted it.*
34. I: The worry that you have said was there before you prayed about it, what worry was it?
35. *R: The worry [laughs].*
36. I: Yes, the worry [chuckles].
37. *R: The worry that was there, I was saying that what if my cervix is completely damaged and they have to remove it? But in the end I just said that God is good, he knows what to do. Afterwards, the worry was gone [chuckles] but yes, I was worried that my cervix might have to be removed [laughs].*
38. I: Alright, after you got there, what do you think went well?
39. *R: We went there to get assistance, and they told us that they want to reduce deaths of women from cancer, so that we can have a healthy Malawi. So I think that the government, by coming up with this research with the aim of helping women get assistance, I think that is what went well. Because like I have said, when you get assistance after things have already become worse, it is often difficult to get better, that is also why they say prevention is better than cure.*
40. I: Alright, what do you think could have been done better?
41. *R: Aa no, I think everything was fine.*
42. I: Everything?
43. R: [Chuckles] Yes, it was my first time of course, but I feel everything went well.
44. I: Alright, from the time you arrived until the time you left for home, you have talked of the conversation you had with the staff where you were asking questions, the HIV testing and then the screening itself; from all that happened, what do you think was the easiest part?
45. *R: I can say everything was all the same.*
46. I: Or tell me what you think was the hardest part of it all?
47. *R: The part I saw was hard, especially for the person who has been found with the cancer cells, they were taking longer to come out of the screening room unlike the one who has not been found with any cancer cells.*
48. I: Okay, so you were part of those who took long?
49. *R: Yes.*
50. I: Okay, so what are your thoughts on the time it was taking?
51. *R: The time was okay just that there is a lot of work that has to be done in there. They also do thermos-coagulation if they have found cancer cells and part is painful, you feel pain. There was a certain woman who went in before me. After she got out he was explaining to her friend that they have removed something from her cervix [chuckles] and the fried was asking what they have removed. After I went in, I realized that she was talking of the machines that they were using [chuckles].*
52. I: [Chuckles] alright, so that was before you went in, and you are hearing someone telling their friend that they have removed something from her cervix, how did that make you feel?
53. *R: [Laughs] Eeh, you know what, when you are the one who needs to be assisted, you don’t get scared. It is the same with the things of God, they say that only the brave shall see the kingdom of God and even at labor, we all know there is nothing easy in there but there is no way you run away. Everything just requires you to be brave if you want to be assisted.*
54. I: Alright, is there anything unexpected that happened?
55. *R: Aa no, they told us everything that was going to happen before hand, so we knew that its either we will be found with cancer or not. They also told us that if you have been found with the cancer cells, they have equipment which they use for thermos-coagulation, they explained everything beforehand.*
56. I: Alright, thank you very much. It could be hard for women to come for their follow up visits, can you tell me why you were unable to come for your follow up visit?
57. *R: What I had asked God to do for me was done and the other disease for which they gave me an injection, there was no problem I was experiencing with it. So I just thanked God that he assisted me, and that was it. I don’t have any problems until now.*
58. I: Alright, what are the reasons why you think other women would be unable to come for their follow up visit?
59. *R: Aaa, I wouldn’t know why other women would fail to go for their follow up visits, the woman is the one who can be in a better position to explain why she failed to go. Like I am explaining that I had received the help I needed and they told me to go back so that they should see if the other disease they had found me with was cured. After I saw that I wasn’t experiencing any other problem, I just thought it wise to believe in God and to praise Him.*
60. I: Alright, but then just thinking of it, I know it is hard for you to know, but just thinking of it, why do you think other women would fail to come for their follow up visit?
61. R: Thinking is also a bit hard because you could think that this is the reason and yet that is not the reason. Besides, the women who went for the screening came from different areas and on the day I went, I think it is only me and another woman who stays at (Name of area) who were found with the problem. So it is really hard to know.
62. I: Alright, but then assuming there are others who failed to go for their follow up visits, I will ask you to think of how best we can help those women so that they can come for their follow up visits and be assisted?
63. *R: What is needed there, just as you have visited me today, you should also do the same for those who did not come. You have the names of everyone who was supposed to come but did not come, so I think it is better if you visited them to find out what made them not to come for their follow up visit, and they will explain why. If it is transport which is the problem, then you can assure them that you will provide the transport once they come; you just need to visit them to find out what the problem is.*
64. I: Alright, what else can we do to help?
65. *R: Mainly it is just hearing from them what their problem is. if it is transport then you will provide it or you can tell her to borrow from someone and you will refund after she has come to the clinic and she has been assisted.*
66. I: Alright, who else did you talk to about the screening besides maybe the friend you went with?
67. *R: As I have said, for anyone to act, it is based on the need they feel they have to be assisted. There are some people who went to get screened and there are others who were reluctant and they did not go, because they felt they did not need any assistance. Only a person who is sick seeks a doctor, one who is not sick does not have any need for a doctor, so even if you explain to someone about the screening and what it is all about, if they don’t feel the need to get screened, there is no way they will go there. But, if they feel that this service is good and it is a service that they needed, they will definitely go.*
68. I: Alright, meaning you talked about the screening with other people?
69. R: Yes.
70. I: Which people did you talk to?
71. *R: I talk to some of my relatives about it but since they said that the will also conduct the screening at (Name of area) from (Name of area), they waited until it came to their area. I remember some people from that area went to get screened at (Name of area) but they were told that they should wait until the screening is happening in their area. Until now however, the screening has not happened in that area.*
72. I: Alright, is there anyone else besides the relatives?
73. R: Aa, for the women in my community, some of them had already gone for the screening, so I did not talk to anyone else, I actually heard it from them as well that there is screening being done at (Name of area).
74. I: Alright, for those who were reluctant to go and get screened, what were the reasons?
75. *R: Aa, they are the ones who know the reasons.*
76. I: They never talked about it like I will not go there because of this and this?
77. *R: No, like I said, I had always wanted to get screened and I was searching for where I could get screened such that when I heard that it was being done at (Name of area), I went and got screened.*
78. I: Alright, does your partner know that you had cervical cancer screening?
79. *R: My husband knows, and when I went to central hospital on 4th (month), we were together.*
80. I: Alright, so what are his thoughts?
81. *R: Aa, I don’t know what he thinks, but after I came back from the screening, I explained to him and he accepted it. 6 weeks passed before we had sex, and then we both left for central hospital.*
82. I: Alright, did he know before you went for the screening or after you had gone for the screening?
83. *R: What happened was that my friend told me in the morning about the screening, she came whilst I was still asleep to tell me that there is cervical cancer screening at (Name of area)and that it had started the day before. After she told me I decided that we should go for the screening, so I went inside the house and told my husband about it, and then he accepted.*
84. I: Okay, if for instance he had said do not go, what would have happened?
85. *R: I would have still gone. It is my life [chuckles]. No one is responsible for looking after my life [laughs]. It’s the same with family planning, the woman might say that I am done with child bearing but the man insists and says I want more children and yet at the end of the day it is the woman who experiences the pain.*
86. I: Alright, do you think your husband is interested in learning more about the screening?
87. *R: Aa, when they were explaining to me he was right there.*
88. I: After you went to central hospital?
89. *R: Yes, we went in the room together and he heard whatever the doctor was explaining to me [chuckles].*
90. I: S do you think he was interested in learning more?
91. *R: Aa, he accepted it.*
92. I: Alright, after you came back from the screening, did you tell him your results?
93. *R: Yes, I explained to him. I told him that I was tested for HIV and I was found negative and from there I was screened for cervical cancer and it seems I have it. But, I told him that the final results will be known on 4th September after we go to central hospital.*
94. I: Okay, how did he react to that?
95. *R: On that day, I could tell that he hasn’t accepted it. But, after I prayed about it, he accepted it the next day.*
96. I: Why do you think he did not accept it on that first day?
97. *R: [Chuckles] You know how men are, maybe he thought that the 6 weeks they had given me was too long [laughs], maybe that is why.*
98. I: Okay, alright what challenge did you experience with that: being told not to have sex for 6 weeks?
99. *R: There was no problem, I was determined to follow the instructions I was given up until I get my final results.*
100. I: Do you think it was a challenge for you husband?
101. *R: He accepted it as well, we even escorted each other to get the final results. Had it been that he had no accepted it, there is no way we would have escorted each other [chuckles].*
102. I: Alright, do you think male partners should be more involved with cervical cancer screening for women?
103. *R: I think they should, it is the same as family planning, even the government is encouraging the men to let their wives use family planning so that by the time they have another child, the first one will be grown enough. Mainly I think the problem is illiteracy or lack of knowledge, especially in the villages. Sometimes you find that the woman gives birth consecutively because the husband refuses her to use family planning. That is why the health personnel nowadays are putting an emphasis on the men because they have a big role to play in the health of their women. It is the same with cervical cancer, the man is supposed to encourage his wife to go for screening. If the woman gets sick, it is the man who will struggle and it is him who will have to spend his money for her to get better.*
104. I: Okay, so what role can they play?
105. *R: Their role is to encourage the women to go to the hospital and get screened for cervical cancer. They can also go together with the wife to the hospital to get screened, that is one thing that is needed. Marriage is made up of two people, so they should both go.*
106. I: Okay, so how can we encourage them to go with their wives for example?
107. *R: They need to be enlightened that cervical cancer is very dangerous and that what they need to do is to encourage their wives to go and get screened for cervical cancer. They need to be reminded that when a child or when the woman is sick, they are the ones who suffer in taking care of them. they end up using money which could have been used for something else in the process of making sure the women gets better. So in order to save their money, they should encourage their wives to go and get screened for cervical cancer.*
108. I: Okay, so when or where is the best place for us to tell them all that you have said?
109. *R: I think it would have been better that when you have come for screening, just as you came last time, you encourage the women to take their husbands as well so that as you are teaching the women about cancer, the men should also hear that message. For them however, you would encourage them to take their wives for cancer screening.*
110. I: Alright, you have said that you had heard about cancer screening before, on the radio, but is there anything new that you learnt about cervical cancer or about cervical cancer screening that you did not know before the study?
111. *R: Yes, at first we were just hearing that there is cervical cancer screening, but we did not know how the screening is done. But after being screened, we learnt how the screening is done. From what I heard the time I was looking for where I can get screened, some people told me that that is a lot of things involved. They told me that there are certain metals which you insert in the vagina and then you take out the whole cervix. After taking it out you apply medicine on it. They used to say scary things [chuckles]. Even though we went for the screening, it was just out of bravery and the need to be assisted. After the screening is when we realized that we were simply scaring each other, there is nothing scary.*
112. I: [Chuckles] Alright, besides that, what other new thing did you learn?
113. *R: Aa, no, I think that is the only thing. The main thing is for you to know if you have it, and if you do, you should get the right assistance do that it should not spread.*
114. I: Alright, who do you think should be screened for cervical cancer, which women?
115. *R: From what we were told, they were saying that someone who is menstruating cannot be screened until they have finished. I think they also said pregnant women cannot be screened, I am not so sure [chuckles].*
116. I: Okay, who else?
117. *R: They said only those who are above 18 years but they should not be above 50 can be screened years old, I think that is what they said.*
118. I: Okay, that is what they said, but what do you think; who should be screened?
119. *R: From what they explained as well, they said cervical cancer is brought about by men if they are sleeping with multiple women, especially those who have not been circumcised. They said the cancer virus hides in the foreskin of the penis and the moment he sleeps with his wife; he transmits it to her. I think there is need to screen because even the girls nowadays cannot be trusted. During our time, when they tell us not to sleep with men we could listen, but with the girls of today, I think there is need to screen them as well. even the older women as well, I have seen an old woman die of cancer, I think she was about 60 years old. She started complaining that her leg was hurting, after some time she started producing vaginal discharge and later she started producing smelly vaginal discharge. By the time they went with her to the hospital, the leg was already rotten and the doctors diagnosed it as cancer, after they scanned her leg. So she died. For a healthy Malawi, I think even the older women should be screened.*
120. I: Okay, how often do you think the screening should be done?
121. *R: [Laughs] that will depend on how you do your work.*
122. I: Okay, but what do you think?
123. *R: [Laughs] I can say that it should be done monthly, but you know better when it should be done.*
124. I: Yes, but what are your thoughts, just tell me how often you think the screening should be done?
125. *R: I thought you test area by area, and then you give the final results and from there you start all over again? So I can make a suggestion and yet you already have a lot of work to do, it all depends on the amount work you have.*
126. I: Okay, but we can plan. In your case for example, you can have chores which need to be done. There can be mopping, cleaning, washing, farming and a lot other, but you plan your time and at the end of the day you find that you have fulfilled all the chores, not so?
127. *R: [Chuckles] Yes, I am able to do everything.*
128. I: It is the same here, we can also plan, all we want is a suggestion.
129. *R: [Chuckles] It can be done monthly; this month you can screen in this area and next month another area.*
130. I: Okay.
131. *R: This is February, so this month you can screen in this area, in (Month) you can go to (Name of health centre) for example and then when you come back here, you will screen those who did not get screened. You tell us to wait for some time before we can get screened again, I have just forgotten the number.*
132. I: Okay.
133. *R: Yes, but at the end of the day everyone should be assisted.*
134. I: Alright, what do women in your community think about cervical cancer screening?
135. *R: Those who got screened or those who did not get screened?*
136. I: Everyone, screened or not screened, women in general, what do they think about cervical cancer screening?
137. *R: Only those who went for the screening know the advantages and the disadvantages of cervical cancer screening, the one who did not get screened does not know anything.*
138. I: Okay, do you think there is any stigma associated with cervical cancer screening?
139. *R: Aa, no, there is no stigma, there is no stigma. Maybe because it is hard to know what your friend is thinking, maybe it is there. It is the same as HIV, when someone has been found with it, instead of encouraging them, you find that others even ridicule them. So I wouldn’t know what other people think.*
140. I: Alright, do you think that women understand the importance of cervical cancer screening?
141. *R: Yes, understanding is there. Especially for someone who had been looking for the service or even the one who has gone for the screening, understanding is there unlike the one who did not go there. They might have heard of the screening, but because they are reluctant to go there, then they wouldn’t really understand.*
142. I: Do you think women are interested in cervical cancer screening and treatment service?
143. R: Yes, they are. The number of women who went at (Name of area) were a lot, and the doctors who were there really had a lot of work to do. I think a lot of women welcomed it because even at (Name of area), the numbers of women who went for the screening were high. Some people say that the cancer first starts with the leg and there are people who have pain in their legs and they tend to be worried. When you have been screened however, you rule our cancer as the cause.
144. I: Alright, why do you think someone would not want to be screened?
145. *R: Aaa [chuckles], like I have said, the one who needs the service is the one who goes to access it. I wouldn’t know why other wouldn’t want to get screened, I don’t know what they think.*
146. I: Okay, but do you think it could be because they are afraid that if they go they will be found with cancer or maybe they are afraid of the treatment?
147. *R: Yes, it is just like HIV, some people still don’t want to get tested. The doctors had to come up with a different way of testing them, if you have gone with a sick child to the hospital, they tell you that before we assist this child, go and get tested. Nowadays they force you to get tested, it is no longer voluntary. That is because some people are still illiterate, they are still worried that they will be found with it if they go and they would rather get sick first. But there is need to get tested so that you should receive treatment ad you should know your status before things get worse.*
148. I: Alright, thank you. What are some of the barriers that women might face in receiving this service?
149. *R: I wouldn’t know; families are also different you know and some men are harsh. As it happened with me for instance, I was told not to have sex until 6 weeks pass. It would be hard for some men to understand that, so maybe that could be a barrier. For understanding men however, those who wish well for their family, they would easily understand.*
150. I: Alright, in your opinion, how should cervical cancer screening be provided to ensure that more women can get screened?
151. *R: As I have said, for someone who does not understand, acceptance is very hard. There is no other way you could do this, you have already come to the villages after you noticed that most women are unable to come to the hospital, maybe due to transport. There is nothing else you can do; this is the only way.*
152. I: Alright, regardless that the screening is being in the villages, some women are still not showing up for the screening, how would you encourage those to go for screening?
153. *R: As I have said, a person who really wants the service can go and access it. But for someone who does not want the service, they cannot go. Even if you encourage them, if they want the service they will not go [laughs]. Even with family planning, a lot of women are scared of it, they refuse to go. Some women turn back after they have already arrived at the hospital, they forget that we need to brave in order to get assisted [laughs].*
154. I: Okay, so there is no way of encouraging them?
155. *R: Yes, if they don’t want to go just leave them [laughs]. Leave them, don’t force them!*
156. I: Alright, let us now talk about self-collected vaginal swab for cervical cancer screening. A new method has been developed for cervical cancer screening. It involves having a woman collect a swab from her vagina and submitting it at her convenience to a health facility for testing. However, unlike VIA, the woman would not get her result immediately and would have to return to health facility to get her result a few hours later or the next day. Have you understood it?
157. *R: I have, the woman will not get her results right away, she will have to wait several hours?*
158. I: Yes, and sometime they might have to go back the next day to get the results.
159. *R: Alright.*
160. I: What do you think about this idea?
161. *R: Mmm, it is hard, I think it is hard.*
162. *I: Okay.*
163. *R: I think it was better when the doctors when coming to the communities. I think they did that after they noticed that some people have trouble coming to the hospital to get screened. But you might have noticed that after they came to the communities, the numbers of women wanting to get screened was high, that was because they did not have to spend any money to get screened. With that method, I think it could take time for women to turn up.*
164. I: Okay, you for instance, would you be interested in this method?
165. *R: I just explained to you that I was looking for this service but I couldn’t find it, that is why I did not hesitate after I heard that it is being at (Name of area)*
166. I: Okay, but what is your interest in this ides I have just explained to you?
167. *R: [Silence] Aa, I should not lie, I am not interested in it because it will be hard for women to go the hospital.*
168. I: Okay, what other challenge could be there?
169. *R: The other challenge would be after the woman has been told to come back for her results; do you think that woman will come back? She will not come back! She will just leave without hearing her results. I don’t think women would accept this ideas, they would not.*
170. I: Okay, tell me any good thing you have seen with this idea?
171. *R: Aa no, I can’t find any good thing.*
172. I: Okay, what do you think is reliable or unreliable with this idea?
173. *R: When they were screening us, they were using vinegar not so? And from what they were taught, the vinegar could show if the woman has cancer or not. Since the vinegar is what they were using to tell if there is a problem or not, should we now say that things have changed?*
174. I: With this idea, the woman will submit her swab and at the hospital, the doctors also have their ways of testing that swab to see if there are cancer cells or not.
175. *R: Okay.*
176. I: Yes, so back to the question, do you think this method is reliable or not?
177. *R: It is not reliable.*
178. I: Why?
179. *R: I think it is not reliable because most women wanted to get screened, but they have difficulties traveling from home to central. They found it was easier to get screened when the screening was being done in the communities; that is the difference.*
180. I: Alright, how do you compare the self-collection and the VIA which you went through?
181. *R: [Chuckles] If they were coming in the communities, it would not be a problem to collect the swab and give it to them. the problem comes in where the woman has to go to the hospital.*
182. I: Alright, how do you think women in your community would think about the self-collecting vaginal swab technique?
183. *R: [Chuckles] It is hard to know what they would think. Maybe some would question why the change in technique, but I think that the VIA method was better off. They were explaining to us that if you have been found with the cancer cells, they will do thermo-coagulation right away.*
184. I: Alright, yes it is hard to know, but which method do you think the women would prefer?
185. *R: [Laughs] it is still hard, some would prefer the self-collecting method because you people were seeing our private parts with the previous method whilst others would prefer the VIA method, it is hard to really know what they would think [chuckles].*
186. I: Alright, you have talked of transport as one challenge with the self-collecting method, what other challenge do you think women would experience?
187. *R: The main challenge is transport, especially after you are told to come the next day for your results. Most women will not go back for the results.*
188. I: Alright, what problems do you think women would have with the self-collection?
189. *R: No, I don’t think they would, they would collect it. But as I have said, we people reason differently, some would prefer to collect it themselves.*
190. I: Alright, now let’s talk about your recommendations for the future of the National cervical cancer screening in Malawi. In your opinion, should the ministry of health consider including self-collected vaginal swab for cervical cancer testing to the cervical cancer screening program?
191. *R: SO that there should be that other one and this one?*
192. I: Yes, there should be 2 methods of screening where one can either be screened through VIA or through self-collected vaginal swab.
193. *R: Alright, there is no problem. The woman would actually have a choice, so I don’t think there would be a problem.*
194. I: Alright, do you think this would make it easy for women to undergo screening?
195. *R: If they were coming in the communities it would.*
196. I: Okay, so the two methods should be there but they should come with them to the communities?
197. *R: Yes, because if the women still have to come to the hospital that would be a problem. If they came to communities, there would be no problem. Most women would not make it to the hospitals, believe what am saying [laughs].*
198. I: Alright, what groups of women could be most suitable for self-collected vaginal swab for cervical cancer testing?
199. *R: [Silence]*
200. I: Which age group or which profession of women do you think would be most suitable for this method?
201. *R: You know, even those with HIV are get screened for cervical cancer, because they also need to be assisted. I would have also loved if the girls could also be screened. Like I said, the girls nowadays are different from the way we were as girls. We could easily listen to what we have been told not to do, but that is not the case with the girls nowadays.*
202. I: Alright, you have talked of girls and those with HIV as well, which other groups do you feel would be most suitable?
203. R: The older ones as well, those above 50 years old, because I think they said that those who are above 50 years old should not be screened, but they are also dying of cancer so they also need to be screened.
204. I: You think they should also use this method?
205. *R: Yes.*
206. I: Why do you think so?
207. *R: The main aim, even for the Malawian government is to reduce the rate of cervical cancer which is killing a lot of women. They want a healthy Malawi, so that is the reason.*
208. I: Alright, which groups of women wouldn’t be suitable for the self-collecting vaginal swab?
209. *R: Then those would be the pregnant and those who are menstruating. I think that is also what they told us.*
210. I: Alright, we have talked of a lot of things.
211. *R: Yes, we have.*
212. I: But before we finish, is there anything you would like to add?
213. R*: What I can add is just an encouragement that this idea of screening for cervical cancer in the communities is a very good idea, the government did very well by thinking of this. Prevention is better than cure and for someone to receive treatment after things have gotten worse is not good. So I think you should continue with this method so that a lot of women should be assisted, that is what I can say.*
214. I: Alright, any questions?
215. *R: With the discussion we just had, I don’t have any question.*
216. I: Alright, so thank you very much for your time.
217. R: Thank you.

THE END
