# Supplementary material for: Perceptions of cervical cancer and motivation for screening among women in Rural Lilongwe, Malawi: A qualitative study
Source: PLoS One. 2022 Feb 7;17(2):e0262590. doi: 10.1371/journal.pone.0262590 (PMC8820632; doi:10.1371/journal.pone.0262590)
Supplement: S3 File — (ZIP) [file pone.0262590.s003.zip › VIA_116 Missed.docx]

**PARTICIPANT ID: VIA_116**

**INTERVIEWER: 466**

**DATE OF INTERVIEW: 27 Nov 2017**

**TYPE OF INTERVIEW: Missed 6 and 12 weeks follow up visits**

**TRANSCRIBER ID: 969**

**KEY: I= Interviewer, R= Respondent**

**Interview summary:**

This participant reported that she missed her scheduled follow up visit date because she was suffering from malaria and when she started getting better her child was sick as well. The participant did not explicitly admit that she was found with cancer cells on the cervix. According to her, she was told that she had a sore which was caused when she was giving birth to her baby. About joining the study, she said she was not aware that she was joining a study and according to her, she said it was because she does not know how to read or write. After probing more, she admitted to have consented for the study but she said she was afraid because she thought she would be screened using the same method that was used while in the community which she said was painful. She said she got the Thermo-coagulation treatment which was very painful and she had abdominal discomfort for some days. She explained that she was concerned when she was traced because some people in the community knew that she was in a study when they saw the tracer. She however said that she was happy that her sore was treated and that she got the right treatment.

**Interview text:**

1. I: So, thank you for meeting with me today. I really appreciate your time and your input will be very helpful. I am working with a team of researchers from the University of North Carolina Project in Malawi like here where we are now. Sorry you were not able to come for your 12-week follow-up appointment. We would still like to hear from you regarding your experience with the cervical cancer screening with VIA and thermo-coagulation treatment. We would also like to hear about any difficulties you had afterwards or any challenges you had to coming for your follow-up visit. So your input is also important to help us understand how best we can conduct cervical cancer screening campaigns in Malawi. There is no right or wrong answer. Everything you say will be kept confidential and that is why we are the two of us here
2. *R: Okay…*
3. I: . Yah, so the aim is to make sure that the information you give should only be used to make this health program and health questionnaire better. So for me to capture whatever you will say, i will audio record this interview to help me remember what was said but your name or any identifiable information will not be connected to anything you say. first we should talk about the VIA screening and Thermo-coagulation treatment experience: Can you tell me your understanding of the cervical cancer screening and treatment you received soetime ago?
4. *R: When I went there I was tested for HIV and when I went for VIA I was told that I had cancer cells. Then, I did not get screened the same day because the doctor who was doing the screening was not available and I was told to come on Monday morning because that was a Saturday. I was asked “How old is your baby?” I said “Three months.” And they told me that “You are supposed to be seen by a Senior doctor from (Name of hospital) but because s/he is not here today, you should go back and come back on Monday morning. You don’t have to be on the line[queue].” So on Monday I left around 12 noon after cooking food. When I went there I was asked “How old is your baby?” I said “Three months” then they said “The sore that was caused by the birth of the baby is still fresh and we need to thermos-coagulate it.*
5. I: What sore were they talking about?
6. *R: Maybe the sore that was caused by the birth of this baby. Yes but when they applied the medication, I had a strong pain in the abdomen.*
7. I: How was the baby born?
8. *R: What?*
9. I: Was the child born vaginally or through Caesar?
10. *R: S/he was born vaginally.*
11. I: Okay...
12. *R: Yes. They were talking about the sore inside the uterus.*
13. I: The sore inside the uterus…?
14. *R: Yes, that was what I was told because we are not able to see inside the vagina. So when they heard that the baby was three months and one week old, they applied the medicine which cause a strong pain in the stomach. Then I woke up and wore my pant…*
15. I: How long did the pain last?
16. *R: The whole month and extended to the next week. I was bedridden and the father of this one grew some vegetables but I never took part in selling them, it was my younger sister who was selling them. The pain was as strong as labor pain. Even the vaginal discharge that was coming out was stinky one; the white one had stopped coming out. I was like; “Is this caused by the medicine that they have applied on me?” That time my mother and my sister were saying “They have taken your energy…”*
17. I: What was this energy they were talking about?
18. *R: It is like when you are having sex, the vaginal discharge is mixed with semen so that content is what they were talking about. I was still in strong pain and I was eagerly waiting for my follow up visit date so that I should go and they should see what the problem is. Even the vaginal discharge, it was yellowish in color and very stinky. I stayed for one month while in pain and during the second week of the other month, it was when the pain ceased a little bit. So when I came here, they asked me; how have you been feeling? I said Since that time, I have been in pain except for this week when I am able to walk a distance. Even at home, my husband grew a lot of vegetables but I have never taken any part in selling those vegetables because of the strong pain in the stomach. I was even failing to eat and when I wanted to urinate, the pain was so strong. They said “It happens like that when they apply you the medicine but after wards it will stop and you will be normal.” So I have seen that up to now the pain is no more. I have been able to do piece works because the father of this one has three wives and he rarely takes care of my family. As I am talking now, I left home with no food and the father of this one is with the other wife. Even though my children have gone for piece work, they have gone there on an empty stomach. So when I was told to come on a follow up visit, I said to myself; “Of course they have said that I should go again, but what am I going to use for transport?” That time, I did not have any money. I was told to come here on 22nd and that was when I was sick. I had strong headache and when I was recovering, the next to be sick was the child. I have just taken him when I was coming here.*
19. I: Okay. Truly It can be hard for people to come for follow-up; apart from being sick, what other problems made you fail to come for your follow up visit?
20. *R: I failed to come because as I said earlier on, I had malaria. So it was yesterday when they [study staff] traced me through the Group Village Head who tends to be my uncle. So he called for me and asked “ I hear you did not go for your follow up visit maybe because you were sick?” I said “Yes.” “So they say you should go there tomorrow.” So here I am today.*
21. I: Okay. Meaning that your main challenge was the sickness?
22. *R: Yes.*
23. I:Okay. What challenges do you think other women have to come for follow-up?
24. *R: I may not know what challenges they may have.*
25. I: Just imagine what challenges you think other women have to come for follow-up visit… there are many challenges in the communities….
26. *R: Maybe funeral, or maybe her child is sick and she has gone to a different health facility… maybe those can be the challenges.*
27. I: What else?
28. *R: Maybe what people say in the communities that studies are fearful.*
29. I: How?
30. *R: [No response].*
31. I: How do you travel to this place?
32. *R: It is a long distance that we walk from where I live to [A place]. From (Name of place), it is where we take minibuses.*
33. I: So what can be the challenge with mobility?
34. *R: It means transport.*
35. I: What other challenge?
36. *R: The problem can be if they have no transport.*
37. I: So how best can we help women overcome those challenges?
38. *R: That is difficult; I don’t know you can help them.*
39. I: You can just imagine.
40. *R: It is difficult.*
41. I: Sometimes you can guess for a solution.
42. *R: Maybe by just giving them transport or coming and find them the way they did, maybe I should say so?*
43. I: Meaning transport should be left there in the community?
44. *R: Maybe. Like in my case, I don’t do any business but I tend to be the bread winner in the house and right now I am not doing any business but the father of this one cannot help with anything. As I am talking now, in my house, there is no single seed of maize. So that is why when they said “You should come,” I just stayed because I had no transport.*
45. I: Okay. So I was asking about challenges because you had already explaining that you failed because of sickness.
46. *R: Okay.*
47. I: Now we should continue talking about your experience of cervical cancer screening and thermo-coagulation treatment. We would like to hear your thoughts about cervical cancer screening campaigns like this one where you got screened, why did you choose to get screened or participate in this study?
48. *R: I should say the truth; I did not know what was happening. I just heard that they are screening for cervical cancer but because I don’t know how to write and read, I did not know the details of the service. So that day I was with my friend and we were going to register for identity cards; on our way we encouraged each other to say “It is better to screen for cervical cancer nowadays because of how the disease is affecting women.” So we agreed to go there and as I said, they did the documentation and told us to test for HIV and they also tested urine. I was told that I was HIV and pregnancy negative. Then I went for cervical cancer screening; I was sent back because I was told to be seen by a senior doctor. When I came it was when I was told that my cervix is okay but I have a sore that was caused by the birth of the baby.*
49. I: They said the cervix is okay?
50. *R: Yes.*
51. I: What did they mean?
52. *R: I don’t know because the doctor told me that the sore caused by the birth of the baby is not healed. That is what they said. And when they said that it was when they did the thermos-coagulation but the pain in the stomach was so strong because of the treatment.*
53. I: What I want to understand is that we are discussing about cervical cancer screening;
54. *R: Yes.*
55. I: So what did they say regarding cervical cancer screening results?
56. *R: They said that I don’t have the cancer cells but the sore caused by the birth of the baby and that was when they treated me with thermos-coagulation.*
57. I: Okay…
58. *R: Yes.*
59. I: So why did you decide to join this study?
60. *R: That is the question I wanted to be asked; I did not know anything about research; I just saw that my friends were going there. Like when they came I was asked “Why are they tracing you to the extent of going to the GVH?” I said “Maybe because I missed my scheduled visit date when I had malaria and before I recovered the child was also sick. That is why they have come..” “Maybe you joined a study?” “No. had I known that it is a study, I would not have joined because of what I hear about research.*
61. I: You would not have joined?
62. *R: Yes, and I was asked; “Do you remember about [name withheld]?” I said “No” “She joined a study and she was being followed.” “Had I known that it is research I would not have joined because I don’t know how to read and write, maybe that is why I joined.” I was answering like that.*
63. I: So my question is; when you were told about a study; was a consent form read to you so that you could sign and join a study? Because participation in a study is voluntary; no one is forced to join a study…
64. *R: I was just being asked; what is your name? where do you live? Who is your GVH? “ Because there was a crowd and I was just responding to what I was asked.*
65. I: But then, did they read a consent form for you so that you could consent for your participation in a study?
66. *R: I was just thinking that the study they are talking about is that the want to screen and give me treatment.*
67. I: But did you consent for your participation?
68. *R: That is why I am telling you that I did not know anything…*
69. I: You just told me that you wanted to be screened and treated right?
70. *R: Yes, about the cervical cancer.*
71. I: I am asking you all these questions because you joined a cervical cancer study…
72. *R: Yes.*
73. I: That is why I am asking you; how did you join the study?
74. *R: That is what I am saying that I don’t know anything because I don’t know how to read. Had it been that I know how to read, I would have known that this is a study and this is not a study but because I don’t know how to read that is why I don’t know anything. It was this time when they traced me that people were saying “If they have followed you, it means it is research.” Because during that time some women were referred to go to (Name of hospital) and I asked them “When we were being referred that time; were you referred to (Name of hospital) or (Name of hospital)?” They said “We were referred to (Name of hospital).” “How were you examined? Was it the same way as they did during the screening here?” They said “No, they were just giving us pills.” They said “Here when they found that we had cancer cells, they treated us with thermos-coagulation. When they referred us to (Name of hospital) they just gave us pills.”*
75. I: Were they referred to *(Name of hospital)*?
76. *R: Yes, they were referred to (Name of hospital).*
77. I: By the same study team that screened you in the community?
78. *R: Yes, the same team. For me to know this, I called my friends when I arrived here at KCH; “Where are you?” “We are here at (Name of hospital).” “not to (Name of hospital)?” “NO.” “But I am here at (Name of hospital).” And they gave me a map to reach here. So when I came I found the nurse who escorted me here and she asked; “How can we help you?” I said “When they came to our community, they screened me and told me that my cervix has no cancer cells but I have a sore that I had when I was giving birth to my child and they treated me but I had very strong pain in the stomach.” And she said “It happens to feel pain like that when you have been treated but with time the pain goes.” That was when I was scheduled to come on 22nd last month which I missed to come.*
79. I: So why did you come on that day?
80. *R: When?*
81. I: The day when you came here?
82. *R: I came because I thought they were going to ask how I was feeling after screening and treatment.*
83. I: But still my question is on the consent; was it read to you so that you understand it?
84. *R: Yes.*
85. I: Did they give you a copy of the consent form?
86. *R: It seems I have it here [Participant looks for a consent form in her bag.]*
87. I: Yes, I wanted a form like this one. Are you the one who thumb printed here?
88. *R: Yes.*
89. I: Was it read to you?
90. *R: Yes.*
91. I: I am not trying to police you but research has got rules and regulations. We are collecting information from you and without this consent form, I cannot continue collecting information from you that means I am doing it unethically and it can be a case against me. By signing this form it means you have confirmed that you have understood the contents of this form and that you have voluntarily accepted to take part in a study. Research studies are approved by regulatory bodies including the government of Malawi.
92. *R: But now my question is; Am I going to be screened and treated the way I was screened and treated when they came to our community?*
93. I: For that question, you can get satisfactory answers from the nurse who escorted you here or any medical personnel. So after this interview I will refer you to the right person to answer you. Understood?
94. *R: Yes.*
95. I: Was there anything you were worried about before the screening?
96. *R: The worry was that some people were running away saying that…. [Chuckles]… Since we are alone here, I will tell you because what people were saying….*
97. I: Feel free; tell me anything you feel I should hear from you. As I said, there is no right or wrong answer; whatever you will tell me here will be very important to this study. So I want to hear as much information as possible from you; so tell me everything you feel I should know.
98. *R: Like my friend whom I came with run away before she was screened…*
99. I: Why did she run away?
100. *R: She was afraid because they were saying that “they are taking out the uterus” because they were indeed taking out the uterus to treat it.*
101. I: Was your uterus taken out when they were treating it?
102. *R: They just screened it and treated it.*
103. I: So what is your worry?
104. *R: My worry is that; am I going to be treated the way it was done on the first day of screening?*
105. I: That is your worry?
106. *R: Yes.*
107. I: Okay. When you heard that the results of your VIA screening was abnormal, how did it make you feel?
108. *R: When they treated me?*
109. I: When you were screened what were your results?
110. *R: They told me about the sore that was caused by the birth of the baby.*
111. I: What did they tell you about cervical cancer?
112. *R: That they found me with cancer cells?*
113. I: Yes..
114. *R: No.*
115. I: They did not tell you?
116. *R: No.*
117. I: They did not give you the results of the cancer screening?
118. *R: No they did not.*
119. I: But you told me that they said you did not have cancer cells…
120. *R: Yes, but they said I had a sore that was there because I had given birth.*
121. I: So how did that make you feel when you were told about the sore?
122. *R: I was happy that I was not found with cancer cells.*
123. I: So why do you think you had thermo-coagulation?
124. *R: What?*
125. I: Why did they treat you?
126. *R: They said they were treating the sore. That was what they said.*
127. I: You have said that people were saying that the research staff take out the uterus…
128. *R: “They take out the uterus…this is not a good thing… “That is what they wee saying.*
129. I: What other misconceptions were people in the community saying about the screening?
130. *R: Some were talking positively because when they were screened, some were found with some small swellings in the stomach and when those were found, they were treating them with thermo-coagulation. To them it was a positive development because those small swellings would turn into cancer but once treated it means the cancer would have no chance to attack them.*
131. I: Were you not found with the small swellings?
132. *R: To say the truth, they did not tell me. I just heard about the sore.*
133. I: Fine…
134. *R: But my friend whom I went with, told me that she was found with those swellings and she was treated; there was also this other woman, she was also found with the swellings and she was treated but there was another woman who also said she had a sore that was caused by the birth of her child but surprisingly, her child is older than mine. So people were saying; “How come that she can still have the sore that was caused by the birth of a child who was born long time ago? How possible is this?” So I think those were the people who were referred to (Name of hospital) for further management.*
135. I: Which group was this?
136. *R: Those whom I am talking about.*
137. I: Which ones?
138. *R: Those who were found with small swellings, they said they went and they were given injection. For the others, they were just given pills without injections. The woman I am talking about that she was found with a sore[caused by the birth of the child], she was just given pills.*
139. I: Okay.
140. *R: Yes, so I asked them; “Do they screen as they did initially?” They said “no.”*
141. I: I will be like repeating what I asked you already; what is your understanding of cancer screening?
142. *R: They said that if one was found with infection, she should be treated. Maybe someone was feeling hot in the stomach or she had strong pain in the stomach, she should be treated.*
143. I: What infection did they say it was?
144. *R: They said cancer.*
145. I: How did they say they diagnose cancer? How does it look like on the cervix?
146. *R: The cervix?*
147. I: Yes.
148. *R: They said that the cervix has swellings.*
149. I: How different were the cancer cells that were defined to you from the sore that you were told you had?
150. *R: They were different because my sore was caused by the birth of the baby and not cancer.*
151. I: Okay. What do you think was done well during the screening?
152. *R:What was done well according to me was the thermo-coagulation treatment after they had found you with the cancer cells or candidiasis.*
153. I: How did you understand the thermo-coagulation treatment that was done on you?
154. *R: I thought I had the thermo-coagulation because of the sore which they told me about.*
155. I: Did they not tell you that they had found you with cancer cells?
156. *R: Maybe that is the case.*
157. I: I want to learn from you because I was not there when you were being treated so I want to know what really happen to you during the cancer screening and treatment exercise.
158. *R: Because when they screened me, they told me that “We have found you with a sore on the cervix and we are going to do thermo-coagulation treatment. How old is your child?” I said “Three months old.” Then they said “After treating you, you should stay for six weeks without having sex.” And I asked them “Like what we do after we have given birth?” They said “More less like the same because you are not supposed to have sex for the said period so that the sore should be healed. Now my thinking was that it was the sore that was caused by the birth of the child not knowing that it was cancerous.*
159. I: So what was your experience to stay for six weeks without sex?
160. *R: I should say I stayed for one month at home; the fifth week, t was when I came here. That period I did not have sex.*
161. I: Okay. what was his reaction when you explained to him that you would stay for six weeks without sex?
162. *R: When I explained to him?*
163. I: Yes.
164. *R: He said “Why?” I said “They have said that the child is still young. They said inside my uterus there is still a sore that was caused when I was delivering this child; it is not yet healed. So they have said that we should not have sex. We are supposed to wait for one month and two weeks.” He said “We are supposed to obey what the doctors tell us. It is okay.” So after one month, I came here the second week of the other month.*
165. I: Not the first week of the other month?
166. *R: No the second week of the other month.*
167. I: Okay, fine. Does your partner know that you were screened for cervical cancer?
168. *R: Yes.*
169. I: What does he think?
170. *R: He just knows that when I was treated, it meant the sore was healed.*
171. I: So apart from your partner, who else did you tell that you were screened for cervical cancer?
172. *R: People from my community know this then my mother and my sister. That time when I was sick it was when they were saying things like “Why did you join this? They draw blood and send to Tanzania?”*
173. I: Why did they say blood is sent to Tanzania?
174. *R: They were saying that there people don’t give birth.*
175. I: Who don’t give birth?
176. *R: People of that country.*
177. I: So why do they send the blood?
178. *R: [Laughter]….*
179. I: I want to learn from you…
180. *R: It was when my son told them that “Aunt, you will be sick the way mother has been sick…” Because I was very sick that time; and my son said “Mother, don’t bother about what people are saying. It doesn’t matter if you die but the treatment you get for your disease is vital.” I said “I went there to get treatment and live a normal life and not what these people are saying. I went for screening because I had strange signs in my body and that I am sick now, it doesn’t matter. During my next visit if I will still be sick, I will explain to her that since I received the treatment, I am still not feeling well and they will know what they can do on me.”*
181. I: So what support did you get from your partner during screening and treatment of cancer cells?
182. *R: When I went for screening and treatment, I told him “I went for cancer screening and treatment and they have treated me and told me that we should stay for six weeks without having sex” and he said, “That is fine so long as you have gotten the treatment I have no problem.”*
183. I: What else does your partner do as part of support since you got screened for cervical cancer?
184. *R: In terms of the transport that he gives me?*
185. I: Whether transport or any other support…
186. *R: Yes, he gives me transport and when I have delayed, he follows me. Maybe if the transport he gave me was not enough, he follows me to give me another transport because he sells apples and grapes in town here.*
187. I: Okay fine. Do you think male partners should be more involved with cervical cancer screening for women?
188. *R: Can you come again?*
189. I: Do you think male partners should be more involved with cervical cancer screening for women?
190. *R:They should be taking part?*
191. I: Yes, like escorting them when they are going for screening?
192. *R: Yes…*
193. I: Or escorting the women to the hospital?
194. *R: To say the truth, he never escorts me…*
195. I: I am asking about men in general…
196. *R: Oh, other men?*
197. I: Yes, like what is your opinion?
198. *R: Okay, they do escort…*
199. I: Do you think they should be more involved?
200. *R: [Chuckles]…*
201. I: I want to learn from you…
202. *R: [No response]…*
203. I: Other men including your partner; do you think it is necessary that male partners should be more involved with cervical cancer screening for women?
204. *R: [No response].*
205. I: Have you understood the question?
206. *R: That is why I was saying…*
207. I: Let me give an example; imagine when you are going for antenatal clinic, we encourage that men should…
208. *R: Should be escorting their spouses….*
209. I: They should be involved right?
210. *R: Yes.*
211. I: So that you should be together when the health workers are providing information…
212. *R: He should also be there.*
213. I: Yes. So now we are talking about cervical cancer screening; like do you think it is necessary that male partners should be more involved with cervical cancer screening for women like the way they do with antenatal clinics?
214. *R: Okay…*
215. I: What is your opinion?
216. *R: Other men can be involved but in terms of the father of this one, never.*
217. I: Why can the father of this one never be involved?
218. *R: To him, so long as he gets the help; on that one, he is an expert, he loves it so much.*
219. I: Like what help does he get?
220. *R: He loves sex very much…*
221. I: [Laughter]….
222. *R: But in terms of support… like you can tell him ‘I am pregnant and we need to go to the clinic for HIV testing,” He doesn’t accept it and he will never. This is our six child but he has never escorted me to antenatal clinic, I go alone. Sometimes it happens that I deliver at the Tradition Birth Attendant; he pays for the cost like K2,500 but he rarely comes or if he comes he will be brief. He brings two coca-cola drinks and two bans. That is all he can do. He does the same things with other pregnancies.*
223. I: But in your opinion, do you think male partners should be more involved with cervical cancer screening for women?
224. *R: Mm…*
225. I: We need your advice…
226. *R: In terms of….?*
227. I: Men being involved when women are having cancer screening services from the hospital…
228. *R: If they are cooperative enough, they should be taking part.*
229. I: What makes you think that way?
230. *R: I am talking for other women not myself, it is impossible.*
231. I: For the other women, why should men be involved with cervical cancer screening for women?
232. *R: They should understand like what they told me that we should stay for six weeks without sex, they should also be there.*
233. I: meaning that you should have the information while you are together?
234. *R: Yes.*
235. I: Okay. what is the other reason why men should be involved with cancer screening of their partners?
236. *R: So that their lives should be healthy.*
237. I: Another point?
238. *R: That is all.*
239. I: Okay. so what was difficult during the time when you were having cancer screening and treatment?
240. *R: As for me, the difficult part was to trace me through the Group Village Head of our community because it made people feel that I had joined a study.*
241. I: So what is your problem about joining a study?
242. *R: [Chuckles]…*
243. I: What has been your experience when you came for your follow up study visit?
244. *R: Because people at home are talking a lot about me that I joined a study.*
245. I: How does that make you feel?
246. *R: Nothing because when I came here I got the required treatment that I needed and there was nothing wrong I experienced like what they were saying I did not see it here…*
247. I: Perhaps did the tracer told other people why he was following you up?
248. *R: No, he just told the GVH and because during the screening campaign the GVH was encouraging all women to go for cancer screening and when he was told, he encouraged me to go to the hospital.*
249. I: The GVH?
250. *R: Yes.*
251. I: So how did the other people knew that you were going for a study?
252. *R: Because they saw the tracer and there was another woman in the village who was in the study and he was being followed the way I was followed so that is how they knew.*
253. I: Not that they were told?
254. *R: No, they just thought on their own.*
255. I: Oh, okay. I wanted to understand that. Now that you came here, how do you feel about being in a study?
256. *R: I don’t see any problem. My understanding is that I was followed because I had missed my scheduled visit and had it been that I did not miss the visit, I would not have been followed. I missed because I was suffering from malaria.*
257. I: Okay, fine. Now let us go back to the men; how best do you think we can educate men about cervical cancer?
258. *R: Like how we are going to educate them when we go home?*
259. I: Even us health workers; how should we educate them?
260. *R: I don’t know how we can do it.*
261. I: Is it difficult?
262. *R: [Chuckles]…*
263. I: Is there anything new you have learned about cervical cancer or cervical cancer screening that you did not know before the study?
264. *R: As I have told you earlier on, I did not know that I was found with cancer cells but I knew that I was found with a sore that was in my uterus after my baby was born. However, after treating the sore, it was when I had a strong pain in the stomach; the pain was so unbearable.*
265. I: What new thing did you not know about cervical cancer?
266. *R: New thing?*
267. I: Yes.
268. *R: I was happy that after being found with a sore that was caused because of the birth of the baby, I was treated and I was happy. I never knew that after giving birth we do have a sore in the uterus.*
269. I: Did you believe that the sore was caused by the birth of your baby?
270. *R: Yes. Maybe they were cheating me they found me with cancer, there I don’t know…*
271. I: No, the doctor doesn’t cheat, he will always tell you what s/he has found.
272. *R: Because when I was screened on the first day, it was when I was told to be seen by a senior doctor. When the senior doctor came, s/he told me that “you have a sore; how old is your baby? We are going to treat you.”*
273. I: Okay. so what did you know about cervical cancer?
274. *R: What I knew?*
275. I: Yes.
276. *R: Like what?*
277. I: Anything that you can say.
278. *R:I knew that now I don’t have cancer because I was treated.*
279. I: What did you learn about the cause of cancer?
280. *R: They said it starts with swellings on the uterus and it goes inside.*
281. I: How about prevention of cancer?
282. *R: Preventing it?*
283. I: How can it be prevented?
284. *R: There is need for thermo-coagulation treatment so that we are protected.*
285. I: Who do you think should be screened for cervical cancer?
286. *R: In our community?*
287. I: Anywhere else…
288. *R: Like the type of person?*
289. I: Yes.
290. *R: There are many people who are supposed to be screened.*
291. I: Which are the people?
292. *R:Should I mention names?*
293. I: How many types of people are there?
294. *R: Oh, women.*
295. I: Women?
296. *R: Yes.*
297. I: What type of women?
298. *R: They should be of our ages but she should not be pregnant?*
299. I: Why not for pregnant women?
300. *R: Because when they came, they said that pregnant women should not be screened.*
301. I: Why did they say that?
302. *R: They just said that pregnant women should not be screened, I don’t know why.*
303. I: Okay. How often should women be screened?
304. *R: They said after three months I think.*
305. I: I want to hear your opinions on how often you feel they should be screened.
306. *R: I heard about that and I don’t know…*
307. I: In your opinion, how often do you think they should be screened?
308. *R: Maybe she can go for screening when she feels pain in her body…*
309. I: Is that what you think?
310. *R: [No response].*
311. I: Because we want to hear your opinions.
312. *R: [Chuckles]… We need to help each other… when I am failing to answer you need to help me.*
313. I: To us, what ever you tell us is very important.
314. *R: [Chuckles]…*
315. I: There is no wrong or right answer.
316. *R: [No response]…*
317. I: Is it hard?
318. *R: [Chuckles]…*
319. I: Fine. What do women in your community think about cervical cancer screening?
320. *R: What do they do?*
321. I: What do they think about cervical cancer screening?
322. *R: About cervical cancer screening?*
323. I: Yes.
324. *R: Some think that if they can be screened, they can have a healthy life but others as I have already told you, they are afraid.*
325. I: Do you think that women in your community understand the importance of cervical cancer screening?
326. *R: What do they do?*
327. I: Do you think they understand the importance of cervical cancer screening?
328. *R: Yes.*
329. I: What makes you think that way?
330. *R: They understand because they want to know their status as regards with cancer.*
331. I: In your opinion, do you think that women are interested in receiving this screening and treatment service?
332. *R: Women?*
333. I: Yes, women in your community; do you think they are interested in receiving this screening and treatment service?
334. *R: Yes.*
335. I: What makes you that way?
336. *R: As I have already said, they are interested because that time when we went, there were many women who went for screening so that they could know their statuses with regards to cancer.*
337. I: Why do you think some women would not want to be screened?
338. *R: Thinking?*
339. I: Why would someone not want to be screened? “Go, I cannot go there.”
340. *R: Some would not want.*
341. I: Why would they not want?
342. *R: Because of fear of what their friends tell them; “They are doing this and that…” So she can just run away while others are getting screened because she has feared what others are saying.*
343. I: What are some of the barriers that women might face in receiving this service?
344. *R: Barriers?*
345. I: What are some of the barriers that women might face in receiving this service?
346. *R: They can be regretting that they ran away when their friends were getting screened. “Had I known I would not have run away…” That time they are very sick, bed ridden and they regret that they did not get screened when they were healthy.*
347. I: What else would prevent them from receiving cervical cancer screening service?
348. *R: That is what I am saying; the fear…*
349. I: Apart from the fear, what else?
350. *R: The other thing is the pregnancy I was talking about.*
351. I: What else? Family issues?
352. *R: Okay, maybe the husband stopping her from getting the service.*
353. I: Concerning friends?
354. *R: Maybe friends stopping them from getting the service.*
355. I: Okay, fine. In your opinion, how should cervical cancer screening be provided to ensure that more women can get screened?
356. *R: To increase the number of women to get screened?*
357. I: Yes.
358. *R: Should I be telling them when I go home?*
359. I: I want your opinions; how should cervical cancer screening be provided to ensure that more women can get screened?
360. *R: So that we can do the work?*
361. I: So that many women can get screened…
362. *R: By providing the information in the communities. Because when they came to follow me up, people were anxious to know what they wanted and in the same way if they can come to sensitize the whole community, people cannot be asking questions or talking negative about cancer screening because they would have firsthand information.*
363. I: How can you encourage the women to go for screening?
364. *R: [No response].*
365. I: Fine. Now I want us to discuss about self-collecting vaginal swabs; Let’s now discuss about self-collected vaginal swab for cervical cancer screening. I heard you saying that women were afraid to get screened because they heard that the uterus is taken out of the womb…
366. *R: Yes.*
367. I: So, [Noise of a child making noise in the background]. a new method has been developed for cervical cancer screening. It involves having a woman collect a swab from her vagina and submitting it at her convenience to a health facility for testing. However, unlike the screening procedure that you went through[VIA],, the woman would not get her result immediately and would have to return to health facility to get her result a few hours later or the next day. What do you think about this method?
368. *R: [No response].*
369. I: That a women should be self-collecting a swab from her vagina and submitting it at a health facility for testing at her own convenient time?
370. *R:[An irritated child making noise. Participant talks to the child to stop her from making noise]… You mean collecting a swab?*
371. I: Yes.
372. *R: You should take it yourself from the vagina?*
373. I: Yes, you should collect it with a swab and submit it at a health facility for testing. After the test, they tell you whether you have cancer cells or not…
374. *R: The swab is good.*
375. I: The swab is good?
376. *R: Yes.*
377. I: What makes you think that way.
378. *R: [Takes time to respond] What do you think?*
379. I: I am asking you. The aim of these questions to you is that we should collect your opinions and those opinions will help to develop questionnaires and to improve this screening program which will help other people in future. That is why I am trying to make you talk so that we can get as much information as possible from you.
380. *R: That is what I have already said that during that time they were pulling the uterus and apply medicine and reinsert it inside so some women were afraid of that and they ran away. There was a crowd of women at the screening venue but when they heard about this, they started leaving one by one. So if this method can be used to screen cervical cancer, it can be helpful and many women can be willing to get screened because you can just give them the swab and they collect the vaginal discharge on their own and give you for testing. This can be a very simple procedure and many women can go for this.*
381. I: Would you be interested in undergoing screening this way?
382. *R: Very much so.*
383. I: Okay. What do you think is the advantage of this method?
384. *R: As you are saying, screening for cervical cancer would be simple using this method..*
385. I: What are the disadvantages of this method?
386. *R:[No response]…*
387. I: Any disadvantages about this method?
388. *R: Okay I should ask you a doctor; when you give us the swab, how shall we be collecting the discharge?*
389. I: You will be inserting the swab inside the vagina and then take it out…
390. *R: Inserting it inside?*
391. I: Yes.
392. *R: The way we insert herbal medicine to abort pregnancy?*
393. I: I don’t know what you do to abort but…
394. *R: No, I have never aborted but some people insert herbal medicine in the vagina to abort…*
395. I: Okay. [Interviewer demonstrates how swabbing would be done] you take a dry swab like this,
396. *R: Yes.*
397. I: Then you insert it in the vagina
398. *R:Will it be there overnight?*
399. I: No. After few seconds you remove it from the vagina… we women we do produce vaginal discharge naturally right?
400. *R: Yes.*
401. I: Yah, so the swab will come out with vaginal discharge on it. So the vaginal discharge that will stick to the swab is what you will take to the health facility for testing.
402. *R: Okay…*
403. I: So I was asking what could be the disadvantage of this method?
404. *R: If it is like this, then it is a good method unlike the VIA method I was talking about.*
405. I: Okay. so where do you think this procedure should be done?
406. *R: Where you will be going…*
407. I: I want to hear your opinion about where you think can be the best place to collect the swab…
408. *R: You are saying the testing is instant right?*
409. I: No, you will submit the swab at your convenient time…
410. *R: Like I submit today…*
411. I: Say you submitted at 8 in the morning then they will tell you that “Come at one in the afternoon to collect your results.”
412. *R: Then we have the right to get the results.*
413. I: So where should the swabs be collected?
414. *R: It means where you went at (Name of place)…*
415. I: You mean they should be coming to the communities to collect the swabs?
416. *R: Yes, because like when they came, there were many women who flocked to the screening venue and it will be easier that way.*
417. I: Meaning that they should be coming to the communities to collect the swabs?
418. *R: Yes.*
419. I: Suppose the health workers have not come to to the communities, they are right here at the hospital, where do you think the swabs should be collected by the women.?
420. *R: If they are here at the hospital?*
421. I: Like the way we are here, but the woman wants to collect vaginal swab to submit at the health facility, where should she collect the swab?
422. *R: It means she should come here at the hospital.*
423. I: Why do you think that way?
424. *R: She should collect it right here.*
425. I: She should collect it from here?
426. *R: Yes.*
427. I: How reliable do you think this method is?
428. *R: Because you have said that it is to be used for cervical cancer screening so it is reliable to choose this method.*
429. I: What do you think other women in your community would think about the self-collected vaginal swab technique for screening?
430. *R: Women in the communities insert bigger things in the vagina than the swab you are talking about. For example my husband has three wives; the first wife, I am second and there is the third wife. He rarely comes to my house and I have the right to insert herbal medicine in my vagina. Women do that; I should educate you as a woman; you might know it but you are a woman…[Laughter]… I take different herbs from the bush; the herbs that I know and I mix them together then I put them on a piece of cloth and insert it in my vagina… inside the vagina. You stay with it overnight and take it out I the morning when you know that he has gone to work. In the evening you insert it again; it means wherever he is, he will not be able to have sex. [Laughter].*
431. I: Do you do that yourself?
432. *R: I do this very often. If you hear about witches, I am one of them; In terms of preventing him from having sex, I am an expert but I have never aborted.*
433. I: Do you think more women would undergo screening with this method?
434. *R: Very much so because many women in my community are in polygamous marriages and they use this method I was describing to you. They insert herbs every night because their partners have other spouses like in my case so it would not be difficult for them to insert a swab in the vagina. They cannot have negative attitudes with this method unlike the method I was screened with, it is painful.*
435. I: What difficulties would women face in self-collection technique?
436. *R: If they can be screened?*
437. I: If they can collect swabs on their own, what difficulties would they face?
438. *R: I thought after collecting the swab, we will be giving you to do the testing and if you find that we have cancer cells, you would treat us?*
439. I: Yes, that will be the case.
440. *R: Yes, it is the same thing with those women after giving you the swabs, you will do the tests and give them results. You will treat them based on their results but they cannot have difficulties collecting the swabs because they are already used to inserting things in their vaginas. .*
441. I: What are some reasons if any why you think women would not want to self-collect?
442. *R: I cannot have problems with collecting vaginal swab but my worry would be here…*
443. I: Where?
444. *R: The pulling of the uterus.*
445. I: You thought “They would also pull my uterus?”
446. *R: That was my worry.*
447. I: So why would women not want to self-collect?
448. *R: Now that would be an individual’s problem because this is the simplest method.*
449. I: Why do you think women would prefer to go to a hospital for screening with medical providers?
450. *R: That would now be the choice of an individual.*
451. I: Why would that individual choose to go to a hospital for screening with a medical provider?
452. *R: [No response].*
453. I: Now let’s talk about your recommendations for the future of the National cervical cancer screening in Malawi: In your opinion, should MOH consider including self-collected vaginal swab for cervical cancer testing to the cervical cancer screening programme?
454. *R: This is my preferred method.*
455. I: Why?
456. *R: I have liked this method because when I compare this method with the method that I was screened, I prefer this one because that one was painful.*
457. I: So you think it should be included?
458. *R: Yes.*
459. I: Would this make it easier for women to undergo screening?
460. *R: Yes it would be easier because as I have already said women would easily insert the swab and collect the discharge that to be screened with metals.*
461. I: What groups of women could be most suitable for self-collected vaginal swab for cervical cancer testing?
462. *R:Groups?*
463. I: Groups of women that could be most suitable for self-collected vaginal swab for cervical cancer testing?
464. *R: They are many.*
465. I: Like which group of women?
466. *R: Like women of my age, like… I should just say married women.*
467. I: Why married women?
468. *R: Because elderly women cannot be screened. They are too old.*
469. I: Fine. What groups of women would not be suitable?
470. *R: The aged are not supposed t provide vaginal swabs because they are old.*
471. I: Thanks. Do you have any questions or additional comments to what we have discussed?
472. *R: No.*
473. I: I thank you so much for your participation in this interview. Your time is very crucial but the information that you have provided will be helpful in future campaigns of cervical cancer screening. Thank you very much.
474. *R: Thanks.*

End of interview.
