# Supplementary material for: Perceptions of cervical cancer and motivation for screening among women in Rural Lilongwe, Malawi: A qualitative study
Source: PLoS One. 2022 Feb 7;17(2):e0262590. doi: 10.1371/journal.pone.0262590 (PMC8820632; doi:10.1371/journal.pone.0262590)
Supplement: S3 File — (ZIP) [file pone.0262590.s003.zip › VIA_171.docx]

**PID: VIA 171**

**DATE OF INTERVIEW: 12 October 2017**

**INTERVIEWER: 466**

**TYPE OF INTERVIEW: 12 weeks follow up**

**TIME: 1 hour 20 minutes 16 seconds**

**TRANSCRIBER ID: 466**

**KEY: I= Interviewer, R= Respondent**

**Interview summary:**

This interview took place on 12 October 2017. It lasted for 1 hour 20 minutes 16 seconds. The woman looked composed and she was very active throughout the interview. According to this woman, she made a decision to get screened for cervical cancer when she heard that the screening crew had come to her area to do the exercise. She decided to go because she wanted to know her cervical cancer status because some women in her area had died of cancer. During the screening, she said she was screened several times by several health providers because they wanted to ascertain the results. When she was told that she had cancer cells, she said she was so afraid and she shivered because she thought she would die but after the Thermo-coagulation treatment and counseling, she regained her positive mood. She said she felt slight pain during thermocoagulation and she thought it was because the screening process had taken a longer time. She however said that she was happy to hear her results and get treated on the spot and to her this was well done.

Regarding the follow up challenges, she said problems like funerals and money can hinder some women to go back to the clinic as scheduled, and she suggested that the clinic staff should plan to pick women from the nearby points in their communities and take them back after the procedures.

In terms of support, she said that her male partner is supportive and he gave her transport money to use during her scheduled visit date. She also said that he promised to wait for the said period by the clinic without having sex. Regarding the community, she said there were some misconceptions like that the clinic staff collect vaginal discharge to sell it but she said she was able to refute such misconceptions because she never experienced something like that at the clinic.

The participant recommends that cancer screening should be done in the communities so that those women who were not screened can get the services.

Regarding the self-collecting vaginal swabs, she said that MOH should consider including this method of cervical cancer screening so that women should be able to choose which method they want to use.

**Interview text:**

I: Thank you for meeting with me today. I really appreciate your time and you should know that your input will be very helpful. Right?

R: Yes.

I: I am working with a team of researchers from the UNC Project; here where we are right?

R: Yes.

I: So your input is very important to us and will help us understand how best we can conduct cervical cancer screening campaigns in Malawi.

R: Okay.

I: So what you are going to tell us will help improve the cervical cancer screening campaigns. So in your responses, there is no right or wrong answer. We will receive everything that you say and everything you say will be confidential. That is why there are the two of us in this room.

R: Fine.

I: Yes. The information you give us is only to be used to make this health program and health questionnaire better. Yah. So as i said I will audio record this interview to help me remember what was said, but your name or any identifiable information will not be connected to anything you say. Right?

R: Yes.

I: Sure. So firstly, Can you tell me your understanding of the cervical cancer screening and treatment you received the past 12 weeks? What is your understanding?

R: My understanding is that when they came to brief us that “We have come to do cervical cancer screening, there were some who had fears, there were some whose partners were beating them… So when I went I said that “For someone not to go to the hospital, it is because of ignorance because it happens that you have an infection in your body which you are not aware of, but you refuse to go to the hospital. So those of us who have come are the ones who need the health care. So you should help us to know if we have a problem or not in our body.” They said “Okay, fine.” I said “Even at home, a woman can be pregnant but she refuses to go to the clinic. She delivers at home maybe up to 10 deliveries. So for us who have been going to the hospital, we go to the hospital.” And I explained to them that “I was once pregnant, and I was having fluid flows without experiencing any pain. I had no problem, but I was having fluid flows, then I started having blood flows, and when I went to the hospital I was assisted.” “Okay?” I said “Yes.” And said “You should screen us so that if we have the problem, you will tell us what to do.” Then it happened that the first one went, the second one went, the third one went, and I was the forth one. So when I entered, I spent almost one hour being screened; the first doctor screened and said “It seems like it has started but I don’t see clearly, let me call another doctor. Are you going to accept?” I said “Yes, I will accept.” S/he called another doctor.

I: How was s/he screening you?

R: S/he was screening on the cervix.

I: What equipment was s/he using?

R: S/he was using something, and s/he was putting medicine on it and insert then rubbing, s/he was lighting with a torch and was seeing while wearing glasses.

I: Fine

R: Yes. Then the other one came and said “It shows that it started, but I am not sure.”

I: What was it s/he was saying it has started?

R: The cancer. And they explained that it starts like small swellings… yah “So there seem to be three of such swellings. So would you like us to call another doctor?” I said “Yes.” So there were two males and two females. All of them screened me and the other one who came; whose number is written here, is the one who assisted me. He told me that “The things [cancer swellings] are present, but they will be cleared today.” So s/he showed me an equipment saying “This is the instrument that we are going to use; we will burn the first, the second and the third. Do you agree that we should burn?” I said “I agree” and s/he said “Okay.” Then they took a torch saying that “because there is no electricity here, we have this torch that we should use to light the cervix.” I said “Okay.” Then s/he took the torch to light and started burning; s/he burnt the first and asked me; “Do you feel pain?” I said “I am not feeling any pain but I am just feeling cold…” S/he said “Okay.” S/he burnt the second one “Have you felt pain?” I said “No I haven’t felt pain.” She burnt the third one and said “Have you felt pain?” I said “I haven’t felt pain.” Then s/he rubbed me with medicine and took a bottle and said “We should also have your urine specimen so that we test at the clinic. You will hear the results of the tests on the day that we will schedule you for the next visit.” I said “Okay, fine I can have time.” They said “Okay, fine.” Then we went to the last table and they told us that “You should abstain sex for six weeks from now. You should come to the clinic on 31, find us so that we give you results of the specimen we have taken.” I said “Fine.” “Are you married?” I said “We are on separation.”

I: Apart from the urine, what other specimen did they take?

R: I am not sure maybe they took something from inside the uterus because the urine was tested while I was there and they saw that I was not pregnant and I told them that “I am on permanent contraception, I don’t know maybe because some people become pregnant after permanent contraception, but I had Tubal Ligation.” They said “Okay, fine.” But the time when they were wiping on the sores, it was when they collected things; I don’t know maybe it was urine so that they should test here.

I: Where did they collect it from?

R: They inserted something like a pen here [pointing the vagina] and pumped something and poured in a bottle. They said “This is enough” and I said “Okay, fine.” Then they documented; “What is your name?” and I mentioned. And they said “When you go to the clinic, firstly you should have the results of the specimen we have taken to see what problem you have in your body.” So when I came here, they said “The specimen that was tested here shows that the burning that was done destroyed the infection and you have no any infection. So tell us how are you feeling now?” I told them that “I am now feeling well. The time when they were burning I did not feel pain but the past days I have been feeling pain… maybe discharging some things like fluid” as we were told that time. It was like vaginal discharge but was smelling like blood. They gave me a phone number that “If you experience something call this number and I called because at some point it was like as if I want to start having menses. They said “You should not be worried, that is the healing of the sores.” I said “Okay fine.” So I stayed and I was noting gradual improvement like from Monday to Sunday of the previous week, I could differentiate how I was the other week. The following week, I could see greater improvement up to the time when I came here.

I: Okay… Sure. So what are your thoughts about cervical cancer screening campaigns like this one where you got screened?

R: I can tell my friends that “Screening for cervical cancer is good because like what the doctor explained to us, the disease [cancer] is not painful but inside you, the organs are being destroyed. So I can explain clearly to my friends so that they should also get help.

I: Okay. So why did you decide to take part in this study?

R: I saw that I was found with the problem [cancer], so I thought I should accept so that I live on.

I: Okay. When did you join the study?

R: I joined the period when they doing the screening. I think it was on 17th, when we were screened. Like on 17th of (Month).

I: When you were being screened, were aware of your cervical cancer status?

R: No.

I: You did not know.

R: I did not know.

I: What influenced you to join the study when you did not know about your cervical cancer status?

R: I did that because the disease [cancer] comes unexpectedly. Like when I had an abortion, I was right here at Central [KCH] in upstairs; after abortion, I was told to go to theatre for a D&C procedure after miscarriage. So I was sent back for one week because they were saying that many women were undergoing uterus removal procedures because they had cancer of the cervix. Yes, and that was why I was quick to accept because it is better if they can remove the uterus and you live on. Yes, that was why accepted to be screened so that if I am found with a problem, I should be assisted and live.

I: Okay. Anything else that influenced you?

R: No, nothing.

I: Okay. Fine. Was there anything you were worried about before the screening?

R: Before I was screened and found that I had developed the disease[cancer]?

I: Yes…

R: No.

I: Okay…

R: But the problem was what I explained to the doctor that “I feel itching like what we call candida, I feel itching here [in the vagina].” And they said “But with this [cancer of the cervix], you don’t feel itching. The itching problem requires another medication.” Yes.

I: Okay.

R: This is what they told me.

I: Okay, thank you very much. So what did you hear about the screening?

R: When they were screening, I did not feel pain.

I: In terms of what people were saying…

R: Okay, what people were saying?

I: Yes.

R: Some people were saying that “It is said that they are collecting vaginal discharge, then they are taking blood…” But I was able to refute that “To say the truth, I never saw that they were collecting vaginal discharge from a person but we were putting urine in a bottle so that they test if you are pregnant or not because when you are pregnant, they don’t examine the cervix.” Yes, I was telling them like that.

I: Okay.

R: I was telling them like that… “No that is a lie, they say they are selling vaginal discharge at the clinic” but I was able to refute to the extent of arguing with my friends.

I: Okay. So when you heard that the results of your cervical cancer screening was abnormal, how did it make you feel? How did you feel in your heart?

R: When they screened me?

I: When they found that your results showed that you had small swellings that can develop into cervical cancer? How did you feel?

R: To say the truth, that time my heart was pumping very fast. When it reached a point when every doctor was coming to test me, I was shivering all over my body because I had fear considering the nature of the disease; when it has affected people in the community, it is a very dangerous disease. So I was so afraid that I cannot hide; I was shivering very much because I slept like this [demonstrates how she slept] but touching my knees, everywhere I was shivering.

I: Okay…

R: Yes, but the doctors encouraged me that “Don’t lose hope, with what we have done here, it [the disease] is gone.” Even at home, I could find myself shivering until when I had to pray when I was regaining my strength until when I noticed that things were improving.

I: What was your understanding of what your results meant?

R: I understood because they emphasized that when the disease has just started, we are able to burn off the cancer cells and that means the cancer is treated. When we see that the disease has progressed, we are able to do an operation and remove the uterus. So I saw that it was better because even if they can remove the uterus, you still have your life. Yes, that was when I accepted that I have to be screened. Because they say that you can find yourself dying but without any signs, like maybe painful abdomen or anything in the vagina, you don’t feel pain. So it is better to get screened so that you know your future.

I: Okay. So when this was done, what do you think was done well?

R: [No response].

I: During the screening what did you appreciate that “This has been done well”?

R: When they were burning off, I did not feel pain but when they inserted an instrument to pull the cervix so that they can be inspecting it; I felt a slight pain.

I: Okay. Was there anything that you think was not done well?

R: No, this was the only part.

I: In terms of the place where they were providing the service; how did you perceive it?

R: The place where I was screened?

I: Yes…

R: It was good.

I: Was it private?

R: Yes because it was like the way we are here, it was closed and even my friends asked “You have stayed long?” I just said “They said they were not seeing properly.” I just said it that way; I did not say anything.

I: How about the time when they were screening you?

R: Time?

I: Yes…

R: It took a long time maybe close to one hour. Because it took time; one came and said “I am not seeing properly” and went. So the instrument that held the cervix, it was painful.

I: Okay…

R: Yes.

I: Fine. What do you think could have been done better so that you don’t feel any pain? How could they have done it?

R: For me not to feel pain, maybe if the last doctor was the one who screened me at the beginning, he would have seen that the disease started and burn off right away, I would not feel pain, but I went with a female doctor who said “I don’t see well,” came another doctor, said “I don’t see well” and called another doctor; when s/he cane s/he said “Call this doctor. In that way time was going.

I: Okay. How was the explanation by the health providers?

R: The explanation?

I: Yes…

R: It was good, and a person was encouraged and was happy. I was happy so that I know my status.

I: How was their explanation in terms of telling you that you had cancer cells that made you fear and shiver?

R: The explanation was good but for me to shiver it was because I thought “The way that person died, it means I will also die and leave my children.” So I started shivering very much. To say the truth this issue is what raised my BP. “ I will leave my children; who is going to raise my children?” So the same time I found myself shivering very much.

I: How were they educating about cervical cancer?

R: They were educating very well and a person was free to ask questions or to give answers, and I was one of the people who was asking questions and encouraging my friends because people were refusing, “Don’t go there”, so I was one of the people who influenced the people to come. Encouraging my friends that “Let us go for screening; nowadays there are different diseases.” Yes.

I: Okay. How was the issue of partner involvement?

R: Partner involvement?

I: Yes, partner involvement like that your partner should be taking part or that male partners should be involved; what role did they play to ensure partner involvement during the education talk.

R: Ah, in terms of male partners, they were not there; we were the only ones. Other men were devaluing the exercise, and some were having conflicts in their families. Yes, but I did this.

I: Why were they having conflicts?

R: Because they were saying that doctors get semen when we come here at the clinic, so some were having conflict because of that.

I: Mh…

R: Yes, so that time I was alone. My husband left in January and I have spent the whole year alone.

I: Okay, fine. So what was the easiest part of this activity?

R: The easiest part?

I: Yes…

R: It was the education that they were giving us before the screening exercise. So for some, when they had answered the questions, they were afraid and were leaving.

I: What was the most difficult part?

R: Nothing.

I: Nothing?

R: Nothing, because the difficult part that I saw was when the doctor was saying that it was not clear to her that I had the cancer cells, and she called another one. That was when I had difficulties because it took more time. That is the only part.

I: You said you were seen by how many doctors?

R: Four. There was a male doctor; short, stout and brown in complexion. And there was a woman, another woman, and the doctor who gave me the phone number; stout, dark in complexion and tall. He was putting on a jacket that time, and up to now I do communicate with him on a phone.

I: Was there anything you did not expect it could happen?

R: Yes. I thought I would die. When I was discharging, I thought I would die and there was my sister whom I asked “Am I going to recover? Because this has never happened to me” So my sister said “God guides in everything. You should just be praying because if the doctor said that this was treated after the burning, be strong.” So at times I was worried, but at times I was strong thinking that “The way they told me, I was really treated.” So I live up to now, I am strong.

I: Okay. So, it can be hard for people to come for follow-up, did you have any challenges coming for this follow-up visit?

R: No.

I: Why not?

R: For me to come they told me that “You should come so that you should get your results and that you should know that you are feeling well or not. Are the sores healing or they are not healing?” Yes, that is how they told me to come here and when I came they explained that “You are now healed; if you have a husband, you can continue having sex.” Yes, sure.

I: Okay, fine. So what challenges do you think other women have that can hinder them from coming for follow-up visit?

R: The challenges are that in the community, we can have the misconceptions and gossiping each other like “This one is doing this”; in so doing, a person gives up.

I: What if there is no gossiping, what challenges can hinder a woman to come here?

R: Nothing, because if she comes here at the clinic, she is doing good for her health because you as doctors you can tell her whatever health problem she can have. You can explain to her and help her with solutions to prolong her life.

I: By challenges, I mean sometimes a person may strongly be willing to go, but because of other challenges, she finds herself fail to go. So I want such challenges.

R: Okay; those challenges can be like if there is a funeral; a funeral of a very close relative; so it can be difficult to come here. Secondly, money; because where we are coming from is very far and to travel from there...

I: Where do you come from?

R: I come from (Name of place). So to travel from (Name of place) to (Name of place), if you are to travel by motor cycle it is K1,000. So maybe you don’t have K1,000; like nowadays we have to be buying food; so you have no food and to find transport to come here... “How do i travel to that place? I really wanted to go but how do I travel?” So you find yourself failing to come. It is your scheduled date, but you find that the person has failed to come. The challenges can be funeral or lack of transport.

I: Okay. So how do you think we can best help women to overcome these challenges?

R: [No response].

I: What can we do to help women so that they dont miss their follow up visits?

R: Maybe if you can be telling the women as I have come today to say “Your next visit date is this; we should meet at such, such a place.” So if the person does not make it on the agreed place, it means she has a problem because they told us that “It is possible to pick people from their homes to the clinic and after procedures we bring them back.” Because like in my case, because a person maintains her own privacy; the time when they came, they picked women brought them here and took them back to the place where they picked them. When I was asked; “Your fellow women went, didn’t you get tested?” I said “I did not get tested.” They said “Okay, fine. Your friends went and they received various items” I said “Is that so?” “Yes, there is something that they sell there.” I said “No, that is not true. A person cannot sell things to the hospital.” They said “Okay, you must have tested already, how do you know?” I said “The one telling you was not saying the truth. As you are speaking here, you cannot know that you have a problem in your body; at the end of the day you find yourself sick not knowing that you had a problem in your body.” Sure.

I: Apart from coming to pick you from places that are closer to your homes, what else can we do to help you overcome the challenge of missing scheduled visits?

R: It is difficult to answer because these are difficult times, because we can say that there should be someone like a chairperson where you should go to leave money, but justice is a problem, and you may think I did not help you.

I: Fine. Now i want us to discuss about the support that partners and the community may provide; did you discuss about screening with anyone else? You said that you were encouraging people that “Let us go, it is good to get screened...” Which group of people did you tell this?

R: I was explaining to people from my community because some people did not go for screening. Because we just got screened and things worked, but other people who did not go for screening, they did not know about it. So that was when I was telling them that “It is good to get screened because you never know that you have a problem but when you get screened they tell you that you have this problem and when you are screened the problem ends.”

I: Specifically who were these people?

R: People from my community like, I have my other mother who is sick....

I: Like relatives?

R: Yes, I was able to explain to them.

I: Others?

R: Others like those who just came to the community to get married, I was able to explain to them.

I: How about your partner?

R: I explained to him because after we had separated, I think he came on 11 August. So when he came, I told him that “Although you have come that we should settle a family dispute, I have a problem; I went to the clinic. So your coming means that you have an objective to achieve, you have not come that I should be cooking food for you, but you need me. So with your coming, there is nothing that can be done because I am sick.” He asked “What are you suffering from?” I said “Doctors are moving everywhere screening for cervical cancer, and women tend to suffer from cancer.” He said “So you were screened?” I said “Yes. The cancer is screened on the cervix, and this means they check for cancer cells on the cervix, and it is not possible to have sex.” “So when shall we start?” I said “Until 31st when i go back to the clinic. That is when they will tell me to start or not.” “Okay, fine. I will wait.” So he waited up to 29th, 30th and 31st when he gave me transport. I came here. And I told him “You should escort me we should go together” but he refused saying “That is for women.” When I came here I explained, and I also told them about the problem that my husband has, and they said “When coming again on 12th, you should be together.”

I: What problem did you explain?

R: I wedded with him in (year). In (Year) he married another woman. In (Year) we started having conflicts; he became impotent; he could get an erection, but when he wants to penetrate, the penis loses the erection. I asked him, because he was working at a certain lodge, that “Maybe someone cheated you to use herbal medicine,” he said “No.” He tried but it failed. “Maybe this problem is coming because you had Tubal Ligation...”

I: At first it was normal...

R: It was normal, and he was troublesome; maybe we could have sex five or six times. We have been doing it like this for all my six deliveries, including the miscarriage. Since he is a guard, we could do four times during the day but nowadays we just see each other. I can start telling him that “I need you,” But he says “Where do i start from?” “What is the problem?” “Maybe touch me.” So I told him that “Maybe we should go to the hospital, it will not help us if we start going to witch doctors. There at the hospital, if they fail, they tell you that you should consult witch doctors. So we should go to the hospital.” He said “No, I don’t want to. The doctors will be laughing at me.” I said “They cannot laugh at you but they will give you treatment.” So this problem exists to date. So I was explaining to them when I came here that “Maybe I have been found with this problem because of my husband.” Because when he tries to do it, it takes him up to one hour trying to ejaculate but he fails. So you find that I feel pain in the vagina because he took time doing sex but failed to ejaculate. He says “I have failed.” So it happens that we do have conflicts; maybe he goes to his home village maybe up to one year without getting married. He says “Another woman will be publicising my problem. So let us be staying, you are my mother.” So we just stay without doing anything. Even if i may try to seduce him in any way, he doesn’t react. He says “There is nothing I feel.” So I explained to the doctor. I told him “Let us go”. He said “I have no transport. Did you not say many things there? Were you talking to a female or male doctor?” I said “she is my friend and when we go, you will be with a male doctor, and I will not be there.” He said “I have no transport.” Sure.

I: So what does he think about your cancer screening?

R: He just said “If you are saying that when you were screened you were found with cancer cells and that you had thermocoagulation treatment, I am happy.”

I: Was he interested to learn more from you?

R: He asked me; “What happened for you to go there?” I said, that doctors came not only here but also at (Name of place), (Name of place), so they were given the whole (Name of place), area. So they came here at (Name of place), and from here they went to (Name of place),. In other areas, people wish they had gone, but we are lucky they came here.” “Okay, fine. No, continue.” That was all.

I: What are his thoughts about coming to the clinic here to learn more?

R: I don’t know what he can think.

I: He did not say anything?

R: No.

I: Okay. So you have said that you discussed your results, right?

R: Yes.

I: Why did you discuss your results with him?

R: Love; and I knew it was because of love, so I decided to tell him what was on my mind. That was when I explained to him.

I: Okay. So you have said that he gave you transport to come to the clinic here; what other support has he been providing since you explained to him that you were found with cancer cells?

R: The other support was that he was buying relish for us to eat at home, soap. There was not any problem because he was saying that “we should wait until you go to the hospital.”

I: Okay. After thermocoagulation treatment, we advise that you should not be having sex for 1 month to allow for healing of the site.

R: Yes.

I: Was this a challenge for you?

R: No, nothing.

I: How supportive was your husband?

R: He accepted saying that it is the same as when I have delivered; he is able to wait for up to two months, sometimes one and half months; so he said he accepted it.

I: Okay, fine. So how do you think male partners should be more involved with cervical cancer screening for women?

R: I don’t know how he should take part because I already explained to him.

I: I am referring to male partners in general; how do you think they should be more involved with cervical cancer screening for women?

R: I don’t know if they can take part because men are harsh.

I: I simply want to hear your opinions for future care; how do you think men should be more involved with cervical cancer screening for women?

R: Maybe if you can be coming to the communities to educate them, and they can have the information and remember that you told them unlike if they hear it from a person like me; they cannot believe it. But if you can be coming to explain to them, some of them they can understand and take part.

I: In your opinion, do you think it is important that men should take part?

R: Yes.

I: Why?

R: Because as I have explained that my husband has a problem, so maybe if he can come here he can be assisted so that problem cannot only be with my husband, maybe other men also have the same problem because these things are confidential because they cannot be publicising; it means they are not respecting themselves.

I: How about men who might not have that problem; how important is it that they should be involved when their female partners are screening for cervical cancer?

R: It is important because even if they don’t have a problem now, but maybe in future they might have that problem.

I: Like what problem?

R: What?

I: Like what problem?

R: A problem like what my husband has, or their female partner’s problem, or a relative like a sister can be sick; so if they don’t have the information, they cannot be able to tell the sister that “You are supposed to go to the clinic for cervical cancer screening.” Sure.

I: So you have said that we should be coming to educate the men; what else do you think we should do so that we encourage men to take part in cervical cancer screening?

R: [No response].

I: Apart from educating them?

R: I don’t know what we can do.

I: Probably how do you think we can educate them about cervical cancer in women?

R: [No response].

I: How can we educate them?

R: You can know how to educate them as doctors; you can tell them that “You don’t come for cervical cancer screening of your female partners but you may not know that she has a problem. You may have sex with her not knowing that she has cancer cells.” You can be explaining to them signs and symptoms of cervical cancer and what happens when they are having sex with women with cervical cancer cells. So they can be afraid to say “If I have sex with a woman with cancer cells, does it not mean that I can also suffer? Oh, it is important to take part.” I feel that way.

I: Okay. Anything else?

R: No.

I: Fine. Now i want to ask about your knowledge and risk assessment. Is there anything new you have learned about cervical cancer or cervical cancer screening that you did not know before the study?

R: No.

I: You knew everything?

R: I did not know.

I: You did not know; what was new that you did not know before you were screened?

R: It was the cervical cancer cells that I was found with. It was something I never knew, but I knew because I had been screened. Had it been that I did not get screened, I would have just been staying without knowing anything.

I: What else about cervical cancer did you know at this time?

R: Nothing.

I: Is that all?

R: Yes.

I: Okay.

R: The only problem I had was that I had candida but that also started after I had stopped giving birth. When I was giving birth, I was not feeling itching, but after I had stopped giving birth, it was when I started feeling itching, and I said “In some way, my husband has infected me.” So when I showed my aunt at home, she said “it is not a sexually transmitted infection but candida (I think she is referring to genital warts). So she told me that “Go and buy a razor blade, we do cut them (I think she Mean warts)” when it was cut, it was more painful than before then it was cut again the following day and i was feeling hot and itching.

I: How about the decision that you should get screened; was it something you were thinking about before?

R: No.

I: How about the decision that you should get treatment.

R: The treatment?

I: Yes...

R: No, it happened the same day, but it was not something that I had thought about.

I: Okay. Who do you think should be screened for cervical cancer?

R: A woman.

I: What type of woman?

R: A woman who is not expecting and who is not in menses. When she is having menses, she is not screened.

I: How old should she be?

R: From 35 up to 50 years.

I: Okay. How about those who are HIV positive?

R: I have forgotten.

I: Why is it that a pregnant woman should not get screened?

R: Because they screen the cervix, and they cannot do the procedure because there is a fetus in the uterus and the woman can miscarry.

I: Okay. Now I want to hear your recommendations for future screening campaigns; What do women in your community think about cervical cancer screening?

R: They think it is good because at the school where the screening was conducted in our area, many women got screening, including teachers. Some were even traveling from our area to (Name of place), on motor bikes after they had realized that our friends received the necessary care.

I: How is the issue of discrimination?

R:Discriminating that we should not have sex?

I: No, discriminating women who were screened?

R: No, there isn’t.

I: Why do you think there is no discrimination?

R: Maybe because they don’t know that our friends got screened and that they are not knowledgeable.

I: Do you think these women have enough knowledge about cervical cancer screening?

R: They are not knowledgeable, because if they were knowledgeable, they would not be saying that “Those people who came were collecting vaginal discharge, and they were collecting from those who had sex. They collect the discharge, they say they sell it at (name of hospital)”, and I was telling them that “That is not true because I am one of those who got screened; they did not collect the vaginal discharge. The one who saw did not see the correct thing; they gave us bottles so that we can put urine specimen for testing pregnancy. If you are found positive in pregnancy testing, they do not screen for cancer because pregnant women are not screened for cancer.” Oh, okay... people were saying that they were collecting vaginal discharge.” “No, that is a lie.”

I: What do they think about their risk of having cervical cancer?

R: No, I cannot know what they think.

I: Perhaps do they think that they are not at a risk of having cervical cancer?

R: They think they are not at risk because they have not been screened, but if they were thinking that they are at risk, they would not have just stayed without getting screened.

I: Do you think women in your community understand the importance of cervical cancer screening?

R: Yes, mainly those who got screened and when we meet we discuss that “When those doctors come, there is need to urgently go for screening because we may just stay without knowing that cancer has started and the advantage is that when they come they say that when they find cancer cells, they burn them.” So it is important to be diligent when they come. And it happened that in our community there were some who had newborn babies, and they insisted that they should be screened and many were screened.

I: Okay. In your opinion, do you think that women are interested in receiving this screening and treatment service?

R: Yes.

I: What makes you think that way?

R: Because like the way I got screened, I knew it by myself, but I have seen the benefits and women do come for screening. [Interruption; someone opened the door and the recorder was paused.]

I: So we were discussing about how interested are women in your area to get screened and receive treatment.

R: Yes.

I: So you said they are interested because they are able to come right?

R: Yes.

I: Okay. So why would a woman want to be screened for cervical cancer?

R: Because they said that this is a sexually transmitted infection. The other reason that was said was that if you got married earlier than 20 years, that problem can develop but the disease is sexually transmitted.

I: So apart from getting married earlier than 20 years, what other factors can make them get screened?

R:The other factors are that because this disease is not sexually transmitted, it can affect any person, even those who got married when they were older than 20 years. Like in my case, I got married at 20, and I had my first born child at 21, but I was found to have the disease. Meaning that I got married when I was older. There are some who get married at 14, 15 some 16, and you find they have started having sex.

I: What can make them not to get screened?

R: Because of lack of willingness to go to the hospital.

I: What else?

R: Not trusting the medical care because of lack of knowledge that “Is there care at the hospital or not?” So they are not screened.

I: What else?

R: Misconceptions.

I: What misconceptions?

R: That they collect vaginal fluid and that it makes the woman weak and if you become pregnant you miscarry.

I: Okay. What other reason may make them not want to be screened?

R: Fear.

I: What can they fear?

R: [Chuckles]... Just fear.

I: Just fear with no reason for fear.

R: Because they have opened the cervix, because they say that they take out the cervix and start cutting the uterus, but like in my case I had permanent contraception, and I went there, but I did not see them cutting the uterus. What they look for is something else that doesn’t involve the uterus. Yes, so because they have not gone there, they talk about other things when the doctors want to help them.

I: Anything else that may make them fear to get screened?

R: That is all.

I: Okay. So, what are the barriers that some women may face in receiving cervical cancer screening?

R: Barriers?

I: Yes.

R: Just like i said, maybe one may be very willing to have cervical cancer screening but maybe she has lost her close relative or her child has died; so it is difficult; that time if you can come, she cannot get screened.

I: Other barriers?

R: No. Maybe the misconceptions; “Where are you going? Come back, they do this...” when she has never gone there.

I: Anything else concerning married women?

R: No.

I: The male partner cannot hinder the woman from going for screening?

R: Maybe telling her that “If you go there, when you will be coming back, our marriage has ended because there they will collect the semen that i give you.” This may make someone who was ready to go fail because of lack of knowledge of the husband.

I: Fine. So, in your opinion, do you think that women are interested in receiving this screening and treatment service?

R: My opinion is that they denied the right of some women who knew later about the service because they said that after this exercise, they will screen next year or so, I have just forgotten. It is difficult for them to get screened. So if you can be coming every two months and see if people are coming because they cannot manage to come here from the community but to go to the communities after maybe four months like how the family planning team does. So if you can be going every month or every two months or every four months; you will find that everyone has gone for screening. Because this thing is new; there are still fears, misconceptions, rumors so there is need to be coming to the communities because those who did not get the service will appreciate that they were supposed to get it. “I did not go, I should go.”

I: How can you encourage women to come?

R: I can encourage them because I got screened, and I was found with the problem. I can tell them that “Go for screening; rush.” In our community many people died; I have my brother in-law who married my sister; his wife died. So he was saying that “My wife would not have died but because there was no screening for cervical cancer in those days. There was no removal of the uterus.” It is said that his wife died in 2003. It’s long ago. “If it was now, she would not have died.” That is what my in-law says. I say “It is true, doctors are treating the disease nowadays.” “They are treating?” I say “Yes.” So he asked “If the woman has been found with cervical cancer and there is nothing they can do but to remove the uterus; can the woman be sweet [during sex] or you will never have sex?” So I just said “She will be normal.” I just answered like that. Sure.

I: Fine. Now i want to ask you about self testing; We will discuss about self-collected vaginal swab for cervical cancer screening. A new method has been developed for cervical cancer screening. It involves having a woman collect a swab from her vagina and submitting it at her convenience to a health facility like here right?

R: Yes.

I: Yah, for testing. However, unlike VIA, the woman would not get her result immediately and would have to return to health facility to get her result a few hours later or the next day.

R: That...?

I: That a woman should be collecting a swab from her vagina and take the specimen she has collected to the health facility where she should wait for results or she should come the next day for results. What do you think?

R: That is also a good idea.

I: What makes you think that way?

R: Because for people who think something is collected, using this method can make the people stop talking about it. They will be saying that “Can the doctors just use something from the swab?”

I: What else?

R: The other thing that makes it a good method is that the numbers can increase because they can only be receiving the swabs. The numbers can increase because this time they are afraid that “They insert instruments... the collect things...” Just thinking about something they have never seen because someone who has seen something happening does not say anything but for someone who has just heard about something, s/he is the one who goes around spreading the rumors.

I: What is the other advantage of this method?

R: The other thing is that there is need for you to come and explain to people because they have to use a swab so where will they be getting the cotton wool? There is need for you doctors to come and explain because you will be describing two methods; there are two methods; one is the VIA and the other one we provide so much cotton wool so that a person can self test by collecting a swab from the vagina. “A person should collect the swab, give us and wait for the results...” Because it is the same as HIV testing; we are drawn blood and we are told to wait for results; within some time we are given the results.

I: What do you think are the disadvantages of this method?

R: Nothing.

I: Okay. What do you think about collecting the swabs at home?

R: At home?

I: Yes.

R: No, because like at our home there is no electricity.

I: When you are collecting the swab, are you supposed to do it from your home?

R: No but from the clinic here; like the time I came, you give me the cotton wool and you tell me to collect the swab. Within a short time I collect and give you; you tell me “Wait there for some minutes” I wait. Then you call me, “Come and collect your results” when I come you ask me; “Would you like us to do this?” Then I should say “Yes.”

I: Do you think this is a reliable method?

R: Yes it is reliable but the challenge is transport because my home is far. So if you can do as they did coming to our community at (Name of place), or you can camp at (Name of place), or any nearby place, it can be possible.

I: Okay, fine. So how can you compare the self testing method and the method that you went through?

R: [No response].

I: How can you differentiate the two methods; the one that you went through and the self testing one?

R: We can differentiate; as in my case, I can say that I felt pain because I was screened for a long time so that they should be certain. For this other method, maybe it is good, but maybe it also has problems.

I: What could be the problem?

R: There are some people who have been going to the hospital since they started giving birth but there are some who just stay at home. So for these ones, they don’t know that you can insert a swab in the vagina. What they know is herbal medicine; when they are in labor, they take herbal medicine, after giving birth they take herbal medicine; they position you upside down after giving birth and pour herbal stuff in your vagina. That is what women in the village do. So such people may not know what a swab is. But for people like me, like there was the other time when I delivered at the hospital, I was taken to theatre, and from there I found myself with a cotton wool on my vagina, but after removing it there was no any problem. So for someone who delivers at home, she may not know cotton wool.

I: Which method would you prefer between the VIA and that you should be collecting a swab on your own?

R: As for me, I can say the same method that I went through because as I am saying, we people are different. There are some people who even fail to clean the vagina; they cannot insert inside. So there is need to insert. So if you don’t know how to insert in the vagina and yet you are supposed to insert a swab to collect the discharge, will you successfully collect it? If you cannot manage to insert and collect the discharge because you are afraid to insert inside? But for people like us who go to the hospital and we do insert here, we can manage.

I: So you said that you can prefer the VIA method...

R: Yes.

I: So I am asking why is that?

R: The doctor will do it with expertise.

I: Fine. What do you think other women in your community would think about the self-collected vaginal swab technique for screening?

R: [No response].

I: What can they think?

R: There I may not know what they can think about.

I: What can be your thoughts?

R: I can say that I should see if I have the cancer cells or not.

I: Okay.

R: That is my opinion, like the way I joined the screening crew, because I wanted to know if I had the cancer cells or not; so I accepted that I should be screened.

I: Do you think many women can choose the self-testing method to screen for cervical cancer?

R: Some can choose the self-testing method, while some can choose the screening method done by doctors so that everything is done right there.

I: Okay. Why do you think some women can prefer the self-testing method?

R: Because as I have said, they deliver at home and because they fear doctors, they can say that “It is better I do it myself so that no one sees my vagina. I should see my vagina myself not the doctor.” Because as I have said, those people who deliver at home, they don’t know that they can be delivered by a doctor, but for someone who delivers at the hospital knows that “When you are delivered by a male doctor, this is what he does, when you are delivered by a female doctor, this is what she does...” We are not even ashamed but for someone who delivers at home; they say “Lean on me” this is said by her mother or grandmother but do you say “lean on me” here? They say “lie here.”

I: What difficulties would women face in self-collection technique? You explained that maybe some are afraid to insert in the vagina to reach...

R: The cervix...

I: What could be other challenges?

R: Other challenges could be failing to hold the cotton wool the way you have instructed them so some cotton wool can remain inside or they may fail to collect the discharge. So that can be a challenge.

I: What can be the challenge?

R: Because they can be saying that “Since I inserted in the vagina, I started feeling pain, cotton wool remained in my vagina for one week...” You see, that is a problem. It is unlike if you insert me cotton wool as a doctor, because you know how you do it, and you are sure it will all come out because you have the skill.

I: What other concerns do you have concerning women failing to collect the swab because they fail to insert in the vagina?

R: I am not able to answer that one.

I: Fine. What are some of the reasons, if any, why you think women may not want to self test?

R: As I have already said, there are some women who don’t insert in the vagina when they are cleaning it, they just clean on top and there are some women like the fat ones, they cannot insert a finger in the vagina because of their body mass; their hand is too short; if they insert here [in the vagina], they cannot reach the required place. Those are the reasons. Because some people don’t see their vagina, they just touch on top.

I: Okay. Why do you think women would prefer to go to a hospital for screening with medical providers?

R: [No response].

I: You said that some women would prefer to be seen by a health provider...

R: Yes.

I: Why?

R: Because they can be sure that the doctors have taken the required amount of specimen unlike if you collect yourself because you may not be sure if you have collected the required amount. So you can be confident that “It is the doctor who collected it, I just laid like this...” I can believe what the doctor has screened.

I: Fine. Now I want us to talk about your recommendations for the future of the National cervical cancer screening in Malawi: In your opinion, should MOH consider including self-collected vaginal swab for cervical cancer testing to the cervical cancer screening programme?

R: They should include it because a person chooses from two options; “I will choose this method, I will choose this one.” So there is need for a self-testing method and one that doctors should be doing the screening; this method should not stop because if screened by a doctor, you have the results immediately; “I have don’t have it, you peacefully go. I have it, you get the treatment right away.” But with self-testing, you will end up saying “Collect another swab, this is not enough.” She goes, when coming “Go collect again there is nothing here...” In that way you are spending more time unlike when it was done by a doctor. So there is need for both methods so that if the self-testing method does not work, you continue with this one.

I: Fine. Do you think this would make it easier for women to undergo screening?

R: Yes it can be easy.

I: What makes you think that way?

R: Because this service is very helpful, and I am not the only one who is appreciating the service; other people in the community say “This is a powerful intervention.” Because people are dying because they are not aware of how cancer starts. So like in the villages when we are sick, we rush to private clinics, and it is not easy to get screened for cancer at a private clinic. My first day of coming here, they said “When coming here you should call us”, and I called and they told me that “you should go to maternity but you should not go inside. When you are there you should call.” I called and they said “Go inside and meet any doctor, there s/he will tell you where you should go.” I went there in maternity, and they told me to go to MIM... there is MIM up there. They shouted at me; they shouted at me, and had it been that I reacted the way I did when I was found with cancer cells during screening, I would have come back crying, but because I had accepted it and made my mind that I have to receive treatment. They shouted at me saying “Do you have money that we can screen you for cervical cancer? The way you are looking do you have money? Where can you get money woman; get out of my site you are wasting my time.” I returned.....

I: Where do you say that is?

R: They say MIM.

I: MIM....? Right here at (Name of place),?

R: They said Ethel.

I: Oh, at Ethel?

R: Yes at Ethel; I found a doctor in white gown but s/he shouted at me. s/he took my health passport book and threw at me; I reasoned, got out of there and came somewhere around this corner... and I saw a man sitting on a table wearing something like this...[She was showing a green color on her wrapper], and I took the health passport book and showed her/him because I said “Even if I should be explaining that this is what happened to my cervix, but they don’t know me, and they will be asking “What happened?” I just showed him here and the man said “Oh, okay, go there where there is an open door. Give the book to anyone else, they will tell you where you should go.” That was when i arrived here.” A certain girl who tests blood told me that “Come here,” that was when i reached at that place. Sure.

I: Okay. So What groups of women could be most suitable for self-collected vaginal swab for cervical cancer testing?

R: They can be found; some will say “I will be screened by a doctor” some will say “I will self collect the vaginal swab.”

I: Mainly what groups of women do you think may want to have self-testing?

R:Those who go to the hospital because they know cotton wool and how to use it, but for someone who has never gone to the hospital may want to be screened by a doctor.

I: Perhaps can you tell me the ages of these women?

R: I may not be able to because maybe more women will choose self-testing or more would prefer to be screened by a doctor... I may not know the exact ages.

I: Fine. And what groups of women would not be suitable for self-testing?

R: Like those who are not able to insert inside [the vagina]. They just clean on top.

I: How do you know the groups who just clean on top?

R: They discuss when we are chatting; “To the extent of inserting? I cannot manage” “We do insert to clean it...” So you see that there are a lot of them who say that. You know when there is a funeral, we women we sleep in one room and we discuss issues and that is when we know that many women just clean on top.

I: Thank you very much. Maybe you can have addition to what we have discussed today?

R: My additional comment is that you doctors you should continue coming to areas where you did the screening so that our friends who want the service, should be screened because they are failing to come here because they just hear “It is at (Name of place),, but they don’t know the specific place; they have never been to (Name of place), so they cannot come here. So I feel if you can find a means that this time we should go back to screen those who did not, you will see that several people will come for screening.

I: Thank you very much. So I thank you so much especially because of your time and your information which is more important to this study. Maybe before we close, perhaps you might have other additional comments?

R: No, nothing.

I: Nothing?

R: Yes.

I: Your time is very vital; sorry that we have delayed you but your information is very important to this study. This marks the end of our discussion.

R: Okay.

I: Thank you.

R: Thanks.

End of interview.
