# Supplementary material for: Perceptions of cervical cancer and motivation for screening among women in Rural Lilongwe, Malawi: A qualitative study
Source: PLoS One. 2022 Feb 7;17(2):e0262590. doi: 10.1371/journal.pone.0262590 (PMC8820632; doi:10.1371/journal.pone.0262590)
Supplement: S3 File — (ZIP) [file pone.0262590.s003.zip › VIA_180 Missed.docx]

**PATICIPANT ID: VIA_180**

**INTERVIEWER ID: 748**

**Date of interview: 27 November 2017**

**Length: 56 Minutes**

**Transcriber: 748**

1. I: So thank you for meeting with me today. You have many things to do. I really appreciate your time and your input will be very helpful. I am working with a team of researchers from the University of North Carolina Project in Malawi….
2. *R: Okay…*
3. I: Sorry you were not able to come for your 12-week follow-up appointment. We would still like to hear from you regarding your experience with the cervical cancer screening with VIA and thermo-coagulation treatment. We would also like to hear about any difficulties you had afterwards or any challenges you had to coming for your follow-up visit. Your input is also important to help us understand how best we can conduct cervical cancer screening campaigns in Malawi. Did they tell you that we are conducting a study?
4. *R: Yes they told me that they want to see if we have the cancer cells so that they can give us treatment and refer us (Name of hospital).*
5. I: Yes. So we are conducting a study on cancer screening. So, as we will be discussing here, there is no right or wrong answer and everything you say will be confidential and only used to make this health program and health questionnaire better.
6. *R: Okay.*
7. I: Sure. So, I will audio record this interview to help me remember what was said, but your name or any identifiable information will not be connected to anything you say.
8. *R: Okay.*
9. I: So I am not expecting you to mention your name as we will be discussing here.
10. *R: Okay.*
11. I: Thank you. So to start with, can you tell me your understanding of the cervical cancer screening and treatment you received sometime back?
12. *R: I was screened but they did not tell me about any results as to whether they found me with cancer cells or not. They just told me to go and buy tablets from X trading centre.*
13. I: What did they say the pills were for?
14. *R: I don’t know. They just said I should be taking I think they said two tablets but I did not go to buy.*
15. I: So they just told you to go and buy some tablets but they did not tell you…?
16. *R: Yes and they even wrote the name of the medicine on the paper.*
17. I: But they did not say why they wanted you to go and buy the medicine?
18. *R: No.*
19. I: And you have said that you did not go to buy?
20. *R: No.*
21. I: Why?
22. *R: I did not have money. [Chuckles]… because we share the money… I told my partner to go and buy for me because he works there in town. So he did not buy because he said he did not have time to go to a shop to buy the medicine. I asked him; “Why can’t you use the lunch hour time?” He said “That is time I have to and look for food and at half one o’clock sharp, I have to go back to work.” So we failed to buy the drugs.*
23. I: Up to now?
24. *R: Yes, up to now. And my partner asked me “Are the drugs they want you to buy for the cervical cancer? Have they found you with cervical cancer?” I said no, they did not tell me that they had found me with cancer but they just said that if I want to know more, I should go to the hospital where they can explain to me. They also said that if they can find an opportunity, they can come to visit me here.*
25. I: So when they were screening you; what screening did you undergo and why did they say they were screening you?
26. *R: Screening?*
27. I: Yes.
28. *R: Because that time we went to register for the citizenship identity cards and they came there in a car and started sensitizing people about cervical cancer. So we said that was our opportunity to be screened.*
29. I: What did they say?
30. *R: They said that “We have come to conduct cervical cancer screening; if you can be found with cancer cells, there is a way we can treat you. We asked them: Suppose you have found us with cervical cancer cells; are you going to give us treatment right away?” They said; “There is treatment but you are supposed to go to Kamuzu Central. That is where you can find the standard care because here we don’t have enough equipment. Women you have this problem but you fail to come in an open because there is a misconception that we collect vaginal discharge.” So some were agreeing to say “It is true; I think in 2005 these people were coming to* (Name of place),  *to sensitize people and those who joined they were inviting them to the hospital but they were reimbursing their transport.” That is what I heard but I cannot cheat to say I have ever been screened for cervical cancer; this is my first time. It is true that there are misconceptions among women but that is not good because women fail to go to the clinic when they have the problem but because they don’t know, they just stay with it and by the time they will think about going to a health facility, the cancer has metastized.*
31. I: After that what happened?
32. *R: Then they called us, before screening they were telling us to go and pass urine. We were giving them the urine and they were testing it. After testing the urine, they were telling us that “everything is okay.” after that they were documenting the urine results. That was what I saw. From there they were telling us to go for HIV testing and after testing we were waiting somewhere for the results. Then they were giving us the results. So there were no any results that I was given about the cervical cancer screening apart from the HIV test results that I received. So after HIV testing, it was when I was screened for cervical cancer…*
33. I: How did they do it?
34. *R: They took a spanner and did like what you people do. Yes, she tested me like that. At first there was one doctor then s/he called another doctor and called a third doctor. There were like three doctors and after they had screened me, they told me to wake up. When I woke up, they did not tell me anything that I was found with cancer cells or not. They just gave me a form and told me to go to (Name of hospital).*
35. I: So the first doctor screened you with two spanners and called the second doctor…
36. *R: Yes…*
37. I: Did the second doctor screen you in the same way as the first doctor did?
38. *R: S/he did the same way. The only difference was that the second doctor used different spanners and not the same spanners that were used by the first doctor.*
39. I: Were the spanners similar or different?
40. *R: They were similar.*
41. I: What about the third one?
42. *R: The third one was just observing what the other two were doing.*
43. I: So after screening they did not tell you anything?
44. *R: They did not do anything. They just screened and let me go.*
45. I: Did they not explain to you what they were doing?
46. *R: No they did not explain to me.*
47. I: What did you think was the objective of the training?
48. *R: I thought after screening me, they would tell me that “We have found you with cervical cancer cells.” That was what I was expecting but I was not told anything. So I believed that I would hear everything from the hospital.*
49. I: So you thought you would hear everything from the hospital…
50. *R: Yes.*
51. I: But you did not go to the hospital…
52. *R: I did not go to the hospital. They came here and found that I was in the garden. When I came here, I found them and they told me that “We have come here as we came at* (Name of place), *…” I said “Yes… thank you.” And they gave me a date.*
53. I: They gave you on this day?
54. *R: Yes.*
55. I: When you were screened were you given a date?
56. *R: No they did not give me a date but I was given a date on this day when they came. So on the date when they said I should go, I failed because one of my children was sick and I did not make it to the hospital.*
57. I: So that was the first tracing visit…
58. *R: Yes that was in (month). They also came and said “We understand that you had problems last time that made you not to come to the hospital but this time we are asking you to come.” I said “I will try to come provided I should find transport.” He said “Your spouse should try to give you transport. You should only find transport to go to the hospital. We will refund your transport to and from the hospital.” I said “Okay, I will come and I will call you.” They even gave me their phone numbers. The challenge this time was that we had a funeral of a Group Village Head and I could not afford to leave. So I went to attend the funeral ceremony.*
59. I: Okay. So since that time, was there another visit when you were asked to go to the hospital and you failed?
60. *R: No. I can say that from January, my partner is on leave for three weeks and I was busy in the garden so that he can help me with farming activities.*
61. I: So you said that you went for cancer screening when you heard that the service providers had come in your area while you were registering for identity cards…
62. *R: Yes.*
63. I: Why did you decide to be screened and participate in this study?
64. *R: It was because we have been hearing a lot that women are at risk of cervical cancer and we wanted to be screened to see if we have the cancer cells or not. Like my young sister has been complaining about abdominal pains and people were saying that it was because she had Tubal Ligations. So they were saying that “Tubal Ligation is causing cervical cancer; it is better to go for other family planning methods.” They were also saying that sometimes you feel itching inside the cervix and because of how I have been feeling, I decided to go for screening.*
65. I: What was your problem?
66. *R: I was feeling itching on the cervix. I even told them when they were asking me questions. They also asked me; do you feel pain during sex? I said “No, I don’t feel pain during sex.*
67. I: Were you worried about anything when you were being screened?
68. *R: I did not have any worry because they told us that we should not think that they are taking our vaginal discharge; where are they going to take the vaginal discharge to? So I was not worried and I knew I was not going to have any problem.*
69. I: Did you hear anything about cervical cancer screening before this screening?
70. *R: Yes I heard about it from my mother. She has been going to (name of hospital) hospital and she has been telling people that “If people say that these people they collect vaginal discharges from women, that is just a misconception.” That is what my mother has been saying. They equipment they use is a spanner; it is not like a pipe which can collect vaginal discharge. If someone wants to know the truth about the screening, it is better to go to the hospital and see by yourselves.” This is what my mother has been saying. So I said let me go and see if what my mother was saying is true. In terms of Tubal Ligation I did it myself.*
71. I: You had Tubal Ligation?
72. *R: Yes…*
73. I: When people were telling you that it causes cancer?
74. *R: I did it because my partner forced me to have it and I had to do it.*
75. I: So how worried were you about getting cancer?
76. *R: I was worried but what else could I do when my partner had given me transport to hire a bicycle and go for Tubal Ligation? I just had to go.*
77. I: So you have said that you were not given the results…
78. *R: Yes…*
79. I: How did you feel when you were told to go to a shop and buy drugs and when you were given a form to go to (Name of hospital)?
80. *R: I was thinking that “Have they told me to go and buy drugs because they have found me with cancer cells or they think I can be telling other people that I have been found with cancer cells?” That was my worry.*
81. I: Did you try to ask the doctors what they had found?
82. *R: No, I did not ask any thing because when I came out of the room where they screened me, I went to a table where I gave them my health passport book. In there they prescribed the drugs and told me that “You should go and buy and they should be less than K200.00.” They told me that I should go and buy at* (Name of place),  *but if I find that they are not available there, I can go to pharmacy.*
83. I: So you have told me what happened on that day; you have arrived, they have tested you for HIV, screened you for cancer… what do you think was done well on this day?
84. *R: I did not find anything wrong because there are misconceptions in the communities. People talk a lot about something before they have had an experience of what they are talking about. So I went there to see it by myself.*
85. I: What were the misconceptions about this that were there?
86. *R: They say that when you go to a hospital like (Nam of hospital), they draw vaginal discharge and when they collect it, they give it to barren women so that they can have their own children. So I was asking; “Is it possible to collect vaginal discharge from another person and give a person who cannot give birth? Is that possible? If science has reached that extent of making a barren woman have children by using another woman’s vaginal discharge then it is a positive development.” I was telling them like that. “To have evidence of what happens there, it is better for us to go and see what actually happens.” I was telling them that.*
87. I: So among everything that happened on that day, what was the easiest part?
88. *R: The easiest part was the screening of the cervix.*
89. I: What happened with the screening?
90. *R: The easiest part was that when I came, I was told to put urine in a bottle and when they tested it they said there was no any problem. They just told me to go and buy drugs.*
91. I: What was the hardest part?
92. *R: The hardest part?*
93. I: Yes…
94. *R: The hardest part was what I have told you that after screening me for cervical cancer, they did not tell me that they had found me with cancer cells or not.*
95. I: Suppose that day you were given the results; we are just imagining; suppose they told you that you were found with cancer cells; would you have gone to the hospital if you were told to do so?
96. *R: I would have gone; even this time I would have gone had it been that I was not busy.*
97. I: So you would have gone if you were not busy?
98. *R: Yes, I would have gone.*
99. I: So you would have gone but this time you have been failing to come; what would you do to ensure that you come to the hospital?
100. *R: Like what he has been doing when he comes for tracing me, he leaves his phone number and the phone numbers of other study staff so that if I plan to come, I can call one of them.*
101. I: So what prevented you from calling the people so that you could come on another date?
102. *R: To say the truth, I did not think about calling because I had no money.*
103. I: So suppose some women were screened for cervical cancer and they were told to go to the hospital; what do you think would make these women fail to come to the hospital?
104. *R: After being screened?*
105. I: Yes… if they can be told to go to (name of hospital); what do you think can make them fail to come?
106. *R: It is just that we don’t take these things seriously because there was a certain woman who was screened at (name of village) and she was told to go to the hospital. When she failed on the date when she was scheduled to go, she was traced and the tracer gave her transport and scheduled her on another date. On that date, she did not go. When I asked her “Why have you not gone to the hospital and yet you were given transport?” She said “I have just decided not to go.” So it is because of our lack of seriousness that we don’t go to the hospital when told to do so. Of course sometimes it happens because of unforeseeable circumstances.*
107. I: What are those unforeseeable circumstances? I want to know.
108. *R: Sometimes there can be funeral or you have gone somewhere to nurse a patient or there is a community meeting where you have a role to play. If that happens, you cannot go to the hospital.*
109. I: So how do you think we can help women to address these problems?
110. *R: I don’t think you can be able to address these problems.*
111. I: Why?
112. *R: Because we people we are different; there are some people who are just not willing to come to the hospital and they would always have excuses when you come to trace them. Like my friend I was talking about; on daily basis she goes to* (Name of place),  *to sell ground nuts, pigeon peas, soya beans but she decided not to go to the hospital even after being given transport money.*
113. I: Meaning that the problem can be addressed by the person herself…?
114. *R: Yes, it is up to the individual to have a positive attitude about going to the hospital.*
115. I: Who did you discuss with about the cervical cancer screening?
116. *R: When I was coming from* (Name of place),  *I came with forms. So when other women in the community saw us they asked; Have they given you forms? I said “No, this is not about the identity cards; we went for cervical cancer screening.” “so they have taken your vaginal discharge?” “We did not see something like that; the best way is that you need to go and see it by yourself.” I have been thinking about going there but I am afraid of this…” My sister in-law is willing to go for cervical cancer screening but she fears what people talk about vaginal discharge collection.*
117. I: Who else apart from those?
118. *R: Those are the only ones.*
119. I: What about your partner?
120. *R: I told him on the same day when I came for screening and he asked “What have they found?” I said “They did not find me with any problem but I am supposed to go to the hospital.” He said “Okay, fine.”*
121. I: What does he think about cervical cancer screening?
122. *R: I cannot say that my partner thinks that I have cervical cancer because I told him what happened when I was screened. So he doesn’t know anything that I have cervical cancer.*
123. I: Of course you were not given the results but what does he think about cervical cancer screening itself?
124. *R: On that one, I don’t know what he thinks.*
125. I: Were you thermos-coagulated on this day?
126. *R: I am not sure.*
127. I: How did you feel during the screening?
128. *R: I felt slight pain and they put cotton wool on the vagina and they told me that “we are putting this cotton wool because you are bleeding.”*
129. I: How much was the blood flow?
130. *R: It was not as much as menses.*
131. I: Were you counselled in any way about sexual life?
132. *R: Yes, they said I should stay for seven days without sex.*
133. I: Seven days?
134. *R: I think so.*
135. I: Were they days, weeks or months?
136. *R: I think they said seven days.*
137. I: How comfortable were you to stay for that period without sex?
138. *R: When they told me I asked; “Is there another way that can be done should the partner insist that he needs sex?” They said “We want to protect you so just convince him to abstain from sex for that period.”*
139. I: How supportive was your partner on that?
140. *R: he was supportive and when I explained to him he did not argue.*
141. I: So do you think men should take part more in the cervical cancer screening of women?
142. *R: He must be involved more because my partner has multiple sexual partners and if I can have a problem of cervical cancer maybe it can be because of him. So I think if he can hear about this, maybe he can stop.*
143. I: You have said that if he can hear about this he can stop. Can you explain on this one?
144. *R: Yes I think so because we got married in (year) and our first born was born in (month and year) but it was found that while the baby was young he was going for other women. We had a conflict and he confessed that he will never do that but few years later he started having other sexual partners until 2011 when he married another woman. He did not have a child with that woman and their marriage ended after one year. After that, he started having other sexual partners. So I feel that if I can be found with a problem of cervical cancer, it can be because of his promiscuity.*
145. I: So you have said that if he can be involved in cervical cancer screening he can stop… how can he stop?
146. *R: He can stop if it can happen that I have been found with cervical cancer and after being treated, he can stick to me.*
147. I: So how do you think he can be involved?
148. *R: If he can be accompanying me to the hospital he can be present when I am being screened for cancer and we can be counselled together. In this way he can know what causes cervical cancer and how it can be prevented. Like the first time when the traces came, I asked if I could be accompanied by my partner to the hospital and he said “there is no problem you can come with him.”*
149. I: Did you plan to come with him when you promised to come the time you were traced for the first time?
150. *R: No I did not think about that because we were supposed to come during the week and it is difficult for him because of the nature of his work. He works for a certain foreigner in a construction company and there are few employees so it is difficult for him to excuse from work.*
151. I: So how can we encourage them to be involved because like for your partner you have said he is very busy and he cannot be able to come to the hospital during the week?
152. *R: Maybe as you have come like this, if you can meet him and tell him that you can do the screening on a Saturday or Sunday when he doesn’t go to work, I think that can help. Like if you had planned to come tomorrow maybe he would have been here and he would have heard whatever we are discussing here and probably he could be the one who would have given you the date when we could come to the hospital.*
153. I: Is there another way to encourage him?
154. *R: No that is the only way.*
155. I: So when you went for cervical cancer screening, was there anything new that you learned?
156. *R: No.*
157. I: Did you not learn about how cervical cancer is caused?
158. *R: No, the new thing was the itching.*
159. I: Did the itching start before you were screened or while you were being screened?
160. *R: They said that it happens that maybe you have been married by an older man or you got married while young; you can be feeling pain in the vagina and that sometimes may cause cervical cancer.*
161. I: So this was something new?
162. *R: Yes..*
163. I: What else?
164. *R: This was the only thing I heard.*
165. I: So who do you think should be screened for cervical cancer?
166. *R: It can be me.*
167. I: Why?
168. *R: Because a man cannot e screened for cervical cancer since he doesn’t have the cervix.*
169. I: Oh, by mentioning about you, you mean a woman?
170. *R: Yes… [Laugh]*
171. I: So have said a woman; there are different women; some are young, some old… what types of women are supposed to be screened for cervical cancer?
172. *R: I can say older women.*
173. I: What type of older women? Married… reproductive…?
174. *R: She can be of my age but not married. If she is willing, she can go for cervical cancer screening because a woman aged 50, 60 or 70 years cannot go for cervical cancer screening because she is too old. So the middle aged women are the ones who are supposed to be screened for cervical cancer. Women above the age of 50 are past the age to be screened but for someone like me, she may not be married but may not be willing to be screened.*
175. I: So how can you encourage such women to be screened for cervical cancer?
176. *R: I can tell her that if she has sex with men, she is at a risk of cervical cancer and she has to be screened.*
177. I: So how often should women be screened for cervical cancer?
178. *R: I think you are the ones to tell us because you know when could be the best time for the women to be screened.*
179. I: When you went for cervical cancer screening, what did they tell you?
180. *R: I think the problem was that I did not ask. If I am not mistaken, they said that if you have not been found with the cervical cancer cells, you are supposed to go again for screening after one year… I think so if I am not mistaken but the problem was that I did not ask.*
181. I: Did you agree with the time they mentioned?
182. *R: The one year waiting period?*
183. I: Yes…
184. *R: I can agree with it because you have seen it that it is the right time to be screened because even when you go for HIV testing, they give us time when we are supposed to go again for another test.*
185. I: So what do women in your community think about cervical cancer screening?
186. *R: It is just that women are not willing to be screened for cervical cancer. In this community, women are not willing to go to the hospital for cervical cancer screening.*
187. I: What else?
188. *R: The reason is what I have said that people think when they go there for cervical cancer screening, they are going to collect their vaginal discharge so because of that fear, the choose not to go.*
189. I: What else besides that fear?
190. *R: That is the main reason.*
191. I: Okay. do they know how cervical cancer is caused?
192. *R: That is the problem; if they can know how cervical cancer is caused maybe they can understand why they are supposed to go for cervical cancer screening but the problem is some of them they don’t know.*
193. I: Like for some who have heard about it, do they know the benefits of cervical cancer screening?
194. *R: Some of them may know the benefits but may just not be willing to go for screening.*
195. I: Why do you think they are not willing when they have the knowledge?
196. *R: Because they have not gone to the hospital and seen by themselves what happens, they are hesitant to go.*
197. I: They are hesitant to go…
198. *R: They are hesitant; like my sister in-law, she was saying that “My husband said if you go there for cervical cancer screening; if you can be screened by a man he can rape you.” So I told her “You are cheating; don’t you know that if you become pregnant you can be delivered by a man? Can a man rape you when you are delivering?” She said “No.” I asked “Why?” She said “Because I can be bleeding.” So I told her that “It is the same thing with cervical cancer screening; there is no way a male doctor can rape you when he is screening you for cervical cancer. Those people are professional and for your information; my first born, I was delivered by a man at (name of hospital). My second born I was delivered by a man at (name of hospital) but neither of them showed signs that they could rape me. So if you take what men are telling you, you will die while they live. They are not the ones who can suffer from cervical cancer. If they can be found with cancer maybe it can be cancer of the leg or other organs…” Even other women also told her that her partner does not care for her life because if people go to the hospital, they don’t expect to be seen by female doctors only.*
199. I: Do you think some women do not go for cervical cancer screening because they fear that they can be found with cervical cancer or they can be stigmatized… do you think they think like that?
200. *R: That can be one of the reasons; some may think that if they go for cervical cancer screening and if they can be found with cancer cells, their partners may tell them that they will leave them and marry another woman. So that could be the fear.*
201. I: So what are some of the barriers that women may face in receiving this service of cervical cancer screening?
202. *R: On that one, I have never hear about it or I have seen it happening.*
203. I: do you think some women may not go to the hospital for cervical cancer screening because their partners stop them from going there?
204. *R: That cannot happen because the problem is with the woman and you are supposed to go to the hospital for screening.*
205. I: So we want to involve more women in cervical cancer screening. How do you think cervical cancer screening should be provided to make sure that more women are involved?
206. *R: There should be some women who went to the hospital for cervical cancer screening who should be encouraging the other women to go to the hospital for screening. If they can see that their friends went and they received treatment without any problems, they can be encouraged to go.*
207. I: Okay. So we are going towards the end of the discussion don’t worry. Now, let us discuss about self-collected vaginal swab for cervical cancer screening. A new method has been developed for cervical cancer screening. It involves having a woman collect a swab from her vagina and submitting it at her convenience to a health facility for testing. However, unlike VIA, the method that was used to screen you, the woman would not get her result immediately and would have to return to health facility to get her result a few hours later or the next day. What do you think about this method?
208. *R: Does it mean that men will be involved in this method?*
209. I: Which men are you referring to? Your partners or the service providers?
210. *R: My partner…*
211. I: In this method, you are going to collect a swab from your vagina and you can put the swab in a plastic paper or on a cotton wool and keep it. When you have tie, you can take that swab to the hospital for screening. So what do you think about this method? In this case a man can be involved just by escorting you or giving you transport to go to the hospital. What do you think about tis method?
212. *R: It can be a good method.*
213. I: How?
214. *R: It means women can adhere to go to the hospital because they will be collecting by themselves. Because people have been afraid that they collect vaginal discharge and to them they thought they use a pipe to collect the vaginal discharge. So if they can be collecting themselves, that fear can no longer be there.*
215. I: Like in your case, can you be interested to be screened using this way?
216. *R: I can be interested.*
217. I: If you may be asked to choose between VIA and this one; which one can you prefer?
218. *R: I can prefer both.*
219. I: [Laugh]… What if you may be asked to choose one?
220. *R: Maybe because this is a new method, I can choose the old one.*
221. I: What difficulties do you think women would have with the self-collection technique?
222. *R: The old one?*
223. I: No the new one?
224. *R: I cannot say anything and about this method because it is just new.*
225. I: How reliable do you think this method is?
226. *R: This method is reliable because this problem is with us women and if we are given an opportunity to collect the swab on our own, it is better we collect it and take it to the hospital for screening so that we know our cervical cancer status.*
227. I: Okay. But do you think this method is reliable or not?
228. *R: I may say this method is reliable or not because you tell us not to douche but now you are telling us to insert a swab… Because at the clinic they tell us not to douche; they say that we should just clean on top and the vagina cannot smell bad.*
229. I: So you feel it is not reliable because you have to insert a swab when collecting?
230. *R: Yes because the way you can collect it is different from how we can collect it ourselves.*
231. I: Okay. What do you think other women in your community would think about the self-collected vaginal swab technique for screening?
232. *R: Ah, on that one, I can say that if we collect ourselves, the swab may dry. Also, there are some women who don’t discharge because of the family planning methods and it can be difficult for them. So they may still need you to collect the swabs for them.*
233. I: You have said that the swabs may dry…
234. *R: Yes…*
235. I: Where do you think is the best place to collect the swabs; at the hospital or at home?
236. *R: They should collect it right there at the hospital. You give them the swab and they should collect the swab the same time and give it to you.*
237. I: So based on what you have said, do you think they can be willing to self-collect the swabs or not?
238. *R: Firstly, you need to educate them. Tell them to go and urinate then give them the swab to collect the discharge. Tell them not to stay with it for one hour. Within few minutes they should bring the swabs so that you can take them for screening.*
239. I: You said you can prefer VIA. Which method do you think these women can prefer?
240. *R: I may not be able to say because they don’t know both methods but if they can be sensitized on both methods, I think they can prefer the new method.*
241. I: Why would they prefer the new method?
242. *R: Because they would collect by themselves.*
243. I: What difficulties would women face with self-collecting technique?
244. *R: I don’t see any problem with that. I feel that you decided to have these two methods so that for some people who may fear the instruments, they may use this method. You know people have fears; they even fear giving birth. Because people fear the first method because they insert instruments but with this one you have to collect the swab yourself. So they can like it. That is my opinion.*
245. I: Do you think some women may not want to collect the swab?
246. *R: No.*
247. I: They may all be willing to collect the swab?
248. *R: Yes. As I have already said that some people feel ashamed to deliver in the presence of other people because they don’t want them to see their private parts. So they may choose this new method where they have to collect the swabs on their own.*
249. I: Now let’s talk about your recommendations for the future of the National cervical cancer screening in Malawi… In your opinion, should Ministry Of Health consider including self-collected vaginal swab for cervical cancer testing to the cervical cancer screening programme?
250. *R: My opinion is that this method should continue.*
251. I: Which method should continue?
252. *R: We were saying that women were afraid of the first method because they thought that they collect their vaginal discharge. So with this self-collected method of the vaginal discharge, many women will be willing to use it and the government should consider including it as one method in cervical cancer screening.*
253. I: What type of women do you think should use this self-collecting method during cervical cancer screening?
254. *R: Women of my age group.*
255. I: When you say women of your age, what do you mean?
256. *R: Like 20, 30, 40 years… because nowadays women get married when they are not less than 20 years while in the past a girl of 18 years old could get married. However, if a woman less than 20 years can have some health problems, she may go for cervical cancer screening.*
257. I: What groups of women would not be suitable for cancer screening?
258. *R: They can be the ones I said from 50 years and above.*
259. I: Why could they not be suitable?
260. *R: Because they stopped giving birth and they are not productive.*
261. I: So we have talked many things today. Maybe you have questions or comments…?
262. *R: My comment is that I should try hard to go to the hospital so that I can have the first hand information of my status.*
263. I: Do you have any worries?
264. *R: No, I don’t have worries. If one can be found with cancer cells, is there a counseling service available?*
265. I: It should be there I am sure but the most important thing is that you should go early.
266. *R: Because maybe BP may rise because of many thoughts.*
267. I: Fine. Anything else?
268. *R: No.*
269. I: Thank you very much.
270. *R: Thank you.*

End of interview
