# Supplementary material for: Perceptions of cervical cancer and motivation for screening among women in Rural Lilongwe, Malawi: A qualitative study
Source: PLoS One. 2022 Feb 7;17(2):e0262590. doi: 10.1371/journal.pone.0262590 (PMC8820632; doi:10.1371/journal.pone.0262590)
Supplement: S3 File — (ZIP) [file pone.0262590.s003.zip › VIA_207 Missed.docx]

**PARTICIPANT ID: VIA 207**

**INTERVIEWER: 466**

**Date of interview: 16 Dec 2017**

1. I: So I would like to thank you very much for meeting me today, at this time, and your input will be very helpful. I am working with a group of researchers from university of North Carolina in Malawi. I am sorry that you could not come at your appointed time, but, we still would like to hear from you concerning your experience with cervical cancer screening with VIA and thermo-coagulation treatment. We also want to hear other problems you encountered after the screening, and the challenges you had that prevented you from coming for the next visit. Your input is very important to us, as it will help us understand how best we can screen cervical cancer here in Malawi.
2. R: Okay.
3. I: There is no wrong or right answer, so everything you can share is very welcome okay, and that you say shall be confidential, and will only be used to make this health program and health questionnaire better. I will audio record this interview to help me remember what was said, but your name or any identifiable information will not be connected to anything you say.
4. R: Okay.
5. I: So firstly can you tell me your understanding of cervical cancer screening and the treatment that you received?
6. R: When they came [screeners] and explained to us about cervical cancer, I saw that as a very good initiative, because we do not know what is going on inside our bodies. The did their job, they screened me and I was found with cervical cancer. They told me that “with what we have found, we are supposed to do thermo- coagulation”, and they did that. After thermo- coagulation they said I should not have sexual intercourse with my husband for three months. After this, they gave me another date to come at (name of Hospital) but I did not manage to come as I went for a very important meeting at (Name of place). Then I went on another day, this day we were not screened, we were just asked several questions and then they told me to come again on 27th. So, my in-law was sick and admitted at (name of Hospital) because she had a stroke, it was not possible for me to come on that day. I came on another day and I was told that “if you missed your appointment then you cannot be helped as the doctors are not available”, so I went back and I never went there again.
7. I: You were told to come on the 27th, which month was it?
8. R: I think it was (Month)
9. I: And that was the time your in law was hospitalized? And which month did you come again?
10. R: I came on 3rd (Month)
11. I: Okay, and that was when you were told there were no doctors?
12. R: Yes, they said if I missed my date then I have to contact the doctors so that they can give me another date. I called on [name not mentioned] and he said that if I missed the date it will be difficult to meet the doctors so we just have to set a different say. Then I went back home.
13. I: Okay, so when you went for screening, how were you screened?
14. R: They used my urine
15. I: And you were told that you were found with cervical cancer from the urine?
16. R: Yes, I was found with cervical cancer.
17. I: Okay, you said thermo- coagulation was done, where was it done?
18. R: Right here at the nursery school. thermo- coagulation was done in there and after that they said I should not have sexual intercourse for 6 weeks with my husband. After the screening I started producing a lot of vaginal discharge and I called the doctor to report that. He said it is nothing to worry about and that it would stop. They also gave me 30 pills that I should be taking 2 times each day. Then the fluids slowly reduced and I now they come out normally.
19. I: Okay, so after screening and thermo-coagulation, the time you came and was told to come on 27 what else did they tell you?
20. R: They did not say anything more.
21. I: They did not tell you the results of the screening?
22. R: No.
23. I: You went to central hospital?
24. R: Yes.
25. I: Which offices?
26. R: I think they call it (Name of place).
27. I: Okay, after you arrived, what did they tell you specifically?
28. R: I met a doctor whom I don’t know, he was not part of the team that came during the screening. He said that I should call the doctor who told me to come and when I called him, he said he was at (name of hospital). He told me that if my appointment already passed, I should just go back home and come on another day.
29. I: Okay, what else happened?
30. R: On that day I was not screened again, they did not even do any examination. They simply asked me questions about how I was feeling that time and I told them that I am fine compared to how I was feeling soon after the screening. I told them the only problem that was left is that I had pains in the lower abdomen. Then they told me that with hat you have explained, you should come again on 27 (month).
31. I: Okay, concerning the screening you had previously, what did they say?
32. R: They did not say anything.
33. I: Alright, so we want to hear your views concerning the cervical cancer screening, just like the one you went through right?
34. R: Yes.
35. I: Why did you chose to get screened or to take part in this study?
36. R: I saw it as the best thing to do because there is no way of knowing what is going on in our bodies. We didn’t even know that there is cervical cancer, we knew that after the screening team came and I was screened and was found with it. So I think that even if the person is not sick, it is good for them to get screened to know their status.
37. I: Before the screening happened you had never heard of cervical cancer screening?
38. R: No, it was my first time.
39. I: Okay, so what made you to have the screening?
40. R: It was because of the way they were explaining. They said that they had come to screen for cervical cancer, I was so amazed because I did not even know how that cancer is found. After they explained all their procedures, they said that it is hard to know if you have this type of cancer because you don’t feel pain and yet in the process you are rotting inside. They said the cancer cells start to eat up the cervix and these cancer cells are transferred by the man during sex. They said that if the cancer has spread on the cervix, it can also spread to other parts of the body and when that happens, there is no way you can get healed. They said that it is better to get screened so that you should know your status. From that, I thought it was better for me to get screened because I might assume that I am fine and yet I have cancer spreading inside of me. I was actually the first one to get screened on that day [chuckles].
41. I: [Chuckles] Okay, so this was your first day hearing of cervical cancer, and you were the first one to get screened; what worries did you have before the screening happened?
42. R: I was very worried! I was telling myself that they are saying that after they have found the cancer cells they will do thermo-coagulation, should we say there will be spanners and they will have to be pulling the cervix as is the case when doing sterilization? Those are some of the worries that I had.
43. I: Okay, what other worries did you have?
44. R: My other worry was that after they have removed the cervix, will I still be alive; wont I die because of the procedure? Wont I end up leaving my children hopeless [laughs] maybe I should just go back! Those are the worries I had.
45. I: Okay, were there any misconceptions about the screening or anything you heard?
46. R: There were a lot of women at that place, and each one of use was worried about their life, no one wanted to be the first one. They were all waiting to see if the one who was going to start will come out fine. The assumption was that maybe we will be unable to walk straight after the screening as it is with family planning. but, I had made up my mind that come what may, I will still get screened. The people who were in front of me came after me because they were scared, I was the first one. After they saw that I have come out fine, they were all encouraged to go in as well.
47. I: Alright, so you have said that after the screening they told you that they have found cancer cells and that they have to do thermo-coagulation, not so?
48. R: Yes.
49. I: How did you feel after they told you that your results were abnormal?
50. R: I was very worried. I thought that ‘now that I have been found with cancer, will I still be alive’. I heard that once you have cancer, it spreads until the whole body is infected. I was worried about that and since there was no way I could see if the cancer is really spreading or not, I thought I would just wait for the time when I die.
51. I: [Chuckles] Okay, so how worried are you currently?
52. R: I am still worried because I don’t know if the cancer is still there or if it ended, since I did not get my final results.
53. I: Okay, what other worry do you have?
54. R: Aa no, the only other worry I have is that since they told me to go back home and come back on another day, even if I went today to get screened again, will they welcome me considering that I missed my appointment?
55. I: Alright, back to day you got screened, from the time you got there until you left for home, what do you think was done well?
56. R: I think what went well is that through the screening, I was able to know something which was developing inside of me. I was not sick at that time and so I used to say ‘how will they find cancer and yet I am not sick’. But now that I got screened, I think it is a good thing because now I know that I have cancer.
57. I: Alright, what do you think could have been better from everything that happened?
58. R: Aa, from the time I got screened?
59. I: From everything that happened the day you got screened, what do you think could have been done better?
60. R: I feel everything went well; the doctors explained very well and everyone understood. The only way a lot of women gathered the courage to go and get screened was because of the way they explained, there is nothing which did not go well. The only thing maybe was if you need to have thermo-coagulation done. The screening process was fine, but during thermo-coagulation there was some pain [chuckles]. Still, I knew that the body is bound to feel pain, but since I was being assisted I didn’t find any problem with that.
61. I: Okay, you have talked of pain during thermo-coagulation, which was the easiest part?
62. R: The easiest part was after they examine you.
63. I: How were they examining you?
64. R: We lied down, just as it is in labor, then they were using things but I couldn’t see them since I was lying down. Then they took some cotton and some metals and they inserted them in the vagina, that is how they tested to see if someone has the cervical cancer or not.
65. I: Okay, so that was the easiest part?
66. R: Yes, that was the easiest part or those who did not have cervical cancer. Those who were found with it however, it meant that they have to go through thermo-coagulation and that was painful. From there, you go home and then you have the vaginal discharge, so it was a worrisome process. But, there is need for women to get screened since it is not hard.
67. I: Alright, it can be hard for people to come for follow-up, you have told me that on the first date you had a meeting to attend to and on the second date your in-law was sick, not so?
68. R: Yes.
69. I: Besides these two, tell me why you were unable to come for your follow up visit?
70. R: After I called the doctor, he told me that he will have to set another day for me to come, and I never heard back from him so I didn’t know when I could go.
71. I: Alright, is there anything else?
72. R: Yes, a funeral. The in-law who was in the hospital died.
73. I: Alright, for other women who were screened and given a date to come to central hospital; what challenges do you think other women have to come for follow-up?
74. R: The other women went and they were given their final result. They were told that they no longer had cancer. The women from my area.
75. I: Okay, there is no one who did not go?
76. R: No, everyone from this area went and they were all told that they no longer had cancer. They went on the dates they were given, at least they had the time to go there, so they were give their results.
77. I: Okay, let us think of other women in other areas who failed to go, what do you think the reasons could be?
78. R: It could be lack of transportation, for us to find money here, it is hard. When the doctor asked me if I was going to be able to come to the clinic or if I would prefer that the doctor’s visit me at home, I explained to him that the only problem is transport. They say they will refund the transport once you go to central but for you to borrow money to go there is a problem. So they might have failed to go because they did not have transport.
79. I: Alright, any other challenges?
80. R: No.
81. I: Alright, how best can we help women overcome the challenge you have talked about?
82. R: People fail to go to the hospital for medical assistance because they don’t have transport. So if you could do it like you used to, there was a time when the car would come to pick people up so that they can receive medical assistance. After they have received treatment, they would be dropped off home again. I think that would be helpful.
83. I: Alright, did you discuss the screening with anyone?
84. R: I am the one who tells my friends about it. I tell them that there is an organization which tests for cervical cancer. At first it was a new thing for me, but after I got screened, I am the one who tell my friends the benefit of getting screened. I tell them that I was not sick but when I got screened, they found me with cancer so it is better to get screened.
85. I: Alright, you discuss it with all your friends?
86. R: Yes, with my fellow women. I tell them that for those who have not been screened, it is better for them to get screened so that they know their status. Most people who want to get screened are because they know that we were found with cancer and yet we are strong and healthy. Most people did not expect us to be found with cancer, so we also tell them that it is better to get screened.
87. I: Alright, what about your partner or family members; did you talk about it with any of them?
88. R: I explained everything to my husband and he understood. I told him that it was just unfortunate that only women were being screened for cancer. I feel it could have been better if the men were also screened because they said that the viruses are transmitted to the woman from the man. I think it would be better if the men were also being screened?
89. I: How would it have been better?
90. R: The benefit would be that the men would also know if they have it or not. If they found means of dealing with it, it would mean that there would be no transmitting to each other.
91. I: Alright, you have said that he understood after you told me, but what was his reaction after you told him?
92. R: He said that it is good that I know my status and I that I should stick to medication up until the cancer is cured.
93. I: You talked about it before or after the screening?
94. R: After the screening. The doctors came and called all the women to come where the screening was taking place and they told us what the screening is about. After they told us, it was mostly women at that place and so we did not even wait to go and tell our husbands first, the opportunity was right in front of us and we took it. We explained to them after the screening had happened.
95. I: Okay, you did not need their permission?
96. R: No, no need.
97. I: Alright, after the screening you were told not to have said for 6 weeks is it?
98. R: Yes.
99. I: Was this a challenge to you? or what did your husband think after you told him?
100. R: It was not a challenge for him but I think he found it hard to accept. That is because after I told him that we should stay 6 weeks without sex, I think he found it difficult not to have sex for 6 weeks. In the end, he got another woman and started to stay with her. I asked myself that is 6 weeks such a long time that he has to take another woman; if it is then how does he manage to wait after I have given birth? I however decided that all that should not bother me, so long as at the end of it all I get cured from cancer.
101. I: That happened after you explained to him that you have to stay for 6 weeks without sex?
102. R: Yes.
103. I: Approximately how much time passed?
104. R: 3 weeks passed and then he went and married another wife, she is still with him.
105. I: Alright, do you think men should be more involved with cervical cancer screening for women?
106. R: I think so.
107. I: How should they?
108. R: I think they should also be screened for cancer. It should be just like it is with HIV, everyone needs to be screened and from there we ca plan ahead. I think if the man has taken part, he is a lot more careful because he knows that the disease could lead to his death. I think he can easily decide to be more careful if he has taken part.
109. I: Alright, so how would we encourage them to be involved?
110. R: It should be just as you taught us about it. You should call them together and tell them that there is cervical cancer and it is spread by men who then transmit it to their wives. Then they should also be screened.
111. I: Okay, but you said that the day you were being screened, only women were there; not so?
112. R: Yes.
113. I: So how will we get the men to come?
114. R: I think that will depend on you, the doctors. I think you need to come and call the men to come, and then you can teach them. After explaining to them, they would take part because they also can’t see the cancer. They would therefore be interested in the screening as well.
115. I: Alright, what new thing did you learn about cervical cancer or cervical cancer screening that you did not know before the study?
116. R: The only thing is that I realized that there is such a thing as cervical cancer screening. It was the first time I had heard of it, I thought cancer only targets the legs or breasts, and not the cervix.
117. I: Okay, and about how to prevent cancer?
118. R: Aa, that would be hard. Preventing cancer is hard for us women because since we are married, it is hard for us to know what he is doing when he leaves home. He could go and sleep with a woman who has the cancer, he will contract it and when he comes home, he will transmit I to me. So it is rather hard.
119. I: Alright, but who do you should be screened for cervical cancer?
120. R: Men and women who have never been screened, older girls as well.
121. I: What do you mean older girls, what age?
122. R: 25 or around that age. It is hard to know if they started having sex or not. Even the 18-year-old girls, they should be taught not to start having sex at such an age. I have heard that sometimes cervical cancer develops because the person starts having sex before the bones are fully developed, so I think the girls should also be taught on that.
123. I: Okay, how often should the screening be done?
124. R: I think if there was a committee put in place specifically for screening and teaching the girls not to sleep around. It is no longer a secret nowadays, everyone knows about sex. It is therefore important to advise them not to have sex before their bones are fully developed since that could lead to cervical cancer. They need to be advised that if they start having sex, they will get pregnant and they are too young for child birth such that in the process, they might develop cervical cancer and in the end they will have their cervix removed. They will have trouble keeping their marriage in the end since most men want children nowadays.
125. I: Alright, so how often do you think the screening should be done?
126. R: I think it needs to be done often. Most people in this area did not get screened, they were scared at that time. they are now worried that they might also have the cervical cancer since we were found with it and yet we were also healthy. So they need to be coming often up until everyone has been screened.
127. I: What do you think those people who did not go for screening were scared of?
128. R: Because some people were telling them that they have seen cervical cancer screening being done before and they take out the whole cervix to do it. They used to say that after the screening the person has trouble walking or doing any household chores. Because of that people opted not to go and get screened, so that they should be able to do household chores. After they saw that we got screened but we are still able to do our chores after the screening, they also want to get screened.
129. I: So you think they are interested in getting screened?
130. R: Yes, they are interested. A lot of women have been asking when you will come again. When they see the car, they think that you have come to conduct the screening.
131. I: Okay, so they were previously scared, do you think they now understand about the screening or they still don’t understand?
132. R: They now understand very well because they look at us, those who got screened as an example. They see how we are fine and we are able to carry out our usual chores and they are also interested. They are worried that they might have the cancer and that it is slowly spreading, so they also want to get screened.
133. I: Okay, do you think some women are scared of getting screened because of stigma?
134. R: Yes.
135. I: Please explain that for me.
136. R: In my case for example, after I was found with cancer, my husband took another wife. Some people lose interest in the screening because they are scared that their marriage will also end.
137. I: Okay, any other thing they might be afraid of happening if they are found with cancer?
138. R: Aa, I think mainly it is that one, that they are scared that their marriage will end. At first it was about being unable to do household chores, but now it is about marriage.
139. I: Alright, thank you. What are some of the barriers that women might face in receiving this service?
140. R: For some women, it could be because they did not tell their husband. What you need to do when you want to conduct the screening is to tell people in advance when you are going to come, so as to give other women the chance of talking to their husbands about it. The day they came here, I thought that they were only going to explain to us what cervical cancer is all about and then give us a day to go to the hospital, we did no know that screening would happen right there. So that would be a challenge for some people and it would create misunderstandings in the home. So you need to come and set a separate date when you are going to come, to give a chance for the women to tell their husband.
141. I: Do you think some men would tell their wives not to go?
142. R: No, I don’t think they would refuse. These are hospital related things so they wouldn’t refuse.
143. I: Alright, how do you think cervical cancer screening should be provided to ensure that more women can get screened?
144. R: If the doctors came and screened them here and then came to pick them up again on their visit date up until they are cured. As I said, the hardest thing to find here is transport. The desire to go to the hospital might be there but it is hard to find money. If they came and explained that they will come back on a certain day to conduct the screening, there would be a lot of women. Some women also fail to get screened because there are menstruating or they are pregnant at that time.
145. I: Alright, let us now discuss about self-collected vaginal swab for cervical cancer screening. A new method has been developed for cervical cancer screening. It involves having a woman collect a swab from her vagina and submitting it at her convenience to a health facility for testing. However, unlike VIA, the woman would not get her result immediately and would have to return to health facility to get her result a few hours later or the next day. Have you understood?
146. R: Mm.
147. I: What do you think about this idea?
148. R: I think the best way is to get screened and to get assisted right away. With the waiting, a lot of people would be lazy, especially since they don’t know what is happening inside their body and they are not even sick.
149. I: Okay,
150. R: Especially when they are told to come back the next day, they would prefer to stay home or to go to hospitals nearby.
151. I: Alright, which method are you more interested in?
152. R: I am more interested in the method were you get screened and then you get the results on the same day.
153. I: Okay, what don’t you like about this idea?
154. R: It would be hard for the person to come back.
155. I: Okay, what are the good things about it?
156. R: The good thing is that after you collect the swab, there is no one else who will need to see you naked. Because you are collecting the swab yourself as well, you can find ways of doing it carefully because you know that if you do it wrong you will feel pain. So I think it is still good.
157. I: Okay, what do you think other women in your community would think about the self-collected vaginal swab technique for screening?
158. R: It would be hard for me to know what another person is thinking; I only know what I think.
159. I: Still, you know the things your friends like and the things they hate. From that, what do you think they would think?
160. R: They would think that maybe these things are satanic, that after they have given the swab with the vaginal discharge, you will find a way to come and pump their blood. That is what happens when you take to heart what other people tell you or what they think.
161. I: Okay, of the two methods, which one do you think they would be most interested in?
162. R: I think of the two they would prefer the VIA one where if they are found with the cancer cells, thermo-coagulation happens right away. This self-collection method is good, but it is not very good.
163. I: Alright, what difficulties do you think women would face in self-collection technique?
164. R: The only difficulty would be with regards to travelling to the hospital. If you are told that your results show that you have the cervical cancer, but then they tell you ‘come on such a day so that the doctors can do thermo-coagulation’. The travelling would be the most difficult, most women wouldn’t access the service because of transport problems.
165. I: Okay, but when it comes to actually collecting the swab, what challenges do you think women would have with this?
166. R: Aa, I don’t see anything that would make the woman fail to collect it. The only reason could be because she is menstruation or because she is pregnant, besides those two I don’t see anything else.
167. I: Alright, let us now talk about your recommendations for the future of the National cervical cancer screening in Malawi:
168. R: Okay.
169. I: In your opinion, should MOH consider including self-collected vaginal swab for cervical cancer testing to the cervical cancer screening program?
170. R: I think they need to consider it because different women will like different methods. I might like this method and yet another woman would not like it, so they need to include this one as well to give the woman an option of choosing which method they want.
171. I: Alright, if that happens, do you think it would make it easier dor women to undergo screening?
172. R: I think it would make it easier because everyone will easily choose the method they would prefer.
173. I: Okay, which groups of women do you think would be most suitable for self-collected vaginal swab for cervical cancer screening?
174. R: Those who are not menstruating and those who are not pregnant can be suitable for this method.
175. I: Okay, why not the pregnant ones?
176. R: Because let’s say they have been found with the cervical cancer, it will be hard for her to have thermo-coagulation because she is pregnant.
177. I: Alright, thank you. Why do you think women would prefer to go to a hospital for screening with medical providers?
178. R: It would be better because if you have been found with the cervical cancer, they will do thermo-coagulation right away, the decision will be made right away. With the self-collecting vaginal swab method, you have to wait some time for the results whereas everything is finalized right away with the VIA method.
179. I: Okay, is there anything else?
180. R: [Chuckles] No.
181. I: [Chuckles] Okay, this is also the end of this discussions, but if you have any questions please ask.
182. R: The question is the same one I had at the beginning that, since I did not get my final results on whether the cancer is still there or if it is gone, how will I know my status?
183. I: Okay, I will take that down and will ask the right people.
184. R: Okay.
185. I: Is there anything you would like to add?
186. R: I just want to encourage you that a lot of people in this areas want to get screened. After we were found with cancer, those who did not get screened are worried that they might also have it. Even those who failed to get screened at that time because they were pregnant or because they were menstruating, they are very interested and they want to get screened. Some were still under-aged but maybe by the time you people come again to screen they might be of age, so you doctors need to come here and make sure that a lot of people are screened.
187. I: Alright, is there anything else?
188. R: No, I think that is all. You just need to come here often to encourage us, so that we should know if the cancer ended or if it is still there.
189. I: Alright, thank you very much for your time today.
190. R: Thank you.

THE END
