# Supplementary material for: Perceptions of cervical cancer and motivation for screening among women in Rural Lilongwe, Malawi: A qualitative study
Source: PLoS One. 2022 Feb 7;17(2):e0262590. doi: 10.1371/journal.pone.0262590 (PMC8820632; doi:10.1371/journal.pone.0262590)
Supplement: S3 File — (ZIP) [file pone.0262590.s003.zip › VIA_233.docx]

**PID: VIA 233**

**DATE OF INTERVIEW: 14 NOVEMBER 2017**

**INTERVIEWER: 466**

**TYPE OF INTERVIEW: 12 WEEKS FOLLOW UP**

**KEY: I= INTERVIEWER, R= RESPONDENT**

**TRANSCRIPT**

1. I. thank you madam for meeting with me today... I am appreciative of your time. Whatever you are going to say in this study is very important. I work with a team of investigators from UNC project Malawi. Whatever you are going to say is very important to us...it will improve on our understanding on how we can work on campaigns that spread about cervical cancer screening. As such there is no right or wrong answer. Whatever you are going to say is very important; and shall be private and confidential that is why there is just the two of us in here-and shall never be shared to anyone... it is only for the purposes of this study and improving health work. Feel free tell us whatever you know... I shall record whatever you shall say using this gadget so that it can help me to retain what has been said. I can’t afford to write down everything you say... I might skip some. Everything that happens in the course of our interview is very important whether moments of laughing or any expression has not to be missed. We are not going to take your name nor any sort of identifier; and shall never be linked to whatever you shall say. Feel free to say whatever you want to say; your experiences... not so? ...
2. P: Yes
3. First of all I would like to know… about screening and the treatment you received for cervical cancer. Can you tell me your understanding about cervical cancer screening and the treatment you received 10 and two weeks ago? How did you understand that?
4. *R. we were supposed to be terrified with regard to what other people used to tell us about cervical cancer screening. We did not yield to their motives because cervical cancer is a very deadly disease that everyone is afraid of it more than AIDS. Cancer is very deadly disease and scary. So after hearing that a team of health workers has come to our area to test women for cervical cancer, we took this opportunity and went for testing. The results indicated that I had cancer cells and they I scraped the area and then they heat it. For a month we were draining water, so they advised us not to have sex and we tried to refrain from sext for six weeks. When we came here on 3rd September... no 3rd (month).. am right? No, (month)... September so they were asking us and we were answering them. That initial visit from the sample of the flesh they scraped from me they said it still indicated cancer. So they told us to go to (name of hospital)... with this woman that I have come with... when we got there they said you were found with cancer cells so return to those who sent you here so that they can give you a date when you can come back her. We came on sixth that time... that means last week thereof in (month) we were designated to come on the third from 3rd from sixth. So we came again on the third last month. So that time when we came all the signs and symptoms of the disease had gone the genitalia was dry no more water came out. So they were asking us and we were answering. And we saw that it was very important for doctors to visit us in the village and treating us for such kind of a disease... I never voluntarily came here but the doctors found us right at the village so I considered it a very precious thing. So today they have taken another sample of my fresh... them made me lye again so we don’t know when we should come back*
5. I: Now I would like to know your ideas about cervical cancer campaigns. Why did you decide to get screened or take part in this study?
6. *R: We saw that it was very important thing and helpful on our lives.*
7. I: Alright... you explained that cervical cancer is very dangerous, can you tell me if there were other things that made you anxious before you got screened?
8. *R: Yes there were rumours that it was a team of Satanists. Why could we tolerate strangers and men to play with our genitals... and confirmed that that was satanic and we are going to be killed. So we said if it is satanic so be it because those people are helping us on health issues. Doctors from the hospital can’t be foolish coming here to see us; and we can’t be smarter more than them…*
9. I: Mmh. What did you hear about cervical cancer before the screening campaign took place?
10. *R: Aah, as for us before coming of the doctors we never heard anything about it. Following the senior group's messages about coming of doctors to our village but it’s when we heard from the doctors about counselling, and all things that are involved. For it was the first time for that type of group of people to come to our area. We were just staying ignorantly-without knowing anything at all... sure…*
11. I: Alright. What other things were the people saying about this screening apart from that they said it was satanic?
12. *R: another thing that was being said... it was those people who didn’t get tested because they had fears... if you observe carefully you are going to see that it is a sign of fear. Some they didn’t get tested... and again a larger group get tested whereby some of us we were found with cancer as we are coming here.*
13. I: How did you feel when you got your screening results and when they screened you and you indicated that you had cancer cells? How did you feel in your heart?
14. *R: It hurt so much for it is a dangerous thing. Even my husband was afraid too that through that process I was found with cancer cells… mmm*
15. I: ...So what did it mean to you after they explained it to you?
16. R*: I understood that after the screening and have find you with the cancer cells they will burn the infected area; after by the heating cancer cell will end. Some the cells don’t get finished... but here it’s a hospital they find means to help us survive*.
17. I: ...Mmh, alright. So during the screening process what do you consider to have happened well?
18. *R: You mean the time when they screened us?*
19. I: Yes, what went on well?
20. *R: As for me I saw that everything was ok and very good in the sense that these people found us at home and tested for the status of our bodies so after they tested us find us with the disease when we least expected... we were doing all our routine works without any problem or pain like digging... etc... so when doctors like you came and find us with such ailments when we were comfortably staying without being aware of them, we felt supported for we knew that they will end them and ensure our survival.*
21. I: What else?
22. *R: Another thing which happened well is that they said that we are...[R: coughs] testing you this disease since your village is far from the hospital. So the arrangement will be like: whenever you don’t have transport you can borrow from friends and we shall reimburse both to and from so that you can take care of the borrowed money if there will be any of that sort. but the money is not enough*
23. I: What do you think could have been made better?
24. *R: About transport?*
25. I: No, everything even if it is about transport... anything which you think if it happened in that way it would have been well
26. *R: We see that it is a great thing for we know that wherever there are good things distance doesn’t matter. Like today I have come at 10 o’clock from the village but we left early in the morning on a bike to drop us at* (Name of place)*. from* (Name of place) *u to this place because we are in search of our luck of survival. So that is where we saw it was good... life is good I have given birth to seven children... that is me, five male children and two female children. so the father to those five died so the one I have come with is the second one-the father of the two and I have made two children with him. So the rets of the children are orphans if I die again who is going to take care of them?..."..." no one my mother died and there will be no one to look after them so I saw it as very important thing which cannot be predicted, but God made us dream of it by making the doctors to rise and meet with us. so we have love for our God who sent the doctors to come for us*
27. I: Ok, during the time they screened you, which part did you find to be easy?
28. *R: During the time they screened us... I found everything to be easy they took urine and blood samples to see if we didn’t have HIV or we weren’t pregnant or we weren’t on menstrual period...* *So they said if we were pregnant they won’t test us, if we were on period likewise they won’t and if we are negative they are going to test us. A lot of people were sent back because they were on period and some pregnant so they went back complaining thinking that perhaps they have "cancer". So I saw that this thing was good and is wasn’t difficult at all...* [I: mmm mmm]*... they were explaining to us about all the involved processes... sure...*
29. I: Now I would like us discuss about challenges which women can face. So like that time they told you come back here to the hospital. And you have explained that you cam. Maybe you can tell about... what type of challenges did you have when coming back for the follow up visit?
30. *R: For us to come back to this hospital?*
31. I: Yes. What challenges did you face?
32. *R: After they tested us cancer?*
33. I: Yes when they tested you at the beginning they told you to come back. Indeed you were able to come but I believe there might have been challenges concerning your coming to come here. We would like to know about things like that.
34. *R: For me to come what hindered me most is transport. My husband tried to find some piece works. He got it... after getting the work done we had to wait for some time before payment was made... so with that type of development it was a bit hard. So we tried and I also got busy searching and I found it and I came alone. I came alone the first trip. so the second trip we came both of us since we found a piece work and then I gathered enough for myself... we topped up by borrowing to cater for my husband's transport and came here at good time.*
35. I: Mmm, ok. There are other women who got screened with you from your area, what do you think can make them fail to come for the follow up visit? What can be another problem?
36. *R: They put us in groups of two on the time we come. so the group that came later said they were not given date of appointment*
37. I: I mean after screening they are told to come for check and are failing to come because of different problems. so I would like to know about the problems that women face about coming for the follow up visit
38. *R: Since...* [I interrupts]
39. I: You have explained about money problems.... what other problems can women have? They really want to come but are failing, perhaps making it but with struggles.
40. *R: Mostly is because of some dangerous eventualities*
41. I: What kind of a dangerous thing?
42. *R: like funeral and you are concerned. For with that for you to move it is a bit difficult. so (name of staff) gave us phone number to make calls in the event of such problems to give us a different... since you doctors are busy people so if we come on the date you have given us and don’t find you it isn’t better at all just like what has happened with us today. They told us to come on the 12th... being Sunday I called (name of staff) if we still had to come on that Sunday. So he said don’t come on Sunday come on Tuesday. So we have come here today we found him gone somewhere far. They had to call him with his friends. Luck enough his colleagues came faster and started helping us.*
43. I: Alright apart from funeral, what other problems?
44. *R: May be a child is sick; or have been suddenly invited somewhere for issues... and that is a problem surely you should be giving us your phone numbers. Sometimes when we flash name of staff) his phone can’t be properly reached... So with your number may be we can be reaching you perhaps it can help.*
45. I: Anything else apart from what you have said?
46. *R:There isn’t anything more, I can’t lie*
47. I: Alright, you have said if we give you phone numbers it can help. What else do you think can we do to better help women deal with the challenges they face?... "..."
48. *R: During the first visit (name of staff) picked us from home to the hospital. So after they gave us transport this time it was enough and we had an extra K500 just because he carried us here.*
49. I: What else can we do?
50. *R: another thing?*
51. I: Yes, apart from picking you as you have said (name of staff) did?
52. *R: I don’t know what to say... [both laughs]*
53. I: Alright, perhaps you will remember in the course of the discussion. I would like to know about the support you get from your husband and the community. When we live in the communities we depend on one another not so...? Who did you discuss with about screening?
54. *R: I explained it to all my relatives like my young sisters, brothers, and others that live with me that I was found with this disease. so everyone at home knows that we come here because of cervical cancer disease.*
55. I: Who else did you talk to apart from your relatives?
56. *R: My husband and we come together just as you can see. So he is happy that doctors came and found me with the disease which is a rare thing*.
57. I: What did your relatives say after you told them?
58. *R: They said: "we are grateful that they find you with the disease before its signs and symptoms. we that the hospital for the faster help"*
59. I: So what do you think does your husband think about cervical cancer?
60. *R:He thinks it is important that we should be coming to the hospital to get help as long as we survive.*
61. I: Alight, what type of support do you expect from your husband?
62. *R: You mean from my husband?*
63. I: ..."..." mmm, so that it can make you come here
64. *R: Oh, for me to come here? what type of support I get from my husband?*
65. I: What type of help or support would you like to get from him?
66. *R: As for my husband... the first thing after I get into a car I vomit and get nausea and usually have blurred vision, so I expect him to hold him.*
67. I: What if he isn’t there but you still need to get to the hospital?
68. *R: I still get here*
69. I: What support does he give you?
70. *R: He tries his best to find money for my transport. Telling the truth he really tries*
71. I: Mmm... alright. Thank you very much. when you look at him, what is the level of his interest in learning more about cervical cancer?
72. *R: He very interested if he was right here beside me and hear about the questions you are asking me it would have been very important*.
73. I: What makes you think he is interested?
74. *R: The love he shows me*
75. I; What type of love?
76. *R: After they tested us we were told to spend six weeks without having sex. I saw that the six weeks was accomplished without asking me for it. We would go together for bathing I took my clothes off and so he would do with his. All he could say is: “better you get well letting a month or two to go, I can’t die...you are still my wife". And am sure that with today's thermo heating and the instruction we have been given to spend a week without sex, he is going to understand*
77. I: Do you think men should strongly take part in cervical cancer screening campaigns?
78. *R: Yes they should take part because we live with them*
79. I: How can men tale part?
80. *R: Men must take part and get involved because this disease sometimes is sexually transmitted... it’s like the man some time... just like the way we are that had a husband who died and now I have this one who also has a wife. So there is too much blood contact mine, my late husband, the wife of my current husband and him as well... so the blood is now mixed...* [I: mmm mmm]. *So that time they were saying that this disease is coming through sexual intercourse.*
81. I; So what should be their role... what should they really be doing?
82. *R: They should be understanding and faithful. Have one wife and love each other*
83. I: What other way should they get involved... on cervical cancer screening?
84. *R: They should be listening... like what you are doing now they should have been part of it. They should know how the disease comes and how the questions are coming... they should be with us to hear about the questions you are asking us now*
85. I: How can the hospital get involved in encouraging men to take part? so that we can make them greatly to take?
86. *R: Just like how we have come today and given us an appointment, the man should not be busy on that day… whether he had a journey surely he must postpone it.*
87. I: So how can we as the hospital encourage them?
88. *R: That will depend on you*
89. I: We want your ideas how should we go about it... [both laughs]. We want to know so that we can improve on the services... yeah
90. *R: The way this thing is happening is that the transport for coming here we have borrowed. and the rest we get it here going back home... the problem comes in when it comes to issue of transport since it means that we have to look for the man's transport to and from.*
91. I: So what do you want us to do?
92. *R: You should be providing transport for us both-woman her and likewise man his own transport.*
93. I: What other way can we encourage them to take part?
94. *R: As partners they cannot leave us during problems like these, so encourage them that they should stop coming with us... on that you should really encourage*
95. I: How should we encourage them?
96. *R: The way to encourage them... you are required to talk to them and give them wise words that they should be escorting us to the hospital and when we get home it should not be something they can use to excuse themselves for their wrongs when we talk to them. if they do that it means there is no love*
97. I: We are talking about men, how can we teach them about cervical cancer? How can we teach them?
98. *R: I feel it is very easy to teach them... the instruction are simple tell them that you see how your wife is here he will again l down for another sample. feel that she loved you and you should also love her the same, devote yourself to her work hard to get transport.... surely some days like how it was today are bit difficult*
99. I: So if we want to teach them about cancer how can we teach them for them to know?
100. *R: OK, for them to learn about cervical cancer... but they surely understand*
101. I: How can we teach them? how do we go about it?
102. *R: Ok... I thought you wrote here, for I do not read.*
103. I: No, we want you to tell us what we should be doing. we don’t have any written material but if you tell us your views the ones conducting the study should be able to come up with education materials indicating how they can go about teaching men
104. *R: Ok... teach men in this way we understand that cancer disease has got some natural causes... some, we understand that it comes through sexual intercourse since the man might have extra sexual partners and it is from where he can bring the disease or AIDS. For AIDS also comes through sexual intercourse. There are a lot of diseases that are coming through sexual intercourse because of lack of understanding of me or my husband. Hence there is need of counselling that men should take care to avoid contacting the disease from other women... if I am wrong you will correct me.*
105. I: Remember we said there is no wrong or right answer... [both laughs]... feel that whatever you are saying is ok and very important. Anything more?
106. *R: Another thing is that, a man... when a woman talking to the man about other things which he is not doing well the man thinks that the woman is just against him. But it is not like that...she just wants both of you to survive. when he sees old people should ask himself will I get older, that’s why some of us we try our best to come to the hospital so that we can reach where our friends reached... you are supposed to give men such kind of encouragement*
107. I: Alight thank you very much. I would like to test your knowledge about cervical cancer. is there anything new you have learnt about cervical cancer? or about screening of cervical cancer, what you knew before the study?
108. *R: I knew nothing*
109. I: I want you to tell what you didn’t know one by one
110. *R: No I… didn’t know that a woman can have cervical cancer. When we hear that so and so had cancer on his hand and have cut it, we really didn’t know what is was like to have cancer. and again that so and so had cervical cancer and have removed the womb, we didn’t know how it was like and how one contracts it. So by giving us the opportunity to see these things with our eyes we are thankful for we didn’t know all this*
111. I: What else apart from knowing about cancer?
112. *R: Another thing? We know that when cancer is detected it is heated*
113. I: What else?
114. *R: They take urine and blood samples*
115. I: You didn’t know about it
116. *R: Sure I didn’t know... and to us we found it strange to be told to go and urinate... blood testing... we have been doing it on different occasions especially HIV/AIDS... but testing us urine in cancer testing, that is strange and we didn’t know*
117. I: What things did you know?
118. *R: Knowing my status, how I can protect myself and how I can do*
119. I: Is there anything else to add?
120. *R: Nothing.*
121. I: Who do you think should get screened for cervical cancer?
122. *R: From the village?*
123. I; Anyone you think is eligible for cervical cancer testing?
124. *R: In our village a lot of people really want to test...*
125. I: what type of a people?
126. *R: There is (Name withheld)…*
127. I: I don’t want you to tell me about their names. I just want you to tell me the type of people you think are supposed to test for screen cervical cancer
128. R: In our village the people who want to get screened cervical cancer is a lot of them because they have been asking us if there was any possibility...
129. I: What type of people?
130. *R: Older people, some of my age. for the ages are different*
131. I: You have just said people, and I won’t be able to know that were referring to a kid, or woman
132. *R: Women... [laughs]... it women who want to test for cervical cancer*
133. I: So you explained about age and made a reference of yourself. What other type of women do you think should get the test?
134. *R:There are some women who are a bit older and really want to know their status. as for us at least am younger saying that I have seven children you can’t believe... saying I have a grandchild... my daughter is pregnant sure... so you can’t t believe that I have seven children... doctors came to our village upon request from our chief who met at meeting in* (Name of place),  *so when they came they said for them to come again three years will have to pass that is why a lot of women are panting for the help and asking if they can come here on their own.*
135. *R: So we said we can know about that.*
136. I: In your opinion how frequent should women get screened for cervical cancer?
137. *R: Only one year, and the other years to monitor those that were not found with the disease*
138. I: Why do you think so?
139. *R: Because this disease is very dangerous. for a person who is sober must appreciate that this disease is dangerous.*
140. I: Mmh mh.
141. *R: It is dangerous disease making you not to "eat what you really crave for"... [both laugh] you will die. For the world even the poor and you make hoes... just ensure your survival and take care of your family... so women are the ones who take care of children regardless of how poor you are still life is more precious than that.*
142. I: Now i would like to know about future cervical cancer screening campaigns. what do women from your community think about cervical cancer screening?
143. *R: They think it is a good for they were ignorant and refused to test for cancer. and now that they know what happens through us and see us we come here and go back without any problem*
144. I: In your opinion do you think women are interested... now that you explained to me that a lot of women would like to come. there might some who would not be interested to come here. in your opinion what do you think can make them not interested to have cervical cancer screening?
145. *R: For them to come here?*
146. I: Or even if doctors visit the village, what can constrain them not to get tested?
147. *R: Some were afraid*
148. I: Indeed you explained so
149. *R: Some said satanic... they know that is good to have a test because treatment is there. so people are encouraged to get tested for they see that treatment is available*
150. I: Maybe you can tell me the problems women can face as they get treatment for cervical cancer?
151. *R: Ok, problems that women face... people have different problems maybe you are on menstrual period or pregnant with these they don’t test you for cancer... since when you are pregnant the baby is already closer to the entrance of the womb as such it can’t be possible to do the scraping on you*
152. I; Perhaps the problems at home concerning husbands
153. *R: Thinking that may be the husband would say that his wife should not be touched in her genitals... that currently is ok, there are no such reports.*
154. I; What about friends?
155. *R: You mean extra marital partners?... [laughs]*
156. I: Meaning friends you hung around with. What role can they play in making women to fail to get screening for cervical cancer?
157. *R: Ok. Some of them are being interested; and others are getting encouragement after our testing. As for those who got screened before us are encouraging us especially on some misconception that spread across-like that the metals which you insert us enters into the stomach. But now we are sure that it doesn’t… all we know after test, you tell us how things are… that’s what they say. Don’t stop visiting us*
158. I; Alright, in your opinion how do you expect the treatment for cervical cancer be offered to ensure that a lot of women get screened?
159. *R: Only a year should pass and the next year…*
160. I: So how should we be doing it?
161. *R: The way you should do it… like now a lot of doctors have know our village; and our GVH has shared them his contact to all doctors were there. So when you are coming you simply call him to mobilise us and thus will keep everyone aware that you are coming for cervical cancer screening*
162. I: Alright. What is the other way we can do so that a lot of women are attracted to het cervical cancer screening?
163. *R: The other way is by giving transport just like how you are doing so that women can come easily. So you may get screened and you are found with the disease… so the requirement of coming for follow up visits when you are poor is a bit difficult. Even the piece works that we do not get us enough money that we can buy food and transport to come here. So you use the same money… if you don’t, what will your children eat? But I understand that the help you are giving us is very important*
164. I: How can you encourage women to get screened for cervical cancer?
165. *R: I would begin by telling them how I was feeling after I was found with the disease and all things I foresaw if the treatment wasn’t available. And then, now that I got the treatment after the heating of the cancer cells… I am now ok and also I have to tell them this. So by comparing these two different times, should be able to motivate women to get screened*
166. I: So we are almost coming to an end of our discussion. Now I would like us to discuss about a new method of self-cervical cancer testing. The new method of cervical cancer testing which has been discovered involves taking vaginal fluid using cotton from the vagina and deliver it to a health centre or hospital whenever a woman is has time. However, it is different from the method you used. This you don’t get your results instantly. She is supposed to get the results after some hours or a day later. What do you think about this method?
167. *R: Very good method*
168. I: How good is it?
169. *R: It is good in the way that a woman will have control over the process… you will insert cotton inside you and get the vaginal fluid and simply take it to the hospital. “You” will still be able to screen no matter how small those things are, you still be able to see and come up with results of our status*
170. I: Would you be interested to get screened using this method?
171. *R: Yes I can do*
172. I: What is the advantages of this method?
173. *R: … Still the hospital will be able to give you treatment and do the thermo heating after you get the vaginal fluid to them.*
174. I: Yes, that is the hospital’s normal arrangement... but what I want to get are your ideas on this method. It is obvious that testing and that she is found with the problem, she is going to get treatment… but we don’t know how it shall be done; what matters is this method of self testing. What I expect of you is to tell me the advantages you see in this method
175. *R: The advantage is there… like you hospital people you know both the advantages and disadvantages of cervical cancer. So it will be up to you to prescribe the appropriate treatment according to the manner of the method of vaginal fluid extraction-whether you will be doing thermo heating or whatsoever… so will be and anybody that comes will appreciate because you are the ones doing everything since it is the doctors doing it, then it is ok. Doctors know better ways to deal with it.*
176. I: What disadvantages do you see in this method?
177. *R: No disadvantages*
178. I: Ok… so do you think this self-testing method should be done at home?
179. *R: It should be happening at the hospital because if you get the vaginal fluid using the cotton for a person like myself I come from a longer distance which by the time I get here the fluid must have dried. I believe it should be kept moist and not dry by the time you get to the hospital so that you can test it easily…[I: alright]… if I am wrong you will correct me*
180. I: Nothing wrong, it is just ok… [both laughs]… how reliable do you find this method?
181. *R: It is very reliable because it is very helpful to the life of a person**
182. I: How helpful is it?
183. *R: The disease is very disadvantage and scary*
184. I: Alright, so how do you compare this new method to the one that was used on you?
185. *R: They are different in the way that the one that was used on me involved a series of tests. First they would take blood sample for HIV testing, and urine, they wanted to see the type of disease one has… so we see that would be a bit different, but still you are the ones who should know better by taking vaginal fluid and screen it; and making us lay down and take spanners, put them insider a woman and check on the cervix if there are sores or not…*
186. I: Alright, since everyone has got a choice, between these two methods which one can you choose?
187. *R: I can choose the one that was used on me*
188. I: Why exactly?
189. *R: Because taking vaginal fluid and urine we can say is the same I saw it. We would assay it is the same method, however this is vaginal fluid and the other is urine… this is urine and the other vaginal fluid. So if they take vaginal fluid, are they going to follow the same process of taking urine?*
190. I: If I may ask what were they checking in urine?
191. *R: To see if the woman is pregnant or in menstrual period. So if you are in period they would say don’t bring urine sample*
192. I: What I would like to know is the method about cancer screening not the processes of urine or blood testing because like you said that involved if you had other complications. Put focus on the method like using of spanners to screen for cervical cancer… [R: if you have cancer disease? … Yeah. So I want to know which method you would prefer most.
193. *R: I saw that the method which was used on me is the best. After they screened us and detected the germs, they burned them and it is when after that I felt much better… So I see from the time they screened us …was it July… June… ooh no but July, and thus from the time they tested us we have been releasing water up until September and by reaching October we had stopped… and this is November you can see am perfectly dry. So I don’t know after today’s inserting that how is going to end.*
194. I: Alright, what do you think women in your community can think of this method of self-testing?
195. *R: The can accept it because it is you doctors who have brought it to them*
196. I: Lets say that some women have chosen to be screened using this self-testing method… what will other women think about it?
197. *R: They can think it is a better method because it has been prescribed by “you”(doctors)… and shall surely get the vaginal fluid and show it you; and cannot find problems with that because she want to live*
198. I: Can a lot of women chose to be screened using this method?
199. *R: Yes they can choose*
200. I: Why?
201. *R: They can choose it because it is a method which has been prescribed by “you” and that treatment is available.*
202. I: To them, what could be the advantages of self-testing?
203. *R: They can think it is a good method because they are taking their own vaginal fluid by themselves and give it to you…*
204. I: What problems can women face in the method of self-testing?
205. *R: Meeting problems?*
206. I: Mmm… what limitations can they face as they test themselves?
207. *R: Maybe is an alternative to fears they have and what they can do is to test themselves*
208. I: Take it from the angle of knowledge you have about women… that the way you know a woman can find problems in such such ways…
209. *R: Problems she can face for self-testing?*
210. I: No but when taking vaginal fluid?
211. *R: I don’t foresee problems. There will be no problems because they are not the ones doing the actual testing… will simply get the vaginal fluid sample and give it to the doctor and wait for results. Thereafter the doctors come back and say that according to the sample we got from we have find you with such such problems..*
212. I: What could be the reasons if there are any that can make a woman not to take samples for testing by themselves?
213. *R: Because doctors you are the ones who know all problems of our bodies. As for us we are un learned people, ignorant and illiterate; but you better how to help us according to your school…*
214. I: Alright, any additions?
215. *R: No*
216. I: Finally, I would like to ask you about comments you can give about the future of cervical cancer screening. So in your opinion should the MoH recommend this method of self-testing with cotton be a method for screening cancer?
217. *R: Yes it is possible*
218. I: Why do you think so?
219. *R: Because it is helpful… helpful in a way that health workers continue visiting us in different areas so that we can get help from them…*
220. I: So do you really think that the MoH should recommend this method as one of the methods of cervical cancer screening in this country?
221. *R: Yes*
222. I: Why do you think so?
223. *R: I thought you saying… is itself testing or taking vaginal fluid right here at the hospital?*
224. I: Doesn’t matter where, but only the issue of taking the vaginal fluid itself is what we are concerned with here
225. *R: It is a bit problematic there… for one to get vaginal fluid from home and take it to the doctor…*
226. I: We really don’t know about where this fluid should be taken from. It will depend on what people will say. So whether it will be home or hospital, but the issues is there should be two methods-one used on you previously, and the other involving taking vaginal fluid using cotton and take it to the doctor
227. *R: It is better that doctors should be coming to the villages because most of us come from remotely located places-very far*
228. I: Do you think that this method can make it easier for women to test for cervical cancer?
229. *R: By bringing in this method there might have been something you saw…*
230. I: I want you to tell me your thoughts… [both laughs]
231. *R: These methods are slightly different. The other one it was the doctors who conducted the testing and you were able to see everything … and could commence treatment instantly-right there. Whilst this one I will just get vaginal fluid and give it to the doctor off I go.*
232. I: I Am asking that with this method of self-taking of vaginal fluid and take it to doctor for testing, will it make it easy for woman to test for cervical cancer?
233. *R; That will be difficult?*
234. I: How?
235. *R: Since you said that a woman will be taking the fluid by herself, better if it was taken by doctor.*
236. I: Alright, alright… which groups of women do you think would be suitable to use this method of extracting vaginal fluid with cotton and test for cancer?
237. *R: A lot of people*
238. I: Which type of people?
239. *R: Groups of women*
240. I: Which groups of women?
241. *R: How can I put it…*
242. I: I think there must be different groups of women
243. *R: Oh, I would say older women*
244. I: Why older women?
245. *R: Some are shy because are not used to some of these. I can say that, us young women we have been delivering babies from the hospital whilst them at home. So as for us we know that even a male doctor can help a woman just like the way a female doctor would. So I can’t be shy with a person whom am not intending to have sex with. All what they want is to help us. So as for younger women there isn’t any problem… but older women are shy to get screened. Like in our village, girls and younger women who missed the first time are much interested and say they can’t understand how they missed this opportunity.*
246. I: Alright, what type of women would could not be suitable for this self-testing method?
247. *R: Young women should use the hospital method because they are used… just like when they are delivering babies they are also helped by male doctors*
248. I: With that, I have to the end of question I had. Unless you have comments
249. *R: I have a comment… when we got screened for cervical cancer I commenced treatment by thermo heating and there wasn’t any pill or tablet given to me u like with disease like malaria whereby you are given and injection or tablet. But as for this nothing like that, it seems it is very reliable…*
250. I: Any other comment?
251. *R: No*
252. I: Thanks for your time and ideas, they will help in improving services for cervical cancer treatment and screening…
