# Supplementary material for: Perceptions of cervical cancer and motivation for screening among women in Rural Lilongwe, Malawi: A qualitative study
Source: PLoS One. 2022 Feb 7;17(2):e0262590. doi: 10.1371/journal.pone.0262590 (PMC8820632; doi:10.1371/journal.pone.0262590)
Supplement: S3 File — (ZIP) [file pone.0262590.s003.zip › VIA_239.docx]

**PID: VIA 239**

**DATE OF INTERVIEW: 2 Nov 2017**

**INTERVIEWER: 466**

**TYPE OF INTERVIEW: 12 weeks follow up**

**KEY: I= Interviewer, R= Respondent**

**Interview summary:**

According to this woman, she made a decision to get screened for cervical cancer when she heard that the cervical cancer screening team had come to her area to do the exercise. She decided to go because she wanted to know her cervical cancer status. During screening, she said she was learnt that she was positive for cervical cancer, she said she was worried and thought that maybe her husband is cheating on her by having multiple sexual partners, then she was told about the Thermo-coagulation treatment and counseling, she became comfortable, During thermo- coagulation process, she felt pain, she said it felt like her uterus were being pulled and she thought it was because of material used during the screening process. Despite feeling the pain, she was not worried because according to her, the pain was part of the healing process, She was happy that results and treatment happened on the spot.

Regarding the follow up challenges, she said monetary challenges and long distance were the main hindrance for some women to go back to the clinic as scheduled and she suggested that the clinic staff should be provide a vehicle to pick women from their areas. The participant recommends that the screening should continue in the communities so that many women can benefit from the services. On self-collection of vaginal swabs, she said that MOH should consider this method, because it will be easy for more women, though she not very confident with the method, she that it’s tricky because sample could be comprised since its being done by non-professionals’.

**Interview text:**

1. I: Thank you for meeting with me today. I really appreciate your time and you should know that your input will be very helpful. I am working with a team of researchers from the UNC Project; where we are!
2. *R: Okay.*
3. I: Your input is very important to us and will help us understand how best we can conduct cervical cancer screening campaigns in Malawi.
4. *R: Okay.*
5. I: So all what you are going to say will help improve the cervical cancer screening campaigns, in your responses, there are no right or wrong answers. We will gladly make use of everything that you will say and will be confidential. That is why there are the two of us in this room.
6. *R: Alright!.*
7. I: Yes. The information you give us is only to be used to make this health programs better, as i said I will record this interview to help me remember everything said, but your name or any identifiable information will not be connected to anything you say. Understand?
8. *R: Yes.*
9. I: Firstly, I would like to know your understanding on the cervical cancer screening and the treatment you received the past 12 weeks?
10. *R: What i can say is, during all these years i was just staying, but when the providers came, after i got screened, i was one of those in the group, but when i got treatment i was told that am fine.*
11. I: what group is this?
12. *R: The group that were found with some viruses that cause cervical cancer during screening.*
13. I: What is your understanding on why the screening was happening?
14. *R: Because i was just staying without knowing what was going on in my body, it was to my advantage that when the hospital people came to do screening so i could benefit.*
15. I: What equipment did they use during screening?
16. *R: I wouldn’t know the type of instruments they were using. But they were putting something and a glass when screening.*
17. I: Alright. Why where they doing that?
18. *R: they wanted to know if I had viruses that causes cervical cancer.*
19. I: What kind of viruses s were these?
20. *R: I wouldn’t know because i was laying on my back,*
21. I: Alright! But they were saying you have viruses that cause cervical cancer?
22. *R: Yes they were saying that its a virus that causes cervical cancer*
23. I: Okay. So you said what was your result after screening?
24. *R: I was found positive for the viruses that causes cervical cancer, they applied thermo coagulation and I was told not have sex in six weeks. When they came for screening in our area they took a biopsy for testing here, so when i came here i was told that i no longer have the viruses because of the treatment they gave me during screening.*
25. I: Okay. What was happening during the whole screening process?
26. *R: At first they were testing our urine, because those who were pregnant, lactating, or those who have had an abortion before or those on menses were not allowed to get screened.*
27. I: Why were these people not allowed?
28. *R: I don’t know!.*
29. I: What influenced you to get screened?
30. *R: I did that because i wanted to know if i have the viruses/cancer or not. If i did not get screened that means the cells that were developing could have progressed to worse.*
31. I: Okay. Apart from wanting to know your cervical cancer status, What else made go for screening?
32. *R: i wanted to get early treatment, because we were told that if detected early, you can get treated on the spot. So that encouraged me to do screening so that i get treated early.*
33. I: Okay. Fine. Was there anything you were worried about before the screening?
34. *R: No, nothing!*
35. I: Okay, nothing?
36. *R: Yes.*
37. I: In your opinion!, what do you think went well or made you happy during the exercise?
38. *R:I was happy with the fact that they were able to apply thermo –coagulation to those suspected to have the virus at same point.*
39. I: Okay, What do you think did not go well during screening?
40. *R: When they were screening, I felt pain. It felt like they are pulling my uterus, this made me feel uncomfortable. But it wasn’t for long!*
41. I: In terms of what people were saying…
42. *R: Okay, what people were saying?*
43. I: Yes.
44. *R: Some people were saying that “cancer cannot be cured and when some heard that i tested positive, they thought i was going to die, and gossip about it.*
45. I: Okay. So when you heard that the results of your cervical cancer screening was abnormal, how did it make you feel? How did you feel in your heart?
46. *R: When they screened me?*
47. I: When they found that your results showed that you had tested positive for cervical cancer? How did you feel?
48. *R: I was worried, i started thinking that maybe my husband is having an affair with multiple women, and since people say its had to cure cancer, but through counselling i learnt that through thermo coagulation the cells that develop into cancer can die, and i was told that i will get help, so i became confident that i will be fine.*
49. I: Before you got screened what were people saying in your area?
50. *R: they were saying that those found positive for cervical cancer, means they have been married several times, since i had been married before.*
51. I: You mentioned about being married before, how many times have you been married?
52. *R: This is my second marriage, i was 4 days old in my second marriage when i went for screening.*
53. I: How did you feel just after hearing your results.?
54. *R: i accepted the results, because i realized that if i worry, it will not change anything.*
55. I: During the screening what did you appreciate that “This has been done well”?
56. *R: When they were burning off, I did not feel pain but when they inserted an instrument to pull the cervix so that they can be inspecting it; I felt a slight pain.*
57. I: What do you think they could have done better to easy the pain?
58. *R: i think there was nothing they could have done, because i think the pain was part of the process when getting treatment.*
59. Okay. Was there anything that you think went well during the process?
60. *R: No, this was the only part.*
61. I: In terms of the place where they were providing the service; how did you perceive it?
62. *R: Yes! Getting treatment at the same spot made me feel happy!*
63. I: Okay! Anything else?
64. *R: No.*
65. I: in future, what do think can be done to make others comfortable during screening procedure?
66. *R: i would encourage all others to go for screening, because its better to know your cervical cancer status, before its gets worse. All you need to do is accept and go for it*
67. I: How was the environment where the exercise took place?
68. *R: It took place at a nursery school where a make shift of the screening clinic was set up.!*
69. I: How about your privacy, do you think it was protected?
70. *R:It was fine, because it was just you and the doctor, no one could know the results of others, you could only see the papers that were given to bring to KCH.*
71. I: What about duration of the screening process?
72. *R: It took about 5 minutes to finish the procedure*
73. I: Was the time okay with you?
74. *R: It was okay with me.*
75. I: How did the health providers conduct the exercise?
76. *R: they were interactives, asking question and encouraging us.*
77. I: How was the care provided to you?
78. *R: After thermo- coagulation administering, they gave us six weeks sex break, so the wound can heal properly.*
79. I: How was your understanding on the six weeks sex break?
80. *R: It was fine with me*
81. I: How about male insolvent, how did you husband got involved?
82. *R: i explained to him about the results and the six weeks sex break, he accepted and said that we should follow what the hospital had said.*
83. I: Okay, fine. So what was the easiest part of the exercise?
84. *R: The screening part!*
85. I: How?
86. *R: The part where thermo coagulation is being administered.*
87. I: What was the most difficult part?
88. *R: The moment i felt like my uterus is being pulled out.*
89. I: Was there anything you did not expect?
90. *R: Yes. I wasn’t expecting to found VIA positive.*
91. I: anything else?
92. *R: That’s all.*
93. I: Did you have any challenges coming for this follow-up visit?
94. *R: Yes! The main problem is the distance from our area to the depot and transport money..*
95. I: Were there any other challenges?
96. *R: No!*
97. I: What other challenges do you think other women have that can hinder them from coming for follow-up visit?
98. *R: Long distance and monetary issues.*
99. I: What if there was money and the distance was not long, what can make one mii follow up visits.
100. *R: ” no answer”.*
101. I: Just imagine transport is there but you miss the follow-up visits*...*
102. *R: that will be laziness.!*
103. I: any other challenges you can think of that women face?, for example married women.
104. *R: “..........................”maybe the husband will not permit the wife to go for follow-ups.*
105. I: Any other challenges, What about community activities or problems?
106. *R: “no response”.*
107. I: You mentioned that you live very far from the depot, and that you had no money to come to the hospital, you came or you failed?
108. *R: I came.*
109. I: What else can we do to help you overcome the challenge of missing scheduled visits?.
110. *R: “....................” better roads and having a vehicle provided by the hospital to pick us from our areas, like they did, because people are afraid to lend you money, maybe because of the way you look.*
111. I: Fine. I wanted to know support that partners and the community provides; did you tell anyone about screening?
112. *R: I told many people like my mom, friends and anyone who asks.*
113. I: Why were you so comfortable to telling everyone?
114. *R: Because i accepted the results and i was happy with the treatment i received, and i thought i was wise that other women could benefit from this exercise.*
115. I: How about your partner?
116. *R: I explained to him, at first he kept quiet.*
117. I: Why did he keep quiet at first?
118. *R: I think maybe be he was sad about the news, he was not expecting,*
119. I:Did he say anything?
120. *R: He asked me, if the disease will be treated. I showed him the hospital papers, he read and encouraged me to continue visiting the clinic.*
121. I: So what does he think about your cancer screening?
122. *R: He asked about the screening procedures.*
123. I: Was he interested to know more about the exercise?
124. *R: Yes! Because he asked me all about the screening procedures*
125. I: Why did you discuss your results with him?
126. *R: I had to inform him about my results, because i was also told to not have sex for six weeks so he had to know, because he would want to have sex within the said period.*
127. I: Was there any other reason as to why you decided to share your results with him?
128. *R: I am his wife, i had to tell him..*
129. I: What did he say?
130. *R: He understood, despite that our marriage was still new.*
131. I: Was it hard for you to stay away from sex for six weeks?
132. *R: It wasn’t hard, though sometimes he could say that the viruses are gone because its been a while since i got treated and we ended up having sex after 2 weeks of screening*
133. I: Okay, How did this affected you?
134. *R: It affected me because we did not follow what the hospital advised.*
135. I: What happened to reach the point of having sex after two weeks?
136. *R: He insisted that the viruses were no more, and he wont manage staying for that long without sex.*
137. I: Do you think its important for male partners to be involved in cervical cancer screening campaigns?
138. *R: Yes, they should be supportive, taking their women to depot, provide transport and pocket money during follow up visits*
139. I: What are other reasons why men should be involved in this exercise?
140. *R: So they know their women’s health, because when they could be encouraging each other..*
141. I: In your opinion, what do you think should happen to make men take part?
142. *R: They should be escorting their wives to the hospital, so that when the doctors give advices, men should also be there, because its better for him to hear it for himself from the doctors than just hearing it from the wife, sometimes he may think the woman is lying.*
143. I: How do we educate men on the importance of being involved in these issues?
144. *R: For example, they he escorted me here today, that when you can have a talk with him about issues to do with cancer and the importance of male involvement.*
145. I: Is there anything new you have learnt about cervical cancer or cervical cancer screening that you did not know before?
146. *R: I have learnt that cervical cancer is real and that we should not ignore calls by the hospital to go for screening, because if detected early, then we can get help right away.*
147. I: What else?.
148. *R: That’s all.*
149. I: Okay. Who do you think is eligible for cervical cancer screening?
150. *R: woman aged between 25 up to 50,*
151. I: How often should women go for screening?
152. *R: Every year..*
153. I: Why do you say so?
154. *R:Because you might never know when the cells have started developing.*
155. I: I will go back to the other question about, who is suppose to be screened? What about those who are pregnant?
156. *R:During screening, we were told that a pregnant woman is not eligible for cervical cancer screening.*
157. I: Why?
158. *R: I don’t know, but that what they said.*
159. I: Alright so what happened when you got here?
160. *R:When we came here they didn’t screen us, they only told us about the biopsy result they took when i was getting screened that i no longer had cancer cells.*
161. I: what are the other tests that were carried, apart from the cancer screening?
162. *R:They did HIV tests and urinalysis.*
163. I: Why urinalysis?
164. *R: They wanted to found out if i was pregnant.*
165. I: So! what was happening after you get the results?
166. *R: We were just following what we were told to do.*
167. I: What was the results for urine?
168. *R: I was negative for pregnancy.*
169. I: and now?
170. *R: I am pregnant.*
171. I: When did you get pregnant?
172. *R: Same September, after we asked if it was possible to have a child after being found positive for cervical cancer.*
173. I: How did you feel when you leant about it after going through thermo coagulation?
174. *R: I was sad because, i was supposed to come for follow-up, that when i leant that i was pregnant.*
175. I: How many kids do you have?
176. *R: I have three kids.*
177. I: How old is the last one?
178. *R: the last one is five years old.*
179. I: I wanted to know about your future plans after being treated for cervical cancer and now that you are pregnant?
180. *R:I will still go for Cervical cancer screening in future.*
181. I: Any other plans?
182. *R: i will stop giving birth.*
183. I: why would you stop giving birth?
184. *R: Because i have enough children now.*
185. I: How many children would you consider enough for you?
186. *R: four is enough.*
187. I: How many boys and girls do you have?
188. *R: I only have boys.*
189. I: You only want boys?
190. *R: no i want all girls;*
191. *“all laughs”*
192. I: According to you, what do you think other women feel about cervical cancer screening?
193. *R: They want to take part, now seeing us that were found positive that we got treated and that we get transport reimbursements.*
194. I: Do you think they have more or less knowledge on cervical cancer?
195. *R: The don’t know much because they have never been screened, they only hear here and there.*
196. I:You mentioned that community were talking about you testing positive for cervical cancer, do you think there is discrimination in your area?
197. *R: no, there is not, because we could still chat with the community.*
198. I: are there other women who looks like there are at risk of cervical cancer?
199. *R: No!*
200. I:are there women who seem not to understand about cervical cancer screening?
201. *R: Yes! Some still dont come for screening, despite having doctors visiting for several days.*
202. I:Why do you think they chose not to be screened?
203. *R: i think they are just afraid about being found positive for cervical cancer.*
204. I: Okay! On that same note, do you think women are interested to get screened for cervical cancer and be treated.
205. *R: yes there is an interest because for us who were found positive, we came 9 for follow ups at first.*
206. I: What can hinder other women come cervical cancer screening?
207. *R: Sometimes its the misconception in the community, that can be discouraging then they end staying at home.*
208. I: anything else that they fear?
209. *R:Nothing*
210. I:What about married women?
211. *R:Sometimes husbands can prohibit their wives from attending screening for cervical cancer.*
212. I:Who else can stop them?
213. *R: “no response”*
214. I: What can be done to make more women go for cervical cancer screening?
215. *R:By sensitizing the community about cervical cancer screening*
216. I: Any other way?
217. *R: By advertising in the radios about the importance of this campaign.*
218. I:What about you? Can you encourage your fellow women in your area?
219. *R: Yes i can.*
220. I: How?
221. *R: i would tell them my story, because if i stayed at home not going for screened , it could have been worse, i would have gotten help early.*
222. I: Now, We will discuss about self-collected vaginal swab for cervical cancer screening. A new method has been developed for cervical cancer screening. It involves having a woman collect a swab from her vagina and submitting it at her convenience to a health facility like here.
223. *R: Yes.*
224. I: Yah, for testing. However, unlike VIA, the woman would not get her result immediately and would have to return to health facility to get her result a few hours later or the next day. What do you think?
225. *R: i think its not a good idea.*
226. I: What makes you think that way?
227. *R: Because you will not get an instant result and help!*
228. I: Yes, you will get help.
229. *R: Okay then its fine, because some people will be shy to let the doctors do the procedure.*
230. I: Would you be interested to do self- collected vaginal swab?
231. *R: Yes!*
232. I: What are the advantages of this method?
233. *R:”.......................” to those who are shy, then they can just do it themselves.*
234. I: What do you think are the disadvantages of this method?
235. *R: “ no response”.*
236. I: Do you think this is a reliable method?
237. *R: I don’t think so.*
238. I: Why?
239. *R: I think the sample can be compromised*
240. I: How can you differentiate the two methods; the one that you went through and the self testing one?
241. *R: i cant differentiate because i have never done the new method.*
242. I: But what do you think could be different between the two?
243. *R: Self collected vaginal swab is done by yourself and results delay, while VIA is done by the professional and you get result instantly.*
244. I: Fine. What do you think other women in your community would think about the self-collected vaginal swab technique for screening?
245. *R: they can find it easy to do..*
246. I: Do you think many women can choose the self testing method to screen for cervical cancer?
247. *R: Yes!*
248. *I: why?.*
249. *R: because its comfortable doing it yourself.*
250. I: What difficulties would women face in self-collection technique? You explained that maybe some are afraid to insert in the vagina to reach...
251. I: What could be other challenges?
252. *R: i feel like they can end up getting inadequate sample for testing*
253. I: What can be the challenge?
254. *R: [no response]*
255. I: Fine. What are some of the reasons, if any, why you think women may not want to the sample themselves?
256. *R: “............” i don’t know!*
257. I: You said that some women would prefer to be seen by a health provider...
258. *R: Yes. they would to be screened by a professional, get encouraged as well*
259. I: Fine. Now i want us to talk about your recommendations for the future of the National cervical cancer screening in Malawi: In your opinion, should MOH consider including self-collected vaginal swab for cervical cancer testing to the cervical cancer screening programme?
260. *R: They should adopt the method, if some have tried and they think its effective then they can adopt the method.*
261. I: Fine. Do you think this would make it easier for women to undergo screening?
262. *R: Yes it can be easy.*
263. *I: What groups of women do you thnk could be eligible for self-collected vaginal swab?*
264. *R: from 26 years old up to 50 years old.*
265. I: Why?
266. *R: because they are within the period of being able to give birth.*
267. I: What makes you think that way?
268. *R: that the only reason i had.*
269. I: So! Who is not eligible.
270. *R:[No responce]*
271. (all laughs)
272. I: anyway do you have anything to add?
273. *R: i have a question, since am pregnnant, hoe is it going to be?*
274. I:What do you think?
275. *R: They said you will tell me!*
276. I: Have you been given the results?
277. *R:They said from here i will meet someone!*
278. I:Okay, you will be told what to do!
279. *R: Alright!*
280. I: This is the end of our discussion, thank you so much for your time...
281. *R: Thanks!*
282. *End of interview.*
