# Supplementary material for: Perceptions of cervical cancer and motivation for screening among women in Rural Lilongwe, Malawi: A qualitative study
Source: PLoS One. 2022 Feb 7;17(2):e0262590. doi: 10.1371/journal.pone.0262590 (PMC8820632; doi:10.1371/journal.pone.0262590)
Supplement: S3 File — (ZIP) [file pone.0262590.s003.zip › VIA_245.docx]

**PID: VIA 245**

**DATE OF INTERVIEW: 19 October 2017**

**INTERVIEWER: 466**

**TYPE OF INTERVIEW: 12 weeks follow up**

**TIME: 59 minutes 15 seconds**

**KEY: I= Interviewer, R= Respondent**

**INTERVIEW SUMMARY**

Client reported to the clinic for her 12 week visit as scheduled. She was interviewed after her study procedures within Tidziwe premises. She explained that she never experienced any problems after thermocoagulation. She reported to be happy with the screening which was done in the community as it helped her to know the problems she has and reduced cost. She further reported that many women were not interested to be tested in the community because they did not want to be examined by the doctors and some felt that the program is satanic. However, others are willing to have screening. She further said that men should be involved in the programme because cancer cell are sexually transmitted so that they should be able to reduce the number of sexual partners they have. She did not have any problems with self-collection of vaginal swab for cervical cancer screening however, she felt that being examine by the doctors is much better because they are well trained on how to collect the swabs and they will also be able to identify other problems which woman may have. She feels this method will help a lot of women to be tested for cancer. The interview lasted for 59 minutes 15 seconds.

**TRANSCRIPT:**

1. **I:** thank you madam for meeting with me today. whatever you are going to say in this study is very important. I work with a team of researchers from UNC project Malawi. Whatever you are going to say is very important to us... so that it can enhance our understanding on how we can improve on campaigns that spread about the importance of cervical cancer screening. There is no right or wrong answer. Whatever you are going to say shall be confidential and used only for the purposes of this study and improving health work... *[Respondent murmuring on the background]…* I will record whatever you are going to say using this recorder so that it can help me to retain what has been said because I can’t remember everything said. Note that your name or any sort of identifier shall never be linked to whatever you will say. Feel free to say whatever you want to say and also your experiences.
2. R: Okay.
3. I: First of all I would like to know… huh… about screening… of cervical cancer. Can you tell me your understanding about cervical cancer screening… especially cervical cancer and the treatment you received?
4. **R**: *I can answer in this way. The time they screened me for cervical cancer...when they told me I have the germs (cancer cells). I saw that I could have died because if couldn’t meet the hospital team (referring to the team during screening) my survival was uncertain.*
5. *I: mmm]*
6. *R: I was very thankful and appreciative when they gave me a day of appointment to come for follow up visit here at the hospital. When I came here and got my results, I was happy because I knew I will be helped. With the help I received I see that my life is now better because at that time they burned the cancer cells and when I came here to get my results I was told that my womb was damaged in one side. So if I had stayed back in the village I couldn’t have known my status. Now I appreciate that I am going to stay healthy if I am going to face any problems then it’s going to be a different one. But as for that one is reduced..*
7. I: mmh mmh. What were the problems you were facing before you got screened for cervical cancer?...[cough on the background]
8. **R:** *Before I got tested for cervical cancer, I used to have stomach burns, all times my stomach would burn. So I used to attributed them to some of the problems I have had in the past*.
9. **I**: what were these problems?
10. **R**: *That time when I was pregnant I accidentally fall from a bicycle. So I delivered a baby with red skin on the head meaning I had it hit on the ground badly. so I think I also got myself injured from that time.*
11. *I: mh mh*
12. *R: So all along I have been uncertain about the condition in my body without knowing that I had other problems.*
13. **I**: mh mh.. what method did they use to test you?
14. **R**: *when testing us?*
15. **I**: Yes, when screening you…
16. **R**: *They came to our village with different equipment. So when testing us they said they had medicine which they spray and after they spray the medication the germs [cancer cells] get burn. However, they explained that they will still give us appointment dates to meet them at the hospital so that they can monitor the status of the cancer cells to check whether you are healed or should continue with the therapy up to the point when they are satisfied that we are completely healed.*
17. **I**: So where they screened you is it where they burned the cells (thermocoagulation) or what really happened during that time?
18. **R**: *the time they screened us they sprayed some medicine in the [vagina] and they explained to us that it was that medicine which kills those cancer cells.*
19. **I**: so how did they know that you had cancer cells?
20. R: *After they screened us since they have their own ways they follow... they have right equipment and we can’t really tell how it all worked but I am sure that they had the right equipment*
21. **I**: what did they do before they burned the cells (thermocoagulation?
22. **R**: Firstly, *when they came we went there and got registered, they gave us some papers to write on our names and asked us some different questions which we answered. They tested our blood, urine... and then we went in and lied on a bed and they had a thing like a bulb which they inserted me into the vagina. They used it in the way they know it and tried to search for the entrance to the inside of the womb. So they know that this is how one who has cancer cells looks and one who doesn’t looks like since they are trained. And all this was known by them as for us we depended on their report no matter the result… whether one didn’t have or is found with the cancer cells. So they worked for a number of days and they screened a lot of people in our community*
23. **I**: Alright. So what were your results because you explained to me that they wanted to know if people had cervical cancer? As for you what were your results?
24. **R**: *That time they told me that they found me with the cancer cells. They continued to say that "I should not worry it is curable as long as you follow what we (doctors) are telling you". so I understood and followed what they told me to come to the hospital for follow up. Today is my second time to come here and I can feel the difference compared with the past. The first time I came they prescribed some pills for me and I got them so I have been taking them up to this day of appointment and here I am.*
25. **I**: what else happened apart from spraying medicine on you and beaming on you with the bulb (VIA)?
26. **R**: *that time I didn’t feel anything unusual only the heat after the spray inside. So after that and when I started coming to the hospital to receive my treatment since I haven’t felt any problem*
27. **I**: Now I would like to know your ideas about campaigns… you know about campaigns, right?
28. R: mmmh
29. I: Yeah, campaigns about cervical cancer screening like the extent that...like how you were screening. Why did you decide to take part in this screening campaign?
30. **R**: I *wanted to know about the healthy of my life. I saw that it was a good development and that I had to take part so that I can know my status.*
31. **I**: Were you anxious of anything before you got screened?
32. **R**: *Nothing, I never experienced any problem. I discovered everything when I went there and got screened*
33. **I**: What did you hear before the screening took place?
34. **R**: *When the hospital staff came they met with the chief to mobilise people that one day health workers will come to our community to screen women for cervical cancer. So I thought that it was an honour by the health workers and I didn’t have to hesitate but to go so that I can also know my status. So I went to know the advantages and disadvantages of such things and now I am happy that I have a healthy life compared to the time I didn’t know anything. I would have been waning inside little by little without knowing only to be discovered too late when the womb is severely damaged. So I though it wise to go and be screened.*
35. **I**: were there any misconceptions spreading in your community about the cervical cancer screening?
36. **R**: *Yes they were there...some said it was not good for a woman to have her nudes exposed but as for me I saw that such stories were nothing and couldn’t help me in any way*.
37. **I**: is there anything else you remember that was said apart from being screened at the genital parts?
38. **R**: *Some people said it was satanic especially for some of us who were referred to the hospital and we would have our blood sucked...but I wasn’t convinced for it wasn’t my first time to seek help from the hospital. I have been coming here at the hospital several times with my child or be someone's guardian with blood shortage problem, all these times nothing unusual happened to me that’s why I was confident to come to receive help. They say all these because they have never been found in such problems or situations*.
39. **I**: How did you feel when you got your screening results and the fact that they were abnormal?
40. **R**: *It made me feel encouraged to participate in health related activities that come to our village*.
41. **I:** When they told you that we have find you with some cancer cells, how did you feel that time?
42. **R**: *Before they started everything they took us through counselling sessions; and this counselling session gave me confidence that I will get healed because who we are talking about here are doctors and they confidently assured us that cervical cancer was benign as compared to skin cancer. When the womb is not severely damaged it can be removed and one continue to survive with no problem at all. So I was ready and prepared for any result even if it would mean to come for removal of the womb*. [Interruption someone entered the room]
43. **I**: … So you were explaining...
44. **R**: *Yes...I was supposed to continue staying the way I used to because I cannot be the first person with this disease. So coming of the health workers is a sign that the disease is real and can annihilate people. So I can be among those people who can go and spread the news that I had the disease but now I don’t.*
45. **I**: So you are telling me that you didn’t have fear that time?
46. **R***: I don’t have any fears...and even now am not afraid i come here to the hospital with confidence to get treatment*
47. **I**: What exactly did you understand?
48. **R**: *yes I did*
49. **I:** How did you understand the meaning of the results?
50. **R:** *They said those who will be found with the disease should not worry nor despair because the cancer cells take a long time in the body to start showing signs and symptoms and discovering that you have the disease. So the time you start showing symptoms it’s when many parts have been severely damaged. So now we testing so that people can know their status early before the symptoms*
51. **I**: Alright, what is it that you are happy about during that process?
52. **R**: *when they screened me as compared to my friends who were also screened because they said they were discharging a lot of water and some even had some traces of blood but for me I didn’t see any of such signs and I was confident that the cancer cells have not fully developed.*
53. **I**: Now I refer to the whole process like beginning with the arrival of the people who came to screening test you... what really went on well?
54. **R**: *I am happy about the testing process because when they tested me I saw that everything was ok considering the fact that it has caused fear in a lot of people. and I was not afraid because I didn’t experience any problem.*
55. **I**: How was the screening place?
56. **R**: *they tested us right in the village. They were given a building I think it is a nursery school\… and they ensured all protocols of privacy and it was only "you" and the doctor no any other person watching or hearing the results. It was ending right in there.*
57. **I**: what about the time? How was it?
58. **R***: it was around past 11* AM
59. **I:** how did you look at the time taken for the whole procedure?
60. **R:** *as for time... I am not clear, what do you mean?*
61. **I:** Time... like you said it was past one, and again am interested to know how long it took to complete the process?
62. **R**: *when I got inside I didn’t spend much time though I didn’t have a time piece to determine the actual duration. But still you could spend some minutes for it was too involving... like screening you, take some pieces (biopsy) and did what they knew on us...*
63. **I**: how did it affect you that you took some time receiving treatment?
64. **R**: *I saw that I spent much time since the process was more interactive- I asked questions and so they did to me, and I saw that I took several minutes. With that said it doesn’t mean that I was not comfortable or that I was afraid no…we interacted just as you and me are now.*
65. **I:** Alright, what else do you think should have been done to make things better?
66. **R**: Aah, *I don’t know what else need to be done because we come here to get help just as they came to us. So mostly we look unto you hospital people you are the one who know better*
67. **I**: We want to know/get your ideas so that it can help bring improvement in the services that the hospital give you-to be satisfied…
68. **R:**  *when I came for follow up visit and told me that the womb was damaged on one side I asked them a question if they were going to remove it or they see that the problem wasn’t severe. So they told me it wasn’t severe and promised to put me on treatment. I went back home and started taking my medication according to the number of days they gave. Today is my second visit coming here… so if there can be any other problem or perhaps change of medicine then you are the one to tell me.*
69. **I:** Okay, Thank you. According to your explanation, what did you see as easy?
70. **R:** *As for me nothing was odd. Everything was just ok since they followed normal and usual procedures apart from the cancer screening part. Blood testing for HIV I have done before, urine screening I have also done that before-especially for pregnancy testing. So cancer testing was the only new thing to me.*
71. **I:** so, was it a hard thing to get screened for cervical cancer?
72. **R:** *No, it wasn’t*
73. **I:** Was there anything that you didn’t expect to happen during the time they tested you?
74. **R:** *Nothing was unusual*
75. **I:** Alright thank you. So after testing you had to come back to the hospital, It might be difficult for people to come back to the hospital for follow up visits. Was it a difficult thing for you and other people to come back to the hospital? Did you face any challenges to come back for the check-up visits?
76. **R:** *During the time they came to our village we complained about distance from the village to the hospital; and the schedules that we were given with regard to transportation costs it was a bit problematic. So the doctor said they would come and pick us up. During the first visit it happened but when we got here we were split into two-others were taken to Ethel Mutharika wing and some of us were assisted here. As for us who were here we got delayed till late hours so we were given transport and walked to Shoprite to get a bus. So we got there late and this time mostly transport fares go up than normal. Had it been that we were not given transport then we would have asked for some place to sleep over. So the biggest challenge is transportation.*
77. **I:** anything else*?*
78. **R:** *No the only problem is transport. If anything the only problem and if I don’t have transport money then I will have to give an excuse.*
79. **I:** Apart from transport, what other challenges do you think can hinder women to come for check up/follow up visits?
80. **R:** *Unless something unusual happens…*
81. I: Interrupts: Like what?*]...*
82. *R: Even when there is a funeral, we try our best to release such people who are in need of going to the hospital. So the main problem is transport otherwise the rest, nooo… may be if your own child dies, and then could you leave that behind and off to the hospital? Nooo, you can’t. So if the funeral is not in my household I would rather go to the hospital, my life comes first.*
83. **I:** Perhaps you can remember something that you would like to add on the problems?
84. **R:** *No*
85. **I:** How best do you think can we do to deal with the challenges that women face when coming for follow-up visits to the hospital as you have mentioned about money insufficiency?
86. **R:** *As for me there is nothing that I can advise since we are the ones in need of help. If I talk about money it will be like we are much interested in the money and this is wrong.*
87. **I:** It’s not going to be wrong we just want to get your views… whatever you are going to say it will help us to improve on the services. So feel comfortable and tell us everything that you think/know. Don’t be shy and think that if I say this. I will show that I love money. All we want is to see that you are getting the best services.
88. **R:** *The only thing I can say is for you just to increase the transport money so that it caters for all the involved costs. Sometimes we have to borrow money, so with random transport fare hikes we really need enough so that we can be able to give back money to the ones who borrowed us.*
89. I: Is there anything else?
90. *R: No*
91. **I:** Okay. Now I would like to about the support you get from your husband and the community. Did you tell anyone about your getting screened for cervical cancer?
92. **R:** *I am open, I tell people everything especially the ones who ask me. Even the chiefs, I told them. So I can tell you that everyone at my village know about my problem. I always want people to know what is good or bad so that I should not be responsible for any wrong choices they make. They should not die because I have hidden information from them.*
93. **I:** can you recall the people who you told about your problem (being found with cervical cancer).
94. **R:** There are so many people and *I can mention their names.*
95. **I:** No, don’t mention their names, just tell me the type of relationship you have with them.
96. **R:** *all my relatives know about my condition, I told them about what I used to feel in my stomach before*
97. **I:** So what did they say after hearing that?
98. **R:** *they appreciated for what I did and they encouraged me a lot; and I can assure that this was also important to those who have not yet gone for screening may be they were afraid of what people would talk about them. This will encourage them and may be reach to a decision of going for testing.*
99. **I:** Did they have questions?
100. **R:** *questions were there like how does this disease come to be. So I answered exactly the same way the doctor told me like through sexual intercourse.*
101. **I:A**apart from relatives, who else did you tell about this news?
102. **R:** *Some of my friends I chat with and I told them everything without hiding anything…*
103. *I: [*Interviewer interrupts:] You chat with…?.
104. *R: So some admired and longed to know their status too. So those whose communities didn’t get this chance they asked how they can access this opportunity and we have been asking doctors of the same. So they tell us if they have transport they can come but they will not receive special treatment as you do… so we reported that back to our friends…*
105. I: alright*… (door makes clinging sound on the background someone entered the room)*
106. **I:** You have explained that you have explained that you shared your results with your relatives, and friends. Who else? What about your husband?
107. **R:** *I separated with my husband so the time I joined this program he wasn’t around. So I didn’t want to tell him on the phone I will wait till the time we meet face to face so that if there shall be questions we shall clarify to each other right there.*
108. **I:** what makes you think it is important to discuss this with him?
109. **R:** *Since this disease is sexually transmitted he really needs to know so that he can abstain from sex. As for me I am getting my medication whilst him, isn’t. So if he continues, he will spread the disease to a lot of people*
110. **I:** any other reason?
111. **R:** *No*
112. **I:** after thermocoagulation of the cancer cells, you were advised not to have sex for a month to give time for healing. Was this hard for you?
113. **R:** *Not at all since am already separated from a man. From the time my man moved out, I have never had sex till today.*
114. **I:** Alright… do you think it is important that men should get involved in the cervical cancer screening campaign?
115. **R:** *they just told us that this disease is sexually transmitted but we didn’t ask how it is like when you continue having sex with a man after treatment. So the question that remains is wont he transmit it again to you.*
116. **I:** What am asking about is that should men take part in the cervical cancer screening campaign?
117. **R:** *I don’t know. Considering the way we were tested I feel it could be difficult for a man. So I don’t know what method they can use to test a man but as for us women we know because this disease attacks a woman on the womb entrance (meaning cervix)*
118. **I:** By taking part I don’t mean screening, but in all the processes and procedures even the support they can give you.
119. *R: Yes they should be involved*
120. I: Why do you think that way?
121. **R:** *Since it is a sexually transmitted disease, it requires a man who can exercise self-control for this disease I feel is not different from HIV/AIDS in the way it spreads. For I see that to get both of these diseases is through sex and to avoid both you need self-control-don’t have multiple sexual partners.*
122. **I:** What other ways can they be involved apart from remaining faithful and not having too many sexual partners?
123. **R:** *I don’t know other ways through which this disease is transmitted. Whether by borrowing razors or etc… (she missed the question)*
124. **I:** what about in other ways like accompanying you when coming for screening?
125. **R:** *that is very good and important.*
126. **I:** why?
127. **R:** *they should see how it happens so that they can learn that this is a problem. If one is ignorant about the importance of going to the hospital he can’t bother about treatment and this means you cant get cured. So if they can be coming and appreciate that if no research had been implemented it meant no ending of the disease and considering that they had to meet all the transportation costs and the problems at home… really they should be coming to learn a number of lessons from these.*
128. **I:** So how do you think can we encourage these men to take part?
129. **R:** *May be as for me it is just a matter of inviting him to come with me to the hospital… tell him all the issues involved. With that I don’t think he can hesitate*
130. **I:** Even if it wont take him coming but just to ensure his support in what you do about Cervical Cancer screening?
131. **R:** *for them to take part it can be hard… mmm, it can be hard, for most of the times when it comes to issue of testing there must have been a push factor… so, as for me… like how I came here… to take my husband it couldn’t be hard for he knows about the history of my health. I can just tell him and he is going to come. However, people understand things differently.*
132. **I:** So how can the hospital personnel take part in encouraging male involvement?
133. **R:** *like when they came to the village they should have made it open that men should also come with their wives. I believe a lot of them would have been attracted to come during the campaign.*
134. **I:** if we get to the village how can we teach men about cervical cancer?
135. **R:** *Follow the same procedures you did like going through the chiefs to mobilise men for you and have them sit down and teach them just like how you did with us. They should understand the problems associated with this disease*
136. **I:** I would like to test your knowledge about cervical cancer. Have you discovered/learnt a new thing about cervical cancer which you didn’t know before joining this study?
137. **R:** *I didn’t know my status and how my body was… so the coming of this study has helped me to know my status and I take it as a precious thing since a lot of people didn’t get such kind of an opportunity… [Sound of vehicle alarm on the background]*
138. **I:** what other new things?
139. **R:** *I didn’t know that my womb was damaged, I knew it that time… [sound of a vehicle alarm continues on the background]. So if I could not have taken part in this study, I wouldn’t have known my status*
140. **I:** Another thing?
141. **R:** *Those are the only new things I have known in my life*
142. **I:** Previously did you know the cause of cervical cancer?
143. **R:** *I didn’t know, I have learnt it the time that cervical cancer makes other pass out water and blood. Initially I would attribute such problems to other diseases without knowing what really it was.*
144. **I:** Alright, did you know how women could be protected from cervical cancer?
145. **R:** *I didn’t know and I didn’t ask about how one protect herself from the disease.*
146. **I:** thank you very much… did you know about availability of opportunity of getting screened for cervical cancer?
147. **R:** *No I didn’t know.*
148. **I:** what about treatment options?
149. **R:** *No I didn’t I was just continuing with my life without anticipating any of it*
150. **I:** So who do you really think must get cervical cancer screening?
151. **R:** *I think any mature woman since they told us about the age cut off.*
152. **I:** You have said they told you about the age limits for cervical cancer screening. So what was the age range or limit?
153. **R:** *they told us from 25 years to 50 years, these are the women who need to undergo cervical cancer screening.*
154. **I:** Alright what other categories of women are eligible to be screened?
155. **R:** *I didn’t get that type of information.*
156. **I:** Okay. how often do you think women should get screened for cancer?
157. **R:** *people who came to our village said they will come back after three years. But as for us we don’t know how long will it take to be tested again and further assessments… on that I don’t know. What I remember is that every three years one can have a test.*
158. **I:** In your opinion how long do you think should take to have a test?
159. **R:** *I think after a months*
160. **I:** how many months to be specific?
161. **R:** *it should be six to eight months*
162. **I:** what makes you think that way?
163. **R:** *I am saying this because after one has had a test and has not been found with disease, it doesn’t mean that she will remain like that without getting exposed to risks. There are a lot of things that people face and do. So at least getting tested a number of times would be helpful for behaviour change and reinforcement of that changed behaviour.*
164. **I:** Alright, what are your comments regarding future cervical cancer screening. What do women from your community think about screening for cervical cancer?
165. **R:** *women from our community say that they can’t do and don’t want to be examine naked in front of a man. But I say that’s nonsense it’s just limiting yourself from getting help. I consider it a greater opportunity to be approached by health workers… they cannot be out of their mind coming all the way from long distance with vehicles just to play and prank people. So I feel this screening is a good move.*
166. **I:** Anymore thing that women from your community think about cervical cancer screening?
167. **R:** *Some women ask because they are afraid of the equipment that is used. They ask about us feeling pain during the screening procedures, so we just answered them that there is nothing that we feel inside us. We didn’t even notice that they extracted some things from us (biopsy) we discovered about it when we came here for the follow up visits. So we tell them that there is nothing to be afraid of… if one is able to deliver a baby… so a baby with that metal as smaller as it is which one is dangerous?*
168. **I: Okay** according to your explanation, do you think they know about the process of cervical cancer screening
169. **R:** *Some have the knowledge through friends who initially got the testing before coming of your organisation, and its them who tell their friends about the whole issue… like me, I got the opportunity to know about this before from friends who during the time. I had to get screened they were saying that I will be inserted a metal… and this didn’t put me off I was bold enough to face it whilst to some it was their source of discouragement. As for us we tell friends that the metal isn’t dangerous at all, it was specially customised for that purpose and it can’t cause any danger*
170. **I:** what about discrimination, do you think women who get screened for cervical cancer get discriminated against?
171. **R:** *There is no discrimination, its just question they ask and we answer them about everything starting with the registration process where the doctor ask us question perhaps to know our history and all about the follow up visits.*
172. **I:** Thank you… may be, do they think that they are not at a risk of getting cervical cancer? What do they say?
173. **R:** *we tell them that with this disease they cant know that they have or they don’t unless they get screened.*
174. **I:** So, what do they say about their risks of getting cancer?
175. **R: T***hey say they are not sure about the risk-whether they contacted cancer or not. What they think… matters is survival. Otherwise they are only afraid or care a lot about getting undressed. Doubt should be there because one cannot be 50% sure since you and your husband have your own ways and where you go you know by yourself. As for me I came because I doubted about my condition.*
176. **I:** do you think that women from your community understand the importance of cervical cancer screening?
177. **R*:*** *Yes they understand and this is demonstrated through the attitude they are showing following what we tell them. They ask so many questions an are willing to undergo screening. So they regret about missing the opportunity in the first instance they say that: “had I known I would have joined”, so they send us to inquire for them… and when the doctors came they approached them to ask about what they can do to join. So they denied them still with comforting words that the door is wide open as long as they are ready to meet the costs of transportation by themselves*
178. **I:** So do you think by doing that they are showing interest in having screened for cervical cancer?
179. **R:** *There are a lot of them, at home people come may be 4 or 5 wanting to come with me to the hospital but I tell them exactly what the doctors told us. So we can let them to come to meet the doctors as long as they are prepared to meet the transportation costs*
180. **I:** what do you think can limit a person from getting screened?
181. **R:** *It is just because of ignorance and lack of knowledge. People are different in the way they understand things… we should know that hearing and understanding are two different things. So despite hearing about the matter they choose to act centrally, just because they didn’t understand.*
182. **I:** anything else to add?
183. **R:** *The second thing is like I talked about fears and shy of being examined. As for me I accepted it I know that I was born once and once I shall die. Different diseases are out there you don’t know which one will take you.*
184. **I:** is there anything more that can make them not to be interested in testing for cercal cancer?
185. **R:** *no there isn’t*
186. **I:** what challenges can women face in accessing treatment for cervical cancer?
187. **R:** *the challenges are the rumours that spread and misconceptions that it is satanic. But as for me I despised such reports and decided to have the screening and the chiefs encouraged us that there was nothing to be fear*
188. **I:** What hindrances from their husbands do you think can limit them from getting treatment?
189. **R:** *I think after the husband has forbidden you not to go, you should still go because after you die you wont take the husband’s life*
190. **I:** what about from other people?
191. **R:** *As for me I think it is better to go to the hospital and leave everything behind*
192. **I:** Okay, in your opinion what do you think should be done on cervical cancer campaigns to ensure that a lot of women are getting screened?
193. **R:** *As for me I joined because they found me right from the village. So considering the distances and locations of main hospitals it is very unlikely that women can make choices on their own to come unless they are approached and tested right there at the village. So this can encourage a lot of women to come for screening because they don’t feel sick. Otherwise they don’t see any reason to use their money just for screening*
194. **I:** So How can you encourage people to get screened for cervical cancer?
195. **R:** *I will be encouraging them with reference to the story of my life. Comparing how I was and how I feel now-so that when you come they will be compelled to seek help.*
196. **I:** now I would like us to talk about self-cervical cancer testing. A New method for testing cervical cancer has been discovered it is involving self collection of female fluids using cotton wool from the vagina and deliver it to the hospital whenever a woman has got time. However, it is different from the method that was used on you, this one you don’t get the results instantly… the woman is supposed to get the results after some hours or a day later. What do you think about this method?
197. **R:** *It is good development for those who are afraid of being screened. However it requires one who doesn’t get tired with walking… it is good it will give other people chances to screen for cancer and be able to know their status*
198. **I:** Okay, would you be interested to get screened using this method?
199. **R:** *Yes I can do…*
200. **I:** Do you think… apart from what you said that other people are afraid of using a metal on them, what other advantages do you find in this method?
201. **R:** *It is also good to those who do not want to expose their nudes to the doctor since they will do it with cotton by themselves and simply take it to the doctor. As for me I always feel it is important for the doctors to do the tests on me because it will give me an opportunity to know if I have other problems which doctors can discovered in the course of the examination. … So I feel self testing hides other problems which women may have. That’s my feeling.*
202. I: Mmh, what disadvantages do you see in this method?
203. **R:** *It doesn’t have any disadvantages, it is just ok.*
204. **I:** Do you think it is ok that it should be done at home?
205. **R:** *very important*
206. **I:** Why do you think in that way?
207. **R:** *Because it will bring the chances to be visited by the doctors right there in the villages hence reduced transportation costs. Hence there is no problem for me welcoming a doctor at home. Can you imagine how tiresome it is to wait on long queues at the hospital? It is effective to have them come at home…*
208. I: it is effective?
209. *R: Very effective and a thing to be proud of because there are a lot of people out there missing such kind of an opportunity.*
210. **I: Okay.** How do you compare this new method and the one that was used on you?
211. **R:** *The new method is good for those who are shy whilst those that do not care about being undressed in front of a doctor can use the one that was used on me. Hence as for me I prefer the one that was used on me because I was able to know about my cancer status and the other problems about my womb. So I feel being examined by the doctors is very good for you to know other problems that you may have.*
212. **I:** Mmh, in your opinion, what do you think other women from our community think about this new method of using cotton to collect specimen on their own for cancer screening?
213. **R: T***hey can think it is a good method to use unlike having being inserted some metal with someone..,*
214. **I:** What makes you think that?
215. **R:** *It is the report I get from them. They say: “they should insert a metal in the vagina like you are delivering while lying on bed…”, hence I guess they will feel it is good to do self collection of vaginal specimen.*
216. **I:** So do you think a lot of women can choose this method?
217. **R:** *Yes, especially the ones I said are afraid of being inserted metals (speculum examination)*
218. **I:** Alright… ok, thank you very much… [Silence for some seconds]… what problems do you think women can face on this method of self-collection of vaginal swab?
219. **R:P***problems can be there like I said understanding is different so may be one would insert the cotton in wrong position and hurt herself. Unlike when it is a doctor doing everything on you as they are trained and knows where to place those cottons. We don’t know that we have a number of holes we just take it for granted and hide on the pretext of nature.*
220. **I:** What are your fears about this self-collection of vaginal swab?
221. **R:** *My fears are that may be I can’t get the cotton to where I was expected to reach inside me. So I can get it and go for testing and perhaps the results can be that I don’t have the cancer yet I brought wrong fluid. While the other method you as doctors know where exactly where to place your equipment.*
222. **I:** what do you think can hinder you to use the self-collection of vaginal swab?
223. **R:** *I don’t know the right positions where to get the right fluid since I am doing it alone. May be am doing it with fear, as a result I can end up having the wrong results.*
224. **I:** What other reasons do you think can discourage women to use this self-collection of vaginal swab?
225. **R:** *It is the same reasons I have already given. Reaching on the wrong position and perhaps getting the incorrect amount of fluid as expected. As for us women some days you release a lot of vaginal fluids and some days just a little. So the fear would be, after I do the inserting of cotton by myself, have I done it correctly?*
226. **I:** Is there anything to add?
227. **R:** *No*
228. **I:** what makes you think would make a lot of women to prefer getting tested by a physician compared to the self-collection of cervical swab?
229. **R:** *because some people are afraid of being inserted a metal and that was the reason why I agreed. The thing is if the person is knowledgeable enough it can help.*
230. **I:** What do you mean by knowledgeable?
231. **R:** *Some people are cowards they don’t love their lives so they would could collect very little specimen if they do it themselves. So it is better she should be open enough and request for the other method that are performed by the doctors*
232. **I:** anything else
233. **R:** *No*
234. **I:** Now I would like to get your comments on cervical cancer screening. In your opinion should the Ministry of Health recommend this method of self-collection with cotton to be a method for screening cancer?
235. **R:** *Yes they should recommend it so that people can choose by themselves the method they are most comfortable with. It will reduce fears that women have on certain method whether being undressed before male doctors. So I feel more people will get tested.*
236. **I:** Do you think this can make women to be comfortable to go for cervical cancer screening?
237. **R:** *Yes it cannot be a problem because it will give chances to those that are afraid of the examination done by the doctors… since some do not understand that some of these things are just normal procedures.*
238. **I:** Alright, who are the type of women who can be most suitable for this self collection method for cervical cancer screening?
239. **R:** *Especially women aged 40 t0 50 who care about that. As for us young one we are dynamic and know the trends in health. So there are very few women of my age like 20s to 30s who might have something to do with such methods. We understand… we have gone to hospitals for different sorts of help, like HIV test, delivering children, etc… whilst older ones have grown up in an era where there wasn’t such kind of dynamism. So I feel these groups of people who are aged 40-50 are the ones who are normally shy to be examined by the doctors. They are afraid of the speculum examination.*
240. I: Okay. Which groups of women do you think will not be suitable for self collection of specimen?
241. R: Those who are 30 years and below can be able to do that but those who are old they don’t think properly. Normally people when they are aging they behave like children.
242. I: Okay. Do you have any questions?
243. R: No I don’t have, you have asked me a lot of question
244. I: Okay these are all the question I had for you. Do you have any comments?
245. R: I just want to thank the team for the screening I have gone through in the community. I feel protected now otherwise I feel I could have decided to go to the hospital when the cancer has already advanced.
246. I: Do you have any more comments\
247. R: No
248. I: Thanks you very much for accepting to be interviewed to day and for the useful responses which you have given to us. This is the end of our interview.
249. R: Thank you
