# Supplementary material for: Perceptions of cervical cancer and motivation for screening among women in Rural Lilongwe, Malawi: A qualitative study
Source: PLoS One. 2022 Feb 7;17(2):e0262590. doi: 10.1371/journal.pone.0262590 (PMC8820632; doi:10.1371/journal.pone.0262590)
Supplement: S3 File — (ZIP) [file pone.0262590.s003.zip › VIA_251.docx]

**PID: VIA 251**

**DATE OF INTERVIEW:**

**INTERVIEWER: 466**

**TYPE OF INTERVIEW: 12 WEEKS FOLLOW UP**

**KEY: I= INTERVIEWER, R= RESPONDENT**

**TRANSCRIPT**

1. I: thank you for meeting with me. Thank you for your time, whatever you are going to say shall be very helpful that we may know a lot from you. I am part of a team of data collectors for University of North Carolina. Whatever you will tell us is very important because it will help us improve on cervical cancer screening campaigns. there is no right or wrong answer; whatever you will say, will be confidential and used only for the purposes of this study and improve health services. I will record the discussion to help me get what was said. Note that I am not going to take your name or any identifier as such there is not going to be any link between what you say and your identification... I guess you understand
2. *R: You mean that in this discussion I should not mention my name?*
3. I: Mmm mmh, don’t mention your name... sure, sure... so can you tell me your understanding about cervical cancer screening and the treatment which you received?
4. *R: Yes*
5. I: Please tell me.
6. *R: In the beginning on 6th when the vehicle arrived, they said that: "we are researchers and conducting a study on cervical cancer" so we understood them and decide to approach them. So when got there they took urine samples and tested it, and then tested us for AIDS and finally they tested us the Cancer. They removed part of flesh and test it...*
7. *I: from where?*
8. *I: Right inside my cervix... and took it for testing and they told us that we shall get the results on the sixth and that is how we got here.*
9. I: When they tested urine what were they testing it for?... for you said they tested your urine, even blood... that was HIV I understood I guess and later they took part of your flesh. what were they looking for in the urine?
10. *R: Maybe they wanted to check if we were pregnant. Because they were not involving pregnant women*
11. I: Alright, after you came what were your results?
12. *R: They said that they have found what could cause or not*
13. I: Cause of what?
14. *R: Signs of cervical cancer... so they were telling us that I might start in the future or not*
15. I: Mmhh mmh. So you are saying they told you that the results indicated that you could start cancer or not... so for it not to start
16. *R: They did some heating with medicine*
17. I: So they heated you...
18. I: What else happened?
19. *The time they did the Thermos-heating?*
20. I: Mm... so you have got the thermos-heating here?
21. *R: When we got it there... we have also done it here. so the time I got the heating way back home I was draining some water... later they came to test another group so I approached them and explained to them that I am draining some water frequently. So their answer was that it happened because of the medicine which they used on me but they assured me that it said going to stop. Indeed, later on it stopped and no water came out*
22. I: How did you feel when you were draining water?
23. *R: I was afraid and thought I have contracted another disease. So I started believing in what people used to say when they came. So they were saying that they will suck our blood and they are satanic so a lot of people refrained and couldn’t come. but as for us we gathered courage and prepared for anything... just like family planning some say that I am not going to use them God is the one to control everything in me*
24. I: So you said you were afraid. What were thoughts considering all those rumors that surrounded you... you said that some said you will be sucked of blood... satanic etc... what were your fears?
25. *R: so what worried me was that when cancer spreads in gets inside into the stomach whereby they remove the womb... so in the process that you haven’t started getting sick, if they remove the womb, cant that be a straight way to death. so that was my worry... I will die without getting sick*
26. I: So are those fears continuing?
27. *R: At least better*
28. I: You say... better; what does that mean?
29. *R: That caused me to think a lot and my heart could pant rapidly... the time I was going to "Ethel" my BP rose.*
30. I: What made you go to (Name hospital), was it because of the BP?
31. *R: When we got here during the first visit we were told to go to Ethel Mutharika and there again we were told to come on the 3rd of (months). so when I came on the 3rd is when my BP rose... because they asked me if I was ready to be admitted, and I asked myself that should I get admitted just because of this disease, no for me. then I answered I am not ready for admission, so they told me to sit and wait... come back on the third so it when I was coming that I asked myself what I'm going to meet*
32. I: So you worried about what you are going to meet
33. R*: So when I arrived I was told my doctor wasn’t around hence I had to go back and come on a different day which they assigned to me*
34. I: Mmh mmm. So BP rose because of the issue that you will be admitted or it was a different issue?
35. *R: It was just thoughts*
36. I: I want to hear about the thoughts that’s why we are here... empty everything... [R laughs: hahhaha]
37. *R: I have never gone through operation so my fears were that if I am going to be operated on what type of operation is it going to be for there are different types... another one of a child birth they open... in the past they put you into deep sleep, I don’t know how they are doing it now. some say that they just inject you... so that is what worried me most*
38. I: So you thought you will be killed arrive... [both laughs]. Alright, alright. Now I would like to hear about screening campaigns especially the one which you went through. You said that some refused and couldn’t go. So why did you choose to go for screening and be part of the study?
39. *R: I thought it was wise to know the status of my body before I start showing symptoms*
40. I: anything else?
41. *R: I should receive faster treatment before the disease does great damage (before it spreads.*
42. I: Of all the things that people used to say that you be sucked blood... satanic etc... What made you bold enough to continue and go for screening? Apart from the two you have mentioned what else if you got any?... "..." was there any other reason that motivated you to go for screening apart from that you wanted to know your status and quick medication
43. *R: I reasoned that doctors cant come to us to kill us. Just like other family planning campaigns they visit us right here in the village. So appreciated that it was their strategy that they would visit us carry us and then bring us back. so I said whatever comes i am ready, better I go than regret later*
44. I: Alight, ok. Was there anything that made you worry before going for screening?... you have explained about other fears like water etc... But that was after. Now am talking about before you went for testing, what fears did you have?
45. *R :In my body?*
46. I: No, in your thoughts.
47. *R: I didn’t have any fears.*
48. I: Did you hear anything about the screening before it happened?
49. *R: No*
50. I: Oh so that was the first time?
51. *R: Yes*
52. I: Alright.. Ok. So the people who were talking, talked just after the arrival of the people?
53. *R: Yes... because this is how it happened when the vehicle arrived some tried to inquire what it came for. so by telling them it is here for cancer, and they said no, don’t just rush to join things... and I said I will go. they went on to say: "please not that, you may end up rushing for strange things"... [both laugh]*
54. I: So when you got the results of your screening and realized that it was bad? How did this made you feel?
55. *R: It made me realize my status fast. Had it been I relaxed and didn’t gat the screening by the time I could get sick, I couldn’t know it. so because we have discovered it early, that’s why I was able to rush here to the hospital and got the treatment*
56. I: After they told you that you had the cancer cells, didn’t your heart pant, and worried?
57. *R: Worries were there this disease is very dangerous*
58. I: Tell me about your worries
59. *R: What made me worry are the children i have. I thought that if they find me with the disease who is going to take care of the children some of them are in secondary schools being the one taking care of them as a parent I though it wouldn’t end well...if i start getting sick of this disease, how will the children get the care... so being a parent who takes care of them I said they shouldn’t stop schooling or even some activities stop at home. and this disease is incurable*
60. I: Did it affect the way you do your usual household chores?
61. *R: You mean how it affected me?*
62. I: Mmm
63. *R: When you are sick you cant work since you are in pain and sometimes because of anticipated disease you get discouraged and get weaker as such you cant work.*
64. I: Did you understand the meaning of the results you got?
65. *R: Yes I understood*
66. I: what did it mean to you? You said they told you that you might have or don’t, so what did it mean?
67. *R: "..." That was a difficult explanation. it just like you went to test for pregnancy and they tell you have or you might not have...*
68. I: So you said they told you that you have or you don’t have. so in your thoughts what did you think it meant by "you might not have"?
69. *R: Mmh, it meant the disease was there, now those signs were available. so i just accepted that I had it*
70. I: Alright. What do you think happened well during the time the tested you?
71. *R: What happened in my body?*
72. I: Everything, your body, how thing happened when you got there... what you think happened well, what was it?
73. *R: Screening*
74. I: Screening?
75. *R: Yes since we I have known what is going on in my body*
76. I: What do you think would have been done better? it was good but you think it could have been done better, what was it? "..." [door bangs]... what could have been better?
77. *R: Since this was my first time to test i have no standard to compare... that the way they have tested me now can go beyond this, i didn’t have that kind of knowledge.*
78. I: Yes its true it was your first time but am sure you were able to take note of the place, what they explained to you about the process and everything that happened... despite being your first time didn’t you notice something that was not ok? Not that you are comparing but just the intuition you have about circumstances... thinking that may be they would have done better...
79. *R: For the time I was tested and the time they screened me here its the same. when i came here they also inserted a metal inside me and then they said we will take again another sample of your flesh to see if the cancer cells are there or they vanished. so I saw it was the same*
80. I: So the way they welcome you and explained things was the same... [R: yes]... alright ok, what... mmm what part did you find easy?
81. *R: Simple part?*
82. I: Mmm
83. *R: Urine testing*
84. I: Urine testing?
85. *R: Yes... even HIV testing*
86. I: Oh not difficult? ... what was difficult?
87. *R: Cervical cancer testing*
88. I: How difficult was it?
89. *R: For a metal to enter into your genital parts is very painful*
90. I: Oh it hurt
91. *R: Yeah... since inside parts are very soft so for them to get you inside a metal mmm.... [both laugh: hahaha...]*
92. I:Mmh mmh, tell me more.
93. *R: I felt pain but not much pain.*
94. I: Was this pain just when they inserted you the metal or even when they cut you part of the inside flesh?
95. *R: Both ways the time they removed the metal and even the time they cut the flesh since a body is a body*
96. I: Was there anything that happened you idnt expect?
97. *R: Yes... didn’t expect to be found with cancer... I just thought i was ok yet i moved with it*
98. I: Ok. so... [sound of papers] "..." mmh so we will now discuss what happens after the screening. it can be difficult for other people too come back for follow up visits. did you have any problem coming back for follow up?
99. *R: Coming here?*
100. I: Yes... after they screened you they told you to come here on the six and again on another date
101. *R: Depending on where I come from it can be difficult sometimes to get transport. so the time I was coming I borrowed money; and when we got here lucky enough we were reimbursed all of our transport costs. then I was able to pay back where I borrowed*
102. I: Is there any other problem?
103. *R: No any other problem*
104. I: Which other problems do you think other women face and find it hard to come back here?... other women now...
105. *R: The other women... someone approached me had a problem of draining water so was asking me about it.*
106. I: What happens to her?
107. *R: She releases water which has bad smell*
108. I: What is it exactly (asked about the vernacular word)
109. *R: She releases vaginal fluid... so it is smelly. so she was asking "what really happens there", so I said initially these people come to us with a specific number of women they were looking for... about 4 hundred something... so if I take you with me you might be sent back saying that they can’t add any more. So she asked again that, "what can we do so that we may as well be assisted". Just told her to lay lol wait for us we inquire of it first that there are some who would like to meet doctors...... so may be that could be their opportunity... and thus the complaints that they have. Note that in the first place they didn’t want but maybe they were sent back because they were in period...*
110. I: Ok I mean those women who went for screening and there after they were told to come back to get their results. so am talking about those kind of women the problems they can have and make them fail to come back?
111. *R: There are no problems... but some like I said were sent back during the time we were been screened because they were in period so it is these women who ask us when we get home*
112. I: alright... ok... so... lets say some women were told to come back here on such such a day but they didn’t come. What do you think might have constrained them?
113. *R: Like someone we were together and openly told me that she will never come back again, and I asked why. So she said: "am afraid because I don’t know what these people would do us they can be a group of Satanists". so I am really not sure about what they thing since we come from different families and we value things differently as we talk. Yap that’s it indeed there was one among our group who said she will never come back here*
114. I: Another one you heard about?
115. *R: A lot of them*
116. I: What did they say was their challenge?
117. *R: They didn’t explain*
118. I: So what do you think should we do to motivate those who fail to come reach a point of coming? like you said about that woman, what can we do to make her come?
119. *R: We should be encourage them...*
120. I: How are you going to do it
121. *R: We will be telling them that "you should go there is nothing to be afraid we were there and these are type of treatments available... so the person will understand that for seeing us*
122. I: So you are going to encourage them?
123. *R: Yes*
124. I: What about us, what can we do?
125. *R: If you find chances you can be visiting us and encourage us as well*
126. I: Alright... alright... now we are going to talk about the support "we" can get from the community and people surrounding us so that "we" can be able to back. did you discuss with anyone about you getting cervical cancer screening?
127. *R: No... when the vehicle came I just told my husband that I am going for cancer screening and he said ok. Then after that I came back I reported to him again and he asked about the results so I told him everything so said ok fine. so as I said that I was asked if I was ready to be admitted and I refused since I was alone I didn’t know what could happen... so if am with someone and in the event of being admitted will be the one to take the message...*
128. I: So you have come with him?
129. *R: Yes*
130. I: Ok... so you said after you explained to him he said ok. now after you told him the results what did he say?
131. *R: He accepted, and said that it is good that they have discovered early, so be committed because this disease is deadly*
132. I: Apart from your husband is there anyone else you?
133. *R: Its my friend I talked about... the one I said she releases smelly vaginal fluid. so she was asking me about everything and as well i asked her why she didn’t join in the first place. so she said the time I came I was in period so I was sent back. so I don’t know if its fine for me to go there. so I said to her it is not up to me to decide that you should go or not... since you are not in the files so with in adequate transport you may end up being in trouble*
134. I: So what did she say when you told her that you got screening?
135. *R: With the way she understands she said she admired and loved if she got screened too*
136. I: So when you were telling your husband that health workers have come at school so and so... did you require his permission or you were just informing him?
137. *R: Since its marriage you inform each other, either he says go or don’t go...*
138. I: Meaning that if he said don’t go, you couldn’t come
139. *R: I am not really sure of the outcome but this is a matter of my life. so I think I would have done what is the best for my life*
140. I: When you were telling him, was he interested much to learn about cervical cancer?
141. *R: He was interested since he permitted me to go*
142. I: ... And when you came back he asked...
143. *R: Yeah sure*
144. I: Alright. After the thermos-heating of the affected area you were advised not to have sex for a month. Was this a difficult thing for you?
145. *R: To me it was not difficult but to the husband it was. he kept on asking me about it but he could understand because he is the one who in the first place allowed me to*
146. I: So after you explain to him what could he do?
147. *R: Nothing... [both laugh]... what else can he do*
148. I: Did he agree with it or because he didn’t have an alternative?
149. *R: ...Hahahaha, he accepted it*
150. I: You laughed...
151. *R: Since he is a person and I don’t go everywhere with him so I can’t know what he does when he is away. but as for the condition that we were in the house he understood*
152. I: Oh he understood
153. *R: Yes*
154. I: Do you think that men should be greatly involved in cervical cancer screening campaigns?
155. *R: Yes*
156. I: Why?
157. *R: We are together*
158. I: Why? You are together... [Some laughing on the background]
159. *R: The problems i will face will also affect him*
160. I: If they take part how is it going to help?
161. *R: When they take part?*
162. I: Mmm
163. *R: I don’t understand what you are trying to say*
164. I: like if they cant’ take part this is the problem... and if they take part this is the advantage...
165. *R: Ooh if a person has no business in escorting you to the hospital it means he wants you to die, but if he does it means he wants you to be healthy and in such a way he enlarges...*
166. I: If he encourages you how would you feel?
167. *R: If he encourages?*
168. I: Yes
169. *R: I will feel good since that shows that he wants me well*
170. I: So what role can men play?
171. *R: Encouraging me that I should be going to the hospital*
172. I: Apart from encouraging
173. *R: May be some days you may feel down and don’t want to go so it takes your partner to encourage you... that go and get treatment. Like I explained about our colleague who fall of didn’t want to come back that was a result of the discussions that they were having in their house. if one of them knew about the importance I believe should have been able to encourage her partner... so may be because in the village a lot of hearsays and such these build up fears like family planning issue a lot of rumors spread and this discourage others as such they keep on having as many children as they can*
174. I: So you mean men should be coming when they are screening you? during your time did he come with you?
175. *R: No he didn’t*
176. I: So you think should they be coming or not?
177. *R: I don’t know*
178. I: In your opinion what would you love?
179. *R: They should come since we sleep together*
180. I: So how can we encourage men to take part?
181. *R: Like us or you?*
182. I: I mean us since yours you already come with. I mean other men how can we encourage them so that they can also be taking part
183. *R:According to the way you were trained you know how to go about it*
184. I: It is you since the men belong to you... should be able to tell us that if you do this and that ... what can we do?... you can have school but men are a different thing and on top of that we are talking about your men... in your opinion what can we do
185. *R: It is the same you are also a human and can face the same problem. in your house how can you discuss this with your man?*
186. I: That’s my house... but now we are talking about you. How can you encourage/, motivate them?
187. R: I think... when you discuss with us invite them to sit behind us. So as we talk you can be talking to them and encourage them that they made a great choice to help us and emphasize that it is good that he wants us to be health. Go on to say that household with sick people has no peace. so if he was weak in some way he can get encouraged by hearing your words unlike my words-he can take them for grunted
188. I: oh ok
189. *R: May be am wrong*
190. I: Nothing wrong like i said in the beginning... whatever you saying is very important. Alright. is there anything new you have learned about cervical cancer or screening which you didin,t know before this study?
191. *R: What I didn’t know?*
192. I: yYeah… like about cancer, the way you can protect yourself or anything new you have learnt
193. *R: No... [I: so it was only those two you learnt]... since i joined whilst it started some time later so I don’t know what other things they learned before me. so its just like the ANC when you have come late you miss what others have learnt*
194. I: Like who do you think should get cervical cancer screening?
195. *R: Me*
196. I: You?
197. *R: Can you ask the question again*
198. I: Who do you think should get cervical cancer screening you can think of age, number of children one has, HIV status etc... Which group?
199. *R: Everyone disease can attack anyone they don’t choose*
200. I: How often should women get screened?
201. *R: How often...*
202. I: Like after how long after the previous screening?
203. *R: I can’t tell that should be well known by doctors. I am a client needing help I can’t know*
204. I: Yes that is true. however as a client if you count the days you have been given you to come, you might think that it would have been better if I was told to go and come on such such a date instead of such such a date... apart from that, doctor knows when you should come back. After how long would you love to get screening?
205. *R: Mmh... after a month*
206. I: Why choosing a month?
207. *R: So may be after screening you there might be some things which they didnt see. so that time should be enough for them to see those things that remained*
208. I: Mmm... is this monthly screening for everyone or those that were found with cancer cells?
209. *R: The one with signs of cancer cells after a month.*
210. I: Those who were not found with the signs?
211. *R: Can o it anytime*
212. I: What... about cervical cancer screening... in your area what do women think about it?
213. *R: Others accept that, but is good; while others don’t*
214. I: Those who refuse...
215. *R: We let them be*
216. I: Why do they refuse?
217. *R: They say it causes BP-"just like when you are found with HIV some commit suicide and their BP shoots". so they conclude that it is better they dont know before hand*
218. I: So they refuse in fear of hanging themselves?
219. *R: Maybe*
220. I: The way you look t it do you think they foresee any threat in screening?
221. *R: Yes I think they think so like what doctors would do to them*
222. I: Like what
223. *R: I can’t tell*
224. I: Do you think that women in your community understands the importance of cervical cancer screening?
225. *R: Some they do some they don’t*
226. I: So some don’t... who are many those who don’t know or those who understand?
227. *R: Those who don’t understand*
228. I: Why don’t they understand?
229. *R: I can’t tell what they think*
230. I: But what do you think makes them ones who don’t understand to be many?
231. *R: Like in our area they will be watching us since we come and go. so by asking us we will be telling them how good it is to come here. we will also tell them that this process is similar to family planning as such people will be motivated to come*
232. I: But where do you think this misunderstanding is coming from?
233. *R: Fears*
234. I: Afraid of what doctors will do to them or there is something else?
235. *R: I don’t know people think differently*
236. I: In your opinion, do you think that women are interested to get cervical cancer screening?
237. *R: Yes*
238. I: Why do you think they are interested?
239. *R: This disease is dangerous*
240. I: What do you think can make a person not to want to get screening? Apart from that he is afraid of being sucked or any other fears like that.... is there anything else?
241. *R: Its just because of ignorance in -spite of fears*
242. I: You said
243. *R: It the person's own lack of interest... ignorance*
244. I: What should we do for them to know?
245. *R: Should guide them... some sort of enlightenment, a teacher*
246. I: So are they afraid of the treatment or screening?
247. *R: They are afraid of screening. if she is screened and that there was need for treatment, how can she be afraid of the medicine she will continue to receive to get better*
248. I: What challenges can women face when getting treatment?
249. *R: Eeeeh, mmh..*
250. I: You have taken a deep breathe...
251. *R: You said that women should...*
252. I: What can hinder women... challenges that women can face when getting treatment
253. *R: At home or hospital?*
254. I: Anywhere you can either start with home. What challenges are there at home?
255. *R: There are different problems in the village maybe one has no food, or money for transport. Or maybe want to find some piece work to make ends-meat in order to get something for the kids. so all those things are a limit*
256. I: Not only coming here... but when the vehicle comes there, what can make other women not to come and participate? In that case we have ruled out transport since the clinic has visited you, what can be the problem?
257. *R: Like I said initially that ignorance and personal choice. so you can approach a friend and say hey, there is vehicle... will say uh, "go I am not going". May be sometimes without a valid reason you find them failing to come*
258. I: Or can it happen because of loved ones or other people?
259. *R: Yes it can because other people accepts what others say and yet the problem is with you*
260. I: In your opinion, how should the treatment of cervical cancer be given in order to ensure that a lot of women are screened?
261. *R: We should be encouraging them*
262. I: Encouraging them?
263. *R: yes for are the ones who stay there and are closer to them than you are .so we should be the first ones to spread the good news and you come after us after we have already guided them*
264. I: So how would you encourage them?
265. *R: After I they come and ask me that oh you back, will say yes am back. "How did it go" and will say everything just fine what about you? an then tell her that you should go for screening for that disease does not immediately start; it takes time for it to start showing signs and symptoms. So it is better to know how your body is. With what you tell the person if she understands then she will decide to go*
266. I: This was according to situation that one has asked you about your visit. What if someone didn’t ask you, how can you encourage them?
267. *R: There are times when you chatting with friends and you are faced by a chance that you are talking about that disease. so with that opportunity you start telling them that women we lagging behind its better that we follow such method... by that you give chances to others to know*
268. I: So you mean that when you are in your groups you do discuss about cancer?
269. *R: Yes we do*
270. I: So what do other women say/
271. *R; Others say that, "ts good others say it isn’t why should you about how you are going to die? this disease is dangerous it is incurable so is it good to know about your death-there is no medicine people die"*
272. I: So you saying they say cervical cancer is like death... do you also discuss about the causes?
273. *R: No we don’t- we don’t know about it*
274. I: What about its prevention?
275. *R: I don’t know*
276. I: Now we will talk about new method of cervical cancer testing. this method is involving a woman extracting vaginal fluid using cotton wool and deliver it to a hospital for testing at her time of convenience... are you getting me?
277. *R: Repeat*
278. I: Ok am saying now we discuss about self-testing. This method involves taking cotton and extract vaginal fluid samples from the cervix and deliver it to a hospital for testing at her time of convenience. So the difference between VIA (VIA" is the method which thy used on you) and this method is that you will not be getting the results at the same time. You have to come back to the hospital after some hours or a day after. You understand... So what do you think about this method?
279. *R: Didn’t it get dry*
280. I: What did you just say...?
281. *R: ... A lot of people can’t choose it*
282. I: What makes you think so
283. *R: only if a woman comes from home and take the cotton and extract the fluid right here that could be possible... Mmm... but not that tomorrow she is going to the hospital and she takes the cotton today and bring it tomorrow that wouldn’t work...*
284. I: Or let’s say... smell?... like the way you have come, you take cotton and extract the fluid and take it to the doctor or nurse... assuming that it is happening right here, what do you think about it?
285. *R: It is better*
286. I: Its better than the one you used
287. *R: Aah, its not... the cotton method is better*
288. I: How good is it?
289. *R: I have just answered*
290. I: You have used a different method before, now there is need of introducing a new method the one we just talked according to the way you understand-that instead of the doctor taking the samples on you, you are doing it alone by using cotton and give it to the doctor...
291. *R: Will you get the results same time?*
292. I: No, but the other one could get the results instantly
293. *R: So the one you get results same day is better*
294. I: Why?
295. *R: Since you will get the results faster*
296. I: So the other one with no instant results, how good is it? or how bad is it?
297. *R: It not good*
298. I: Tell me how bad it is?
299. *R: Increases level of anxiety since you are still uncertain of the results*
300. I: Ok. what is its advantage?
301. *R: It is not painful inside "here"*
302. I: Any other advantage?
303. *R: No*
304. I: Ok, do you think it is reliable or not?
305. *R: It is not reliable because there will not be samples of flesh where cancer cell are found... so just relying on vaginal fluid would be reliable*
306. I: So it is not reliable because they are not taking part of fresh for screening?
307. *R: Yeah, they have to take the flesh and other samples by the other method... so here you are just giving one sample and no screening of flesh*
308. I: According to your choice, which method is better?
309. *R: Since I have never used the one we are talking about, to me the one that was used on me is better*
310. I: Better is the one you already used
311. *R: If I had used both then I should have been able to compare...*
312. I: But as for now...
313. *R: Better is the old one*
314. I: What do you think can women from your community think about the conduct of taking vaginal fluid by themselves using cotton?... you said you prefer the other method. what about other women, what can they think about it?
315. *R: They will choose the cotton method*
316. I: Why/ what do you think can make them choose this one?
317. *R: Fear*
318. I: Fear of what?
319. *R: Metal*
320. I: Another thing?
321. *R: Some are afraid that they will be inserted a metal in their stomach... so you become afraid that you will start feeling pain may be they have forgotten to pull it out*
322. I: Ok... so many women can choose this cotton method?
323. *R: I am just being hypothetical*
324. I: So what can women think about the conduct of taking their own vaginal fluid using cotton?
325. *R: ... "..."... We perceive things differently... may be some may not be comfortable with it... but as for me its fine since it is your own body... there are different things in the body... so your own body you can’t be uncomfortable*
326. I: Alright, what are the problems they can face in using this method? Not being comfortable, any other problem?
327. *R: "..."... At the village?*
328. I: Mmm
329. *R: We are going to repeat what has already been said. Transport problems some may be because of misunderstandings with the husband... those are also problems*
330. I: Ok. what fears do you have that the woman should be testing herself?
331. *R: Nothing*
332. I: Alright "..." there might some women who may opt to be screened by a doctor and other may choose to use this self-testing method. what do you think can make women to choose to be screened by a doctor unlike to self-test?
333. *R: I don’t know what they can think... since we think differently*
334. I: I just want to know how you think... why would they choose to be screened by a health worker unlike this cotton method?
335. *R: May be because of fear*
336. I: Fear of...
337. *R: Afraid that may be, "I will over insert the cotton and it will get stuck". so seeing the doctor is the best option*
338. I: Ok now let’s see at the comments you have about the future of cervical cancer screening in Malawi. In your opinion, do you think the ministry of health should include the method of self-testing with cotton to be part of cancer screening methods in Malawi? do you think that it should be added?
339. *R: Should be merged with this one?*
340. I: There is already another one, so with the one we have just talked about do you think it should be added among the cancer screening methods?
341. *R: It should be added*
342. I: Why?
343. *R: It the same method.... to increase person's choice. one should be able choose the method she is comfortable with. for you shall be asking that should we give you this method or that? so an individual should be able to choose*
344. I: So they should add so that people can choose as compared to now
345. *R: Yes... it will be ones freedom to choose the method*
346. I: So now do think people do not have the freedom?
347. *R: How can there be freedom with one method can you say I want such method? no you can because there is only one... the cotton one has not yet been introduced*
348. I: So you think that this can make it easy for women to get screened?
349. *R: Yes*
350. I: So it can be easy, why?
351. *R: It is simple*
352. I: It is simple?
353. *R: Mmm... simple can there be some who can say I won’t go because of fear I doubt... maybe there are other things I don’t know, but as for just taking cotton and show it...*
354. I: Alright, which groups of women would be suitable to use this method of taking vaginal fluid?
355. *R: I don’t know.*
356. I: There are different types of women. those like professionals, business women, who stopped giving birth... which group do you think is suitable?
357. *R: The ones who are still bearing children*
358. I: The ones who are still bearing children and not the others?
359. *R: Mmm... the others stopped but the one who is still bearing children is the one facing the risk*
360. I: Which one are not suitable?
361. *R: All of them are suitable*
362. I: Hahah... you have changed -why have changed?
363. *R: Everyone wants to survive*
364. I: Which should be the groups... you say everyone wants life
365. *R: "..."... All groups*
366. I: That’s the end of our discussion... any additions
367. *R: Should add on that?*
368. I: Yeah what you would like to add or comments
369. *R: I would like to say that the organization should not stop visiting us in the village... should continue giving counselling about this disease, for one can’t say that I don’t have the disease may be you have the cancer cells*
370. I: So what do you think will be the advantage of continuing?
371. *R: People will be able to know their status whether they are ok or not. so you will be given medication that burns the cancer*
372. I: In your opinion, if the people visit your village again do you think a lot of people will come or not?
373. *R: Mmh, they can go again*
374. I: A lot or few? Women to be screened, will they be a lot or few?
375. *R: There will be a lot?*
376. I: Why?
377. *R: They are watching us that we are going back home which is very different from what they were thinking... we come and go back without any problem so they will say no we just get frightened for nothing*
378. I: Anything
379. *R: They should be helping us*
380. I: In what way?
381. *R: We come from home we have nothing... so the transport they give us is not enough considering the distance*
382. I: Alright thank you for your time
