# Supplementary material for: Perceptions of cervical cancer and motivation for screening among women in Rural Lilongwe, Malawi: A qualitative study
Source: PLoS One. 2022 Feb 7;17(2):e0262590. doi: 10.1371/journal.pone.0262590 (PMC8820632; doi:10.1371/journal.pone.0262590)
Supplement: S3 File — (ZIP) [file pone.0262590.s003.zip › VIA_269.docx]

**PID: VIA 269**

**DATE OF INTERVIEW: 09 November 2017**

**INTERVIEWER: 466**

**TYPE OF INTERVIEW: 12 WEEKS FOLLOW UP**

**TIME: 48 min 37 sec**

**KEY: I= INTERVIEWER, R= RESPONDENT**

**TRANSCRIPT**

1. **I**: thank you for your time today whatever you are going to say is very important. I am working with a team of researchers from University of North Carolina normally called UNC project. Your input is very important to us because it will enhance our understanding on how we can improve on campaigns that spread about the importance of cervical cancer screening here in Malawi. There is no right or wrong answer. As such don’t worry that you might give a wrong answer. Whatever you are going to say it is going to be private and confidential. It will only be used for the purposes of this study and improving health projects. I will record the discussion using this device so that I can be able to retrieve the information after the interview. No information of your identification shall be linked to what you shall say. First of all I would like you to tell me your understanding about cervical cancer screening which you got 10 and 2 weeks ago… Like 12 weeks not so? What is your understanding about that screening?
2. **R**: *they identified cancer signs on my cervix and they sprayed some medicine and said I had to take six weeks without having sex with my husband. I followed the instructions because I accepted the fact that I before screening I was ignorant about my condition. So telling me that they have found some cancer cells inside my cervix I felt that it was very important and worthy following the advice given to me.*
3. **I:** What method did they use when screening you?
4. **R**: S*ame equipment they used me today*
5. **I**: what equipment is that?
6. **R**: *you mean the equipment... metals, machines*
7. **I**: *... Mm mmh mm,* so what were they doing exactly?
8. **R**: *They were inserting them inside here.*
9. **I:** Mmh mmh (encouraging her to talk more)
10. *R: So they were inserting it in here (Meaning inside the vagina). When they insert the metals they were able to see the problem but rather the disease inside my stomach, yeah. So when they see the disease in my stomach with the metals they inserted the metals inside my vagina so that they would do their work according to the knowledge they have.*
11. **I**: Alright, so what were your results after screening?
12. **R:** *they said I had signs of cancer... yeah and if not treated I can have cancer.*
13. I: Mmh mmh
14. *R: So when we came back on the 6th of (month) that should be on the 6th of August they told me that according to the samples they took after the therapy it shows no signs of cancer. So they said I should come back on the 19th of (month). So before the 19th (the community educator name withheld) came home like on the 15th and told us that the names who have to come to the hospital tomorrow are as follows... so they called other women-about two of them and said: "as for some of you your dates of appointment have changed. So only two per people will come on 19th considering the long process " So and so (names withheld) are supposed to come on the 2nd of November, that is why we are here today.*
15. **I:** Okay. Apart from screening what other activities/procedures took place?
16. **R**: *Water came out*
17. **I**: No, apart from the test they conducted on you, what other things happened that day?
18. **R:** I*t was a long process, after initial test we were taken to one point after another...*
19. I: [interrupts) What was happening on the other points?
20. *R: They were asking us questions*
21. **I:** So what type of questions?
22. **R***: It was about the result of our testing. So they were counselling and encourage us to go back home with hope*.
23. **I**: Alright Thank you. Now would like to get your opinion about cervical cancer screening campaign-the screening which you received. why did you choose to get screened or to be part of this study?
24. **R**: *I wanted to know the condition of my body, I was just staying ignorantly without knowing that I am having a problem in my body...*
25. I: Mmh mmh
26. *R: So thats why I volunteered myself... [Sound of a song on the background; and small pause]*
27. **I**: We were discussing why you chose to be screened for cancer and you said you wanted to know the status of your body...
28. R: mmm, for *me I felt that it was a very precious thing to know and being visited by doctors in our village despite the talks by other people who were saying that why should I be inserted metals into my genital. So to us that wasn’t a problem because we knew that we are women who give birth and from our genital comes out a very big thing more than a metal which is a child. So why would it be so hard for me to be tested for cancer and know my status. Hence we saw that it wasn’t an issue at all.*
29. **I**: Was there anything that made you worried before the screening process?
30. **R**: *Not at all*
31. **I**: what is that you heard about cervical cancer screening before you got screened?
32. **R***: Before health workers came to our village?*
33. **I:** mmh
34. **R**: I *heard nothing*
35. **I**: You heard nothing?
36. **R**: M*mh*
37. **I**: Alright were there any rumours and misconceptions about cancer screening in your village?
38. **R***: Yes people were talking a lot of things.*
39. **I**: What were they saying?
40. **R**: *They were saying: "would you really go there to be tested for cancer, what are your problems? It means you doubt yourself” They said so many things. So we realized that what they were saying was useless, they could not be smarter than health workers.*
41. **I**: What other things were they saying?
42. **R**: *They were saying since I have cancer signs they will deliberately Make us to be going to the hospital so that they can be sucking my blood and eventually I die. So I said no, that cant happen they have come to help us, how can they do such a horrible thing?*
43. **I;** Alright, so when you got the results and see that they were not good how did you feel?
44. **R**: *I was happy to know that I have the problem because in the past I considered myself to be health which dangerous. But knowing my results I felt it was good.... I have the opportunity to get treatment.*
45. **I**: How did you feel when they told you that you have cancer cells?
46. **R***: I was anxious and afraid since I knew that cancer is a deadly disease and has no cure. So I said to myself that my life is at risk what can I do... so I just recalled to what the health workers told us to follow their instructions and get treatment.*
47. *I:* Mmh mmh alright. Did you understand what it meant after they screened you?
48. **R**: *yes, not having sex..*
49. **I:** What exactly did they say?
50. **R:** *We understood because they said we should not quickly indulge in sex activities for the reason that they have cut a portion of the flesh which had cancer cells to do further assessments. So we don’t need to have sex with a man they might hurt you on the wound.*
51. **I:** alright...mmh alright... so they explained to you that you don’t have to have sex?
52. **R**: *Yes they did and even today they have said I should let 7 days to pass without having sex because today they have again taken samples of my fresh on my cervix*
53. **I:** Alright, thank you very much. So with that how do you feel in your heart?
54. **R**: *I am very happy because cancer is deadly as compared to AIDS... I wanted to know about my status and how I can continue with my life*
55. **I**: alright. What do you think happened during the screening process?
56. **R**: *As for me what really fascinated me that time was the fact they found me with the disease whilst in the past I was just staying ignorantly. So with this initiative I was able to know about my status and gave confidence that I will have the opportunity to get treatment.*
57. **I**: So what can you say went on well?
58. **R**: *I now know my condition unlike in the past. i didn’t know what was happening inside my body.*
59. **I**: So after they screened you, were you happy?
60. **R**: *Yes because now I knew what was in body*
61. **I:** what other good thing did you notice?
62. **R:** *being visited by health workers and asking us to work with them politely and making it clear that it wasn’t compulsory to be screened. They said that those who don’t want should not come. So I responded positively and approached the doctors*
63. **I**: alright. What do you think could have been done better?
64. **R**: *what I think could have been done better?*
65. **I**: mmm
66. **R***: It is the same thing i am saying that we value their coming to our village... we were just staying thinking we were ok not knowing that we had a problem*
67. **I:** What do you think didn’t happen well?
68. **R**: *nothing*
69. **I**: Alright what do you think happened... oh sorry... what was the most difficult part?
70. **R*:*** *The part which they were cutting us part of our fresh. like I said they were inserting metals inside us so being the first time it was a bit difficult and could cause some pains*
71. **I**: How painful was it?
72. **R**: *They were inserting metals inside us...*
73. **I**: Oh ok... alright... [Door opens and closes voice of apologetic woman on the background]... you have said about difficult thing. is there any other difficult part?
74. **R:** *No there wasn’t*
75. **I**: What about the part that was not hard?
76. **R**: *There wasn’t as well*
77. **I:** So we can say all you had were difficult situations?
78. **R**: *Yes it was difficult... [I laughs: mm hahahaha]...*
79. **I**: So everything was difficult right...
80. **R:** *Yes it was difficult... but I can’t say it was difficult since they wanted to help us so they had to go through the required processes*.
81. **I:** Alright... so can be difficult for you... especially on your side you have said it was difficult, was there anything which you didn’t expect on that day?
82. **R:** *What I didn’t expect was the cancer which they found me with otherwise I was curious to know my status whether i had AIDS or not. But as for this kind of disease I didn’t expect*
83. **I**: so that time they told you to come back. I believe it can be a difficult thing to come back for the follow-up visits. Did you have any challenges coming back for the follow up visit which you were told?
84. **R:** T*here wasn’t a problem because Mr (name withheld) came to pick us because initially we told him that we could not manage to come we are poor... we cant get transport. so you might think that we have betrayed you. So he said we shouldn’t worry he is going to come to pick us. So he came and pick us*
85. **I:** what other problems do you think other women who cannot be picked can face and limit them to come for the following visit?
86. **R**: *transport*
87. **I:** Mmm mh, anything more?
88. **R**: T*hey don’t have the permission to come... like now a lot are saying if I had joined from the beginning I could have known the problems I have and be able to be coming to the clinic as you do.*
89. **I**: I am talking about women who got screened now they are failing to come here... the first problem you have said transport, what can be another problem?
90. **R**: *their own unwillingness*
91. **I:** Another thing?
92. **R**: *Misconceptions that when we come here something bad will be done to us*
93. **I:** what kind of a bad thing?
94. **R**: *I don’t know... [Both laughs.]*
95. I: What do they mention about this bad thing?
96. **R***: They say you will sack us blood why should we go to the central hospital as if there is any pain anywhere? so we said as for us we will go.*
97. **I:** So what do they say the blood is for?
98. **R:** *they say it’s satanic*
99. **I**: oh ok satanic... mmh mmh… Alright, apart from that what else can hinder women after screening to come back for the follow up visit?
100. **R**: *I don’t know what they can think... (Gives a chichewa ideom: "phukusi la moyo sakusungira ni wina umasunga wekha" Meaning you have to care for your own life insteas of relying on other people}*
101. **I:** With regard to what you have said about lack of transport, women's own unwillingness to come back for follow up visit and being discouraged by people, how best do you think can we help to eliminate the challenges that limit them from coming to the hospital?
102. **R*:*** *You have to go back and encourage with us as models*
103. **I;** Is there anything else?
104. **R**: *No*
105. **I:** Do you have any other idea as to how we can do to make women come back to the hospital for follow-up visits?
106. **R:** *It is not difficult*
107. **I:** What do you think can be done to make it simple for women to come back for follow up visits?
108. **R**: *Mr (name withheld) told us that if we don’t have transport we can borrow and they are going to reimburse after we get here. So I dont see any other bigger challenge than transport.*
109. **I:** Alright. I would like to know about the support you get from your husband and the community. did you discuss the issue about your screening with anyone else?
110. **R:** *Yes*
111. **I**: who did you talk to?
112. **R:** *I talked to friends that even though you didn’t test for the cancer you hated yourself. At least a person should know the status of his body if you think we are mad one day we shall be smarter after you die with your ignorance... when things of health like these have come to our community jump in quickly for the problem that was found in us... we never expected that we can have it*
113. **I:** Apart from your friends... or lets say you have explained to your friends like you did, what were they saying?
114. **R**: *They were saying it’s none of their business*
115. **I:** what do you think made them say that?
116. **R**: *We don’t know*
117. **I**: Who else did you tell apart from your friends?
118. **R**: *My husband*
119. **I:** How did you tell him about it?
120. **R:** *“I said my husband we were visited by health workers who were testing women for cervical cancer and HIV. I also took part in the test and the results show that I have cancer cells but HIV I don’t. So they treated me and took a sample of fresh from the infected area and they have given us dates to go to the hospital for follow up. In addition with that cutting of flesh means we can’t have sex till am certified that am ok according to the number of weeks we have been given". He understood and nothing was difficult with him. And Mr (name withheld) came to pick us up and after some time when we came here they told us that we were free to enjoy sex with my husband.*
121. **I**: what did your husband say?
122. **R**: H*e was happy because he also though I was ok just as i used to*
123. **I**: How did he express his happiness?
124. **R**: *He said it was good that the health workers came to our community without them we would have continued our lives without know our problem*
125. **I**: What type of question was he asking?
126. **R: He** *didn’t s any questions*
127. **I:** So what do think is he thinking about cervical cancer?
128. **R**: H*e is also afraid and anxious if I am really going to be healed for now that hope isn’t clear*
129. **I:** What makes you think that way?
130. **R**: That the disease will end?
131. **I**; yeah
132. **R**: *Since we are still coming here and up to date we haven’t yet gotten a tangible report that the disease is gone. We will wait for the main report from the doctors here that the disease completely healed*
133. **I:** Alight, thank you very much. What type of support do you expect from your husband?
134. **R**: *I expect him to provide for all the three meals breakfast, lunch and supper but since he is poor he can only manage lunch I understand him*
135. **I:** I mean about you coming to the hospital... about the screening you got, what support do you expect from him?
136. **R:** *from my husband?*
137. **I**: Mmm
138. **R**: C*oming to the hospital together as we have done (she came with the partner)*
139. **I**; What else?
140. **R:** *Nothing*
141. **I:** What if you don’t come together?
142. **R**: *No problem as long as he gives me transport*
143. **I:** alright, thank you very much... Do you see that he has passion to know more about cervical cancer screening?
144. **R*:*** *Yes*
145. **I:** Why are you saying that?
146. **R**: W*hat he says is what makes me say that*
147. **I:** What does he say?
148. **R**: *He says that the health workers have done well to come to our community because they have revealed the hidden disease if we had waited till when i started showing signs of the disease it would have been impossible to get healed.*
149. **I:** Alright. Thank you. Why did you think it was important to discuss it with him?
150. **R**: *I wanted him to know my status*
151. **I:** After knowing what should he do?
152. **R:** *I wanted us to be doing things together like a family*
153. **I:** Alright thank you very much. after the thermo heating on the infected area you were advised not to have sex for a month for the place to heal. was this a hard thing for you?
154. **R***: No. he understood*
155. **I**: he agreed... understood?
156. **R:** *There was no problem*
157. **I**: And he allowed you to stay for a month without sex?
158. **R:** *Mmm*
159. I: How did you feel about this?
160. *\***R**: *I felt good because I knew that if they have given us that number of days they have seen something we better follow it.*
161. **I:** Do you think men should strongly take part in cervical cancer screening campaigns?
162. **R:** Y*es they should be encouraging their wives*
163. **I:** How can they take part?
164. **R:** *They should be encouraging their wives to go for cervical screening to know their status*
165. **I;** Any other way?
166. **R**: T*here isn’t…*
167. **I:** How can the hospital get involved in encouraging men to take part?
168. **R**: Y*ou can encourage them in the way you want*
169. **I**: we want your ideas how should we go about it?
170. **R:**Y*you are the one to encourage them have counselling sessions with men together with us if they are understanding enough they should be able to force their wives to go for screening.*
171. **I:** Alright, ok if we come to your village how can we teach men about cervical cancer
172. **R:** *You know how to do it*
173. **I**: I want to get your views with regard to the knowledge you have about men from your village, how can we explain to them?
174. **R:** Y*ou can explain to them for most of them have women in their homes so they should be able to understand the dangers involved. no man would want his wife to die and leave him with kids*
175. **I:** Is there anything new you have learnt about cervical cancer/about cervical cancer screening which you dint know before the survey started?
176. **R**: *Nothing*
177. **I**: You knew everything?
178. **R**: *No I didn’t know anything*
179. **I:** What things did you know?
180. **R**: *Knowing my status, how I can protect myself and how I can do*
181. **I**: In the way you can do what?
182. **R**: *Like the way we have come here, for if I didn’t get screened I could not have known my status*
183. **I**: You talked about protecting how can you protect yourself?
184. **R**: *I am talking about the treatment which we have been given here by health workers*
185. **I**: ... [Silence]… so who do you think should get screened for cervical cancer?
186. **R***: Woman*
187. **I;** Woman?
188. **R**: *mmm*
189. **I**: What type of a woman?
190. **R**: *Aged from 25 and above*
191. **I:** What makes you think so?
192. **R**: *They are the ones in child bearing age and have matured body*
193. **I:** What do you mean by mature body?
194. **R**: *When we got to the doctor to screen us they said the eligible age is 25 and above*
195. **I**: Alright, these eligible women of 25 year of age and above, how often should they test for cancer?
196. R: *Every three years which means after testing this year they will have to do it again in the fourth year*
197. **I**: Why do you think so?
198. **R**: That’s what they told us
199. **I:** As for in the future how should it be?
200. **R**: A *year should be ok because one cannot know the time a disease would start and one cannot prepare for a disease*
201. **I**: Alright. I would like you to tell me your opinion about what women from your community think about cervical cancer screening?
202. **R**: *I can’t know*
203. **I:** You can’t know... but I heard you saying some women from your area didn’t want to come for testing... [R: oh yeah]...what do you think was the reason?
204. **R:** *I don’t know what they were think for it was an opportunity for a car to come to our area to test us I thought every woman was supposed to come for testing. since we think differently some we went and some didn’t.*
205. **I**: What other thing do you think do they hold?
206. **R**: *I can’t know*
207. **I**: How do you think di the message about the issue got to them?
208. **R**: *Mmm, it is difficult*
209. **I**: Do you think they know better about cervical cancer?
210. **R**: *No they don’t, had it been they knew they would have come for testing. they don’t know about the advantages and it disadvantages*
211. **I**: Why do you think they don’t know?
212. **R**: *They don’t know because had it been that they knew they would have taken a step testing. but you can see they don’t have that kind of consciousness*
213. **I**: Alright. in your area are there any reports of discrimination?
214. **R**: *There is no being discriminated against*
215. **I**: What about think of being at a risk of catching cervical cancer among women in your area? how is it?
216. **R**: *They don’t talk about it*
217. **I:** Do you think women in your area understands the importance of screening for cervical cancer?
218. **R:** *To some it is important to others not*
219. **I**: Why to some is it important?
220. **R**: *They want to know their status whilst those who don’t care about their life*
221. **I:** Do you think that women are interested in screening for cervical cancer and it treatment?
222. **R**: *Some are interested some not. those that are interested go for testing those who dont they think it isn’t important to them*
223. **I**: Alright, so what do you think can make a person not to want to get screened for cervical cancer?
224. **R**: *Ignorance*
225. **I**: Ignorance? [I: mmm]... what else?
226. **R**: *Low self-esteem... [I: how] ... others they are afraid of their reactions after they got the results they think if they are found with it they may commit suicide. as such they choose not to go*
227. **I:** What else can hinder...
228. **R**: *Nothing more*
229. **I:** What problems can women face when getting treatment for cervical cancer after screening?
230. **R**: *Don’t know*
231. **I:** Let’s say the woman is married wants to move from home and go to screen for cancer, what challenges can she face?
232. **R:** ... *Telling her husband what she wants to do... I want to the hospital to get screened for cancer and I would like you to come with me. if he refuses and just ask for money for transport*
233. **I:** So what are the challenges can she face when explaining it to her husband?
234. **R**: *I don’t know*
235. **I**: Or I should say what problems or hindrances can she face that can make her fail to go to the hospital?
236. **R:** There are so many problems that can hinder a person but as for this case I am not sure what can limit her
237. **I**: So as in this case what could be the husband's contributing factor for the woman to fail to go to the hospital?
238. **R:** *Not giving his wife transport can make the woman fail*
239. **I:** What else?
240. **R**: *There are some men who forbids their wives to go to the hospital for their own known reasons*
241. **I**: What about partners, how can they influence woman's failure?
242. **R**: *I can’t know*
243. **I**: Alright. [I clear throat]... in your opinion how do you think should the treatment for cervical cancer be rolled-out to ensure that a lot of women get screened for cancer? ... how should the treatment be delivered?
244. **R**: *As for us you came to us in our village and those that wanted to get screened for cancer they did and those who didn’t want it was their right as well. so I feel it is difficult if we start talking about those who didn’t get screened in getting treatment; how are they going to be helped?*
245. **I**: Ok, in your opinion how can we help them?
246. **R**: *It’s difficult for when the help came in the first instance they shunned it so how are they going to be helped? it is difficult*
247. **I**: How can you encourage them to go for screening?
248. **R:** *We can tell them to go for screening so that they can know about their status which is the same case with us*
249. **I:** Alright... currently I would like us to talk about a new method of self-testing for cancer which involves taking cotton and use it to extract vaginal fluid and deliver to the hospital. to test at their own time of convenience. however, it is different from the method which you use, this one a woman will not get her results instantly but after some hours or following day. what do you think about this new method?
250. **R**: *I think the same one I used when I heard the hospital has visited I didn’t hesitate but to take part*.
251. **I**: I mean this new method which involves extracting vaginal fluid using cotton
252. **R**: *It is good for those who were shy that and concerned that people will see their nakedness. so such people… they will think it is important to do the testing by themselves and deliver the cotton to the hospital*
253. **I:** Alright... would you be interested to use this method?
254. **R**: *Yes, I can be interested if I was not tested already*
255. **I:** Why are you saying so?
256. **R**: *So that I should know about my status*
257. **I:** Oh ok, what are other advantages of this method apart from the ones you have mention already?
258. **R**: *As for me there isn’t any other advantage besides that*
259. **I:** What about the disadvantages of this method
260. **R:** *There isn’t s well*
261. **I:** Do you think this should be happening at home?
262. **R**: *No at the hospital*
263. **I:** How reliable is it?
264. **R:** *This is reliable is you can be smart because these things are important and help us women*
265. **I**: Alright, how would you compare this method of self-testing and the one that was done on you by a doctor?
266. **R:** *These are two different methods with the self-test you would just get the fluid and take it to the hospital while the other method the doctors will scan you in your stomach and spray some medicine. so you see the difference this one you are given medication whilst the self-test its just extracting vaginal fluid without protection*
267. **I:** Among these two which one do you prefer most
268. **R***: Spraying us some medicine*
269. **I**: Why?
270. **R***: To ensure that the germs that have been found should die*
271. **I**: What can other women in your community think about the conduct of using cotton to extract vaginal fluid for testing?
272. **R***: It is difficult*
273. **I**: What if you tell them about this method that... what can other women do?
274. **R: I***t is difficult about what they can say we think differently. what is good to me cannot the same be good to another*
275. **I:** Ok. Do you think that a lot of women can choose to use this method?
276. **R:** *Yes*
277. **I**: Why do you think so?
278. **R**: *Because if they find you with the disease they will right away put you on treatment*
279. **I**: Especially the self-testing method
280. **R:** T*this is very difficult to answer because what other person thinks is not the same with what you think*
281. **I**: What dangers or risks can women face as they do self-testing/
282. **R**: *Mmm, it is difficult (with lower voice)*
283. **I:** Are you tired?
284. **R**: *No am not... [both busted out laughing: hahhah]*
285. **I**: Here we talking about a woman taking the sample by herself. what do you think are the likely challenges can she face?
286. **R**: *It is difficult in the way once you deliver the sample you are gone unlike when the doctors perform it by themselves because they will give you treatment. waiting for the results is another time one has to bear*
287. **I:** But it won’t be taking much time
288. **R**: *Mmm, but still that’s time. treatment you get right after screening is different from one you will get a day after*
289. **I:** What other reason can make women reluctant to perform self-testing?
290. **R**: *As for me I can’t because I have seen the importance of being screed by a doctor*
291. **I**: What about other women?
292. **R**: *I can’t tell about what they think*
293. **I**: What makes you think women would prefer to be screened by a doctor?
294. **R**:T*hose who want can go... without forcing anyone*
295. **I**; Why
296. **R**: *Everyone watches over her own life... or [local language ideom: "phukusi la moyo sakusungila ni amzako"] would you force your friend to go to the hospital when she doesn’t want/ no, it is difficult*
297. **I**: So why do you think she might want to opt for hospital screening?
298. **R**: *She should to know her status just like how we did*
299. **I**: I want to get your comments about cervical cancer screening work in Malawi. in your opinion, should the MOH add the self-testing as one of the methods of cervical cancer screening methods?
300. **R**:M*mmm (seems not to be sure)*
301. **I:** We want to know your ideas that apart from the testing you went through, should there be another one whereby women can perform the test on their own?
302. **R***: They should be screened by the doctor*
303. **I:** Why do you think so?
304. ***R***: *As for us we were tested by the doctor and treatment wasn’t a problem*
305. **I**: Alright, do you think this can make women to easily get screened for cervical cancer?
306. **R**: *It’s not difficult*
307. **I**: You mean the self-testing?
308. **R**: *No that’s nonstarter, doctors should do it you*
309. **I**: Alright… thank you very much... so you are saying that it is not good for women to do the self-testing
310. **R***: Mmm*
311. **I**: So may which categories of women do you think it wouldn’t be appropriate for the self-testing?
312. **R***: Each and every woman is not supposed to do the self-testing. everyone should or must see the doctor*
313. **I:** Thank you very much that marks the end of the questions I had unless you have a question. perhaps you have something to raise which you didn’t during the course of the interview
314. **R:** *As for me after they sprayed the medicine inside me after some days I found out that I starting releasing water and then puss. when that stopped I felt relieved and my body went back to normal*
315. **I**: Is there anything more you would like to add
316. **R***: I come from a long distance it will take me time to get home after I leave*
317. **I:** So what are your thoughts?
318. **R**: *If they should have been providing a vehicle just like the way mr (Name withheld) came to pick... but the amount of money they give is not enough... we get into debts that we were not supposed to*
319. **I**: Anything more?
320. **R**: *No*
321. **I**: Madam I would like to thank you for your time... this is the end of our discussion... thank you very much!
322. **R***: Thank you*
