# Supplementary material for: Perceptions of cervical cancer and motivation for screening among women in Rural Lilongwe, Malawi: A qualitative study
Source: PLoS One. 2022 Feb 7;17(2):e0262590. doi: 10.1371/journal.pone.0262590 (PMC8820632; doi:10.1371/journal.pone.0262590)
Supplement: S3 File — (ZIP) [file pone.0262590.s003.zip › VIA_293.docx]

**PID: VIA 293**

**INTERVIEWER ID: 748**

**DATE OF INTERVIEW: 24 October 2017**

**TYPE OF INTERVIEW: 12 weeks follow up**

**KEY: I= Interviewer, R= Respondent**

1. I: Thank you for meeting with me today. I really appreciate for your time; whatever you are going to say shall be very important. I am working with a team of data collectors for University of North Carolina Malawi. They are the ones who conduct different kinds of research but from North Carolina-America but they are operating from here as you can see the building. Your ideas are very important to us, it will help us understand the importance of cervical cancer screening campaigns here in Malawi. As such there is no right or wrong answer. Don’t be prompted to think that you are going give us wrong answers-whatever you feel like saying: "say it". Please note that what you are going to tell us is private and confidential and shall be used only for the purposes of this study. I shall record the discussion to help us properly write the proceedings for the discussion. However, no any identification information about you shall be linked to what you are going to say.
2. R: OKay
3. First of all I would like to know about cervical cancer screening. Can you tell me your understanding about cervical cancer screening and the treatment which you received?
4. *R: Yes, after they screened me they told me they have found me with cancer cells*
5. I: Did they say cancer cells?
6. *R: Yes, so they burned...*
7. I: Please raise your voice… Alright, what method did they use to find you with things that cause cancer?
8. *R: You mean the metals?*
9. I: ... Mmh.... what method did they use... just tell me what happened.
10. *R: That time I took off my clothes and lied and they inserted a metal in my vagina and they told me that they have found me cancer cells on my cervix*
11. I: mmh, what was their aim to screen you using "that" metal?
12. *R: They wanted to find out if I have cancer cells. so they wanted se if the germs are there or not*
13. I: I want to understand there... you have said things about germs. uh, how were they explaining about these germs?
14. *R: How they looked?*
15. I: Mmh, like what they could see. what were they explaining to you as to what they saw?
16. *R: They just told me that I have been found with the germs and that they will burn it. that is what they told me*
17. I: You have explained that they found you with cancer cells and that they will burn the cells... what else happened at that time?
18. *R: In my body?*
19. I: Yes or what was happening at that "place"?
20. *R: What was happening was first to take the metal and insert it, then they said we have burned with electric fire from battery.*
21. I: So after you arrived you went straight and started inserting you metals?
22. *R: After we arrived we went through counselling sessions and explained to us about the whole process. They said first, we are going to test urine to whether you are pregnant or not, so if they find us negative then they would proceed to screen us for cancer.*
23. I: So what was this counselling about-what did they tell you?
24. *R: They were telling us that we want to screen you for cancer and this is transmitted through sexual intercourse, or because of a man's uncleanness. that was what they told us.*
25. I: What else?
26. *R: About the counselling?*
27. I: Mmmh
28. *R: One more thing they said was that it was private and confidential. After everything everyone will walk out normally as she does no impaired movement.*
29. I: Besides, that was there anything else?
30. *R: They also tested us for HIV*
31. I: So what was your result?
32. *R: The HIV?*
33. I: Mmm
34. *R: It was ok*
35. I: Now I would like to know your ideas about campaigning. you know what campaigning is?... Campaign about cervical cancer screening like the one which you received. Why did you decide to get screened or to participate in this study?
36. *R: I did that because I wanted to know the condition of my body. you can just be staying and never be certain that you are ok or not. so this time I thought it wise to go and get screened*
37. I: What made you to go and find out about your condition?
38. *R: When I heard the chief announcing the news... then I said aaah… no its wise that I go*
39. I: What did he announce about?
40. *R: He announced that village (name of village) has been visited by a mobile clinic which is screening women for cervical cancer so women are requested. that is why I decided to go, for it was an advantage to be visited by doctors in our homes.*
41. I: Was there anything which you worried about before the testing?
42. *R: There wasn’t*
43. I: What else did you hear about apart from what the chief said?
44. *R: About cancer?*
45. I: Mmh
46. *R: We used to hear that cancer is dangerous it has no cure but then I couldn’t take it seriously... so when they announced I decided to go*
47. I: What were your fears before testing?
48. *R: I worried that if I will be found with the disease I shall be in trouble and even my children as well. for it takes some time while one you are still struggling with the disease before you die... sure that was my fear*
49. I: Is there any other fear?
50. *R: No*
51. I: Were there any misconceptions that used to spread about cancer screening?
52. *R: People used to talk a lot. They were saying that the equipment which they are using are painful, and people should go. but as for me I gathered courage and told myself that I will go and see for myself*
53. I: Alright, thank you so much. How did you feel after you got your results and find out that they were not good at all?
54. *R: I thought that I did well now that I know my problem and if I get sick I will know where it is coming from... now that I have received treatment I am very happy*
55. I: I want to know about how you felt right at the time they told you about your status. how did you feel when they told you, you had cancer cells?
56. *R: I was comfortable because before everything they told us that whosoever they are going to find with the cancer cells they will burn them right away*
57. I: So you didn’t have any fear?
58. *R: No*
59. I: Did you understand what it meant?
60. *R: Yes, when they explained to us*
61. I: Mmh, so you understood what it meant by being found with cervical cancer cells?
62. *R: The understanding was that it was the beginning of getting sick in the body*
63. I: What do you by beginning of sickness in your body?
64. *R: It is like disease which you didn’t know before, and now have been discovered the time you are sick*
65. I: so at the time they were screening you what can you say that it went on well?
66. *R: The good thing was the message I received that, "I have been found with the cancers cells and they will burn". that was the good thing*
67. I: As for you what are your comments about the place?
68. *R: Place?*
69. I: Place where they tested you... how was the place?
70. *R: It was ok*
71. I: What do you mean by ok?
72. *R: It was private and that no one else would see or hear anything*
73. I: What about the time it took to test you?
74. *R: You mean duration?*
75. I: Mmh
76. *R: It took longer, for some when they went inside they didn’t take much time, but as for me it took a lot of time. so I said that I have taken time because they have find me with the cancer, that is what I thought.*
77. I: So after you saw that it was taking longer how did you feel?
78. *R: Couldn’t do anything since I was looking for help*
79. I: What are your comments about the treatment you received?
80. *R: For me I saw it was ok. After the treatment I started passing out water... for I arrived late for the counselling... so I didn’t know anything about passing out. so after I saw that water, water, water and a day passed. Later I decided to go and ask by the health workers had moved to a different location (name of location). so I went to my friend who was also diagnosed with cancer so she said that they made an announcement that we will be releasing water... so that is not a problem" sure*
81. I: Alright. so like the way they explained to you, how do you look at it?
82. *R: Explaining...*
83. I: The time they were teaching you about cervical cancer, how was the teaching?
84. *R: They taught us with love so that we can know everything*
85. I: What made you think that they wanted you to know about everything? how were they doing it
86. *R: The way they talked. they talked with love so that one can understand what they said. that is how I knew that they taught with love for a harsh person cannot be hidden likewise one with love*
87. I: What do you think would have been made better as compared to that time?... May be there is something which you are saying that if it happened this way, things would have been better...
88. *R: As for me I can’t say that if it happened in this way because it was my first time but I just understood because what they were telling me I perceived that it was good*
89. I: Why?
90. *R: I did that because I wanted to know the condition of my body. you can just be staying and never be certain that you are ok or not. so this time I thought it wise to go and get screened*
91. I: What made you to go and find out about your condition?
92. *R: When I heard the chief announcing the news... then I said aaah, no its wise that I go*
93. I: What did he announce about?
94. *R: He announced that village (name of village) has been visited by a mobile clinic which is screening women for cervical cancer so women are requested that is why I decided to go, for it was an advantage to be visited by doctors in our homes.*
95. I: Was there anything which you worried about before the testing?
96. *R: There wasn’t*
97. I: What else did you hear about apart from what the chief said?
98. *R: About cancer?*
99. I: mmh
100. *R: We used to hear that cancer is dangerous it has no cure but then I couldn’t take it seriously... so when they announced I decided to go*
101. I: What were your fears before testing?
102. *R: I worried that if I be found with the disease I shall be in trouble and even my children as well. for it takes some time while one you are still struggling with the disease before you die... sure that was my fear*
103. I: Is there any other fear?
104. *R: No*
105. I: Were there any misconceptions that used to spread about cancer screening?
106. *R: People used to talk a lot. They were saying that the equipment which they use are painful, and people should go. But as for me I gathered courage and told myself that I will go and see for myself*
107. I: Alright, thank you so much. How did you feel after you got your results and find out that they were not good at all?
108. *R: I thought that I did well now that I know my problem and if I get sick I will know where it is coming from... now that I have received treatment I am very happy*
109. I: I want to know about how you felt right at the time they told you about your status. how did you feel when they told you, you had cancer cells?
110. *R: I was comfortable because before everything they told us that whosoever they are going to find with the cancer cells they will burn them right away*
111. I: So you didn’t have any fear?
112. *R: No*
113. I: Did you understand what it meant?
114. *R: Yes, when they explained to us*
115. I: Mmh, so you understood what it meant by being found with cervical cancer cells?
116. *R: The understanding was that it was the beginning of getting sick in the body*
117. I: what do you by beginning of sickness in your body?
118. *R: It is like disease which you didn’t know before, and now have been discovered the time you are sick*
119. I: So at the time they were screening you what can you say that it went on well?
120. *R: The good thing was the message I received that, "I have been found with the cancers cells and they will burn". that was the good thing*
121. I: As for you what are your comments about the place?
122. *R: Place?*
123. I: Place where they tested you... how was the place?
124. *R: It was ok*
125. I: What do you mean by ok?
126. *R: It was private and that no one else would see or hear anything*
127. I: what about the time it took to test you?
128. *R: You mean duration?*
129. I: Mmh
130. *R: It took longer, for some when they went inside they didn’t take much time, but as for me it took a lot of time. So I said that I have taken time because they have find me with the cancer, that is what I thought.*
131. I: So, after you saw that it was taking longer, how did you feel?
132. *R: Couldn’t do anything since I was looking for help*
133. I: What are your comments about the treatment you received?
134. *R: For me I saw it was ok. After the treatment I started passing out water... for I arrived late for the counselling... so I didn’t know anything about passing out. So after I saw that water, water, water and a day passed. later I decided to go and ask by the health workers had moved to a different location (name of village). so I went to my friend who was also diagnosed with cancer and she said that they made an announcement that we will be releasing water... so that is not a problem" sure*
135. I: Alright. So like the way they explained to you, how do you look at it?
136. *R: Explaining...*
137. I: The time they were teaching you about cervical cancer, how was the teaching?
138. *R: they taught us with love so that we can know everything*
139. I: what made you think that they wanted you to know about everything? how were they doing it
140. *R: The way they talked. They talked with love so that one can understand what they said. that is how I knew that they taught with love for a harsh person cannot be hidden likewise one with love*
141. I: What do you think would have been made better as compared to that time?... may be there is something which you are saying that if it happened this way, things would have been better...
142. *R: As for me I can’t say that if it happened in this way because it was my first time but I just understood because what they were telling I perceived that it was good*
143. I: Alright... at the time you were getting screened, how do you look at the need for men take part? How important is it?
144. *R: It is important that they take part to enhance their understanding especially when it comes to issue of spending some weeks without sex. Some they do understand and some they don’t. so they have to take part so that they can hear it by themselves from the doctors*
145. I: What... did you find to be simple during your screening?
146. *R: The time I was being tested?*
147. I: Mmm
148. *R: It was easy for me on the aspect that it was free of charge. you could come anyhow you wanted and after they testing you off you go without paying anything*
149. I: What else?
150. *R: "..."*
151. I: There isn’t more? Alright. w\Which part did you find difficult or hard?
152. *R: When they tested me and find me with...*
153. I: Not necessarily. Anything concerning the process which you feel it was hard
154. *R: As for me there wasn’t any hard part*
155. I: Was there anything that happened which you didn’t expect?
156. *R: Yes. I didn’t expect that I will be found with the disease*
157. I: Alright. There are problems that people face after screening and to come back for check-ups. it can be difficult for people to come back. Did you have any challenge when you had to come back for this check-up visit?
158. *R: Problems were there but since we asked them the time they came about possible limits like transport. so in the discussions they told us that we can borrow and they are going to reimburse. so I considered it to be an easy thing to borrow money from someone and after I return from my trip I pay them back*
159. I: Alright thank you. Which problems do you think... you have said that you because you borrowed there wasn’t any problem but which problems do you think other women can face and make them fail to come back for check-up visits?
160. *R: Problems might be there considering the way we live in the village. There is much trust when it comes to issue of borrowing each other money. so some women might be refused and as such making them to fail to come*
161. I: Apart from money problems, what other problems?
162. *R: The second trips?*
163. I: Problems that can make a person fail to come to the hospital
164. *R: It’s because of funeral or sickness*
165. I: Is there any other?
166. *R: Those are the only problems that can make you fail to leave your home, imagine you are sick or your child is sick... but they said that if it was a funeral you ask to be excuse but if it is your child who is sick then that is a problem. That’s what they told us*
167. I: What about spouse's influence?
168. *R: Like your husband denying you?*
169. I: Mmmh
170. *R: No... but if the man is understanding cant deny you*
171. I: Alrigt thank you so much. So how do you think can we help to deal with these problems?
172. *R: Like on the issue...*
173. I: The problems which you have just talked about like sickness, money....
174. R: *As for problems like sickness and funeral that one you can’t put a check on, because they are uncertainties. But as for money, just that women need to be trustworthy in order to be taken serious by friends whenever you ask for anything. if you are trustworthy, people give you money with no problems*
175. I: Is there any other way we can deal with these problems?
176. *R: I think those are the only ways*
177. I: Now I would like to know about the support you can get from the community or spouse. Did you discuss with anyone about your screening?
178. *R: I discussed...*
179. I: Whom did you discuss with?
180. *R: First I would say I discussed with my relatives and my husband*
181. I: what did your relatives said after you explained to them?
182. *R: They commended me that I have done a good thing... because it is something that we didn’t expect... it is well that now you have find treatment*
183. I: Did they have any questions?
184. *R: No questions*
185. I: What did your relatives say when you told them?
186. *R: When I told my relatives they said, "it has been nice... because when things start to backfire in future we wouldn’t have known the origin"*
187. I: Did they have questions?
188. *R: No they didn’t they just encouraged*
189. I: Since you told your husband that they found you with cervical cancer cells... what exactly does he think about this?
190. *R: I can’t really tell for I don’t know what he thinks*
191. I: When you are coming here do you need his permission?
192. *R: Yes and we come together*
193. I: Ok. what any other support do you get from your husband apart from escorting you?
194. *R: Concerning this disease?*
195. I: no any support he gives you for you to come here?
196. *R: transport*
197. I: Alright. When you were explaining this to him, do you think he was interested to know more?
198. *R: Yes*
199. I: How do you know that he is interested-what does he do exactly?
200. *R: I saw it through his actions that he is able to escort and he is always zealous to be present even when you are asking me questions like this*
201. I: Alright thank you. So... you have explained that you discussed with your husband... so why did you tell your husband about your results?
202. *R: Because when I was going for the testing I told him and expressed about the possible outcomes that whether they will find me with the disease or not. so after I came back I said to him: "I am back from the trim, the results were not good but now it is fine. so they have asked us to spend six weeks without sex"*
203. I: Alright, thank you. After screening and the thermo heating of the infected area you were advised to spend a month without sex was this a difficult task to you?
204. R: It wasn’t difficult?
205. I: Does your husband help or agree with this?
206. *R: Yes he does however, the duration is long for a man who has one sexual partner so it becomes difficult*
207. I: So what was he doing?
208. *R: Nothing else just keeping promise*
209. I: So what was he saying concerning sex?
210. *R: He was just saying that: "the days are just do much considering a man who has one woman it is difficult". that is what he was saying and i figured out that the days are too much*
211. I: How did you feel?
212. *R: Nothing else as for me my concern was on the treatment I was getting*
213. I: Do you think that men should greatly take part in women's cervical cancer screening?
214. *R: Yes they should for them to know how it "comes"*
215. I: So how do you think should they take part?
216. *R: On that part they can when you invite them to come during teaching sessions so that they see that here and there things are not good. Especially on the part that it is caused by a man's uncleanness that they don’t wash their parts properly… when you try to talk to them they take it for granted but if they hear from a different they would take it seriously.*
217. I: Alright. Apart from teaching them what other way can we use to encourage men that that they should be taking part?
218. *R: They should be taking part on following the instruction about refraining from sex for a month*
219. I: Is there anything else?
220. *R: They should also know that they are required to give us the transport*
221. I: So how can we encourage them?
222. *R: As for them, men from the village… you need to go through the chief so that he summons them; and when they come explain to them everything, unlike calling individual by individual*
223. I: Mmh, alright. So you have explained that we have to teach men about cervical cancer. so how can we teach them?
224. *R: So it should be you who should find the way how to best approach men*
225. I: In your opinion what could be one of the ways?
226. *R: I don’t know*
227. I: Is there anything new you have learned about cervical cancer of screening which you didn’t know before the study?
228. *R: Yes there is.*
229. I: Like what?
230. *R: I didn’t know how cancer is tested. to me it was strange to learn that they also insert metals to test how a person is*
231. I: What else?
232. *R: Another new thing is about how this disease is transmitted I didn’t know it was through sexual intercourse*
233. I: What else didn’t you know apart from the use of metals?
234. *R: I dint know that cancer disease was contagious*
235. I: Who do you think should get screened for cervical cancer?
236. *R: Women*
237. I: What type of a woman?
238. *R: Woman who has given birth before.*
239. I: Is there any other type of woman?... or i should ask why should it be a woman who gave birth before?
240. *R: Because she haves sex with a man*
241. I: Another type of woman?
242. *R: An old woman*
243. I: How old?
244. *R: Starting from 50 years old and above*
245. I: Why 5o years?
246. *R: Because she has sex*
247. I: So how often should these women get screened?
248. *R: When they get screened for the first time as for the rest they will know from their doctor*
249. I: No, we want this type of work to be done according to your preference-like how you want women to be screened. so it will be up to the researchers to respond to the choices of women
250. *R: Any type of women or old women*
251. I: Those who can get screened like the examples you have given...
252. *R: As for me I think that after screening those that have been found with the cancer should come for confirmatory check on the status of the cancer cells. so as they wait, at least a year should pass*
253. I: Why one year?
254. *R: I saw that three years is too much*
255. I: why?
256. *R: I saw that the days are too many… it can happen that within those three years the cancer cells might resurface and thus making you think the first treatment didn’t go well... but as for a year it is 12 months so you can be seeing your results with no problems*
257. I: Now I would like you to give your comments about future screening. in your opinion, what do women from your community think about cervical cancer screening?
258. *R: Women think that... those that didn’t test?*
259. I: all women from your community
260. *R: They are afraid of getting tested for getting a result which they least expected. as for other are determined to get tested for them to know their status.*
261. I: What else do they think about screening?
262. *R: Another thing they think is that if they find them with the cancer cells their marriage is going to collapse in the course of six weeks without sex*
263. I: Anything else
264. *R: No*
265. I: Do you think that women from your area understand the importance fo getting screened for cervical cancer?
266. *R: Yes they do understand*
267. I: What makes you think so?
268. *R: Because they are certain that they will know the status of their life*
269. I: What else?
270. *R:Tthe only important thing i know they understand is knowing their status*
271. I: In your opinion do you think that women are interested to screen for cervical cancer and receive its treatment?
272. *R: Very much*
273. I: What makes you think that way?
274. *R: I saw the time we went for screening there was a multitude and even after us i also heard that larger group of women went for screening*
275. I; Is there anything you would like to add... what do you think could be the reason making a person not to go for screening?
276. *R: It’s just because of fear. We people are born differently and it’s because of fear*
277. I: What can they be afraid of?
278. *R:Because of how other people explain about the issue... like insertion of the metal etc. so they say if i go and find me with the cancer cells how will it become of me... so it is because of fears.*
279. I: Is there anything else that can make them unable to go for screening?
280. *R: I am sure that it is only because of fear*
281. I: What problems do you think that women can have as they go for screening?
282. *R: Aaah... there is no problem*
283. I: Imagine a woman need to leave from home... So what could be the problems she can face?
284. *R: When I was leaving from home..., maybe he husband can forbid her to go. That one as well is problem because you really wanted to go but someone is refusing you*
285. I: Is there any other problem
286. *R: Yes when a woman is having "her monthly period" she can’t go. Because that time she is also willing but because of her condition she can’t go*
287. I: Alright what other problems?
288. *R: When a woman is pregnant she can’t go for screening because what she wanted didn’t happen*
289. I: Another problem?
290. *R: That’s all*
291. I: In your opinion, how should cervical cancer screening or treatment be offered to ensure that a lot of women get screened?
292. *R: As for me the campaign they held in our village like coming and encouraging us to go for screening and assuring us of transport is sufficient support... they should continue since some villages are located very far and thus making it hard for a woman to come*
293. I: Alright what other method can be used to ensure that a lot of women get screened?
294. *R: The same methods I have talked about*
295. I: Alright. How can you encourage women for they to get screened?
296. *R: I can tell women that: "women it is good to go to the hospital to get tested so that you can know how your body is, these days good health depends on testing it is really important that you should be going there". that is how I can tell them*
297. I: Now I would like us to talk about taking testing samples by yourself. Now let’s talk about taking samples of testing by yourself from the vagina using cotton... cervical cancer testing. A new method of testing cancer has been discovered which is involving a woman taking cotton and extract vaginal fluid from her vagina and deliver it to a health centre of hospital like this one so that they can test it at her time of convenience. However, unlike the method that was used on you, this one women will not be getting their results instantly as have to wait for some hours or come a day after. what do you think about these ideas?
298. *R: It is very good method for I can simply get a cotton and take the samples to the hospital without waiting for the health workers to come to the village*
299. I: Alright. What makes you think it is a good method?
300. *R: For you to wait for them to come it is hassle, while this you will do at your own time and bring the sample to the hospital... without waiting for such a long time*
301. I: Would you be interested to get screened using this method?
302. *R: Very much*
303. I: Mmh.. what other advantages do you see in this method?
304. *R: The advantage is that you are the one in control, for you think how to do it and when to do it*
305. I: What could be the disadvantage?
306. *R: Since we have to wait for some time even up to a day, it means even if you come from a longer distance you still have to come for the following day. that is its disadvantage*
307. I; Is there any other disadvantage? Should it happen from home?
308. *R: Like taking the cotton...?*
309. I: Mmh
310. *R: You can see by yourself how you do it when you take it from home or when you arrive at the facility*
311. I: Do you think that it is a reliable method?
312. *R: It is very reliable*
313. I: Why do you think so?
314. *R: As I said that some people are afraid with metals... and others do not want to show others their genital parts. so it is a good method to such people*
315. I: How would you compare this method of self-testing and the one which was used on you?
316. *R: I could compare these method as... to someone who is not understand can think that they are different methods that the one they use cotton is bogus method. as to someone who is understanding can think that it is real*
317. I: As for you what method would you prefer?
318. *R: The one they screen you*
319. I: Why?
320. *R: You get your results sometime*
321. I: Alright. In your opinion what can women from your community think about this method of taking vaginal fluid by themselves using cotton?
322. *R: Some can choose this method of self-testing but others would choose the other in order to get the results same time*
323. I: Do you think that women can choose to get screened using this method?
324. *R: Yes*
325. I: Why
326. *R: Because of fears and being shy*
327. I: What challenges can women face as they take testing samples by themselves?
328. *R: Problems could be there because they cannot be certain if the vaginal fluid is enough or not*
329. I: Alright. what are your fears on this issue?
330. *R: The one taking sample by yourself?*
331. I: Yes
332. *R: My fears are that a person need to be taught about the measure of the fluid she needs to extract*
333. I: What can hinder women from taking samples by themselves?
334. *R: What can make them fail?*
335. I: Mmh
336. *R: There are some women who do not release much fluids while others they release much. so that could be a challenge to a woman who doesn’t release much vaginal fluid or does but after a long time... [I: mmm]*
337. I: Alright. in your opinion what could be the reasons that can make women unwilling to take fluid samples by themselves?
338. *R: Reasons?... [I: mmm]... aah, it is just because of fear*
339. I: fear of what?
340. *R:Aaah, fear of getting the wrong measure of vaginal fluid sample. They can’t be sure if they have collected so much or so little*
341. I: What can they think will happen?
342. *R:They might think that the doctor would tell them that, "you have collected too little fluid or may be say you have brought too much... it was supposed to be like this so and so"*
343. I: Alright what makes you think women would love to go to the hospital to be screened by a doctor unlike by herself?
344. *R: The reason is that...*
345. I: Why would they love to be screened by health workers?
346. *R: Because they want to know their status that is her cervix ok or not*
347. I: On the choice of place where to get tested, why would they opt for the hospital?
348. *R: Testing using cotton?*
349. I: Yeah, why would they want to be helped by a doctor?
350. *R: ... Not taking it from home but to the hospital?*
351. I: Mmm
352. *R: She might think that it is going to dry whilst it is required to be moist*
353. I: Now let’s talk about how you can comment on the future of cervical cancer screening in Malawi. In your opinion should the MoH include this method of self-testing to be part of methods for cervical cancer testing in Malawi?
354. *R: As for me I think that the cotton method should not be included because the way I felt during the time they helped it was really nice. Women should just forget everything for most of the things like giving birth it is done at the hospital where they also take their clothes off. So taking clothes off isn’t an issue at all, there is nothing to be afraid of*
355. I: Iike I said with the method which was used on you, should another method be added to make them two?
356. *R: It is important and that the method should be that one which involves cotton...*
357. I: Why?
358. *R: Because some women are shy*
359. I: Alright. Do you think this can make it easy for women to get screened for cervical cancer?
360. *R: Yes*
361. I: Why do you think so?
362. *R: It will be one's choice whether to go to the hospital or use cotton... women will choose a method which they are most comfortable with*
363. I: Which type of women would be most suitable to take fluid samples for cervical cancer test with cotton?
364. *R: It should be women from ages of 50 and above*
365. I: Why?
366. *R: They are "matured"*
367. I: What about it?
368. *R: When they are such old... you can recall that in the past people did not mostly deliver at the hospital so they think it is strange to take their clothes off before another person. it's all because they couldn’t deliver at hospital, they did it at home where there was perhaps one or two individuals or sometimes even by themselves*
369. I: Alright. Which are the groups that are suitable for this method?
370. *R: Women who are below 50*
371. I: Why?
372. *R: Because these days most of women deliver their babies at the hospital so there is nothing strange or new.*
373. I: So now I have reached the end of the questions I had. may be you have something to add which we might have missed during the discussion... what you would love to add about cervical cancer
374. *R: I just appreciate for the development to test women for cervical cancer. It was something which women did not expect or I can say that I didn’t expect. like the testimony I have is that in the past I used to feel week and felt general pains in my stomach... so when these came and after I got screened, and my body started to change and I said to myself what exactly... or was my body immunity dwindling. it might be true that my immunity was declining, as compared to now am much better. I still feel stomach pains but not as severe or frequent as I used to... sure*
375. I: Thank you very much. Anything more?
376. R: No
377. I: Thank you for your ideas that is the end of our discussion...
