# Supplementary material for: Perceptions of cervical cancer and motivation for screening among women in Rural Lilongwe, Malawi: A qualitative study
Source: PLoS One. 2022 Feb 7;17(2):e0262590. doi: 10.1371/journal.pone.0262590 (PMC8820632; doi:10.1371/journal.pone.0262590)
Supplement: S3 File — (ZIP) [file pone.0262590.s003.zip › VIA_329 Mssed.docx]

**Participant ID: VIA_329**

**Interviewer ID: 466**

**Date of interview: 21 December 2017**

**Type: Missed visit 6 and 12**

**Interview summary:**

This participant reported that she missed her scheduled follow up visit date because she had misunderstandings with her husband. She said that her husband did not really understand why she had to come and he thought that she had prostitution intentions and so he did not allow her to come and since she relies on him to provide transport, she had no other means of getting here. The participant said that after screening, she was told that she had some cancer cells and she had Thermo-coagulation after which she was told to report to the clinic after 6 weeks which she missed. For her to get screened, she said she heard that it was a family planning clinic and so she came with intention of getting a family planning method only to realize that it was a cervical cancer screening clinic. She decided to still get screened as she thought it is better to get the screening done with and worry about other diseases. After she got the Thermo-coagulation treatment, she says she was given condoms and was told not to have sex for 6 weeks which was very hard on her husband. Her husband threw the condoms away and she said his reason was that he did not see why he has to use condoms with his wife, unless she was planning on starting prostitution and after a lot of arguments, they only managed not to have sex for 2 weeks. She explained that the tracer who had been to her house several times had helped because he would at times meet her husband who later on was convinced to let her come to the clinic after carefully reading the consent form as well. She was glad that she was screened and agreed that husbands should play a role in the screening process although she was not sure of what role they would play. She said she would still prefer the VIA screening method as opposed to the self-collecting vaginal swabs and her reason was that VIA is more reliable since it is done by medical personnel, therefore the results from VIA screening can easily be trusted unlike the results from the self-collecting vaginal swab.

**Interview text:**

1. I: Welcome,
2. *R: Thank you.*
3. I: Thank you very much for meeting with me today.
4. *R: Alright,*
5. I: I appreciate your time and know that whatever you share with me today will be very helpful. I am working with a team of researchers from the University of North Carolina in Malawi, where we are right now for instance is part of where this group carries out its work.
6. *R: Alright.*
7. I: Am sorry that you did not make it to your 6-week appointment follow-up. We would like to hear your experience regarding cervical cancer screening with VIA and thermo-coagulation treatment. We would also like to hear about any difficulties you had afterwards or any challenges you had to coming for your follow-up visit.
8. *R: Alright.*
9. I: So your views are very important as they will help us understand how best we can conduct cervical cancer screening campaigns in Malawi.
10. *R: Alright.*
11. I: There are no right or wrong answers here right,
12. *R: [Chuckles] Alright.*
13. I: We will accept anything you share with me and also know that whatever we discuss here will be kept confidential and will only be used for the benefit of the health program and health questionnaire better. So do not worry about hearing anything you say elsewhere alright?
14. *R: Yes.*
15. I: I will record our conversation using these two devices, the aim of recording is so that it can help me remember whatever you say during this conversation, it is impossible to recall everything by heart not so?
16. *R: Yes.*
17. I: But, your name or any identifiable information will not be connected to anything you say in this room.
18. *R: Alright.*
19. I: Have you understood?
20. *R: Yes, I have.*
21. I: Is there any worry or concerns?
22. *R: No, there isn’t [chuckles].*
23. I: Alright, can you tell me your understanding of the cervical cancer screening and the treatment you received?
24. *R: Yes, when I was screened, they told me that the cancer cells have just started and that they did thermo-coagulation. Then they told me to come to the clinic after 6 weeks. But, I did not come because of several reasons from home. Before I went for the screening, there was a car going around which was announcing that those who want to change their family planning method or those who want any permanent family planning method should go. I was taken up by that and we went where they said the family planning is taking place. When we got there we realized that it was cancer screening and not family planning, but we decided to stay and still get screened. They gave us several documents and when I went home, I explained to my husband but we did not seem to agree on what to do. Then, a certain community tracer had been coming to my home, he has been there almost 3 times now. This time around however is when he [my husband] decided that we should come to the clinic and see how it goes, that was also after he read the documents they gave us, and that is how we have come here. before today, whenever I ask for transport he used to refuse and say ‘there is something you want to do there and not for the medication’, so it was hard for me to come here.*
25. I: Alright, and today you have come together with your husband?
26. *R: Yes, we are both here.*
27. I: Alright, you said that you were going to get a family planning method only to realize that there was cancer screening and not family planning,
28. *R: Yes, my aim was to remove the Norplant I had and put on a new one, but when I got there I was told that they are screening for cancer.*
29. I: But you still decided to get screened?
30. *R: Yes, I still did [chuckles].*
31. I: What kind of screening did you undergo when you got there?
32. *R: I lied down so I could barely see what they were doing. But I could see them taking their equipment and they were using it in the vagina.*
33. I: Alright.
34. *R: After the screening was done, they told me that I would experience excess vaginal fluids for 2 weeks and indeed that was what happened. They gave me condoms as well so that when I want to have sex with my husband we should use them. But my husband did not understand why he had to use the condoms and so he threw them away. But a week passed before we had sex.*
35. I: When did they advise you to use the condoms, after 1 week?
36. *R: No, they just gave me the condoms so that whenever we want to have sex we should use them. That did not make sense to my husband however and especially since I did not tell him that I am going for cancer screening, he just concluded that if I keep the condoms I will use them to sleep with other men.*
37. I: Okay.
38. *R: After 2 weeks I noticed that the vaginal discharge stopped and since that time, I haven’t experienced any kind of pain. I only feel itchy once in a while, months pass by before I can feel any itch.*
39. I: When were you screened?
40. *R: I think that was in July.*
41. I: Okay, how often do you feel the itching in a month?
42. *R: Maybe once, that is the only thing I feel in my body.*
43. I: How bad is the itching when it happens?
44. *R: Not bad, the vagina just irritates for a little time and then it stops. I don’t even worry about anything, since I started my family planning method I don’t get menstrual periods so that does not worry me.*
45. I: Alright, after screening you said you were told that the cancer cells are there?
46. *R: They said the cells had just started and that is why they did thermos-coagulation and told me to come back after 6 weeks, to which I agreed. But because of the problems I had with my husband, I was unable to come until today.*
47. I: Alright, what do you think the aim of the screening was?
48. *R: I don’t know what their aim was, they said they were taking biopsy which they brought here to the clinic so that they could see if the cancer cells were really there. When they examined it here they found that the cancer cells are not there, but when they screened me they told me that the cells were there. They have just told me to come back after 3 years.*
49. I: Alright, you said you were unable to see what was happening since you were lying down-
50. *R: Yes, I could only see the things they were using and I could feel that they are doing something in my vagina. At times I would move after feeling pain and they were telling me not to move [laughs].*
51. I: [laughs]
52. *R: From there they told me to get up and they said I could go home. After I went home I just noticed the discharge on my pant, and that went on until two weeks, just as they had told me. It happened as they had said, they did not lie about anything.*
53. I: Besides being screened, what else happened on that day when you went for screening?
54. *R: At first I bled a little and it was after the bleeding that the vaginal discharge started to come out.*
55. I: Whilst you were still at the screening place?
56. *R: No, that was after I got home. When I went to the toilet I noticed that there was blood on my underwear. When the bleeding stopped is when I felt the discharge coming out.*
57. I: How much blood was there?
58. *R: Ii, very little blood.*
59. I: Okay, but whilst you were still at the screening place, what else happened?
60. *R: No, nothing else happened, and I did not see anything strange.*
61. I: Alright, thank you. We would like to hear your thoughts about cervical cancer screening campaigns like this one where you got screened right?
62. *R: Yes.*
63. I: Why did you choose to get screened or participate in this study?
64. *R: I chose to take part because I can just be staying at home and yet I have cancer cells developing in me. So, I thought it wise to get screened so that I should know if I have it or not. That is why I went ahead and got screened.*
65. I: Alright, was there anything that you were worried about before the screening happened?
66. *R: I should not lie; I was not worried about anything before the screening. As I go about my day to day life, there is no single day or time when I feel pain or when I have abnormal periods, that does not happen. This is my fourth year without periods, and I cannot say that I feel any kind of pain. Maybe the only pain could be what I have said that sometimes, once a month, I feel a bit itchy in the vagina, that is the kind of pain I can say I feel. I mean would you consider a headache as abnormal? [laughs] So yes, I was not worried about anything, and it’s not like I knew what was happening in the first place.*
67. I: Before they came for the screening, what had you heard about cervical cancer screening?
68. *R: The first time I heard of it is when I left home to go and get my Norplant removed, there at (name of area), that is the same day I got screened.*
69. I: So that was your first time hearing about cervical cancer screening?
70. *R: Yes! That was the first time. there were four of us that day but one of us did not get screened. She went back home because she said she did not tell her husband that she was going to get screened. The three of us are the ones who got screened.*
71. I: So before you got to the place where the screening was happening you had not heard anything about it?
72. *R: No, the ones who were doing community sensitization were saying that a family planning team is coming and so that is what we had in mind when we left our homes. We did not know that it was cervical cancer screening until we got there.*
73. I: Alright, so you had never heard of it and when you left home you had intentions of changing your Norplant, not so?
74. *R: Yes, but when I arrived they told me that they are not removing Norplant, they are screening for cervical cancer.*
75. I: Yes, so all along your plan was to get your Norplant changed, what encouraged you to get screened for cancer when you arrived and realized that they are screening for cervical cancer and not doing any family planning?
76. *R: I was encouraged because I told myself that I should just get screened and then the next day I will still go for my family planning. The next day after I got screened, I went to (name of health centre) and I also had my blood tested both at the cancer screening and at (name of health centre) both results said I was negative. I was happy that I screened for everything and I am not worried about my health up to now.*
77. I: Okay, after you were told that the results of your VIA screening was abnormal, you said they told you that the cancer cells had just started not so?
78. *R: Yes.*
79. I: So how did it make you feel?
80. *R: I was not worried because they told me that they did thermos-coagulation and that when they have done that it means the cells are dead. That was when I had courage and was not worried because it meant that they had assisted me. Because of the problems I had at home however, I was unable to come at 6 weeks, but that was the time I would have gotten my final results at that time. Today they have told me that after testing the biopsy which they took, they have not found any cancer cells. They have also told me to come again after 3 years to get rescreened.*
81. I: Alright, before they screened you they had already told you that if they find a problem they will do thermo-coagulation?
82. *R: Yes, they told us in advance. First of all, they did a urine test for pregnancy and the results showed that most of us were not pregnant. From there is when they screened us. they told us that if they find that the cancer cells have spread so much already, the person is referred to (name of hospital) hospital where the cervix is removed. But I still said that it is better if I get screened so that I know where I stand.*
83. I: Alright, but after they explained to you and told you that we are not doing any family planning here, we are screening for cancer and that if they find any cancer cells they will either thermos-coagulate or you will be referred to (name of hospital) didn’t you have any worries or concerns?
84. *R: No, I had no worries. I was determined to know my body’s status. I knew that if I get scared and yet the cancer cells are there, there is nothing I will benefit. That is why I gathered the courage to get screened. Most women were turning back but still most of us got screened.*
85. I: Alright, what do you think was done well? the way things happened on that day, what did you feel went well?
86. *R: I would say that I knew the status of my body in terms of cancer. So what went well is that I knew my body’s status from the results of the screening that was done. I can’t say that this is the good thing I experienced on that day because I was feeling pain when they were screening. I still see the good in the pain though because it’s not like am dead, I am still alive until today.*
87. I: Alright, but what do you think could have been done better than it was?
88. *R: Aaa, I did not notice anything.*
89. I: Okay, it could be the length of time it took or anything.
90. *R: Aa, it did take some time, I should not lie.*
91. I: Okay, so how did you feel about the time it took?
92. *R: Aaa, it was not worrisome though, I think it was about 30 minutes so I was not worried. After that they told me to come at central after 6 weeks.*
93. I: Alright, is there anything that you feel could have been done better on that day, from the time you arrived until you left?
94. *R: No, I just thought it wise that since I was already there I should just get screened, although it was not what I came for. I said to myself that this service is here so I need to make use of it and know if I have cancer or not. That is why I was encouraged to get tested.*
95. I: Alright, you told me that at some point during the screening process you would feel pain.
96. *R: [Chuckles] yes, I would close my legs even you know, it was painful [laughs].*
97. I: So maybe besides the pain, what else did you find as the difficult part?
98. *R: Mmm, nothing. From the time I stood up and went home, I did not have any difficulties or any other pain.*
99. I: Alright, and what part did you find to be easy?
100. *R: There wasn’t.*
101. I: From the time you arrived to the time you left for home.
102. *R: The easy part was when we were being assisted.*
103. I: What do you mean being assisted?
104. *R: The one who were educating us. after we arrived, we had a sensitization talk and that is what I found easy. From there to testing is what I found a bit hard since it was painful.*
105. I: Alright, is there anything unexpected that happened?
106. *R: No, there wasn’t.*
107. I: You knew everything that was going to happen?
108. *R: Yes, I knew that I am getting tested and afterwards if I have the cells then we’ll see what to do, if they say I don’t have it then that is it, at least I will have known. Knew that if I went back home maybe I will only realize it after it have spread, so it was better to know.*
109. I: It can be hard for people to come for follow-up, after they are told to come to the clinic after several weeks right?
110. *R: mmm.*
111. I: Tell me the challenges that you had coming for this follow-up visit?
112. *R: I was unable to come because we had misunderstandings with my husband, we were not on the same page. What was happening is after I tell him that I need to come, he was making his own conclusions. The condoms I was given for example, his conclusion was that I want to start prostitution. However, he was curious because the community tracer was coming to my home and he would meet my husband when he comes. After telling him that I need to go to the clinic all he was saying was that he will give me transport to come, but he wasn’t and that is why I was unable to come. This time around however, he read the consent I was given and finished it. After he read it is when he now said let us go there together. All I said was let’s all go so that you should really see what will happen there.*
113. I: Okay, what other challenge was there?
114. *R: No, that was the only reason. I mean if someone does not give you transport, where else will you get it [chuckles].*
115. I: Alright, thank you very much. What challenges do you think other women face which make them unable to come for their follow up visit?
116. *R: For most of them, when we were telling them that there is cervical cancer screening being done, they were refusing to come for whatever reason. We would tell them that we were screened and now they told us to go to central hospital, they would still refuse. It was as though you are telling them something bad and so most of them refused to come. Only 3 women in our village came to be screened, the rest refused.*
117. I: Okay, after you got screened and you were told to come after 6 weeks, those are the women am referring to. Those who got screened and were given a date to come, not those who refused to get screened. What are some of the reasons why such women wouldn’t come?
118. *R: For those women, we were going in to get screened one at a time and when they come out, after we ask them how the screening went, all they were saying was I am fine. I am the only one who opened up and told them that u have been told to go to central hospital because they found the cancer cells. All they said in response was that ‘you should go; we have been told that we don’t have cancer cells’. After that response, even if it was you, would you have the guts to follow up if they have gone to the clinic or not? It was also hard for me.*
119. I: Okay, but say they have been told to come to the clinic after such a time, what do you think could make them unable to come?
120. *R: Aa on that, I wouldn’t really know why they would fail to come. It could be the husband or any other reason so I wouldn’t know. As I said, when we left home, our intention was to get a family planning method so it is quite hard to know what a friend of mine experienced after she went home, it is quite hard.*
121. I: Alright, but how do you think we could do to help those who fail to come to follow up?
122. *R: Maybe if my husband also heard the counsel that you provide so that they easily understand when we are explaining to them. Currently my husband understands because they have explained to him in detail here and he now believes what I was telling him. The bad conclusions he was making have now been resolved.*
123. I: Alright, besides that, how else can we help?
124. *R: Perhaps if you went and called for a community gathering where you also explain these things. It would be hard for them to believe what I am saying for example, but if you called for a meeting then that would work. It could also be that other people still don’t know about cancer and they only realize it after it can’t be treated and yet they were doing chores and everything normally.*
125. I: Alright, did you discuss this screening with anyone else?
126. *R: Yes, I discussed it with some women from my village. Their response was that they would not it.*
127. I: That was after you were already screened?
128. *R: Yes, after I was screened. I tried to tell them that ‘there is cervical cancer and it is a disease that affects the cervix. The only way to know if you have it or not is to get screened’ and they said you go and get tested, we will not go. Then I just stopped trying to explain to them because of how they were responding, after all, everyone is responsible for their own lives. When they find a problem, maybe they will decide to go and get screened.*
129. I: Alright, besides the women, who else did you discuss it with?
130. *R: I told my relatives, but their response was also the same, they said no.*
131. I: Alright, how did your husband react after you told him that you went for cancer screening?
132. *R: He told me that it is hard to believe that is where I went since I brought home condoms. That was when I gave him the consent forms so that he should read and maybe be convinced but that did not work since he was angry. After a month is when I noticed him calm down and he read the letter in more detail until today when he said we should go to the clinic.*
133. I: Alright, regarding you being screened for cervical cancer, what did he think?
134. *R: I don’t know what his view on that is because he did not say anything. It would have been easier if he had told me, but he did not say anything.*
135. I: Alright, you said initially he was angry, but then after a month he seemed to calm down. When that happened, did he seem interest to know what happened?
136. *R: Yes, that was also when he started to read the letters carefully. He would call me to ask me what happened and how it all went, and I was explaining to him. Afterwards I would remind him to say let’s go to the clinic and he used to say he does not have money. There was nothing else I could do; it is his pocket. Today is when I heard him say that let us borrow some money and go to the clinic.*
137. I: Alright, did you also tell him the results of the screening?
138. *R: Yes, I also told him.*
139. I: Why did you tell him?
140. *R: So that he should know, he should not what to expect. [Chuckles] because its either the cancer will spread or it will be controlled, so I wanted him to be ready.*
141. I: Okay, any other reason?
142. *R: I told him so that he should not be surprised when he sees the condoms they gave me and I ask him to use it during sex. That was also why I told him.*
143. I: Alright, after thermo-coagulation treatment we advise against not having sex for 1 month to allow for healing, was this a challenge for you?
144. *R: After I told him, it was difficult for him, he did not understand. As a result, we were arguing a lot in the house. We managed to stay for 2 weeks without sex but that was because he was angry. The third week however things got worse [chuckles] and then we had sex. So it only worked for 2 weeks and that was because he was angry. Everyone was focusing on their own things for that 2 weeks.*
145. I: Alright, how did that make you feel though? After being told that you should not have sex for a week then you tell your husband and he gets angry over that; how did that make you feel?
146. *R: I was hurt because I felt like he was depriving me of my rights.*
147. I: In what way?
148. *R: I was told that they found cancer cells and they did thermos-coagulation, then they told me to wait for six weeks without having sex. After I told my husband he asked me to say ‘is it possible for people who are married to stay for six weeks without having sex; how is that even possible?’ All I said was that is what they told me. Then after 2 weeks I noticed that eeh [chuckles] he was not going to manage.*
149. I: Okay, so how do you think we can help for such problems not to happen?
150. *R: I don’t know what you can do honestly.*
151. I: [Chuckles] Just think about it, what do you think we can do?
152. *R: It is hard for me to make suggestions because even me, I failed for there to be an understanding in the house. Maybe if the husband would be understanding enough to use condoms, maybe that could work. In my case however, he even refused the condoms so it was hard for me to know what to do.*
153. I: Alright, do you think male partners should be more involved with cervical cancer screening for women?
154. *R: I could respond and say yes they should be involved, whilst the partners don’t think so. As such, it is hard for me to give a response because I do not know what my partner would think. I might say yes they should take part, but then my partner refuses to take part, how can that work?*
155. I: Okay, but you know what is done when you come for screening, so from your point of view, do you think they should take part?
156. *R: My partner told me today that this is the last time he is coming here, with those words would you even be encouraged to say they should take part [chuckles].*
157. I: Why did he say he will not come again?
158. *R: All he said was that I should be coming here alone and not with him.*
159. I: Why not?
160. *R: I don’t know, maybe he is now convinced. From that can you say that my partner is taking part? No, the only way for them to be involved is if you gave me medication and told me that my husband should be taking it as well, that’s when it could work I think.*
161. I: Alright, you said before you got screened you were counselled not so?
162. *R: Yes.*
163. I: On that day, let’s say it was not just you, but the both of you were there and he listened to counselling; do you think there would have been any benefit?
164. *R: Yes, he would have been convinced, so that would have been the benefit. When I tell him, he thinks that I am lying so if he was there then he would have heard it for himself.*
165. I: Alright, in future, if there are more screening for cervical cancer, do you think the male partners should not take part or not?
166. *R: They should, and because they are now convinced that what we used to tell them is true, they can really take part.*
167. I: What role would they play?
168. *R: I don’t know role the role they would play; I think it would be better if they suggested the roe themselves because it is hard for me to explain.*
169. I: Whether they agree with the role or not; what role do you feel they could play?
170. *R: Maybe by agreeing to wait until the six weeks is over before they can have sex with their wives; I think that would be helpful.*
171. I: Alright, what else?
172. *R: [Laughs] aa, it is hard to explain.*
173. I: Alright, do you think they should be there during screening?
174. *R: Men should not be there because it was mostly male doctors who were screening us. Do you think a man would allow another man to do tests on his wife?*
175. I: Why not?
176. *R: That would not work, most husbands would not agree.*
177. I: Okay, if it were a woman what would happen?
178. *R: Maybe that could work, he would know that it I a fellow woman.*
179. I: Alright, how do you think we could encourage these partners to be involved?
180. *R: You can encourage them by informing them that because the women has the disease, if they insist on having sex with her before six weeks is over, they could contract the disease as well. they can transmit the cancer to each other because during sex, the man’s penis reaches the cervix, meaning there is every chance for the man to get cancer as well.*
181. I: Alright, what is the best way of educating men about cervical cancer?
182. *R: The best way is by going together with them and getting the advice together with them so that they can easily get convinced. Most wouldn’t believe what you are telling them, but if you all hear what is being said it can work better.*
183. I: Alright, is there anything new you have learned about cervical cancer or cervical cancer screening that you did not know before the study?
184. *R: Yes, I did not know that people can have cervical cancer, I had never heard of it.*
185. I: Okay,
186. *R: I heard of it the same day I got screened when I had planned to change my family planning method.*
187. I: Alright, what else?
188. *R: It’s just that, I did not know that there is such a thing as cervical cancer. I thought it can only develop on the legs and the hands but never on the cervix.*
189. I: Alright, did you also learn the causes of cancer?
190. *R: Yes, they said one of the causes is if you buy medicine which supposedly helps to tighten the vagina, they said that can cause cancer. They also said that if you use soap for the vagina it can also cause cancer, and I did not know all that.*
191. I: Alright, who do you think should be screened for cervical cancer?
192. *R: I think I could encourage my relatives to get screened.*
193. I: Okay, what do you mean?
194. *R: I have my elder and younger sister; I would encourage my older sister to get screened because she is still of child bearing age. It can happen that after she gets pregnant, they realize that she has cervical cancer. I would therefore encourage her so that she should know where she stands.*
195. I: Okay, besides your relatives who else do you think needs to get screened?
196. *R: I would also tell the women in my community of the need to get screened before it’s too late to know whether they have it or not.*
197. I: What kind of women?
198. *R: They told us that even an old woman can have cancer. It does not matter whether the person is young or old, they need to get screened.*
199. I: Meaning every woman?
200. *R: Yes, every woman.*
201. I: Okay, how often should they be screened?
202. *R: It should be often because sometimes they can say we have done thermo-coagulation and yet the cells did not die. You therefore need to be screened often so that if they find that the cells have spread, they can refer to Ethel on time.*
203. I: Okay, so the woman should be screened again after how long?
204. *R: They said after six weeks.*
205. I: But what do you think? How long should it take?
206. *R: I think after 4 or 3 months, and not 6 weeks.*
207. I: Why not 6 weeks?
208. *R: [Laughs] I don’t really know what others would think, I am only saying what I think would be better.*
209. I: Okay, exactly who should be screened at this 4 months? Those who were told to come after six weeks or those who have never been screened before or those who were screened but no cancer cells were found?
210. *R: At 6 weeks those who should come are the ones who were scheduled to come.*
211. I: Alright, what do women in your community think about cervical cancer screening? You have told me that some refused to get screened and other did, but what are their thoughts on the screening itself?
212. *R: I don’t know what they think of it, I could say that this is what they think and yet it is not. Some refused to get screened because they hadn’t told their husband about it and yet some of us also hadn’t told them but we still got screened.*
213. I: When your chatting, what do they say about it?
214. *R: They say that they cannot get tested, they say it is all fake. Others say that I need to go and tell my husband first and others say that they are in their menstrual periods and so they can’t get screened.*
215. I: Alright, for those who say that it is fake, why do you think they say so?
216. *R: I don’t really know; I think it is because they have never been there. I think once they get screened and the get their results is when they would believe that these things are real.*
217. I: Alright, but do you think they understand the importance of cervical cancer screening?
218. *R: I think they do because some of then turned back after they had arrived at the place and had been sensitized.*
219. I: Okay, and the others?
220. *R: The others did not even go there, they completely stayed at home.*
221. I: Okay, so some understand whilst others do not?
222. *R: Yes, they are in 2 groups.*
223. I: Alright, in your opinion, do you think that women are interested in receiving this screening and treatment service?
224. *R: It goes back to what I said that I can think that they are interested but I wouldn’t really know because there is no way of telling what they are really thinking. I made a decision and went to get screened, but I don’t know if the others are interested or not.*
225. I: Yes, you can’t know what they are really thinking, but from the way they respond or react, do you think they are interested or not?
226. *R: They are interested; some women would find me home after they went to get screened but did not find anyone. Some would say they had stopped screening and so they did not find anyone but whether that is true or not, I wouldn’t not. There are other people like my niece, she came here and got screened here, so they are interested.*
227. I: Alright, why do you think someone would not want to be screened, any reason you can think of?
228. *R: Some thought that the screening is related to HIV testing and so if they go there they might be found with HIV. Some gave excuses about their husbands. They did not know that this screening was different from the HIV testing.*
229. I: Okay, apart from that, why else do you think other people wouldn’t want to get screened?
230. *R: I think they would not want to get screened because they do not experience any problems in their body forgetting that diseases like cancer take time before they show any signs. you wouldn’t know you have cancer if you don’t get screened, they even told us that you can have cancer for 10 years before it starts to show. So most people were not bothered because they feel fine.*
231. I: Alright, do you think it is possible that they were afraid of being found with cancer cells after they get screened?
232. *R: Yes, it is possible and they were afraid that they would be worried or concerned if they are diagnosed with cancer. To avoid being worried then maybe they opted not to get screened.*
233. I: Alright, do you think there is any stigma associated with being screened positive?
234. *R: No, there isn’t. We have had cases were people have died of cancer before, but people went and even slept there, so there is no stigma.*
235. I: Alright, what are some of the barriers that women might face in receiving this service?
236. *R: Ee, I cannot explain that, I would lie. I have never been asked that question so I don’t know.*
237. I: [Chuckles] It’s not about whether you have been asked before or not, you can tell that such a person fails to do this because of this reason or they are able to do this because of this reason, not so?
238. *R: Yes.*
239. I: So regarding this cancer screening, what do you think are the barriers that women can face?
240. *R: Maybe one of them could be when you are told that you should not have sex for 6 weeks and yet they cannot manage. There are some men who would understand and others who would not understand so that could be a barrier. Another reason is that when I know that I have cancer, I will be worried and my daily life would change because of that. That can make some women to decide not to get screened and wait for the cancer to show signs.*
241. I: Alright, in your opinion, how should cervical cancer screening be provided to ensure that more women can get screened? You have said that only four women from your village got screened.
242. *R: Yes.*
243. I: So how do you think the screening should be provided so that a lot more women than that are screened?
244. *R: Maybe by visiting them.*
245. I: Visiting who?
246. *R: The women or conducting community sensitization so that more women are aware. You can even pass through the chiefs and then the chiefs can use their people to pass the message along. The women would then be urged to go and get screened in large numbers because the message is coming from the chief and not mere people like me.*
247. I: Alright, those are the things that we can do not so?
248. *R: Yes.*
249. I: What can you do, how would you encourage them?
250. *R: I would encourage them yes, but coming from me, everything I say is useless.*
251. I: Okay, how do you encourage them?
252. *R: I tell them that when we hear things, it is better to act and take part. In my case, I went for family planning but whilst there I found a cancer clinic where after I got screened, they told me that cancer cells are developing. From there they assisted me and did thermos-coagulation and they have told me to go to central after 6 weeks. After telling them that, I don’t know if it is out of fear or what, they respond by saying that I am lying. ‘How can a healthy person like you have cancer, you have other reasons for going to central hospital and not for cancer’. With that, how can you encourage them since they think you are lying.*
253. I: Regardless all the things they were saying, what still encouraged you?
254. *R: I was screened and I was told my results and the next steps that I should take. That is why I came here.*
255. I: Alright, let us now talk about self-collected vaginal swab for cervical cancer screening. It is a new method has been developed for cervical cancer screening. This new method involves having a woman collect a swab from her vagina and submitting it to a health facility for testing whenever she can. Do you understand?
256. *R: Yes.*
257. I: However, unlike VIA which you had, the woman would not get her result immediately, you would have to return to health facility to get your result a few hours later or the next day. Have you understood this method?
258. *R: Yes.*
259. I: What do you think of this idea where the woman is self-collecting her vaginal swab and submitting to a hospital when she can for cancer screening?
260. *R: I could do it and submit at the health center.*
261. I: Why do you say that?
262. *R: So that I should know if I am cancer free or not*.
263. I: What benefit do you see in this idea?
264. *R: The benefit is that I would know, that time all they told me was that they found the cells and they have thermos-coagulated. Today, they have told me that they did not detect any cancer cells in the biopsy they took. But, they have told me that there are a lot of things involved for cancer to develop and it can happen that the cells can develop again. I would therefore do this so that I should know my status.*
265. I: Alright, where do you think the self-collection should happen?
266. *R: It can be a home or here at the clinic, mainly I think at home would be better.*
267. I: Why at home?
268. *R: Time consuming.*
269. I: Explain that for me.
270. *R: [Chuckles] if I was to do it here, does it mean I would have to go back home with it or?*
271. I: Oh okay, after collecting, you will submit it to the health center and come back maybe the next day or wait for several hours. Do you still think doing it at home is better?
272. *R: Yes, for us to come here it is very costly, so it would be a burden to come back here and get the results.*
273. I: Alright, how would you compare the idea of self-collection to the VIA screening you went through?
274. *R: The difference is that with self-collection I will leave the swab here until I get results.*
275. I: Which method would you prefer?
276. *R: The self-collection one.*
277. I: Why that one?
278. *R: I will not feel any pain [chuckles]*
279. I: [Chuckles] alright. what do you think the women in your community would thing about the idea of self-collection?
280. *R: Those women have never been screened. I think for them, they need to first of all come here and undergo VIA like we did.*
281. I: Okay, but of the two, which one do you think the women would prefer?
282. *R: I think the self-collection method.*
283. I: Why do you think so?
284. *R: After we explained to them concerning VIA, maybe some were afraid of the whole procedure and all the things it involves. With this one, they would be interested because it is an easier method.*
285. I: Alright, what challenges do you think would women face in self-collection technique?
286. *R: The challenge would be that after you do the self-collection maybe you will not have collected enough, if you submit that swab I don’t think the results will really be conclusive. So I think this would only work if you have been screened before. If you have never been screened by the doctors and you just get the swab, maybe it won’t work. Especially if you go to (name of health centre), they will just tell you that you don’t have cancer and yet you did not do the collection well. There is need for doctors to examine you first through VIA before you can do this self-collection.*
287. I: Alright, besides that, what do you think would make a woman unable to do the self-collection?
288. *R: Some would be lazy and say ‘how can you detect cancer in this way’? and so some will not do it.*
289. I: Alright, what are other reasons, if any, why you think women would not want to self-collect?
290. *R: Some would prefer it to be done by doctors, for them to trust the results. This self-collection is done by the individual who knows nothing about medicine, so people would find it hard to believe and they would prefer VIA where it is only skilled doctors who can do it.*
291. I: Alright, let us now talk about your recommendations for the future of the National cervical cancer screening in Malawi, just your recommendations right?
292. *R: Yes.*
293. I: In your opinion, should MOH consider including self-collected vaginal swab for cervical cancer testing to the cervical cancer screening program?
294. *R: Yes, they can.*
295. I: Explain that.
296. *R: After doing the self-collection, you bring the swab here. if you bring it here, at a hospital like this one, you would really know if you have cancer or not unlike if you went with the swab (name of health centre). But, because of transport problems, it would be hard for one to come here with the swab, that is what I think.*
297. I: Okay, so this should be considered?
298. *R: Yes, it should.*
299. I: But the swab should be brought here and not at other hospitals?
300. *R: Yes, because at this hospital they will be in a better place to know if you have cancer or not. At (name of health centre), they would just look at the swab and make conclusions without doing any tests.*
301. I: Alright, you also talked of transport issues right?
302. *R: Yes.*
303. I: What can we do to help out with this?
304. *R: If they could come to our households or provide transport for the person to come here and be screened.*
305. I: It has to be done here?
306. *R: Yes, at this hospital.*
307. I: Okay, so this idea should be considered, but it should only be done here?
308. *R: Yes!*
309. I: And where there are transport issues transport has to be provided?
310. *R: Yes, to pick up the person and bring her here to be examined, when done they drop her off.*
311. I: Alright, I understand. But do you think that would make it easy for most women to get screened?
312. *R: Yes, it would be easy because you are assured that you will travel safely. A lot of people would come for screening because now that the screening in the villages stopped, some people still want to get screened just that they don’t have the money to come here. Once they hear that there is a car that comes to pick people to and from the hospital, a lot of people will come.*
313. I: Okay, so let’s say a lot of people really come and they are brought here to be screened, between the two methods; VIA and self-collection, which one would they prefer?
314. *R: They would prefer VIA. It would depend on the individual, some would choose VIA and others would go for self-collection.*
315. I: What do you think would be the reason for them choosing VIA?
316. *R: For them to easily trust the results. They would think it is better for them to get screened and get their results right away, than for them to have to wait a while before they know their status.*
317. I: Alright, thank you very much. What groups of women could be most suitable for self-collected vaginal swab for cervical cancer testing?
318. *R: Mainly those of child bearing age. For those who are on menopause, it takes time for them to release any vaginal fluid since they even stopped menstruation. This method would work easier for those who can still bear children because their discharge is not a problem. You know the discharge is followed by the menstruation.*
319. I: Alright, besides those on menopause, which groups of women do you feel would not be suitable?
320. *R: Suitable for what?*
321. I: For the self-collection method.
322. *R: Aa, they would choose for themselves. I cannot really decide for them.*
323. I: Okay, thank you. We have talked quite a lot, but do you have any questions before we finish?
324. *R: No, I don’t have any question, I have understood everything and I know that I have a role to play in encouraging other women to come and get screened. Those who are willing will come and those who are not willing will not come.*
325. I: Alright, any comment?
326. *R: My comment is that I am grateful because you have taught me things that I did not know. The self-collection method for example, I did not know of it, but now I am interested in doing it.*
327. I: Alright, but remember that the self-collection method is only an idea and it has not been implemented right?
328. *R: Oh okay.*
329. I: Yes, that is why I was asking if you think it should be considered.
330. *R: Oh okay, yes it should. You can just get the swab to the hospital and get your results, so it is good.*
331. I: Alright, thank you very much for your time today.
332. *R: Thank you so much.*

THE END
