# Supplementary material for: Perceptions of cervical cancer and motivation for screening among women in Rural Lilongwe, Malawi: A qualitative study
Source: PLoS One. 2022 Feb 7;17(2):e0262590. doi: 10.1371/journal.pone.0262590 (PMC8820632; doi:10.1371/journal.pone.0262590)
Supplement: S3 File — (ZIP) [file pone.0262590.s003.zip › VIA_351 Missed.docx]

**PID: VIA_351**

**INTERVIEWER ID: 466**

**DATE OF INTERVIEW: 14 Dec 2017**

**TYPE OF INTERVIEW: Missed 6 and 12 weeks follow up visits**

**KEY: I= Interviewer, R= Respondent**

**Interview summary:**

This is the participant who missed her scheduled visit date in (date and month). According to her, she did not understand where she was supposed to go when she was referred to go to KCH. With her previous experience, she thought cancer issues are dealt with at (name of hospital) and that was where she went on the specified date. Regarding cervical cancer screening experience, the participant said that she decided to go for screening because she had been experiencing abnormal menses. When she went for screening, she was told that she had cancer cells at an established stage and she was referred to go to (name of hospital) for management. When she went to hospital, she was scheduled for operation which had been deferred but finally she had her uterus removed. She feels some women may fail to go for their follow up visits because of sicknesses, death or lack of transport money. In terms of support, she said that her partner is not very supportive because he likes drinking beer. She said that women in her community are very willing to go for cervical cancer screening because they have experiences of people who have suffered from cancer. She said that some women do come to the hospital to get screened so that they know if they have cancer cells or not. Regarding the self-collecting vaginal swabs, she said that it is an easy and simple method because women who are ashamed to be seen by doctors can easily use this method. She however said that some women would be ashamed to carry the swabs to the hospital and this would prevent them from collecting the swabs. All in all she recommended that the government of Malawi should consider including self-collecting vaginal swabs as a method for cervical cancer screening so that women should have a choice between the VIA and self-collecting vaginal swabs method.

**Interview text:**

1. I: Thank you for meeting with me today. I really appreciate your time and your input will be very helpful. I am working with a team of researchers from the University of North Carolina Project in Malawi but this project is from America. So we are working here in Malawi.
2. *R: Okay.*
3. I: Sorry you were not able to come for your 12-week follow-up appointment right?.
4. *R:Yes*
5. I: However, we would still like to hear from you regarding your experience with the cervical cancer screening with VIA and thermo-coagulation treatment of the cancer cells.
6. *R:Okay.*
7. I: We would also like to hear about any difficulties you had afterwards or any challenges you had to coming for your follow-up visit right?.
8. *R: Yes.*
9. I: Your input is also important to help us understand how best we can conduct cervical cancer screening campaigns in Malawi. So mostly it is about the campaign right, so that many people should know about the service and that they should be able to access the service. So there is no right or wrong answer. Anything that you say is very vital. We rely on your information. Everything you say will be confidential and that is why you see that we are the two of us here right?
10. *R: Yes.*
11. I: And they will only be used to make this health program and health questionnaire like this one better. I will audio record this interview to help me remember what was said, but your name or any identifiable information will not be connected to anything you say. So firstly I would like to know about your VIA screening and Thermo-coagulation treatment experience: Can you tell me your understanding of the cervical cancer screening and treatment you received that time?
12. *R: Like in August?*
13. I: Yes.
14. *R: Okay, when I was referred to come here, I did not know that I was supposed to come here. I went there at* (name of hospital)  *and when they saw my card, they asked; “did they tell you to come here?” I said “Yes.” The after seeing the card they welcomed me and examined me and they saw that what the cervical cancer screening team found in me was true. That was on 5th of (month)r and they told me to come on 14 September and they told me that “We will do the operation on 18 of (month). So I came and I spent a week. When the date was due for operation, they told me that the machine was out of service. So they rescheduled me to 28 September and they told me that “We will do the operation on 2 (month)” So I came and on 2 (month), the operation failed again. They said “You should go, we will give you another date. I was very worried because I was coming with my daughter and transport was a problem because we were coming from far. I told them that “But money is a problem and we having difficulties to find money to come here frequently.” They said “You will find means to have transport to come here.” We were having difficulties. Sometimes we could go to relatives to Area 36 so that they could assist us. After explaining to them our situation, they could understand us. So when we came on 2 November, they told me that “We will do the operation on 6 November” and I was operated on 6 November.*
15. I: So I want to know your experience of the screening and Thermo-coagulation experience that you had when you were screened that time in the community before you were referred here. I wanted to know your understanding of cervical cancer screening and treatment.
16. *R: In terms of treatment, I cannot say much…*
17. I: What happened that time?
18. *R: For me to be found with cancer cells?*
19. I: That they should screen you… I want to know because I was not there so I want you to tell me like a story of what happened.
20. *R: True. When they screened me at (Name of area), they found that I had blood then they asked me “Are you still menstruating?” I said “Yes.” When they screened me they saw that I had cancer and they told me that we should document outside there. After documentation, it was when they referred me to come here and that was when I came. In terms of Thermo-coagulation of the cancer cells, I cannot say anything because I don’t know what happened.*
21. I: Can you explain to me the screening process; how did it go?
22. *R: Okay. what happened is that I had Tubal Ligation in (Year). Since then I have been having menses every month and it has been a heavy one. Some of my age mates had stopped but I have been having it. Sometimes I could menstruate twice or three times a month. Then I said “This is not a normal way of menstruating, I hear there is cancer and this could be cancer because most of my friends stopped menstruating. Sometime in future I will be found with cancer.” So this time they found us chatting in a group and told us about the cervical cancer screening. I said “This is my chance to get screened because of the problem I am having in my body.” So I went leaving my friends where we were. When I went there, I was found with cancer cells and I said “You see, they have found me with cancer and they are going to treat me but if I had not gone there, nothing would have happened and I would realize when the cancer has metastized and eventually I would die. But now look they will treat me and I will have a healthy life.” It was when I started following their instructions; “You should go to KCH, do this, do that up to the time when I was operated. That was what happened.*
23. I: Okay. So when they told you that you had cancer cells, what did they do?
24. *R: They just screened me and found me with cancer but that I had cancer cells that I don’t know how they did it. When they found that I had cancer, it was when they referred me to come on 15 of (month); when I came here they also screened me and confirmed that I had cancer and they scheduled me for operation. I was very worried when they were scheduling me and reschedule for another date. I was worried to say “These delays will enable the cancer to spread but luckily it did not spread. For others they were not able to do the operation because the cancer had spread all over.*
25. I: Okay. so we would like to hear your thoughts about cervical cancer screening campaigns like this one where you got screened; We know that you were assisted here but we would like to know more about what happened in the community. why did you choose to get screened or participate in this study?
26. *R: It was because of how my health has been because sometimes you just think it is a simple problem when it is a big problem. That was why I took courage to be screened and that was when I was told that I was found with cancer. To say the truth, people admire me because I was found with cancer before I was sick and bed ridden. I was strong and health when I was found with cancer.*
27. I: What else were you worried about apart from the abnormal menses?
28. *R: It is because in our family many people suffer from cancer and that was why I was quick to decide for screening. I did not want to go there when I am weak; while strong, I decided to go and know my status.*
29. I: *What had you heard about the screening?*
30. *R: Hearing from people?*
31. I: Yes.
32. *R: I did not see from people; it was because I had seen it with my eyes. My mother also suffered from cancer. She was discharging watery fluids and they told us that we had delayed in seeking health care. I was with her at* (name of hospital) *there until when she died last January. My last born also died of cancer when he was eight years and three months. He died in (year).*
33. I: Whose child was this?
34. *R: My last born. I was with him here also.*
35. I: What type of cancer was that?
36. *R: It was cancer of the blood because he was anemic every week. When he was transfused blood, few hours later he was anemic and they told me that he had cancer. I believed because even his stools showed that his lungs and kidneys were destroyed. With that experience, I said “No, cancer is real; I have experience of the death of my mother and son. Let me go for screening and see how they can help me.” I was here with my child and the doctors reached the extent of telling me that “There is nothing we can do, all the internal organs of this boy are destroyed; you should just go home on palliative care.” And it was true because I even accepted that there is nothing that we were going to achieve. I went home on Wednesday, they next Wednesday the boy was no more. So it is not something that I have seen from other people but my personal experience. I have seen my people dying of cancer and I thought the earlier the better, I should be screened and see how I can be assisted rather than being screened when there can be no solution to the problem.*
37. I: Okay. Maybe there are some misconceptions which people were talking in the community about the screening?
38. *R: They were saying a lot of things like “Even though they should remove the cancer cells, there are some remaining cells which are untreatable and they kill.” And I said “Whether some cells will fail to be removed or not, I am going for screening and God will help me through.” They were talking a lot “It is a sexually transmitted infection. And my drunkard husband when he is drunk “You are a prostitute, you contracted cancer from men..” “Okay, because you have said it.” That is what they say.*
39. I: Okay. So apart from that, what else have people been talking about the screening?
40. *R: That was the only thing.*
41. I: When you heard that the results of your VIA screening was abnormal, how did it make you feel?
42. *R: I was worried and that was why I decided to follow the instructions from the doctor. When they told me that they had found me with cancer I said “It is important that I follow the instructions that they have given me so that I should lead a healthy life.*
43. I: Okay. How can you describe this worry? Was it fear or…
44. *R: Firstly it was fear and I was feeling sorry for myself. However, because of the way I have been having menses, I knew that “one day in future, I will be found with cancer.” So I had been worried that I will be sick and I will die but with time, I accepted it up to the time when I got screened. So fear was there as a human being.*
45. I: During the time when you were screened, what was done well?
46. *R: Everything went on well.*
47. I: For the sake of someone who is not here but will read what you are saying; what is it that went on well?
48. *R: What can I say? Because when I entered the room I was told to lie down and I was screened then told me what they found. So I saw that everything went on well.*
49. I: Perhaps how was the place where you were being screened?
50. *R: It was good.*
51. I: Time when you were screened?
52. *R: It was in the afternoon.*
53. I: How did you perceive the time taken for screening.
54. *R: It was not long.*
55. I: How about the treatment?
56. *R: I was not given any treatment, they just documented.*
57. I: You did not get the Thermo-coagulation treatment?
58. *R: No.*
59. I: But other women were Thermo-coagulated?
60. *R: Yes.*
61. I: Okay….
62. *R: They just saw that the cancer was in a developed stage and there was need to remove the uterus. They did not give me the Thermo-coagulation treatment; I don’t know it.*
63. I: How was your discussion with the research staff?
64. *R: The interaction was very good.*
65. I: How good was it?
66. *R: They were explaining to us; “We want to examine you, we have found you with cancer, this is what you should do, we are going to refer you to* (name of hospital)  *so that we can assist you…” I was just following what they were telling me.*
67. I: Sometimes the attitude…
68. *R: … No, they were not selfish; they were understanding and knew how to help a person.*
69. I: Okay. What was the easiest part?
70. *R: The screening part was very easy because they just insert something and take it out; it did not take a long time. I did not have a strong pain and I did not cry when coming out of it.*
71. I: What was the difficult part?
72. *R: Nothing was difficult.*
73. I: Was there anything unexpected?
74. *R: Very much so.*
75. I: What was it?
76. *R: That I was found with cancer because I thought I was born and I would die of another illness rather than cancer but that it happened that I was found with cancer, I just accepted that it had happened and there is nothing I can do about it. In our community we have been seeing people suffering from cancer like the wife to our chief suffered from cancer but she delayed to seek medical care and she died.*
77. I: Okay fine. Now I want us to talk about follow-up challenges. You have explained to me that when they saw that you had cancer cells on your cervix, they referred you to KCH for further management right?
78. *R: Yes.*
79. I: So I want to talk about you and other people. It can be hard for people to come for follow-up; tell me why you were unable to come for your 12 week follow up?
80. *R: It was misunderstanding on my part. When they said that I should go to* (name of hospital)*, in my mind I was thinking about* (name of hospital) *because that was where I went with my mother and that was where I went on the 15th of (month). Had it been that they gave me the right directions to this place or that they gave me a phone number so that I could be communicating with them, I would not have missed my visit. They just said “You should come to* (name of hospital)*” and by* (name of hospital) *I thought* (name of hospital)*.*
81. I: We know that misunderstanding played a major role in your missing the study visit but then What challenges do you think other women have to come for follow-up?
82. *R: If they can be given the right directions they can come.*
83. I: Suppose they give them the right directions but still they fail to come, what can be the cause?
84. *R: It means she will just decide not to come. These women don’t miss. I remember there is a certain woman from our community, she also came and she was just Thermo-coagulated they did not remove the uterus. Whoever is referred here comes.*
85. I: So imagine that the woman was ready to come for her follow up visit but then there has been a challenge that prevents her from coming to the hospital for her follow up visit; what could be this challenge?
86. *R: Maybe if there can be funeral or her relative is very sick or she went somewhere else where there is an illness or funeral maybe she cannot come.*
87. I: What else?
88. *R: Maybe she can have no transport money to come here. I was strong because I reached the extent of exchanging with my wrappers; when I found the money I was getting the wrappers back. Right now I have one wrapper which I have not taken back. I said I should not fail to go to the hospital because I had no money; I was just doing that unless there was funeral all sickness but to fail to come to the hospital because I did not have money; that I did not allow to happen.*
89. I: So you have mentioned about funeral, sicknesses, lack of transport; what else do you think could be a challenge that could prevent women from coming to their follow up visits?
90. *R: Maybe some people discouraging them from coming here?*
91. I: So how best can we help women overcome those challenges?
92. *R: If these women can be coming from my community, I can be encouraging them. I can be telling them “Go,” “I have no money…” I can be telling them that “Borrow money and go.” I can be encouraging them.*
93. I: Is there anything else that can be done by you or health workers?
94. *R: Like what?*
95. I: Like helping women to overcome their problems?
96. *R: They can be coming I am sure.*
97. I: Okay. Now I want to ask about partner and community support; Did you discuss this screening with anyone else?
98. *R: No.*
99. I: No?
100. *R: I did not tell anyone else that “Oh, I am going for screening” no. But when I was found with the cancer cells, I told my partner that “I went for cervical cancer screening and I have been found with cancer cells.”*
101. I: What did he say when you told him?
102. *R: He accepted.*
103. I: How was the discussion?
104. *R: I told him what happened and he said “Go there, this is a fatal disease and we are lucky that they have found it at this earlier stage when it can be treated. It could be difficult for us if you were very sick.” We came together here and he is outside there.*
105. I: Did he have any questions?
106. *R: He asked; “Why have you been found with this disease?” “It is because of how I have been menstruating. That was a sign that one day I would be found with cancer. So here I am they found the disease in me. I should just follow instructions from the hospital.”*
107. I: Okay.
108. *R: Yes.*
109. I: So what does he think about this?
110. *R: He thinks that “My wife is still sick because they removed the uterus” and we are just staying without having sex.*
111. I: What do you mean by that?
112. *R: He knows that I am still sick since they removed my uterus and he does not demand sex from me.*
113. I: Oh, okay. Do you need his approval on anything?
114. *R: No.*
115. I: What support do you need from him?
116. *R: Nothing.*
117. I: Why?
118. *R: He is a drunkard and he does not provide even transport for me to come here. I have to look for it myself. I have come with him he is drinking water there; he drunk a lot yesterday. I have come with him because they said “You should come with your partner,” I said maybe because the disease is somehow sexually transmitted, let me go with him.”*
119. I: Okay. So what does he think about cervical cancer screening?
120. *R: He thinks they did a commendable job to remove the uterus.*
121. I: What does he think about the screening itself?
122. *R: He has a very positive attitude of screening.*
123. I: Does he seem to learn more?
124. *R: Yes.*
125. I: How do you know?
126. *R: His interest is not explicit because he is always drunk. I just tell him; “I went.., I am back…”*
127. I: Fine. So you have said that you discussed your screening results with your partner….
128. *R: Yes I told hi that “I have been found with cervical cancer” and he said “Okay, go to the hospital so that you should be assisted.”*
129. I: How does he support you?
130. *R: He does not support me in any way. What he knows is to drink.*
131. I: But he has escorted you today…
132. *R: He has come because they said he should come; otherwise even the time when I was admitted there, he never came. I was with my daughter that was all. He had no money.*
133. I: So money was his problem?
134. *R: Money…*
135. I: Not that he did not want?
136. *R: Money was a problem.*
137. I: Okay. so the way you know your partner, how can you describe him in terms of the support he provides to you?
138. *R: There is no support. He does not support me.*
139. I: The way you know him, how concerned is he with your problem?
140. *R: He just says that “You should not be working, let the wound heal first.”*
141. I: So who does the work?
142. *R: We have not even worked in the garden this year because he just goes for piece work and when he finds money he uses it to buy beer.*
143. I: Fine. You said that Thermo-coagulation treatment was not given to you?
144. *R: No.*
145. I: Were you not told to stay for one month without sex?
146. *R: No I don’t know about that. However, on my own I decided not to have sex.*
147. I: You were not told anything when they were screening you in the community there?
148. *R: No.*
149. I: Okay.
150. *R: They gave me condoms and I said “I am not sure if he will be able to use them because he is a drunkard.”*
151. I: When were you supposed to start using the condoms?
152. *R: That same day.*
153. I: That same day?
154. *R: Yes and in the future.*
155. I: Do you think male partners should be more involved with cervical cancer screening for women**?**
156. *R: I don’t know how they should be involved; maybe you will tell me.*
157. I: I want to learn from you and I am talking about men in general not only your partner; how do you think they should be involved in the screening of cervical cancer screening for women?
158. *R: I don’t know how to answer that one. Maybe they should be doing the work at home.*
159. I: That is why I have told you that anything that you tell me is right; don’t doubt yourself.
160. *R: As I am sick like this, he can just be doing the work at home.*
161. I: How else?
162. *R: In terms of sex, sometimes he can have the feeling for sex but he should know that “My friend is sick.”*
163. I: What else, for men in general?
164. *R: That is all.*
165. I: So how do you think *we can include men more?*
166. *R: If you can provide the information, if we tell them they can be motivated.*
167. I: How can the information be provided to them?
168. *R: To men?*
169. I: Yes, how can they be told?
170. *R: Telling them.*
171. I: How?
172. *R: Just telling them.*
173. I: What method can we use to tell them about cervical cancer screening for women and that they should be taking part?
174. *R: I don’t know what method can be used.*
175. I: Fine. Now I want us to talk about knowledge and personal risk assessment of cervical cancer; Is there anything new you have learned about cervical cancer or cervical cancer screening that you did not know before the study?
176. *R: What I did not know?*
177. I: Yes.
178. *R:I did not know that I could be found with cervical cancer.*
179. I: What else?
180. *R: That is all.*
181. I: If you were to respond for other women; what is it that they don’t know about cervical cancer?
182. *R: Because of their lack of knowledge.*
183. I: Their lack of knowledge?
184. *R: Yes, because they are not willing to come to the hospital.*
185. I: Okay. Who do you think should be screened for cervical cancer?
186. *R: Like in our community?*
187. I: Yes.
188. *R: There are many women and a lot of them are still going to* (name of hospital)  *to seek the service.*
189. I: Like which group of women?
190. *R: The child bearing group or women of my age. They are going because they want to know if they are affected or not well in advance.*
191. I: Is there another group of women who you think should be screened?
192. *R: Those are the ones I can think of but if others would want to be screened they would still go and get their results.*
193. I: I meant the size of women you may think of…
194. *R: Any woman whether old or young, they can still come.*
195. I: So what would be the eligibility of these women?
196. *R: [No response]*
197. I: because you have said “whether old or young…” so what could be the eligibility for these young or old women for them to be screened for cervical cancer?
198. *R: They have to go to the clinic to be screened.*
199. I:Fine. So how often should these women be screened?
200. *R: Whenever they may want.*
201. I: I want to hear you input so that if it changes in future, you should be able to say that “I contributed to this change…”
202. *R: [No response].*
203. I: How often do you think they should be screened?
204. *R: Frequently.*
205. I: How frequent? Like how many months apart or after how many years?
206. *R: Some say after three years… they say like that.*
207. I: Those are the opinions of other people saying “three months” but what do you say?
208. *R: it should be every month.*
209. I: What makes you think that way?
210. *R: So that you should know if the cancer cells have started or not because if you take long to be screened, you will be screened when the cancer has spread and it will be difficult to manage.*
211. I: Fine. Now I want to hear your commendations for future screening campaigns; What do women in your community think about cervical cancer screening?
212. *R: many women are willing to be screened. “They removed your uterus? That is a good idea, it means they have removed the problem.” They are willing and some are coming to the hospital to be screened.*
213. I: What else can you share?
214. *R: That is all.*
215. I: Do you think women in your area understand the importance of cervical cancer screening?
216. *R: Yes.*
217. I: What makes you think that way?
218. *R: Because they have seen how people are suffering with cervical cancer.*
219. I: Perhaps have they experienced it or they just hear people talking about it?
220. *R: They have experienced it and they know that if they delay, cancer cannot be managed. That is why they are going for screening so that they should be seen before the cancer gets established.*
221. I: In your opinion, do you think that women are interested in receiving this screening and treatment service?
222. *R: They have the interest.*
223. I: What makes you feel that way?
224. *R: Because they do explain.*
225. I: What do they explain?
226. *R: They say that “I should go for screening so that I know my status in terms of cancer.”*
227. I: Why do you think someone would not want to be screened for cervical cancer?
228. *R: Laziness.*
229. I: What else?
230. *R: Lack of knowledge about cervical cancer.*
231. I: What else?
232. *R: That is all I know.*
233. I: Fine. So What are some of the barriers that women might face in receiving this service?
234. *R: There can be no barriers.*
235. I: There are no barriers?
236. *R: Yes.*
237. I: Maybe what barriers can be from their partners?
238. *R: Because some men say that “That is none of my business. I am not the one suffering and I don’t want to go to the hospital.” Some say that “Yes, go there and get screened so that we should know if you have it or not.” Some say that. Some don’t take it seriously, some believe it is real.*
239. I: Okay. What barriers can be from mere friends?
240. *R: Some can prevent them from going but some can encourage them to go.*
241. I: How can they prevent them from going?
242. *R: “Why don’t you stay here? Why going to the hospital as if you are sick? What are you going to do there?”*
243. I: Okay. Who else can prevent the women from going for cancer screening?
244. *R: What?*
245. I: Apart from male partners, friends, who else can prevent the women from going for cervical cancer screening?
246. *R: Maybe their grand parents or their mothers…*
247. I: Okay, relatives?
248. *R: Yes.*
249. I: Fine.
250. *R: “Don’t go there. Why going to the hospital as if you are sick, why don’t you just stay?” I want to go for cervical cancer screening.” “Don’t go.” That can be a relative, a child…*
251. I: Okay. In your opinion, how should cervical cancer screening be provided to ensure that more women can get screened?
252. *R: Like the procedure?*
253. I: How should cervical cancer screening be provided to ensure that more women can get screened?
254. *R: The way it is being done; people should be coming to the hospital for screening because the health workers cannot be screening people from their homes; those who want to be screened should come to the hospital.*
255. I: Okay, fine. Do you have anything to add?
256. *R: No.*
257. I: How would you encourage women to go for screening?
258. *R: I would encourage them.*
259. I: How would you encourage them?
260. *R: I would tell them that there is nothing you should fear, look at me, I had cancer but now it is history. Let me be your living example.*
261. I: Okay, fine. Now I want us to talk about self-collecting vaginal swabs; Let’s now discuss about self-collected vaginal swab for cervical cancer screening. A new method has been developed for cervical cancer screening. It involves having a woman collect a swab from her vagina and submitting it at her convenience to a health facility for testing.
262. *R: Okay?*
263. I: Yes. However, unlike VIA, the woman would not get her result immediately and would have to return to health facility to get her result a few hours later or the next day.
264. *R: Can I be collecting the swabs with my condition?*
265. I: No, you should answer on behalf of other people.
266. *R: Okay.*
267. I: What is your opinion on this method?
268. *R: I can encourage them because I have never heard that people are using vaginal swabs to screen cervical cancer. I can be telling them that “there is another method whereby you are supposed to do this” but I have never heard about it before. I have never seen people collecting vaginal discharge to submit to the doctor, I am hearing this now.*
269. I: But do you think this is an effective way of screening for cervical cancer?
270. *R: It is a good method.*
271. I: How?
272. *R: So that they should be able to see if the vaginal discharge has traits of cancer cells. It is good unlike just staying and cleaning it; you may not know if you have the problem because I hear that some women produce more vaginal discharge.*
273. I: If you were able to; like if they had not removed your uterus, would you be willing to be screened for cervical cancer using this method?
274. *R: Had it been that I heard about this method, I would have accepted to be screened using this method because it is easy and straight forward since you are collecting the swab yourself and you just give it to the doctor to do the tests. It is unlike going there to be screened but you just collect it and show them “this is my discharge,” and they do the tests and give you the results. So you know whether you have the cancer cells or not.*
275. I: How reliable is this method?
276. *R: It is easy and simple.*
277. I: It is simple?
278. *R: Yes.*
279. I: How unreliable is it?
280. *R: [Participant seemed to be tired]… It is reliable.*
281. I: Don’t worry we are about to finish.
282. *R: Okay.*
283. I: How does the idea of self-collection compare to the VIA screening you went through?
284. *R: Self-collection is simple. So you can choose from the two.*
285. I: So how do you differentiate the two?
286. *R: The best is to collect the swab and show them.*
287. I: Why?
288. *R: Because some are ashamed to be seen by doctors; “Why should I show my vagina to the doctor as if I am giving birth? Why should I show my vagina to a male doctor?” They are ashamed.*
289. I: Okay, they are ashamed?
290. *R: Yes, they are.*
291. I: What do you think other women in your community would think about the self-collected vaginal swab technique for screening?
292. *R: If this method can be explained to them that “There is another simple method where you simply collect a vaginal swab yourself and bring it to the hospital…*
293. I: So what can they think?
294. *R: They can be willing to self-collect the swab unlike the VIA method because they are ashamed.*
295. I: What else would they think?
296. *R: That is all.*
297. I: So, do you think more women would undergo screening with this method?
298. *R: Yes, they can choose it because it is a good method.*
299. I: Okay. What difficulties would women face in self-collection technique?
300. *R: They can have no difficulties.*
301. I: What are some reasons if any why you think women would not want to self-collect?
302. *R: They can still be ashamed.*
303. I: Like they are collecting on their own…
304. *R: Like “Should I collect my vaginal discharge and take it to the hospital?”*
305. I: Fine. Is there another reason?
306. *R: No.*
307. I: Okay. So, finally, let’s talk about your recommendations for the future of the National cervical cancer screening in Malawi: In your opinion, should MOH consider including self-collected vaginal swab for cervical cancer testing to the cervical cancer screening programme?
308. *R:[No response]*
309. I: This method that we were talking about, should it be considered as one of the methods for cervical cancer testing to the cervical cancer screening programme?
310. *R: There should not be one method.*
311. I: When you go there, like what happens with family planning; you choose whether to use DEPO, pills, Norplant and all that. Now in the same way, apart from VIA which was used to screen you, should self-collected vaginal swab be considered as a way for cancer screening in Malawi? Should it be included to the cervical cancer screening programme here in Malawi? What is your opinion.
312. *R: There should be two methods.*
313. I: What makes you think that way?
314. *R: People should have a choice; whether to choose the simple method or the VIA method. This is a simple method because people are afraid “Oh, it is painful, they pull out the uterus, they insert metals…” The self-collected vaginal swab would be the simplest method.*
315. I: So which groups of women could be most suitable for self-collected vaginal swab for cervical cancer testing?
316. *R: Young ladies.*
317. I: Why young ladies?
318. *R: They are the ones who are sexually active than old women.*
319. I: Is there another reason why you think that way?
320. *R: No.*
321. I: Apart from young women, who else could be most suitable for self-collected vaginal swab for cervical cancer testing?
322. *R: Like women of my age, a bit older than me, or younger than me, they can be screened.*
323. I: Which groups of women would not be most suitable for self-collected vaginal swab for cervical cancer testing?
324. *R: Children or old women. They would say “At my age, why should I do this?”*
325. I: Okay. So these are all the questions I had. I wonder if you have questions for me or comments on what we have discussed?
326. *R: No, I have no question.*
327. I: I thank you for your time. Your time is vital, we understand that you have to go elsewhere for other services but that you spared your time to meet and discuss with me, I do not take it for granted. This marks the need of our discussion.

End of interview
